# Supplementary material for: Fluorocarbonylation via palladium/phosphine synergistic catalysis
Source: Nat Commun. 2023 Jul 31;14:4583. doi: 10.1038/s41467-023-40180-6 (PMC10390470; doi:10.1038/s41467-023-40180-6)
Supplement: Supplementary file 1 — Supplementary Information [file 41467_2023_40180_MOESM1_ESM.pdf]

Supplementary Information for

Fluorocarbonylation via Palladium/Phosphine Synergistic Catalysis

Mingxin Zhao[+], Miao Chen[+], Tian Wang, Suhan Yang, Qian Peng,* and Pingping Tang*

[+]These authors contributed equally: Mingxin Zhao, Miao Chen.

*To whom correspondence should be addressed. E-mail: ptang@nankai.edu.cn, qpeng@nankai.edu.cn.

Table of Contents

Supplementary Notes S4
Supplementary Methods S5
General Procedure A S5
General Procedure B S5
General Procedure C S6
General Procedure D S6
4-Fluoro-*N*-(phenylmethyl)-benzamide (3a) S7
3-Fluoro-*N*-(phenylmethyl)-benzamide (3b) S7
4-Chloro-*N*-(phenylmethyl)-benzamide (3c) S8
3,5-Difluoro-*N*-(phenylmethyl)-benzamide (3d) S8
2-Methyl-*N*-(phenylmethyl)-benzamide (3e) S9
1-Methyl-*N*-(phenylmethyl)-1*H*-pyrazole-5-carboxamide (3f) S9
N-(Phenylmethyl)-1,3-benzodioxole-5-carboxamide (3g) S10
4-Methyl-*N*-(phenylmethyl)-benzamide (3h) S10
4-Methoxy-*N*-(phenylmethyl)benzamide (3i) S11
2, 4-Dimethyl-*N*-(phenylmethyl)benzamide (3j) S11
N-(phenylmethyl)[1,1'-biphenyl]-4-carboxamide (3k) S12
(1,1'-Biphenyl)-4-carbonyl fluoride (2k) S12
N-(phenylmethyl)[1,1'-biphenyl]-2-carboxamide (3l) S13
3-Methoxy-*N*-(phenylmethyl)benzamide (3m) S13
4-Acetyl-*N*-(phenylmethyl)benzamide (3n) S14
2,3-Methyl-*N*-(phenylmethyl)-benzamide (3o) S14
4-Nitro-*N*-(phenylmethyl)-benzamide (3p) S15
4-Cyano-*N*-(phenylmethyl)-benzamide (3q) S15
N-(Phenylmethyl)-2-naphthalenecarboxamide (3r) S16
N-(Phenylmethyl)benzamide (3s) S16
N-(phenylmethyl)-2-(trifluoromethoxy)-benzamide (3t) S17
N-(phenylmethyl)-2-(trifluoromethoxy)-benzamide (3u) S18
3,4,5-Trifluoro-*N*-(phenylmethyl)-benzamide (3v) S18
N-benzyl-3-phenylpropanamide (3w) S19
N-benzyl-4-phenylbutanamide (3x) S19
N-benzyl-5-phenylpentanamide (3y) S20
N-benzyl-4-(4-fluorophenoxy)butanamide (3z) S20
N-benzyl-4-phenoxybutanamide (3aa) S21
N-benzyl-4-(4-chlorophenoxy)butanamide (3ab) S21
N-benzyl-4-(4-bromophenoxy)butanamide (3ac) S22
N-benzyl-4-(4-cyanophenoxy)butanamide (3ad) S22
N-benzyl-4-(4-(benzyloxy)phenoxy)butanamide (3ae) S23
N-benzyl-4-(5-methoxy-2-methyl-3-(2-methoxy-2-oxoethyl)-1*H*-indole-1-

Scarbonyl)benzamide (3af) S24
N-benzyl-4-(4-((1-isopropoxy-2-methyl-1-oxopropan-2-yl)oxy)benzoyl)benzamide (3ag) S24
Gibberellic acid derivative (3ah)....S25
Triclosan derivative (3ai) S26
Deoxycholic acid derivative (2aj) S26
Estrone derivative (2ak) S27
Potassium trifluoro(phenethyl)-borane (1w) S28
Potassium trifluoro(3-phenylpropyl)-borane (1x) S28
Potassium trifluoro(4-phenylbutyl)-borane (1y) S29
Potassium trifluoro(3-(4-fluorophenoxy)propyl)-borane (1z)....S30
Potassium trifluoro(3-phenoxypropyl)-borane (1aa)....S31
Potassium trifluoro(3-(4-chlorophenoxy)propyl)-borane (1ab) S31
Potassium trifluoro(3-(4-bromophenoxy)propyl)-borane (1ac) S32
Potassium trifluoro(3-(4-cyanophenoxy)propyl)borane (1ad) S33
Potassium trifluoro(3-(4-(benzyloxy)phenoxy)propyl)-borane (1ae) S34
Synthesis of 4-(5-methoxy-2-methyl-3-(2-methoxy-2-oxoethyl)-1*H*-indole-1-carbonyl)phenyltrifluoroboric acid potassium salt (1af) S35
Synthesis of 4-(4-((1-isopropoxy-2-methyl-1-oxopropan-2-yl)oxy)benzoyl) phenyltrifluoroboric acid potassium salt (1ag)....S37
Gibberellic acid derivative (1ah)....S38
Potassium trifluoro(4-(2-(5-chloro-2-(3,5-dichlorophenoxy)phenoxy)-2-oxoethyl)phenyl)borane (1ai)....S40
Deoxycholic acid derivative (1aj) S41
Estrone derivative (1ak) S43
Synthesis of Pd(II)-OCF_3 complex (5a) S44
Synthesis of dicyclohexyldifluoro(2',4',6'-triisopropyl-3,6-dimethoxy-(1,1'-biphenyl)-2-yl)-λ^5-phosphane (7)....S44
Synthesis of (5-(diphenylphosphaneyl)-9,9-dimethyl-9H-xanthen-4-yl)difluorodiphenyl-λ^5-phosphane (L5)....S45
Supplementary Discussion....S46
Effect of Pd catalyst on the reaction....S46
Effect of solvent on the reaction S46
Effect of AgF amount on the reaction S47
Effect of P ligand on the reaction....S48
CO Detection Experiment....S49
Isotope Labelling Experiment....S50
^{19}F NMR Monitoring Experiment....S51
Computational studies....S51
a.Computational Methods S51
b.The comparison of the direct reductive elimination to β-F elimination S52
c.Other Transition States S53
d. Computed Energies of the Stationary Points....S57
Crystallographic Data....S58

X-ray Crystal Structure Data for Pd(II)-OCF3 complex (5a) (CCDC: 2099433) .S58
X-ray Crystal Structure Data for dicyclohexyldifluoro(2',4',6'-triisopropyl-3,6-dimethoxy-(1,1'-biphenyl)-2-yl)-λ^5-phosphane (7) (CCDC: 2099395).................S67
X-ray Crystal Structure Data for (5-(diphenylphosphaneyl)-9,9-dimethyl-9H-xanthen-4-yl)difluorodiphenyl-λ^5-phosphane (L5) (CCDC: 2243163).................S75
Supplementary References..S155

Supplementary Notes

All reactions were conducted in oven-dried glassware under an atmosphere of nitrogen unless otherwise noted. EA, Acetone, DCE, DMA, 1, 4-dioxane, Et_2O, DMSO and DCM were dried by distillation over CaH_2. MeCN used in reactions were dried by stirring over CaH_2 (5%w/v) overnight, and fractional distillation, then bubbled in argon balloon for 0.5 h, THF, dioxane and toluene were dried by distillation over sodium/benzophenone. $CDCl_3$ was purchased from Sigma-Aldrich. AgF was purchased from J&K. OCF_3 reagents were prepared according to the reported literatures.[1] Cesium fluoride was dried at 120 °C under reduced pressure from TCI. TLC was performed on silica gel Huanghai HSGF254 plates and visualized by quenching of UV fluorescence (λ_{max}= 254 nm). 200-300 mesh silica gel was purchased from Qingdao Haiyang Chemical Co., China. Unless otherwise noted, all other reagents and starting materials were purchased from commercial sources and used without further purification. The data for NMR spectra (^{1}H NMR, ^{13}C NMR and ^{19}F NMR) were recorded at 293 K on a Bruker AVANCE AV 400 (400 MHz, 101 MHz and 376 MHz) and chemical shifts were recorded relative to the solvent resonance. Signal positions were recorded in ppm and the following abbreviations are used singularly or in combination to indicate the multiplicity of signals: s singlet, d doublet, t triplet, q quartet, m multiplet, Hz Hertz. For ^{1}H NMR: $CDCl_3$ = δ 7.26 ppm. For ^{13}C NMR: $CDCl_3$ = δ 77.16 ppm. Mass spectra were obtained on Agilent 6520 Q-TOF LC/MS and Aligent 7890/5975C-GS/MSD. HRMS were obtained on VG ZAB-HS(ESI), Thermo Fisher Q-Exactive Orbitrap(ESI) and GCT Premier(EI). GCMS analysis was performed on an Aligent 7890/5975C-GS/MSD. HRMS were obtained on VG ZAB-HS(ESI), Thermo Fisher Q-Exactive Orbitrap(ESI) and GCT Premier(EI). GCMS analysis was performed on an Aligent 7890/5975C-GS/MSD. The product was separated on a 30 m length by 0.25 mm id Hp-5MS column coated with a 0.25 μm film. Reverse phase preparatory HPLC separation were performed on Shimazu HPLC system (Pump model: LC-20AP, detector model: SPD-M20A) and was used a Innoval ODS-2 21.2×250 mm, 10 *μm* column packing with acetonitrile/water as an eluent. Single crystal X-ray diffraction data were collected on Rigaku Saturn70 diffractometer at 113(2) K (for compound 3t) with Mo-Kα radiation (λ = 0.71073 Å) and SCX-Mini diffractometer at 293(2) K (for compound 4kk and compound 4ss) with Mo-Kα radiation (λ = 0.71075 Å). ^{19}F NMR spectrometer used standard pulse sequences with 16 scans and an optimized relaxation delays (d1= 20 s for ^{19}F NMR).Carbon monoxide detection tube was purchased from Hebi Huaan Gas Detection Technology Co., Ltd. China.

Supplementary Methods

General Procedure A

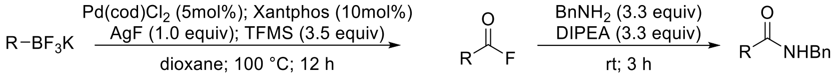

1 2 3

In a N_2 glovebox, to aryl/alkyl fluoroboric acid potassium salt (**1**) (0.25 mmol, 1.00 equiv.), Pd(cod)Cl_2 (3.5 mg, 0.0125 mmol, 0.05 equiv.), AgF (31.7 mg, 0.25 mmol, 1.00 equiv.) and Xantphos (14.5 mg, 0.025mmol, 0.10 equiv.) in a 4.00 mL sealed vial tube were added 1,4-dioxane (2.00 mL) and TFMS (trifluoromethyl 4-fluorobenzenesulfonate) (140.0 µL, 0.875 mmol, 3.50 equiv.). Then the sealed vial was taken outside the glovebox and the reaction mixture was stirred for 12 h at 100 °C.

The system was cooled to room temperature, bubbled with argon for 10 minutes, and added $BnNH_2$ (88.4 mg, 0.825 mmol, 3.3 equiv.) and diisopropylethylamine (DIPEA) (106.6 mg, 0.825 mmol, 3.3 equiv.), the reaction mixture was stirred for 3 h at room temperature. The system was filtered and concentrated *in vacuo*. The residue was purified by preparative TLC eluting with *n*-hexane/EA.

General Procedure B

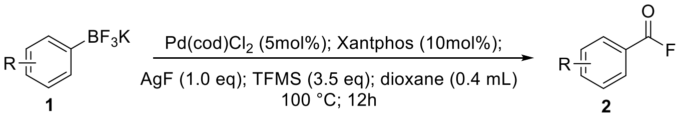

1 2

In a N_2 glovebox, to aryl fluoroboric acid potassium salt (**1**) (0.25 mmol, 1.00 equiv.), Pd(Cod)Cl_2 (3.5 mg, 0.0125 mmol, 0.05 equiv.), AgF (31.7 mg, 0.25 mmol, 1.00 equiv.) and Xantphos (14.5 mg, 0.025mmol, 0.10 equiv.) in a 4.00 mL sealed vial tube were added 1,4-dioxane (2.00 mL) and TFMS (trifluoromethyl 4-fluorobenzenesulfonate) (140.0 µL, 0.875 mmol, 3.50 equiv.). Then the sealed vial was taken outside the glovebox and the reaction mixture was stirred for 12 h at 100 °C.

The system was filtered and concentrated *in vacuo*. The residue was purified by preparative HPLC with acetonitrile/water as an eluent.

General Procedure C

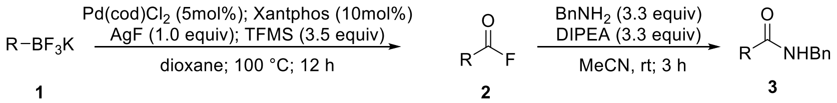

In a N_2 glovebox, to aryl/alkyl fluoroboric acid potassium salt (**1**) (0.10 mmol, 1.00 equiv.), Pd(cod)Cl_2 (1.4 mg, 0.005 mmol, 0.05 equiv.), AgF (31.7 mg, 0.25 mmol, 1.00 equiv.) and Xantphos (5.80 mg, 0.010mmol, 0.10 equiv.) in a 4.00 mL sealed vial tube were added 1,4-dioxane (0.80 mL) and TFMS (trifluoromethyl 4-fluorobenzenesulfonate) (64.0 µL, 0.40 mmol, 4.0 equiv.). Then the sealed vial was taken outside the glovebox and the reaction mixture was stirred for 12 h at 100 °C. The system was cooled to room temperature, and was concentrated in *vacuo*. The system was dissolved in MeCN, $BnNH_2$ (88.4 mg, 0.825 mmol, 3.3 equiv.) and diisopropylethylamine (DIPEA) (106.6 mg, 0.825 mmol, 3.3 equiv.) was added in three portions over three hours at room temperature. The system was filtered and concentrated *in vacuo*. The residue was purified by preparative TLC eluting with *n*-hexane/EA.

General Procedure D

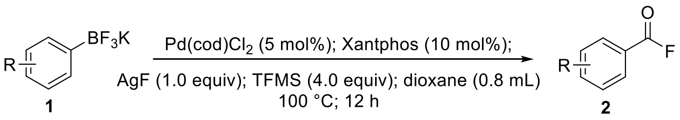

In a N_2 glovebox, to aryl fluoroboric acid potassium salt (**1**) (0.10 mmol, 1.00 equiv.) Pd(cod)Cl_2 (1.4 mg, 0.005 mmol, 0.05 equiv.), AgF (31.7 mg, 0.25 mmol, 1.00 equiv.) and Xantphos (5.80 mg, 0.010mmol, 0.10 equiv.) in a 4.00 mL sealed vial tube were added 1,4-dioxane (0.80 mL) and TFMS (trifluoromethyl 4-fluorobenzenesulfonate) (64.0 µL, 0.40 mmol, 4.0 equiv.). Then the sealed vial was taken outside the glovebox and the reaction mixture was stirred for 12 h at 100 °C. The system was cooled to room temperature, and was concentrated in *vacuo*. The residue was purified by preparative HPLC with acetonitrile/water as an eluent.

4-Fluoro-*N*-(phenylmethyl)-benzamide (3a)

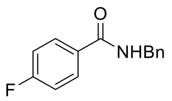

Following the general procedure A, using 4-fluorophenyltrifluoroboric acid potassium salt (**1a**) (50.5 mg, 0.25 mmol). Purification by preparative TLC eluting with *n*-hexane/EA afforded **3a** as a white solid (47.0 mg, 82%). The ^{19}F NMR yield of the volatile 4-fluorobenzoyl fluoride (**2a**) was determined by comparing the integration of the ^{19}F NMR resonance of 4-fluorobenzoyl fluoride (17.12 ppm) with that of tribromofluoromethane (4.04 ppm). (99% ^{19}F NMR Yield).

R_f = 0.30 [*n*-hexane : EA = 3 : 1(v/v)]. NMR Spectroscopy: ^{1}H NMR (400 MHz, $CDCl_3$) δ 7.79-7.75 (m, 2H), 7.38-7.19 (m, 5H), 7.05-7.01 (t, *J* = 8.4 Hz, 2H), 6.87 (m, 1H), 4.56 (d, *J* = 6.0 Hz, 2H); ^{13}C NMR (101 MHz, $CDCl_3$) δ 166.4, 164.7 (d, $J_{C\text{-}F}$ = 253.5 Hz), 138.2, 130.5 (d, $J_{C\text{-}F}$ = 3.0 Hz), 129.4 (d, $J_{C\text{-}F}$ = 9.1 Hz), 128.8, 127.8, 127.6, 115.5 (d, $J_{C\text{-}F}$ = 22.2 Hz), 44.1; ^{19}F NMR (376 MHz, $CDCl_3$): δ = -108.0 - -108.2 (m, 1F).

The spectroscopic data matched those reported in the literature. [1]

3-Fluoro-*N*-(phenylmethyl)-benzamide (3b)

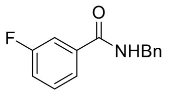

Following the general procedure A, using 3-fluorophenyltrifluoroboric acid potassium salt (**1b**) (50.5 mg, 0.25 mmol). Purification by preparative TLC eluting with *n*-hexane/EA afforded **3b** as a white solid (36.6 mg, 64%). The ^{19}F NMR yield of the volatile 3-fluorobenzoyl fluoride (**2b**) was determined by comparing the integration of the ^{19}F NMR resonance of 3-fluorobenzoyl fluoride (18.23 ppm) with that of tribromofluoromethane (4.04 ppm). (81% ^{19}F NMR Yield).

R_f = 0.30 [*n*-hexane : EA = 3 : 1(v/v)].NMR Spectroscopy: ^{1}H NMR (400 MHz, $CDCl_3$) δ 7.58-7.51 (m, 2H), 7.42-7.27 (m, 6H), 7.24-7.14 (m, 2H), 6.97-6.74 (m, 1H), 4.60 (d, *J* = 5.2 Hz, 2H); ^{13}C NMR (101 MHz, $CDCl_3$) δ 166.3, 162.7 (d, $J_{C\text{-}F}$ = 248.5 Hz), 137.9, 136.6 (d, $J_{C\text{-}F}$ = 7.1 Hz), 130.2 (d, $J_{C\text{-}F}$ = 8.1 Hz), 128.8, 127.9, 127.7, 122.5, 118.5 (d, $J_{C\text{-}F}$ = 21.2 Hz), 114.5 (d, $J_{C\text{-}F}$ = 22.2 Hz), 44.1; ^{19}F NMR (376 MHz, $CDCl_3$): δ = -111.7- -111.8 (m, 1F).

The spectroscopic data matched those reported in the literature. [2]

4-Chloro-*N*-(phenylmethyl)-benzamide (3c)

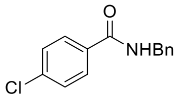

Following the general procedure A, using 4- chlorophenyltrifluoroboric acid potassium salt (**1c**) (54.6 mg, 0.25 mmol). Purification by preparative TLC eluting with *n*-hexane/EA afforded **3c** as a white solid (45.8 mg, 75%). The ^{19}F NMR yield of the volatile 4-chlorobenzoyl fluoride (**2c**) was determined by comparing the integration of the ^{19}F NMR resonance of 4-chlorobenzoyl fluoride (17.44 ppm) with that of tribromofluoromethane (4.04 ppm). (92% ^{19}F NMR Yield).

R_f = 0.18 [*n*-hexane : EA = 3 : 1(v/v)]. NMR Spectroscopy: ^{1}H NMR (400 MHz, $CDCl_3$) δ 7.75-7.68 (m, 2H), 7.40-7.28 (m, 7H), 6.68-6.54 (m, 1H), 4.60 (d, *J* = 5.6 Hz, 2H); ^{13}C NMR (101 MHz, $CDCl_3$) δ 166.4, 138.0, 137.8, 132.7, 128.8, 128.5, 127.9, 127.7, 44.2.

The spectroscopic data matched those reported in the literature. [1]

3,5-Difluoro-*N*-(phenylmethyl)-benzamide (3d)

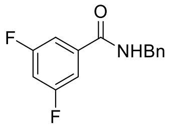

Following the general procedure A, using 3,5-difluorophenyltrifluoroboric acid potassium salt (**1d**) (55.0 mg, 0.25 mmol). Purification by preparative TLC eluting with *n*-hexane/EA afforded **3d** as a white solid (24.1 mg, 39%). The ^{19}F NMR yield of the volatile 3,5-difluorobenzoyl fluoride (**2d**) was determined by comparing the integration of the ^{19}F NMR resonance of 3,5-difluorobenzoyl fluoride (17.39 ppm) with that of tribromofluoromethane (4.04 ppm). (53% ^{19}F NMR Yield).

R_f = 0.40 [*n*-hexane : EA = 3 : 1(v/v)]. NMR Spectroscopy: ^{1}H NMR (400 MHz, $CDCl_3$) δ 7.40-7.28 (m, 7H), 6.95 (ddd, *J* = 8.6, 6.2, 2.3 Hz, 2H), 6.80-6.68 (m, 1H), 4.60 (d, *J* = 5.6 Hz, 2H); ^{13}C NMR (101 MHz, $CDCl_3$) δ 165.06, 165.03, 164.2 (d, $J_{C\text{-}F}$ = 12.1 Hz), 161.7 (d, $J_{C\text{-}F}$ =

12.1 Hz), 137.6, 128.9, 127.9, 127.8, 110.5, 110.4, 110.3, 110.2, 106.92 (t, J = 25.2 Hz), 44.3; ^{19}F NMR (376 MHz, $CDCl_3$): δ = -108.0 (t, J = 7.6 Hz); HRMS-ESI (m/z): Calcd for $C_{14}H_{11}F_2NNaO^+$ [M + Na]$^+$, 270.0701. Found, 270.0701.

2-Methyl-*N*-(phenylmethyl)-benzamide (3e)

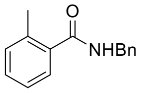

Following the general procedure A, using 2-methylphenyltrifluoroboric acid potassium salt (**1e**) (49.5 mg, 0.25 mmol). Purification by preparative TLC eluting with *n*-hexane/EA afforded **3e** as a white solid (26.9 mg, 48%). The ^{19}F NMR yield of the volatile 2-methylbenzoyl fluoride (**2e**) was determined by comparing the integration of the ^{19}F NMR resonance of 2-methylbenzoyl fluoride (28.49 ppm) with that of tribromofluoromethane (4.04 ppm). (50% ^{19}F NMR Yield).

R_f = 0.30 [*n*-hexane : EA = 3 : 1(v/v)]. NMR Spectroscopy: ^{1}H NMR (400 MHz, $CDCl_3$) δ 7.42-7.26 (m, 7H), 7.25-7.16 (m, 2H), 6.35-6.03 (m, 1H), 4.62 (d, J = 5.6 Hz, 2H); ^{13}C NMR (101 MHz, $CDCl_3$) δ 169.94, 138.25, 136.27, 136.19, 131.05, 129.94, 128.79, 127.85, 127.60, 126.69, 125.73, 43.90, 19.86.

The spectroscopic data matched those reported in the literature. [3]

1-Methyl-*N*-(phenylmethyl)-1*H*-pyrazole-5-carboxamide (3f)

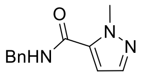

Following the general procedure A, using trifluoro(1-methyl-1H-pyrazol-5-yl)boric acid potassium salt (**1f**) (47.0 mg, 0.25 mmol). Purification by preparative TLC eluting with *n*-hexane/EA afforded **3f** as a white solid (28.3 mg, 53%). The ^{19}F NMR yield of the volatile 1-methyl-1*H*-pyrazole-5-carbonyl fluoride (**2f**) was determined by comparing the integration of the ^{19}F NMR resonance of 1-methyl-1*H*-pyrazole-5-carbonyl fluoride (25.73 ppm) with that of tribromofluoromethane (4.04 ppm). (60% ^{19}F NMR Yield).

R_f = 0.35 [*n*-hexane : EA = 1 : 3 (v/v)]. NMR Spectroscopy: ^{1}H NMR (400 MHz, $CDCl_3$) δ 7.76 (d, J = 24.8 Hz, 2H), 7.38-7.22 (m, 5H), 6.48 (s, 1H), 4.55 (d, J = 5.7 Hz, 2H), 3.86 (s,

3H); ^{13}C NMR (101 MHz, $CDCl_3$) δ 162.5, 138.4, 138.0, 131.9, 128.7, 127.8, 127.5, 118.6, 43.4, 39.3. HRMS-ESI (m/z): Calcd for $C_{12}H_{14}N_3O^+$ $[M + H]^+$, 216.1131. Found, 216.1131.

_N_-(Phenylmethyl)-1,3-benzodioxole-5-carboxamide (3g)

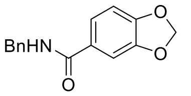

Following the general procedure A, using 3,4-benzodioxoletrifluoroboric acid potassium salt (**1g**) (57.0 mg, 0.25 mmol). Purification by preparative TLC eluting with *n*-hexane/EA afforded **3g** as a white solid (50.4 mg, 79%). The ^{19}F NMR yield of the volatile benzo[*d*][1,3]dioxole-5-carbonyl fluoride (**2g**) was determined by comparing the integration of the ^{19}F NMR resonance of benzo[*d*][1,3]dioxole-5-carbonyl fluoride (15.79 ppm) with that of tribromofluoromethane (4.04 ppm). (99% ^{19}F NMR Yield).

R_f = 0.20 [*n*-hexane : EA = 6 : 1(v/v)]. NMR Spectroscopy: ^{1}H NMR (400 MHz, $CDCl_3$) δ 7.40-7.23 (m, 7H), 6.83-6.64 (m, 2H), 6.00 (s, 2H), 4.60 (d, *J* = 5.6 Hz, 2H); ^{13}C NMR (101 MHz, $CDCl_3$) δ 166.8, 150.3, 147.9, 138.4, 128.7, 128.6, 127.8, 127.5, 121.7, 108.0, 107.7, 101.7, 44.1.

The spectroscopic data matched those reported in the literature. [4]

4-Methyl-_N_-(phenylmethyl)-benzamide (3h)

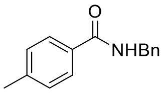

Following the general procedure A, using 4-methylphenyltrifluoroboric acid potassium salt (**1h**) (49.5 mg, 0.25 mmol). Purification by preparative TLC eluting with *n*-hexane/EA afforded **3h** as a white solid (51.6 mg, 92%). The ^{19}F NMR yield of the volatile 4-methylbenzoyl fluoride (**2h**) was determined by comparing the integration of the ^{19}F NMR resonance of 4-methylbenzoyl fluoride (16.58 ppm) with that of tribromofluoromethane (4.04 ppm). (99% ^{19}F NMR Yield).

R_f = 0.30 [*n*-hexane : EA = 3 : 1(v/v)]. NMR Spectroscopy: ^{1}H NMR (400 MHz, $CDCl_3$) δ 7.73 (d, *J* = 7.9 Hz, 2H), 7.40-7.26 (m, 5H), 7.20 (d, *J* = 7.9 Hz, 2H), 6.98-6.82 (m, 1H), 4.60

(d, J = 5.7 Hz, 2H), 2.40 (s, 3H); ^{13}C NMR (101 MHz, $CDCl_3$) δ 167.5, 141.9, 138.5, 131.6, 129.2, 128.7, 127.8, 127.4, 127.1, 44.0, 21.5.

The spectroscopic data matched those reported in the literature. [5]

4-Methoxy-*N*-(phenylmethyl)benzamide (3i)

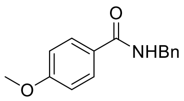

Following the general procedure A, using 4-methoxyphenyltrifluoroboric acid potassium salt (**1i**) (53.5 mg, 0.25 mmol). Purification by preparative TLC eluting with *n*-hexane/EA afforded **3i** as a white solid (45.5 mg, 75%). The ^{19}F NMR yield of the volatile 4-methoxybenzoyl fluoride (**2i**) was determined by comparing the integration of the ^{19}F NMR resonance of 4-methoxybenzoyl fluoride (15.37 ppm) with that of tribromofluoromethane (4.04 ppm). (99% ^{19}F NMR Yield).

R_f = 0.20 [*n*-hexane : EA = 3 : 1(v/v)]. NMR Spectroscopy: ^{1}H NMR (400 MHz, $CDCl_3$) δ 7.75 (d, J = 8.8 Hz, 1H), 7.36-7.21 (m, 5H), 6.86 (d, J = 8.8 Hz, 1H), 6.69 (s, 1H), 4.57 (d, J = 5.7 Hz, 1H), 3.81 (s, 3H); ^{13}C NMR (101 MHz, $CDCl_3$) δ 167.0, 162.2, 138.5, 128.9, 128.7, 127.8, 127.5, 126.7, 113.7, 55.4, 44.0.

The spectroscopic data matched those reported in the literature. [1]

2, 4-Dimethyl-*N*-(phenylmethyl)benzamide (3j)

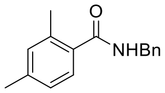

Following the general procedure A, using 2,4-dimethylphenyltrifluoroboric acid potassium salt (**1j**) (53.0 mg, 0.25 mmol). Purification by preparative TLC eluting with *n*-hexane/EA afforded **3j** as a white solid (31.5 mg, 53%). The ^{19}F NMR yield of the volatile 2, 4-dimethylbenzoyl fluoride (**2j**) was determined by comparing the integration of the ^{19}F NMR resonance of 2, 4-dimethylbenzoyl fluoride (27.50 ppm) with that of tribromofluoromethane (4.04 ppm). (64% ^{19}F NMR Yield).

R$_f$ = 0.40 [*n*-hexane : EA = 3 : 1(v/v)]. NMR Spectroscopy: ^{1}H NMR (400 MHz, $CDCl_3$) δ 7.44-7.24(m, 6H), 7.08-6.96 (m, 2H), 6.19 (s, 1H), 4.61 (d, *J* = 5.8 Hz, 1H), 2.45 (s, 1H), 2.35 (s, 1H); ^{13}C NMR (101 MHz, $CDCl_3$) δ 167.0, 140.0, 138.4, 136.3, 133.3, 131.9, 128.8, 127.8, 127.5, 126.8, 126.3, 77.4, 77.1, 76.8, 43.9, 21., 19.9; HRMS-ESI (m/z): Calcd for $C_{16}H_{17}NNaO^+$ $[M + Na]^+$, 262.1202. Found, 262.1202.

N-(phenylmethyl)[1,1'-biphenyl]-4-carboxamide (3k)

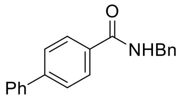

Following the general procedure A, using 2,4-dimethylphenyltrifluoroboric acid potassium salt (**1k**) (65.0 mg, 0.25 mmol). Purification by preparative TLC eluting with *n*-hexane/EA afforded **3k** as a white solid (65.3 mg, 90%).

R$_f$ = 0.30 [*n*-hexane : EA = 3 : 1(v/v)]. NMR Spectroscopy: ^{1}H NMR (400 MHz, $CDCl_3$) δ 7.90-7.84(m, 2H), 7.68-7.58 (m, 4H), 7.49-7.43 (m, 2H), 7.42-7.27 (m, 6H), 6.63-6.51 (m, 1H), 4.67 (d, *J* = 5.7 Hz, 2H); ^{13}C NMR (101 MHz, $CDCl_3$) δ 167.1, 144.4, 140.0, 138.24 133.0, 128.9, 128.8, 128.0, 128.0, 127.7, 127.5, 127.3, 127.2, 44.2.

The spectroscopic data matched those reported in the literature. [6]

(1,1'-Biphenyl)-4-carbonyl fluoride (2k)

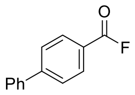

Following the general procedure B, using 2,4-dimethylphenyltrifluoroboric acid potassium salt (**1k**) (65.0 mg, 0.25 mmol).The reaction mixture was purified by preparative HPLC (10 mL/min, detector UV λ_{max} 210 nm, $MeCN/H_2O$ = 40/60 (0 min), $MeCN/H_2O$ = 60:40 (20 min), $MeCN/H_2O$ = 80:20 (40 min), $MeCN/H_2O$ = 100:0 (60 min)) to afford (1,1'-biphenyl)-4-carbonyl fluoride (**2k)** as white solid (42.0 mg, 84%).

NMR Spectroscopy: ^{1}H NMR (400 MHz, $CDCl_3$) δ 8.17-8.04 (m, 1H), 7.75 (d, *J* = 8.0 Hz, 1H), 7.68-7.60 (m, 1H), 7.56-7.41 (m, 2H); ^{13}C NMR (101 MHz, $CDCl_3$) δ 158.1, 154.6, 147.1,

138.2, 131.0, 130.9, 128.1, 127.8, 126.6, 126.3, 122.7, 122.1. ^{19}F NMR (376 MHz, $CDCl_3$) δ 18.14.

The spectroscopic data matched those reported in the literature. [9]

N-(phenylmethyl)[1,1'-biphenyl]-2-carboxamide (3l)

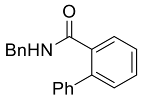

Following the general procedure A, using (4-phenyl)phenyltrifluoroboric acid potassium salt (**1l**) (65.0 mg, 0.25 mmol). Purification by preparative TLC eluting with *n*-hexane/EA afforded **3l** as a white solid (29.6 mg, 41%). The ^{19}F NMR yield of the volatile [1,1'-biphenyl]-2-carbonyl fluoride (**2l**) was determined by comparing the integration of the ^{19}F NMR resonance of [1,1'-biphenyl]-2-carbonyl fluoride (34.88 ppm) with that of tribromofluoromethane (4.04 ppm). (68% ^{19}F NMR Yield).

R_f = 0.30 [*n*-hexane : EA = 3 : 1(v/v)]. NMR Spectroscopy: ^{1}H NMR (400 MHz, $CDCl_3$) δ 7.73 (dd, J = 7.5, 1.6 Hz, 1H), 7.51-7.34(m, 8H), 7.24-7.15(m, 3H), 5.47 (s, 1H), 4.33 (d, J = 5.5 Hz, 2H). ^{13}C NMR (101 MHz, $CDCl_3$) δ 169.4, 140.2, 139.5, 137.4, 135.6, 130.2, 130.2, 128.9, 128.8, 128.7, 128.6, 127.8, 127.6, 127.4, 44.2.

The spectroscopic data matched those reported in the literature. [7]

3-Methoxy-N-(phenylmethyl)benzamide (3m)

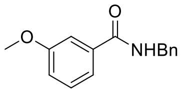

Following the general procedure A, using 3-methoxyphenyltrifluoroboric acid potassium salt (**1m**) (53.5 mg, 0.25 mmol). Purification by preparative TLC eluting with *n*-hexane/EA afforded **3m** as a colorless liquid (51.1 mg, 85%). The ^{19}F NMR yield of the volatile 3-methoxybenzoyl fluoride (**2m**) was determined by comparing the integration of the ^{19}F NMR resonance of 3-methoxybenzoyl fluoride (17.76 ppm) with that of tribromofluoromethane (4.04 ppm). (99% ^{19}F NMR Yield).

R_f = 0.35 [*n*-hexane : EA = 3 : 1(v/v)]. NMR Spectroscopy: ^{1}H NMR (400 MHz, $CDCl_3$) δ 7.40-7.35 (m, 1H); 7.34-7.23 (m, 7H), 7.04-6.97 (m, 1H), 6.93-6.77 (m, 1H), 4.57 (d, *J* = 5.7 Hz, 2H), 3.77 (s, 3H); ^{13}C NMR (101 MHz, $CDCl_3$) δ 167.4, 159.8, 138.3, 135.8, 129.6, 128.7, 127.8, 127.5, 118.8, 117.8, 112.4, 55.4, 44.1. HRMS-ESI (m/z): Calcd for $C_{15}H_{15}NNaO_2^+$ [M + Na]$^+$, 264.0995. Found, 264.0995.

4-Acetyl-*N*-(phenylmethyl)benzamide (3n)

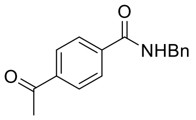

Following the general procedure A, using 4-acetylphenyltrifluoroboric acid potassium salt (**1n**) (56.5 mg, 0.25 mmol). Purification by preparative TLC eluting with *n*-hexane/EA afforded **3n** as a colorless liquid (49.1 mg, 78%). The ^{19}F NMR yield of the volatile 4-acetylbenzoyl fluoride (**2n**) was determined by comparing the integration of the ^{19}F NMR resonance of 4-acetylbenzoyl fluoride (18.81 ppm) with that of tribromofluoromethane (4.04 ppm). (85% ^{19}F NMR Yield).

R_f = 0.20 [*n*-hexane : EA = 3 : 1(v/v)]. NMR Spectroscopy: ^{1}H NMR (400 MHz, $CDCl_3$) δ 7.97-7.80 (m, 2H), 7.37-7.25 (m, 5H), 7.09-6.89(m, 1H), 4.60 (d, *J* = 5.7 Hz, 2H), 2.59 (s, 3H); ^{13}C NMR (101 MHz, $CDCl_3$) δ 197.5, 166.5, 139.1, 138.3, 137.9, 128.8, 128.5, 127.9, 127.7, 127.4, 44.2, 26.8.

The spectroscopic data matched those reported in the literature. [8]

2,3-Methyl-*N*-(phenylmethyl)-benzamide (3o)

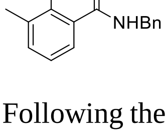

Following the general procedure A, using 2,3-dimethylphenyltrifluoroboric acid potassium salt (**1o**) (53.0 mg, 0.25 mmol). Purification by preparative TLC eluting with *n*-hexane/EA afforded **3o** as a white solid (30.6 mg, 51%). The ^{19}F NMR yield of the volatile 2,3-dimethylbenzoyl fluoride (**2o**) was determined by comparing the integration of the ^{19}F NMR resonance of 2,3-dimethylbenzoyl fluoride (32.13 ppm) with that of tribromofluoromethane (4.04 ppm). (52%

^{19}F NMR Yield).

R_f = 0.40 [*n*-hexane : EA = 3 : 1(v/v)]. NMR Spectroscopy: ^{1}H NMR (400 MHz, $CDCl_3$) δ 7.41-7.28(m, 5H), 7.23-7.15 (m, 2H), 7.14-7.07 (m, 1H), 6.26 (s, 1H), 4.61 (d, *J* = 5.8 Hz, 2H), 2.32 (d, *J* = 10.0 Hz, 6H); ^{13}C NMR (101 MHz, $CDCl_3$) δ 170.7, 138.3, 137.9, 137.1, 134.2, 131.2, 128.8, 127.8, 127.6, 125.5, 124.3, 43.9, 20.3, 16.3.; HRMS-ESI (m/z): Calcd for $C_{16}H_{17}NNaO^+$ [M + Na]$^+$, 262.1202. Found, 262.1202.

4-Nitro-*N*-(phenylmethyl)-benzamide (3p)

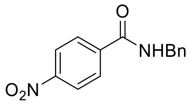

Following the general procedure A, using 4-nitrophenyltrifluoroboric acid potassium salt (**1p**) (57.3 mg, 0.25 mmol). Purification by preparative TLC eluting with *n*-hexane/EA afforded **3p** as a white solid (35.1 mg, 55%). The ^{19}F NMR yield of the volatile 4-nitrobenzoyl fluoride (**2p**) was determined by comparing the integration of the ^{19}F NMR resonance of 4-nitrobenzoyl fluoride (18.53 ppm) with that of tribromofluoromethane (4.04 ppm). (61% ^{19}F NMR Yield).

R_f = 0.30 [*n*-hexane : EA = 3 : 1(v/v)]. NMR Spectroscopy: ^{1}H NMR (400 MHz, $CDCl_3$) δ 8.20 (d, *J* = 8.3 Hz, 1H), 8.02-7.82 (m, 2H), 7.41-7.26 (m, 5H), 7.01 (s, 1H), 4.61 (d, *J* = 5.8 Hz, 1H); ^{13}C NMR (101 MHz, $CDCl_3$) δ 165.5, 149.5, 139.9, 137.5, 128.9, 128.3, 127.9, 123.8, 44.4.

The spectroscopic data matched those reported in the literature. [1]

4-Cyano-*N*-(phenylmethyl)-benzamide (3q)

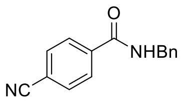

Following the general procedure A, using 4-cyanophenyltrifluoroboric acid potassium salt (**1q**) (57.3 mg, 0.25 mmol). Purification by preparative TLC eluting with *n*-hexane/EA afforded **3q** as a white solid (35.1 mg, 55%). The ^{19}F NMR yield of the volatile 4-cyanobenzoyl fluoride

(**2q**) was determined by comparing the integration of the ^{19}F NMR resonance of 4-cyanobenzoyl fluoride (17.40 ppm) with that of tribromofluoromethane (4.04 ppm). (66% ^{19}F NMR Yield).

R_f = 0.30 [*n*-hexane : EA = 3 : 1(v/v)]. NMR Spectroscopy: ^{1}H NMR (400 MHz, $CDCl_3$) δ 7.77 (dd, *J* = 85.8, 8.1 Hz, 4H), 7.3-7.28 (m, 5H), 7.13-6.96 (m, 1H), 4.61 (d, *J* = 5.7 Hz, 2H); ^{13}C NMR (101 MHz, $CDCl_3$) δ 165.7, 138.2, 137.6, 132.4, 128.9, 127.8, 127.8, 118.1, 115.0, 44.3. The spectroscopic data matched those reported in the literature. [1]

N-(Phenylmethyl)-2-naphthalenecarboxamide (3r)

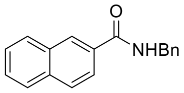

Following the general procedure A, using 2-naphthyltrifluoroboric acid potassium salt (**1r**) (58.5 mg, 0.25 mmol). Purification by preparative TLC eluting with *n*-hexane/EA afforded **3r** as a white solid (42.3 mg, 65%). The ^{19}F NMR yield of the volatile 2-naphthoyl fluoride (**2r**) was determined by comparing the integration of the ^{19}F NMR resonance of 2-naphthoyl fluoride (17.24 ppm) with that of tribromofluoromethane (4.04 ppm). (80% ^{19}F NMR Yield).

R_f = 0.20 [*n*-hexane : EA = 3 : 1(v/v)]. NMR Spectroscopy: ^{1}H NMR (400 MHz, $CDCl_3$) δ 8.28 (s, 1H), 7.95 – 7.71 (m, 4H), 7.63 – 7.41 (m, 2H), 7.35 – 7.20 (m, 5H), 6.94 (s, 1H), 4.63 (d, *J* = 5.7 Hz, 2H); ^{13}C NMR (101 MHz, $CDCl_3$) δ 167.6, 138.3, 134.8, 132.6, 131.6, 129.0, 128.8, 128.4, 128.0, 127.8, 127.7, 127.6, 127.6, 126.7, 123.7, 44.2.

The spectroscopic data matched those reported in the literature. [1]

N-(Phenylmethyl)benzamide (3s)

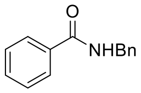

Following the general procedure A, using phenyltrifluoroboric acid potassium salt (**1s**) (46.0 mg, 0.25 mmol). Purification by preparative TLC eluting with *n*-hexane/EA afforded **3s** as a

white solid (40.0 mg, 76%). The ^{19}F NMR yield of the volatile benzoyl fluoride (**2s**) was determined by comparing the integration of the ^{19}F NMR resonance of benzoyl fluoride (17.19 ppm) with that of tribromofluoromethane (4.04 ppm). (99% ^{19}F NMR Yield).

R_f = 0.20 [*n*-hexane : EA = 3 : 1(v/v)]. NMR Spectroscopy: ^{1}H NMR (400 MHz, $CDCl_3$) δ 7.82 (d, *J* = 7.7 Hz, 2H), 7.51 (t, *J* = 7.3 Hz, 1H), 7.46 – 7.26 (m, 7H), 6.84 (s, 1H), 4.63 (d, *J* = 5.6 Hz, 2H); ^{13}C NMR (101 MHz, $CDCl_3$) δ 167.5, 138.3, 134.4, 131.5, 128.8, 128.6, 127.9, 127.6, 127.1, 44.1.

The spectroscopic data matched those reported in the literature. [1]

***N*-(phenylmethyl)-2-(trifluoromethoxy)-benzamide (3t)**

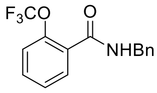

Following the general procedure A, using 2-(trifluoromethoxy)phenyltrifluoroboric acid potassium salt (**1t**) (67.0 mg, 0.25 mmol). Purification by preparative TLC eluting with *n*-hexane/EA afforded **3t** as a white solid (31.0 mg, 40%). The ^{19}F NMR yield of the volatile 2-(trifluoromethoxy)benzoyl fluoride (**2t**) was determined by comparing the integration of the ^{19}F NMR resonance of 2-(trifluoromethoxy)benzoyl fluoride (31.19 ppm) with that of tribromofluoromethane (4.04 ppm). (42% ^{19}F NMR Yield).

R_f = 0.40 [*n*-hexane : EA = 3 : 1(v/v)]. NMR Spectroscopy: ^{1}H NMR (400 MHz, $CDCl_3$) δ 8.00 (dd, *J* = 7.8, 1.9 Hz, 1H), 7.51 (td, *J* = 7.8, 1.9 Hz, 1H), 7.44-7.27 (m, 7H), 6.86 (s, 1H), 4.67 (d, *J* = 5.6 Hz, 2H); ^{13}C NMR (101 MHz, $CDCl_3$) δ 164.3, 145.9, 145.9, 137.6, 132.3, 131.7, 128.8, 127.8, 127.7, 127.4, 121.1, 121.1, 121.0 (q, J_{C-F} = 261.0 Hz), 44.3; ^{19}F NMR (376 MHz, $CDCl_3$) δ -57.25.

The spectroscopic data matched those reported in the literature. [3]

N-(phenylmethyl)-2-(trifluoromethoxy)-benzamide (3u)

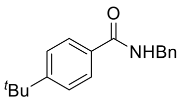

Following the general procedure A, using 2-(trifluoromethoxy)phenyltrifluoroboric acid potassium salt (**1u**) (60.0 mg, 0.25 mmol). Purification by preparative TLC eluting with *n*-hexane/EA afforded **3u** as a white solid (60.6 mg, 90%). The ^{19}F NMR yield of the volatile 4-(*t*-butyl)benzoyl fluoride (**2u**) was determined by comparing the integration of the ^{19}F NMR resonance of 4-(*t*-butyl)benzoyl fluoride (16.98 ppm) with that of tribromofluoromethane (4.04 ppm). (99% ^{19}F NMR Yield).

R_f = 0.30 [*n*-hexane : EA = 3 : 1(v/v)]. NMR Spectroscopy: ^{1}H NMR (400 MHz, $CDCl_3$) δ 7.80 (d, *J* = 8.1 Hz, 2H), 7.44 (d, *J* = 8.1 Hz, 2H), 7.39-7.26 (m, 5H), 7.02-6.93 (m, 1H), 4.62 (d, *J* = 5.7 Hz, 2H), 1.36 (s, 9H); ^{13}C NMR (101 MHz, $CDCl_3$) δ 167.5, 154.9, 138.6, 131.5, 128.0, 127.8, 127.4, 127.0, 125.5, 43.9, 34.9, 31.2.; HRMS-ESI (m/z): Calcd for $C_{18}H_{21}NNaO^+$ [M + Na]$^+$, 290.1515. Found, 290.1515.

3,4,5-Trifluoro-N-(phenylmethyl)-benzamide (3v)

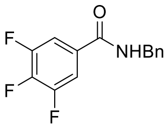

Following the general procedure A, using 3,4,5-trifluorophenyltrifluoroboric acid potassium salt (**1v**) (59.5 mg, 0.25 mmol). Purification by preparative TLC eluting with *n*-hexane/EA afforded **3v** as a white solid (26.6 mg, 40%). The ^{19}F NMR yield of the volatile 3,4,5-trifluorobenzoyl fluoride (**2v**) was determined by comparing the integration of the ^{19}F NMR resonance of 3,4,5-trifluorobenzoyl fluoride (17.84 ppm) with that of tribromofluoromethane (4.04 ppm). (50% ^{19}F NMR Yield).

R_f = 0.30 [*n*-hexane : EA = 3 : 1(v/v)]. NMR Spectroscopy: ^{1}H NMR (400 MHz, $CDCl_3$) δ 7.54-7.41 (m, 2H), 7.39-7.25 (m, 5H), 6.93-6.57 (m, 1H), 4.73-4.48 (m, 2H); ^{13}C NMR (101 MHz, $CDCl_3$) δ 164.3, 152.4, 152.4, 152.3, 152.3, 149.9, 149.9, 149.8, 149.8, 143.4, 143.2,

143.1, 140.8, 140.7, 140.5, 137.4, 130.2, 130.2, 128.9, 127.9, 127.8, 111.9, 111.9, 111.8, 111.7, 44.4; ^{19}F NMR (376 MHz, $CDCl_3$) δ -134.25 (dd, *J* = 20.1, 7.9 Hz), -156.90 (t, *J* = 20.2 Hz).; HRMS-ESI (m/z): Calcd for $C_{14}H_{10}F_3NNaO^+$ $[M + Na]^+$, 288.0607. Found, 288.0607.

N-benzyl-3-phenylpropanamide (3w)

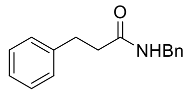

Following the general procedure A, using trifluoro(phenethyl)-λ^4-borane, potassium salt (**1w**) (53.0 mg, 0.25 mmol). Purification by preparative TLC eluting with *n*-hexane/EA afforded **3w** as a white solid (30.0 mg, 50%). The ^{19}F NMR yield of the volatile 3-phenylpropanoyl fluoride (**2w**) was determined by comparing the integration of the ^{19}F NMR resonance of 3-phenylpropanoyl fluoride (42.91 ppm) with that of Phenylsulfonyl fluoride (65.28 ppm). (59% ^{19}F NMR Yield).

R_f = 0.40 [*n*-hexane : EA = 3 : 2 (v/v)]. NMR Spectroscopy: ^{1}H NMR (400 MHz, $CDCl_3$) δ 7.37 – 7.30 (m, 2H), 7.30 – 7.23 (m, 5H), 7.21 – 7.12 (m, 3H), 5.70 (s, 1H), 4.43 (d, *J* = 5.7 Hz, 2H), 2.66 (s, 2H), 2.20 (d, *J* = 7.9 Hz, 2H), 2.00 (s, 2H).; ^{13}C NMR (101 MHz, $CDCl_3$) δ 172.5, 141.4, 138.4, 128.7, 128.5, 128.4, 127.9, 127.6, 126.0, 43.6, 35.9, 35.2, 27.1.

The spectroscopic data matched those reported in the literature. [10]

N-benzyl-4-phenylbutanamide (3x)

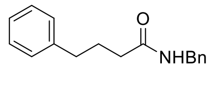

Following the general procedure A, using trifluoro(3-phenylpropyl) -λ^4-borane, potassium salt (**1x**) (56.5 mg, 0.25 mmol). Purification by preparative TLC eluting with *n*-hexane/EA afforded **3x** as a white solid (24.8 mg, 39%). The ^{19}F NMR yield of the volatile 4-phenylbutanoyl fluoride (**2x**) was determined by comparing the integration of the ^{19}F NMR resonance of 4-phenylbutanoyl fluoride (43.44 ppm) with that of Phenylsulfonyl fluoride (65.28 ppm). (50% ^{19}F NMR Yield).

R_f = 0.40 [*n*-hexane : EA = 3 : 2 (v/v)]. NMR Spectroscopy: ^{1}H NMR (400 MHz, $CDCl_3$) δ

7.36 – 7.29 (m, 2H), 7.31 – 7.23 (m, 5H), 7.22 – 7.12 (m, 2H), 5.70 (s, 1H), 4.43 (d, J = 5.7 Hz, 2H), 2.66 (t, J = 7.5 Hz, 2H), 2.20 (d, J = 7.9 Hz, 2H), 2.07 – 1.94 (m, 2H).; ^{13}C NMR (101 MHz, $CDCl_3$) δ 172.5, 141.4, 138.4, 128.7, 128.5, 128.4, 127.9, 127.6, 126.0, 43.6, 35.9, 35.2, 27.1.

The spectroscopic data matched those reported in the literature. [11]

_N_-benzyl-5-phenylpentanamide (3y)

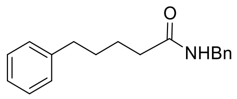

Following the general procedure A, using trifluoro(4-phenylbutyl)-λ^4-borane, potassium salt (**1y**) (24.0 mg, 0.10 mmol). Purification by preparative TLC eluting with *n*-hexane/EA afforded **3y** as a white solid (12.0 mg, 45%). The ^{19}F NMR yield of the volatile 5-phenylpentanoyl fluoride (**2y**) was determined by comparing the integration of the ^{19}F NMR resonance of 5-phenylpentanoyl fluoride (43.25 ppm) with that of Phenylsulfonyl fluoride (65.28 ppm). (64% ^{19}F NMR Yield).

R_f = 0.40 [*n*-hexane : EA = 3 : 2 (v/v)]. NMR Spectroscopy: ^{1}H NMR (400 MHz, $CDCl_3$) δ 7.39 – 7.32 (m, 2H), 7.32 – 7.26 (m, 5H), 7.23 – 7.14 (m, 3H), 5.72 (s, 1H), 4.45 (d, J = 5.7 Hz, 2H), 2.66 (t, J = 7.2 Hz, 2H), 2.25 (t, J = 7.1 Hz, 2H), 1.81 – 1.63 (m, 4H).; ^{13}C NMR (101 MHz, $CDCl_3$) δ 172.5, 141.4, 138.4, 128.7, 128.5, 128.4, 127.9, 127.6, 126.0, 43.6, 35.9, 35.2, 27.1.

The spectroscopic data matched those reported in the literature. [12]

_N_-benzyl-4-(4-fluorophenoxy)butanamide (3z)

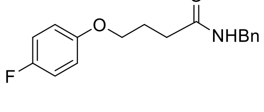

Following the general procedure A, using trifluoro(3-(4-fluorophenoxy)propyl)-λ^4-borane, potassium salt (**1z**) (65.0 mg, 0.25 mmol). Purification by preparative TLC eluting with *n*-hexane/EA afforded **3z** as a white solid (45.1 mg, 63%). The ^{19}F NMR yield of the volatile 4-

(4-fluorophenoxy)butanoyl fluoride (**2z**) was determined by comparing the integration of the ^{19}F NMR resonance of 4-(4-fluorophenoxy)butanoyl fluoride (42.92 ppm) with that of Phenylsulfonyl fluoride (65.28 ppm). (76% ^{19}F NMR Yield).

R_f = 0.30 [*n*-hexane : EA = 3 : 2(v/v)]. NMR Spectroscopy: ^{1}H NMR (400 MHz, $CDCl_3$) δ 7.33 – 7.11 (m, 5H), 7.02 – 6.87 (m, 2H), 6.82 – 6.67 (m, 2H), 5.92 (s, 1H), 4.42 (d, *J* = 5.7 Hz, 2H), 3.95 (t, *J* = 5.9 Hz, 2H), 2.41 (t, *J* = 7.2 Hz, 2H), 2.13 (m, 2H); ^{13}C NMR (101 MHz, $CDCl_3$) δ 172.1, 158.4, 156.1, 154.9, 154.9, 138.2, 128.7, 127.8, 127.6, 115.9, 115.7, 115.4, 115.3, 67.4, 43.7, 32.9, 25.2; ^{19}F NMR (376 MHz, $CDCl_3$): δ = -123.95 – -124.02 (m, 1F). HRMS-ESI (*m/z*): Calcd for $C_{17}H_{18}FNNaO_2^+$ [M + Na]$^+$, 310.1214. Found, 310.1220.

N-benzyl-4-phenoxybutanamide (3aa)

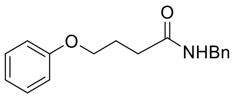

Following the general procedure A, using trifluoro(3-phenoxypropyl)-λ^4-borane, potassium salt (**1aa**) (24.2 mg, 0.10 mmol). Purification by preparative TLC eluting with *n*-hexane/EA afforded **3aa** as a white solid (16.5 mg, 61%). The ^{19}F NMR yield of the volatile 4- 4-phenoxybutanoyl fluoride (**2aa**) was determined by comparing the integration of the ^{19}F NMR resonance of 4-phenoxybutanoyl fluoride (42.72 ppm) with that of Phenylsulfonyl fluoride (65.28 ppm). (63% ^{19}F NMR Yield).

R_f = 0.30 [*n*-hexane : EA = 3 : 2(v/v)]. NMR Spectroscopy: ^{1}H NMR (400 MHz, $CDCl_3$) δ 7.46 – 7.15 (m, 7H), 6.94 (t, *J* = 7.3 Hz, 1H), 6.85 (d, *J* = 8.2 Hz, 2H), 5.89 (s, 1H), 4.43 (d, *J* = 5.7 Hz, 2H), 4.00 (t, *J* = 5.9 Hz, 2H), 2.43 (t, *J* = 7.2 Hz, 2H), 2.29 – 2.01 (m, 2H).; ^{13}C NMR (101 MHz, $CDCl_3$) δ 172.2, 158.8, 138.2, 129.5, 128.7, 127.8, 127.5, 120.8, 114.5, 66.7, 43.7, 33.0, 25.3.; HRMS-ESI (*m/z*): Calcd for $C_{17}H_{20}NO_2^+$ [M + H]$^+$, 270.1489. Found, 270.1489.

N-benzyl-4-(4-chlorophenoxy)butanamide (3ab)

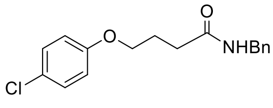

Following the general procedure A, using (3-(4-chlorophenoxy)propyl)trifluoro-λ^4-borane,

potassium salt (**1ab**) (69.1 mg, 0.25 mmol). Purification by preparative TLC eluting with *n*-hexane/EA afforded **3ab** as a white solid (33.0 mg, 43%). The ^{19}F NMR yield of the volatile 4-(4-chlorophenoxy)butanoyl fluoride (**2ab**) was determined by comparing the integration of the ^{19}F NMR resonance of 4-(4-chlorophenoxy)butanoyl fluoride (42.93 ppm) with that of Phenylsulfonyl fluoride (65.28 ppm). (52% ^{19}F NMR Yield).

R_f = 0.30 [*n*-hexane : EA = 3 : 2(v/v)]. NMR Spectroscopy: ^{1}H NMR (400 MHz, $CDCl_3$) δ 7.35 – 7.20 (m, 7H), 6.83 – 6.76 (m, 2H), 5.97 (s, 1H), 4.45 (d, *J* = 5.7 Hz, 2H), 3.98 (t, *J* = 6.0 Hz, 2H), 2.43 (t, *J* = 7.2 Hz, 2H), 2.20 – 2.10 (m, 2H).; ^{13}C NMR (101 MHz, $CDCl_3$) δ 172.1, 157.4, 138.2, 129.4, 128.8, 127.8, 127.6, 125.6, 115.7, 67.2, 43.7, 32.8, 25.1. HRMS-ESI (*m/z*): Calcd for $C_{17}H_{18}ClNNaO_2^+$ [M + Na]$^+$, 326.0918. Found, 326.0922.

_N_-benzyl-4-(4-bromophenoxy)butanamide (3ac)

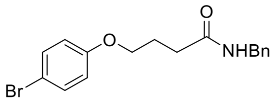

Following the general procedure A, using (3-(4-bromophenoxy)propyl)trifluoro-λ^4-borane, potassium salt (**1ac**) (80.2 mg, 0.25 mmol). Purification by preparative TLC eluting with *n*-hexane/EA afforded **3ac** as a white solid (47.5 mg, 55%). The ^{19}F NMR yield of the volatile 4-(4-bromophenoxy)butanoyl fluoride (**2ac**) was determined by comparing the integration of the ^{19}F NMR resonance of 4-(4-bromophenoxy)butanoyl fluoride (42.94 ppm) with that of Phenylsulfonyl fluoride (65.28 ppm). (93% ^{19}F NMR Yield).

R_f = 0.30 [*n*-hexane : EA = 3 : 2(v/v)]. NMR Spectroscopy: ^{1}H NMR (400 MHz, $CDCl_3$) δ 7.50 – 7.09 (m, 7H), 6.81 – 6.59 (m, 2H), 5.98 (s, 1H), 4.44 (d, *J* = 5.7 Hz, 2H), 3.98 (t, *J* = 6.0 Hz, 2H), 2.43 (t, *J* = 7.2 Hz, 2H), 2.22 – 2.11 (m, 2H).; ^{13}C NMR (101 MHz, $CDCl_3$) δ 172.0, 157.9, 138.2, 132.3, 128.7, 127.8, 127.6, 116.3, 112.9, 67.1, 43.7, 32.8, 25.1. HRMS-ESI (*m/z*): Calcd for $C_{17}H_{19}BrNO_2^+$ [M + H]$^+$, 348.0594. Found, 348.0596.

_N_-benzyl-4-(4-cyanophenoxy)butanamide (3ad)

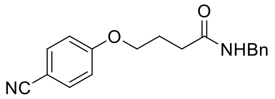

Following the general procedure A, using 4-(3-(trifluoro-λ^4-boraneyl)propoxy)benzonitrile,

potassium salt (**1ad**) (66.8 mg, 0.25 mmol). Purification by preparative TLC eluting with *n*-hexane/EA afforded **3ad** as a white solid (31.0 mg, 42%). The ^{19}F NMR yield of the volatile 4-(4-cyanophenoxy)butanoyl fluoride (**2ad**) was determined by comparing the integration of the ^{19}F NMR resonance of 4-(4-cyanophenoxy)butanoyl fluoride (42.94 ppm) with that of Phenylsulfonyl fluoride (65.28 ppm). (58% ^{19}F NMR Yield).

R_f = 0.30 [*n*-hexane : EA = 3 : 2(v/v)]. NMR Spectroscopy: ^{1}H NMR (400 MHz, $CDCl_3$) δ 7.48 (d, *J* = 8.9 Hz, 2H), 7.29 – 7.14 (m, 5H), 6.83 (d, *J* = 8.8 Hz, 2H), 5.78 (s, 1H), 4.37 (d, *J* = 5.7 Hz, 2H), 3.99 (t, *J* = 6.0 Hz, 2H), 2.35 (t, *J* = 7.1 Hz, 2H), 2.16 – 2.05 (m, 2H).; ^{13}C NMR (101 MHz, $CDCl_3$) δ 171.7, 162.1, 138.1, 134.0, 128.8, 127.8, 127.6, 119.2, 115.2, 104.0, 67.3, 43.7, 32.5, 24.8. HRMS-ESI (*m/z*): Calcd for $C_{18}H_{18}N_2NaO_2^+$ [M + Na]$^+$, 317.1260. Found, 317.1263.

***N*-benzyl-4-(4-(benzyloxy)phenoxy)butanamide (3ae)**

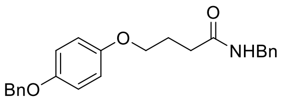

Following the general procedure A, using (3-(4-(benzyloxy)phenoxy)propyl)trifluoro-λ^4-borane, potassium salt (**1ae**) (66.8 mg, 0.25 mmol). Purification by preparative TLC eluting with *n*-hexane/EA afforded **3ae** as a white solid (31.0 mg, 42%). The ^{19}F NMR yield of the volatile 4-(4-(benzyloxy)phenoxy)butanoyl fluoride (**2ae**) was determined by comparing the integration of the ^{19}F NMR resonance of 4-(4-(benzyloxy)phenoxy)butanoyl fluoride (43.01 ppm) with that of Phenylsulfonyl fluoride (65.28 ppm). (45% ^{19}F NMR Yield).

R_f = 0.30 [*n*-hexane : EA = 3 : 2(v/v)]. NMR Spectroscopy: ^{1}H NMR (400 MHz, $CDCl_3$) δ 7.46 – 7.34 (m, 4H), 7.36 – 7.25 (m, 4H), 7.28 – 7.21 (m, 2H), 6.91 – 6.86 (m, 2H), 6.80 – 6.75 (m, 2H), 5.83 (s, 1H), 5.01 (s, 2H), 4.44 (d, *J* = 5.6 Hz, 2H), 3.96 (t, *J* = 5.9 Hz, 2H), 2.43 (t, *J* = 7.2 Hz, 2H), 2.17 – 2.09 (m, 2H).; ^{13}C NMR (101 MHz, $CDCl_3$) δ 172.2, 153.1, 153.1, 138.2, 137.3, 128.7, 128.6, 127.9, 127.8, 127.5, 127.5, 115.9, 115.4, 70.7, 67.4, 43.7, 33.1, 25.3. HRMS-ESI (*m/z*): Calcd for $C_{24}H_{25}NNaO_3^+$ [M + Na]$^+$, 398.1727. Found, 398.1730.

_N_-benzyl-4-(5-methoxy-2-methyl-3-(2-methoxy-2-oxoethyl)-1_H_-indole-1-carbonyl)benzamide (3af)

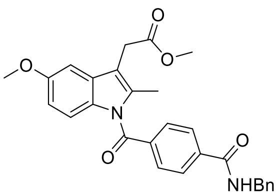

Following the general procedure A, using 4-(5-methoxy-2-methyl-3-(2-methoxy-2-oxoethyl)-1*H*-indole-1-carbonyl)phenyltrifluoroboric acid potassium salt (**1af**) (106.8 mg, 0.25 mmol). Purification by preparative TLC eluting with *n*-hexane/EA afforded **3af** as a white solid (31.8 mg, 28%). The ^{19}F NMR yield of the volatile methyl 2-(1-(4-(fluorocarbonyl)benzoyl)-5-methoxy-2-methyl-1*H*-indol-3-yl)acetate (**2af**) was determined by comparing the integration of the ^{19}F NMR resonance of methyl 2-(1-(4-(fluorocarbonyl)benzoyl)-5-methoxy-2-methyl-1*H*-indol-3-yl)acetate (17.90 ppm) with that of tribromofluoromethane (4.04 ppm). (30% ^{19}F NMR Yield).

R_f = 0.30 [*n*-hexane : EA = 2 : 1(v/v)]. NMR Spectroscopy: ^{1}H NMR (400 MHz, $CDCl_3$) δ 7.82 (dd, *J* = 62.2, 8.4 Hz, 4H), 7.38 (d, *J* = 4.4 Hz, 5H), 6.95 (d, *J* = 2.5 Hz, 1H), 6.83 (d, *J* = 9.0 Hz, 1H), 6.67-6.51 (m, 2H), 4.67 (d, *J* = 5.7 Hz, 2H), 3.83 (s, 3H), 3.70 (s, 3H), 3.66 (s, 2H), 2.36 (s, 3H), 1.64 (s, 1H); ^{13}C NMR (101 MHz, $CDCl_3$) δ 171.3, 168.5, 166.3, 156.2, 138.4, 138.0, 137.8, 136.0, 130.8, 130.7, 129.8, 128.9, 128.0, 127.9, 127.4, 115.1, 112.8, 111.6, 101.4, 55.7, 52.2, 44.4, 30.1, 13.5; HRMS-ESI (m/z): Calcd for $C_{28}H_{26}N_2O_5$ [M]$^+$, 470.1842. Found, 470.1829.

_N_-benzyl-4-(4-((1-isopropoxy-2-methyl-1-oxopropan-2-yl)oxy)benzoyl)benzamide (3ag)

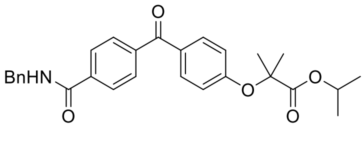

Following the general procedure A, using 4-(4-((1-isopropoxy-2-methyl-1-oxopropan-2-yl)oxy)benzoyl)phenyltrifluoroboric acid potassium salt (**1ag**) (108.1 mg, 0.25 mmol).

Purification by preparative TLC eluting with *n*-hexane/EA afforded **3ag** as a white solid (49.6 mg, 42%). The ^{19}F NMR yield of the volatile isopropyl 2-(4-(4-(fluorocarbonyl)benzoyl)phenoxy)-2-methylpropanoate (**2ag**) was determined by comparing the integration of the ^{19}F NMR resonance of isopropyl 2-(4-(4-(fluorocarbonyl)benzoyl)phenoxy)-2-methylpropanoate (17.87 ppm) with that of tribromofluoromethane (4.04 ppm). (45% ^{19}F NMR Yield).

R_f = 0.50 [*n*-hexane : EA = 2 : 1(v/v)]. NMR Spectroscopy: ^{1}H NMR (400 MHz, $CDCl_3$) δ 7.90 (d, J = 8.3 Hz, 2H), 7.79-7.68 (m, 4H), 7.40 -7.23 (m, 5H), 6.96-6.90 (m, 1H), 6.87 (d, J = 8.8 Hz, 2H), 5.16-5.04 (m, 1H), 4.66 (d, J = 5.6 Hz, 2H), 1.68 (s, 6H), 1.22 (d, J = 6.3 Hz, 6H); ^{13}C NMR (101 MHz, $CDCl_3$) δ 193.7, 172.0, 165.6, 158.9, 139.7, 137.0, 136.2, 131.1, 128.9, 128.7, 127.8, 126.9, 126.6, 125.9, 116.2, 78.4, 68.4, 43.2, 24.3, 20.5; HRMS-ESI (m/z): Calcd for $C_{28}H_{29}NNaO_5^+$ $[M + Na]^+$, 482.1938. Found, 482.1937.

Gibberellic acid derivative (3ah)

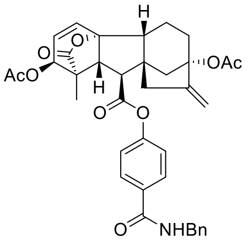

Following the general procedure C, using Gibberellic acid derivative (**1ah**) (35.2 mg, 0.0685 mmol). Purification by preparative TLC eluting with *n*-hexane/EA afforded **3ah** as a white solid (12.5 mg, 35%). The ^{19}F NMR yield of the volatile Triclosan derivative (**2ah**) was determined by comparing the integration of the ^{19}F NMR resonance of Triclosan derivative (**2ah**) (16.35 ppm) with that of tribromofluoromethane (4.04 ppm). (41% ^{19}F NMR Yield).

R_f = 0.30 [*n*-hexane : EA = 1 : 1(v/v)]. NMR Spectroscopy: ^{1}H NMR (400 MHz, $CDCl_3$) δ 7.89 – 7.82 (m, 2H), 7.38 – 7.28 (m, 5H), 7.21 – 7.15 (m, 2H), 6.45 – 6.31 (m, 2H), 5.98 – 5.84 (m, 1H), 5.37 (d, J = 3.8 Hz, 1H), 5.23 – 5.16 (m, 1H), 5.09 – 5.00 (m, 1H), 4.70 – 4.60 (m, 2H), 3.43 – 3.35 (m, 1H), 3.06 – 2.99 (m, 1H), 2.60 (d, J = 10.8 Hz, 1H), 2.54 – 2.47 (m, 1H), 2.43 – 2.36 (m, 2H), 2.34 – 2.23 (m, 2H), 2.12 – 2.09 (m, 2H), 2.04 (s, 3H), 2.02 – 1.99 (m, 1H), 1.78 – 1.68 (m, 3H), 1.26 (s, 3H).; ^{13}C NMR (101 MHz, $CDCl_3$) δ 177.0, 170.2, 170.1, 166.4, 153.2, 152.8, 138.1, 134.2, 132.6, 129.4, 129.0, 128.8, 128.0, 127.9, 121.9, 108.8, 90.0, 84.0,

77.5, 77.2, 76.8, 70.2, 67.2, 53.6, 52.3, 51.6, 51.3, 50.4, 44.4, 42.9, 39.6, 36.7, 31.7, 22.2, 21.0, 16.9, 14.7. HRMS-ESI (*m/z*): Calcd for $C_{37}H_{37}NNaO_9^+$ [M + Na]$^+$, 662.2361. Found, 662.2362.

Triclosan derivative (3ai)

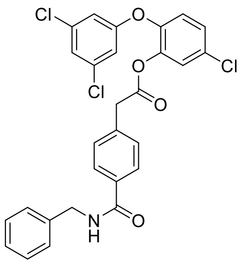

Following the general procedure C, using Triclosan derivative (**1ai**) (35.2 mg, 0.0685 mmol). Purification by preparative TLC eluting with *n*-hexane/EA afforded **3ai** as a white solid (12.5 mg, 35%). The ^{19}F NMR yield of the volatile Triclosan derivative (**2ai**) was determined by comparing the integration of the ^{19}F NMR resonance of Triclosan derivative (**2ai**) (16.35 ppm) with that of tribromofluoromethane (4.04 ppm). (63% ^{19}F NMR Yield).

R$_f$ = 0.40 [*n*-hexane : EA = 3 : 2(v/v)]. NMR Spectroscopy: ^{1}H NMR (400 MHz, $CDCl_3$) δ 8.02 (d, *J* = 8.3 Hz, 2H), 7.39 (d, *J* = 8.1 Hz, 2H), 7.35 – 7.27 (m, 2H), 7.24 – 7.19 (m, 3H), 7.16 – 7.12 (m, 1H), 6.94 – 6.87 (m, 2H), 5.66 (s, 1H), 4.43 (d, *J* = 5.8 Hz, 2H), 3.68 (s, 2H).; ^{13}C NMR (101 MHz, $CDCl_3$) δ 169.7, 163.8, 151.2, 146.9, 141.9, 141.2, 140.6, 138.0, 131.0, 130.4, 129.8, 129.6, 129.5, 128.9, 128.2, 127.8, 124.8, 120.6, 120.5, 44.0, 43.9.

HRMS-ESI (*m/z*): Calcd for $C_{28}H_{20}Cl_3NNaO_4^+$ [M + Na]$^+$, 562.0350. Found, 562.0357.

Deoxycholic acid derivative (2aj)

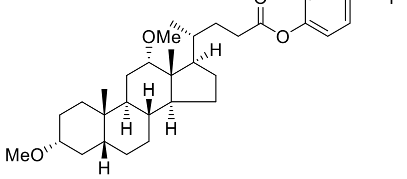

Following the general procedure D, using Deoxycholic acid derivative (**1aj**) (60.2 mg, 0.10 mmol). The reaction mixture was purified by preparative HPLC (10 mL/min, detector UV λ_{max}

210 nm, MeCN/H_2O = 55/45 (0 min), MeCN/H_2O = 75:25 (15 min), MeCN/H_2O = 100:0 (40 min)) to afford Deoxycholic acid derivative (**2aj)** as white solid (17.8 mg, 35%).

NMR Spectroscopy: ^{1}H NMR (400 MHz,$CDCl_3$) δ 8.20 – 7.89 (m, 2H), 7.42 – 7.10 (m, 2H), 3.42 – 3.38 (m, 1H), 3.35 (s, 3H), 3.26 (s, 3H), 3.20 – 3.10 (m, 1H), 2.73 – 2.59 (m, 1H), 2.56 – 2.48 (m, 1H), 1.99 – 1.68 (m, 9H), 1.53 – 1.46 (m, 3H), 1.44 – 1.33 (m, 4H), 1.19 – 1.02 (m, 5H), 1.02 – 0.94 (m, 3H), 0.92 (s, 3H), 0.90 – 0.81 (m, 3H), 0.69 (s, 3H).; ^{13}C NMR (101 MHz, $CDCl_3$) δ 171.9, 157.8, 156.4, 133.2, 133.1, 122.5, 82.2, 80.5, 55.6, 55.6, 48.9, 46.4, 46.3, 36.3, 35.3, 35.0, 34.5, 33.5, 32.6, 31.3, 30.8, 29.7, 27.5, 27.3, 26.8, 26.1, 23. 7, 23.3, 22.0, 17.4, 12.7.; ^{19}F NMR (376 MHz, $CDCl_3$) δ 18.12. HRMS-ESI (*m/z*): Calcd for $C_{33}H_{47}FNaO_5^+$ [M + Na]$^+$, 565.3300. Found, 565.3303.

Estrone derivative (2ak)

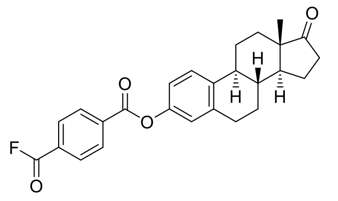

Following the general procedure D, using Deoxycholic acid derivative (**1ak**) (48.0 mg, 0.10 mmol). The reaction mixture was purified by preparative HPLC (10 mL/min, detector UV λ_{max} 210 nm, MeCN/H_2O = 50/50 (0 min), MeCN/H_2O = 70:30 (20 min), MeCN/H_2O = 100:0 (40 min)) to afford Deoxycholic acid derivative (**2ak)** as white solid (23.1 mg, 55%).

NMR Spectroscopy: ^{1}H NMR (400 MHz, $CDCl_3$) δ 8.43 – 8.13 (m, 4H), 7.36 (d, *J* = 8.5 Hz, 1H), 7.05 – 6.91 (m, 2H), 3.01 – 2.88 (m, 2H), 2.58 – 2.48 (m, 1H), 2.48 – 2.40 (m, 1H), 2.38 – 2.29 (m, 1H), 2.23 – 1.95 (m, 4H), 1.72 – 1.42 (m, 6H), 0.93 (s, 3H).; ^{13}C NMR (101 MHz, $CDCl_3$) δ 220.8, 164.1, 148.5, 138.3, 138.0, 135.6, 131.5, 131.5, 130.6, 130.3, 130.2, 126.6, 121.4, 118.6, 50.4, 47.9, 44.2, 38.0, 35.9, 31.6, 29.5, 26.3, 25.8, 21.6, 13.8.; ^{19}F NMR (376 MHz, $CDCl_3$) δ 20.34. HRMS-ESI (*m/z*): Calcd for $C_{26}H_{25}FNaO_4^+$ [M + Na]$^+$, 443.1629. Found, 443.1632.

Potassium trifluoro(phenethyl)-borane (1w)

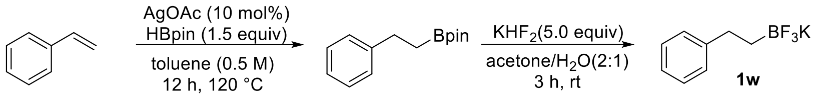

In a 25 mL Shrek tube, the pinacolatoborane (HBpin) (7.5 mmol, 959.9 mg, 1.5 equiv) was added dropwise to the dry toluene (10 mL) solution of styrene (5.0 mmol, 520.8 mg) and silver acetate (0.5 mmol, 83.5 mg, 10 mol%) under the N_2. Then the mixture was stirred at 120 °C for 12 h. The mixture was cooled to room temperature and concentrated in *vacuo*. The residue was dissolved with CH_2Cl_2 (2.0 mL), passed through a pad of silica gel, washed with *n*-hexane/EtOAc 20:1 (v/v), the filtrate was concentrated in vacuo and the crude product 4,4,5,5-tetramethyl-2-phenethyl-1,3,2-dioxaborolane was used for next step directly. The crude product 4,4,5,5-tetramethyl-2-phenethyl-1,3,2-dioxaborolane (2.8 mmol, 638.0 mg, 1.0 equiv) was dissolved in acetone (6.0 ml) and H_2O (3.0 mL), and the KHF_2 (1.073 g, 13.8 mmol, 5.0 equiv) was added to the mixture at room temperature for 3 h. Then the mixture was concentrated in *vacuo*. The residue was dissolved with hot acetone (20 mL), filtered and washed with hot acetone (20 mL) twice. The filtrate was concentrated in *vacuo* and was recrystallized using *n*-hexane and acetone, obtained a white solid potassium trifluoro(phenethyl)-borane **(1w)** (342 mg, 58%).

NMR Spectroscopy: ^{1}H NMR (400 MHz, DMSO-d_6) δ 7.19 (t, J = 7.4 Hz, 2H), 7.15 – 7.08 (m, 2H), 7.05 (t, J = 7.1 Hz, 1H), 2.45 – 2.36 (m, 2H), 0.32 – 0.22 (m, 2H).; ^{13}C NMR (101 MHz, DMSO-d_6) δ 148.6, 128.3, 128.1, 124.8, 32.6, 32.6.; ^{19}F NMR (376 MHz, DMSO-d_6) δ -137.8. The spectroscopic data matched those reported in the literature. [13]

Potassium trifluoro(3-phenylpropyl)-borane (1x)

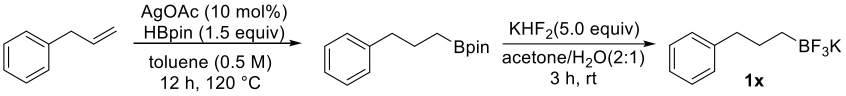

In a 25 mL Shrek tube, the pinacolatoborane (HBpin) (7.5 mmol, 959.9 mg, 1.5 equiv) was added dropwise to the dry toluene (10 mL) solution of allylbenzene (5.0 mmol, 590.9 mg) and silver acetate (0.5 mmol, 83.5 mg, 10 mol%) under the N_2. Then the mixture was stirred at 120 °C for 12 h. The mixture was cooled to room temperature and concentrated in *vacuo*. The residue

was dissolved with CH_2Cl_2 (2.0 mL), passed through a pad of silica gel, washed with *n*-hexane/EtOAc 20:1 (v/v), the filtrate was concentrated in vacuo and the crude product 4,4,5,5-tetramethyl-2-(3-phenylpropyl)-1,3,2-dioxaborolane was used for next step directly. The crude product 4,4,5,5-tetramethyl-2-(3-phenylpropyl)-1,3,2-dioxaborolane (3.86 mmol, 0.950 g, 1.0 equiv) was dissolved in acetone (8.0 ml) and H_2O (4.0 mL), and the KHF_2 (1.507 g, 19.3 mmol, 5.0 equiv) was added to the mixture at room temperature for 3 h. Then the mixture was concentrated in *vacuo*. The residue was dissolved with hot acetone (20 mL), filtered and washed with hot acetone (20 mL) twice. The filtrate was concentrated in *vacuo* and was recrystallized using *n*-hexane and acetone, obtained a white solid potassium trifluoro(3-phenylpropyl)-borane (**1x**) (658 mg, 75%).

NMR Spectroscopy: ^{1}H NMR (400 MHz, DMSO-d_6) δ 7.26 – 7.19 (m, 2H), 7.15 – 7.07 (m, 3H), 2.49 – 2.44 (m, 2H), 1.48 – 1.37 (m, 2H), 0.06 – -0.07 (m, 2H).; ^{13}C NMR (101 MHz, DMSO-d_6) δ 144.6, 128.8, 128.7, 128.4, 125.5, 28.6.; ^{19}F NMR (376 MHz, DMSO-d_6) δ -136.8. The spectroscopic data matched those reported in the literature. [14]

Potassium trifluoro(4-phenylbutyl)-borane (1y)

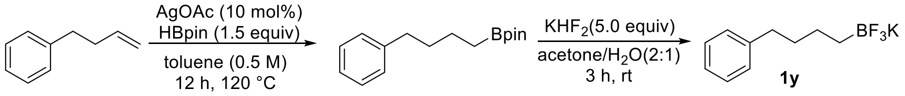

In a 25 mL Shrek tube, the pinacolatoborane (HBpin) (7.5 mmol, 959.9 mg, 1.5 equiv) was added dropwise to the dry toluene (10 mL) solution of 4-phenyl-1-butene (5.0 mmol, 661.0 mg) and silver acetate (0.5 mmol, 83.5 mg, 10 mol%) under the N_2. Then the mixture was stirred at 120 °C for 12 h. The mixture was cooled to room temperature and concentrated in *vacuo*. The residue was dissolved with CH_2Cl_2 (2.0 mL), passed through a pad of silica gel, washed with *n*-hexane/EtOAc 20:1 (v/v), the filtrate was concentrated in vacuo and the crude product 4,4,5,5-tetramethyl-2-(4-phenylbutyl)-1,3,2-dioxaborolane was used for next step directly. The crude product 4,4,5,5-tetramethyl-2-(4-phenylbutyl)-1,3,2-dioxaborolane (4.15 mmol, 1.086 g, 1.0 equiv) was dissolved in acetone (8.0 ml) and H_2O (4.0 mL), and the KHF_2 (1.620 g, 20.7 mmol, 5.0 equiv) was added to the mixture at room temperature for 3 h. Then the mixture was concentrated in *vacuo*. The residue was dissolved with hot acetone (20 mL), filtered and washed

with hot acetone (20 mL) twice. The filtrate was concentrated in *vacuo* and was recrystallized using *n*-hexane and acetone, obtained a white solid potassium trifluoro(4-phenylbutyl)-borane (**1y**) (615 mg, 62%).

NMR Spectroscopy: ^{1}H NMR (400 MHz, Acetone-d_6) δ 7.04 – 6.96 (m, 2H), 6.96 – 6.91 (m, 2H), 6.91 – 6.86 (m, 1H), 2.36 – 2.29 (m, 2H), 1.41 – 1.28 (m, 2H), 1.17 – 1.05 (m, 2H), 0.06 – -0.07 (m, 2H).; ^{13}C NMR (101 MHz, Acetone-d_6) δ 143.8, 128.3, 128.0, 125.1, 36.2, 35.7, 25.4, 25.4.; ^{19}F NMR (376 MHz, DMSO-d_6) δ -136.8.

The spectroscopic data matched those reported in the literature. [13]

Potassium trifluoro(3-(4-fluorophenoxy)propyl)-borane (1z)

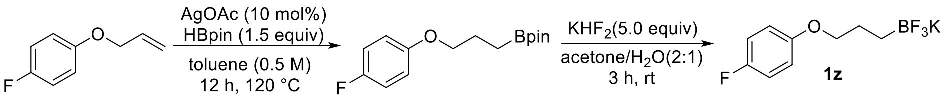

In a 25 mL Shrek tube, the pinacolatoborane (HBpin) (7.5 mmol, 959.9 mg, 1.5 equiv) was added dropwise to the dry toluene (10 mL) solution of allylbenzene (5.0 mmol, 760.8 mg) and silver acetate (0.5 mmol, 83.5 mg, 10 mol%) under the N_2. Then the mixture was stirred at 120 °C for 12 h. The mixture was cooled to room temperature and concentrated in *vacuo*. The residue was dissolved with CH_2Cl_2 (2.0 mL), passed through a pad of silica gel, washed with *n*-hexane/EtOAc 20:1 (v/v), the filtrate was concentrated in vacuo and the crude product 2-(3-(4-fluorophenoxy)propyl)-4,4,5,5-tetramethyl-1,3,2-dioxaborolane was used for next step directly. The crude product 2-(3-(4-fluorophenoxy)propyl)-4,4,5,5-tetramethyl-1,3,2-dioxaborolane (1.17 mmol, 0.3119 g, 1.0 equiv) was dissolved in acetone (6.0 ml) and H_2O (3.0 mL), and the KHF_2 (0.458 g, 5.86 mmol, 5.0 equiv) was added to the mixture at room temperature for 3 h. Then the mixture was concentrated in *vacuo*. The residue was dissolved with hot acetone (20 mL), filtered and washed with hot acetone (20 mL) twice. The filtrate was concentrated in *vacuo* and was recrystallized using *n*-hexane and acetone, obtained a white solid potassium trifluoro(3-(4-fluorophenoxy)propyl)-borane, (**1z**) (217 mg, 71%).

NMR Spectroscopy: ^{1}H NMR (400 MHz, Acetone-d_6) δ 6.84 – 6.74 (m, 2H), 6.71 – 6.63 (m, 2H), 3.67 (t, J = 7.7 Hz, 2H), 1.56 – 1.44 (m, 2H), 0.13 – -0.06 (m, 2H).; ^{13}C NMR (101 MHz, Acetone) δ 157.8, 156.0, 155.5, 115.5, 115.4, 115.4, 115.2, 71.8, 25.3.; ^{19}F NMR (376 MHz,

Acetone-d_6) δ -119.31 – -125.90 (m), -136.21. Mass Spectrometry: HRMS-ESI (*m/z*): Calcd for $C_9H_{10}BF_4O^-$ [M -K]$^-$, 221.0766. Found, 221.0760.

Potassium trifluoro(3-phenoxypropyl)-borane (1aa)

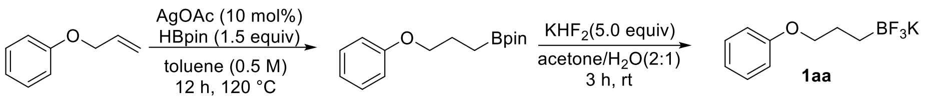

In a 25 mL Shrek tube, the pinacolatoborane (HBpin) (7.5 mmol, 959.9 mg, 1.5 equiv) was added dropwise to the dry toluene (10 mL) solution of (allyloxy)benzene (5.0 mmol, 670.8 mg) and silver acetate (0.5 mmol, 83.5 mg, 10 mol%) under the N_2. Then the mixture was stirred at 120 °C for 12 h. The mixture was cooled to room temperature and concentrated in *vacuo*. The residue was dissolved with CH_2Cl_2 (2.0 mL), passed through a pad of silica gel, washed with *n*-hexane/EtOAc 20:1 (v/v), the filtrate was concentrated in vacuo and the crude product 4,4,5,5-tetramethyl-2-(3-phenoxypropyl)-1,3,2-dioxaborolane was used for next step directly. The crude product 4,4,5,5-tetramethyl-2-(3-phenoxypropyl)-1,3,2-dioxaborolane (1.00 mmol, 0.262 g, 1.0 equiv) was dissolved in acetone (6.0 ml) and H_2O (3.0 mL), and the KHF_2 (0.390 g, 5.00 mmol, 5.0 equiv) was added to the mixture at room temperature for 3 h. Then the mixture was concentrated in *vacuo*. The residue was dissolved with hot acetone (20 mL), filtered and washed with hot acetone (20 mL) twice. The filtrate was concentrated in *vacuo* and was recrystallized using *n*-hexane and acetone, obtained a white solid potassium trifluoro(3-phenoxypropyl)-borane (**1aa**) (134.8 mg, 56%).

NMR Spectroscopy: ^{1}H NMR (400 MHz, DMSO-d_6) δ 7.25 (t, J = 7.5 Hz, 2H), 7.05 – 6.74 (m, 3H), 3.91 – 3.78 (m, 2H), 1.69 – 1.43 (m, 2H), 0.24 – -0.19 (m, 2H).; ^{13}C NMR (101 MHz, DMSO-d_6) δ 159.5, 129.8, 120.2, 114.8, 71.2, 25.8.; ^{19}F NMR (376 MHz, DMSO-d_6) δ -136.21. The spectroscopic data matched those reported in the literature. [13]

Potassium trifluoro(3-(4-chlorophenoxy)propyl)-borane (1ab)

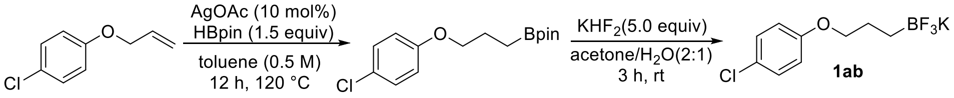

In a 25 mL Shrek tube, the pinacolatoborane (HBpin) (7.5 mmol, 959.9 mg, 1.5 equiv) was

added dropwise to the dry toluene (10 mL) solution of 1-(allyloxy)-4-chlorobenzene (5.0 mmol, 843.1 mg) and silver acetate (0.5 mmol, 83.5 mg, 10 mol%) under the N_2. Then the mixture was stirred at 120 °C for 12 h. The mixture was cooled to room temperature and concentrated in *vacuo*. The residue was dissolved with CH_2Cl_2 (2.0 mL), passed through a pad of silica gel, washed with *n*-hexane/EtOAc 20:1 (v/v), the filtrate was concentrated in vacuo and the crude product 2-(3-(4-chlorophenoxy)propyl)-4,4,5,5-tetramethyl-1,3,2-dioxaborolane was used for next step directly. The crude product 2-(3-(4-chlorophenoxy)propyl)-4,4,5,5-tetramethyl-1,3,2-dioxaborolane (2.71 mmol, 0.805 g, 1.0 equiv) was dissolved in acetone (8.0 ml) and H_2O (4.0 mL), and the KHF_2 (1.060 g, 13.57 mmol, 5.0 equiv) was added to the mixture at room temperature for 3 h. Then the mixture was concentrated in *vacuo*. The residue was dissolved with hot acetone (20 mL), filtered and washed with hot acetone (20 mL) twice. The filtrate was concentrated in *vacuo* and was recrystallized using *n*-hexane and acetone, obtained a white solid potassium trifluoro(3-(4-chlorophenoxy)propyl)-borane (**1ab**) (541 mg, 72%).

NMR Spectroscopy: ^{1}H NMR (400 MHz, Acetone-d_6) δ 7.19 (d, J = 8.4 Hz, 2H), 6.83 (d, J = 8.2 Hz, 2H), 3.82 (m, 2H), 1.66 (m, 2H), 0.32 – 0.02 (m, 2H). ^{13}C NMR (101 MHz, Acetone-d_6) δ 158.5, 129.0, 124.0, 115.9, 71.6, 25.2.; ^{19}F NMR (376 MHz, Acetone-d_6) δ 36.39.

Mass Spectrometry: HRMS-ESI (*m/z*): Calcd for $C_9H_{10}BClF_3O^-$ [M -K]$^-$, 237.0471. Found, 221.0760.

Potassium trifluoro(3-(4-bromophenoxy)propyl)-borane (1ac)

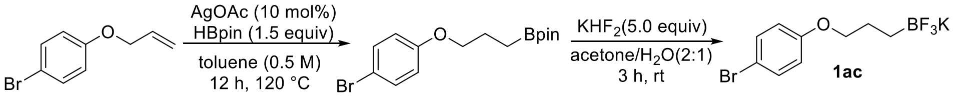

In a 25 mL Shrek tube, the pinacolatoborane (HBpin) (7.5 mmol, 959.9 mg, 1.5 equiv) was added dropwise to the dry toluene (10 mL) solution of 1-(allyloxy)-4-bromobenzene (5.0 mmol, 843.1 mg) and silver acetate (0.5 mmol, 83.5 mg, 10 mol%) under the N_2. Then the mixture was stirred at 120 °C for 12 h. The mixture was cooled to room temperature and concentrated in *vacuo*. The residue was dissolved with CH_2Cl_2 (2.0 mL), passed through a pad of silica gel, washed with *n*-hexane/EtOAc 20:1 (v/v), the filtrate was concentrated in vacuo and the crude product 2-(3-(4-bromophenoxy)propyl)-4,4,5,5-tetramethyl-1,3,2-dioxaborolane was used for

next step directly. The crude product 2-(3-(4-bromophenoxy)propyl)-4,4,5,5-tetramethyl-1,3,2-dioxaborolane (1.59 mmol, 0.543 g, 1.0 equiv) was dissolved in acetone (6.0 ml) and H_2O (3.0 mL), and the KHF_2 (0.621 g, 7.95 mmol, 5.0 equiv) was added to the mixture at room temperature for 3 h. Then the mixture was concentrated in *vacuo*. The residue was dissolved with hot acetone (20 mL), filtered and washed with hot acetone (20 mL) twice. The filtrate was concentrated in *vacuo* and was recrystallized using *n*-hexane and acetone, obtained a white solid potassium trifluoro(3-(4-bromophenoxy)propyl)-borane (**1ac**) (321 mg, 61%).

NMR Spectroscopy: ^{1}H NMR (400 MHz, DMSO-d_6) δ 7.25 (t, J = 7.5 Hz, 2H), 7.05 – 6.74 (m, 3H), 3.91 – 3.78 (m, 2H), 1.69 – 1.43 (m, 2H), 0.24 – -0.19 (m, 2H).; ^{13}C NMR (101 MHz, DMSO-d_6) δ 159.5, 129.8, 120.2, 114.8, 71.2, 25.8.; ^{19}F NMR (376 MHz, DMSO-d_6) δ -136.21. The spectroscopic data matched those reported in the literature. [13]

Potassium trifluoro(3-(4-cyanophenoxy)propyl)borane (1ad)

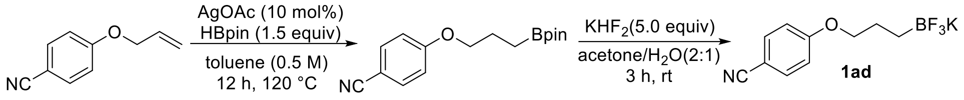

In a 25 mL Shrek tube, the pinacolatoborane (HBpin) (7.5 mmol, 959.9 mg, 1.5 equiv) was added dropwise to the dry toluene (10 mL) solution of 4-(allyloxy)benzonitrile (5.0 mmol, 795.9 mg) and silver acetate (0.5 mmol, 83.5 mg, 10 mol%) under the N_2. Then the mixture was stirred at 120 °C for 12 h. The mixture was cooled to room temperature and concentrated in *vacuo*. The residue was dissolved with CH_2Cl_2 (2.0 mL), passed through a pad of silica gel, washed with *n*-hexane/EtOAc 15:1 (v/v), the filtrate was concentrated in vacuo and the crude product 4-(3-(4,4,5,5-tetramethyl-1,3,2-dioxaborolan-2-yl)propoxy)benzonitrile was used for next step directly. The crude product 4-(3-(4,4,5,5-tetramethyl-1,3,2-dioxaborolan-2-yl)propoxy)benzonitrile (2.14 mmol, 0.615 g, 1.0 equiv) was dissolved in acetone (8.0 ml) and H_2O (4.0 mL), and the KHF_2 (0.836 g, 10.71 mmol, 5.0 equiv) was added to the mixture at room temperature for 3 h. Then the mixture was concentrated in *vacuo*. The residue was dissolved with hot acetone (20 mL), filtered and washed with hot acetone (20 mL) twice. The filtrate was concentrated in *vacuo* and was recrystallized using *n*-hexane and acetone, obtained a white solid potassium trifluoro(3-(4-cyanophenoxy)propyl)borane (**1ad**) (434 mg, 76%).

NMR Spectroscopy: ^{1}H NMR (400 MHz, DMSO-d_6) δ 7.87 – 7.58 (m, 2H), 7.19 – 6.91 (m, 2H), 3.95 (t, J = 7.4 Hz, 2H), 1.69 – 1.47 (m, 2H), 0.19 – -0.04 (m, 2H).; ^{13}C NMR (101 MHz, DMSO-d_6) δ 163.0, 134.6, 119.8, 115.9, 102.5, 71.9, 25.5, 0.6.; ^{19}F NMR (376 MHz, DMSO-d_6) δ -137.03.

The spectroscopic data matched those reported in the literature. [13]

Potassium trifluoro(3-(4-(benzyloxy)phenoxy)propyl)-borane (1ae)

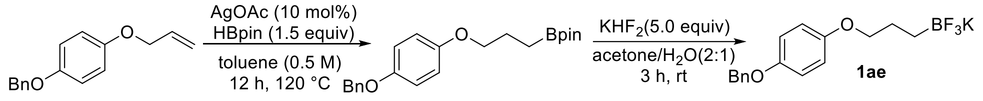

In a 25 mL Shrek tube, the pinacolatoborane (HBpin) (7.5 mmol, 959.9 mg, 1.5 equiv) was added dropwise to the dry toluene (10 mL) solution of 1-(allyloxy)-4-(benzyloxy)benzene (5.0 mmol, 1.201 g) and silver acetate (0.5 mmol, 83.5 mg, 10 mol%) under the N_2. Then the mixture was stirred at 120 °C for 12 h. The mixture was cooled to room temperature and concentrated in *vacuo*. The residue was dissolved with CH_2Cl_2 (2.0 mL), passed through a pad of silica gel, washed with *n*-hexane/EtOAc 20:1 (v/v), the filtrate was concentrated in *vacuo* and the crude product 2-(3-(4-chlorophenoxy)propyl)-4,4,5,5-tetramethyl-1,3,2-dioxaborolane was used for next step directly. The crude product 2-(3-(4-chlorophenoxy)propyl)-4,4,5,5-tetramethyl-1,3,2-dioxaborolane (1.63 mmol, 0.600 g, 1.0 equiv) was dissolved in acetone (6.0 ml) and H_2O (3.0 mL), and the KHF_2 (0.636 g, 8.14 mmol, 5.0 equiv) was added to the mixture at room temperature for 3 h. Then the mixture was concentrated in *vacuo*. The residue was dissolved with hot acetone (20 mL), filtered and washed with hot acetone (20 mL) twice. The filtrate was concentrated in *vacuo* and was recrystallized using *n*-hexane and acetone, obtained a white solid potassium trifluoro(3-(4-(benzyloxy)phenoxy)propyl)-borane (**1ae**) (274 mg, 48%).

NMR Spectroscopy: ^{1}H NMR (400 MHz, DMSO-d_6) δ 7.51 – 7.35 (m, 4H), 7.36 – 7.28 (m, 1H), 6.94 – 6.87 (m, 2H), 6.85 – 6.76 (m, 2H), 5.02 (s, 2H), 3.77 (t, J = 7.3 Hz, 2H), 1.63 – 1.47 (m, 2H), 0.10 – -0.07 (m, 2H).; ^{13}C NMR (101 MHz, DMSO-d_6) δ 153.8, 152.4, 137.9, 128.8, 128.2, 128.1, 116.1, 115.6, 71.8, 70.1, 25.9.; ^{19}F NMR (376 MHz, DMSO-d_6) δ 137.03.

Mass Spectrometry: HRMS-ESI (*m/z*): Calcd for $C_{16}H_{17}BF_3O_2^-$ [M -K]$^-$, 309.1279. Found, 309.1277.

Synthesis of 4-(5-methoxy-2-methyl-3-(2-methoxy-2-oxoethyl)-1*H*-indole-1-carbonyl)phenyltrifluoroboric acid potassium salt (1af)

Step 1

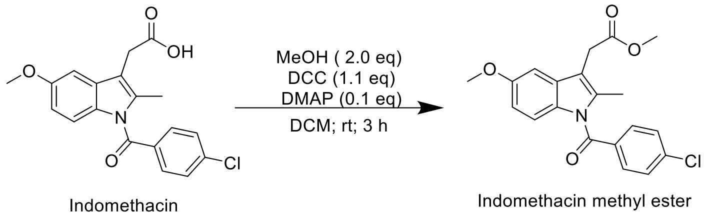

In a 100 mL round-bottom flask, the indomethacin (**4af**) (1.789g, 5.0 mmol, 1.0 equiv) was added to the dry DCM (40 mL) solution of DCC (*N*,*N*'-dicyclohexylcarbodiimide) (1.132g, 1.1 equiv.) and DMAP (4-(dimethylamino) pyridine) (61.1 mg, 0.1 equiv.). Then added dry MeOH (0.4 mL, 2.0 eq) to the solution under the Ar. The mixture was stirred at room temperature for 3 h and used TLC to detect the reaction. Added saturated sodium bicarbonate solution to quench the reaction, extracted twice with DCM, dry the organic phase with anhydrous sodium sulfate, and was dried *in vacuo.* Separated using column chromatography, obtained a light yellow solid Indomethacin methyl ester (1.710 g, 92%).

R_f = 0.30 [*n*-hexane : EA = 5 : 1(v/v)]. ^{1}H NMR (400 MHz, $CDCl_3$) δ 7.69-7.63 (m, 2H), 7.49-7.44 (m, 2H), 6.96 (d, *J* = 2.5 Hz, 1H), 6.86 (d, *J* = 9.0 Hz, 1H), 6.67 (dd, *J* = 9.0, 2.6 Hz, 1H), 3.84 (s, 3H), 3.70 (s, 3H), 3.67 (s, 2H), 2.39 (s, 3H); ^{13}C NMR (101 MHz, $CDCl_3$) δ 171.4, 168.3, 156.0, 139.3, 136.0, 133.9, 131.3, 131.1, 130.8, 130.7, 129.2, 129.1, 115.0, 115.0, 112.5, 111.6, 111.6, 101.3, 55.7, 55.7, 52.2, 52.2, 30.2, 13.4.

The spectroscopic data matched those reported in the literature. [17]

Step 2

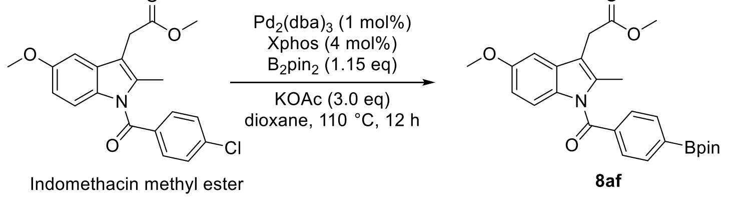

In a 25 mL Schlenk tube, Indomethacin methyl ester (2.0 mmol, 743.8 mg, 1.0 equiv.) tris(dibenzylideneacetone) dipalladium(0)($Pd_2(dba)_3$) (18.31 mg, 1 mol%) 2-dicyclohexylphosphino-2',4',6'-tri-*i*-propyl-1,1'-biphenyl (X-Phos) (38.14 mg, 4 mol%)

bis(pinacolato)diboron (584.1 mg, 1.15 equiv.) and potassium acetate (588.8 mg, 3.0 equiv.) was dissolved in 9.3 mL dioxane. The mixture was bubbled for 10 minutes, stirred at 110°C for 12 h and used TLC to detect the reaction. Added water to quench the reaction, extracted three times with ethyl acetate, dry the organic phase with anhydrous sodium sulfate, and was dried *in vacuo.* Separated using column chromatography, obtained colorless liquid Methyl 2-(5-methoxy-2-methyl-1-(4-(4,4,5,5-tetramethyl-1,3,2-dioxaborolan-2-yl)benzoyl)-1H-indol-3-yl)acetate **(8af)** (530 mg, 57%).

R_f = 0.30 [*n*-hexane : EA = 5 : 1(v/v)]. ^{1}H NMR (400 MHz, $CDCl_3$) δ 7.91 (d, *J* = 8.1 Hz, 2H), 7.68 (d, *J* = 8.1 Hz, 2H), 6.95 (d, *J* = 2.6 Hz, 1H), 6.88 (d, *J* = 9.0 Hz, 1H), 6.64 (dd, *J* = 9.0, 2.5 Hz, 1H), 3.83 (s, 3H), 3.70 (s, 3H), 3.67 (s, 2H), 2.36 (s, 3H), 1.38 (s, 12H); ^{13}C NMR (101 MHz, $CDCl_3$) δ 171.3, 169.4, 156.0, 138.0, 136.0, 135.0, 130.9, 130.6, 128.6, 115.2, 112.4, 111.5, 101.2, 84.3, 55.6, 52.1, 30.1, 24.9, 13.4.

The spectroscopic data matched those reported in the literature. [16]

Step 3

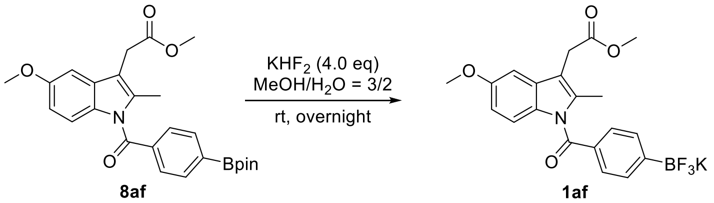

In a 25 mL round-bottom flask, **8af** (3.0 mmol, 1.390g, 1.0 equiv.) was dissolved in 4.5 mL MeOH, and potassium hydrogen fluoride was dissolved in 3.0 mL water, and added to the mixture. The mixture was stirred at room temperature overnight. The solid was filtered off and washed with hot acetone (10 mL) three times, then was dried *in vacuo* to obtain a light yellow solid 4-(5-methoxy-2-methyl-3-(2-methoxy-2-oxoethyl)-1H-indole-1-carbonyl) phenyltrifluoroboric acid potassium salt **(1af)** (970 mg, 75%).

^{1}H NMR (400 MHz, DMSO-d_6) δ 7.51 (d, *J* = 8.1 Hz, 2H), 7.41 (d, *J* = 7.7 Hz, 2H), 7.01 (d, *J* = 2.5 Hz, 1H), 6.83 (d, *J* = 9.0 Hz, 1H), 6.68 (dd, *J* = 9.0, 2.6 Hz, 1H), 3.79 (s, 2H), 3.76 (s, 3H), 3.64 (s, 3H), 2.25 (s, 3H); ^{13}C NMR (101 MHz, DMSO-d_6) δ 171.2, 169.9, 155.2, 135.6, 131.8, 131.6, 130.5, 130.1, 127.7, 114.2, 111.6, 111.1, 101.2, 55.3, 51.8, 29.1, 12.8. ^{19}F NMR (376 MHz, DMSO-d_6) δ -136.05.

The spectroscopic data matched those reported in the literature. [15]

Synthesis of 4-(4-((1-isopropoxy-2-methyl-1-oxopropan-2-yl)oxy)benzoyl) phenyltrifluoroboric acid potassium salt (1ag)

Step 1

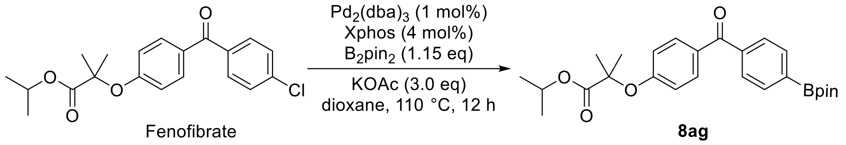

In a 25 mL Schlenk tube, Fenofibrate (2.0 mmol, 721.7 mg, 1.0 equiv.) tris(dibenzylideneacetone) dipalladium(0)($Pd_2(dba)_3$) (18.31 mg, 1 mol%) 2-dicyclohexylphosphino-2',4',6'-tri-*i*-propyl-1,1'-biphenyl (X-Phos) (38.14 mg, 4 mol%) bis(pinacolato)diboron (584.1 mg, 1.15 equiv.) and potassium acetate (588.8 mg, 3.0 equiv.) was dissolved in 9.3 mL dioxane. The mixture was bubbled for 10 minutes, stirred at 110°C for 12 h and used TLC to detect the reaction. Added water to quench the reaction, extracted three times with ethyl acetate, dry the organic phase with anhydrous sodium sulfate, and was dried *in vacuo.* Separated using column chromatography, obtained colorless liquid isopropyl 2-methyl-2-(4-(4-(4,4,5,5-tetramethyl-1,3,2-dioxaborolan-2-yl)benzoyl)phenoxy)propanoate **(8ag)** (685 mg, 76%).

R_f = 0.30 [*n*-hexane : EA = 6 : 1(v/v)]. ^{1}H NMR (400 MHz, $CDCl_3$) δ 7.94-7.88 (m, 2H), 7.80-7.67 (m, 4H), 6.93-6.79 (m, 2H), 5.17-5.02 (m, 1H), 1.68 (s, 6H), 1.39 (s, 12H), 1.22 (d, *J* = 6.3 Hz, 6H); ^{13}C NMR (101 MHz, $CDCl_3$) δ 195.8, 173.2, 159.6, 140.4, 134.7, 134.3, 132.3, 131.9, 130.5, 128.9, 128.6, 117.1, 117.1, 84.2, 79.4, 69.5, 69.2, 25.5, 25.2, 25.0, 24.8, 21.6, 21.4.

The spectroscopic data matched those reported in the literature. [18]

Step 2

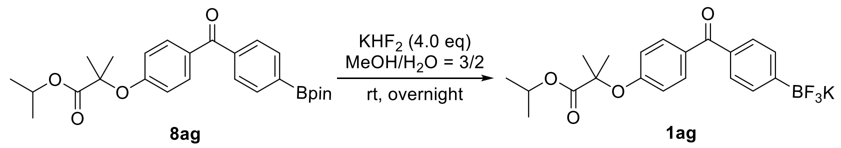

In a 25 mL round-bottom flask, **8x** (3.0 mmol, 1.357g, 1.0 equiv.) was dissolved in 4.5 mL

MeOH, and potassium hydrogen fluoride was dissolved in 3.0 mL mater, and added to the mixture. The mixture was stirred at room temperature overnight. The solid was filtered off and washed with hot acetone (10 mL) three times, then was dried in vacuo to obtain a light yellow solid 4-(4-((1-isopropoxy-2-methyl-1-oxopropan-2-yl)oxy)benzoyl) phenyltrifluoroboric acid potassium salt **(1ag)** (1.030 g, 79%) .

^{1}H NMR (400 MHz, DMSO-d_6) δ 7.79 – 7.56 (m, 2H), 7.64 – 7.27 (m, 4H), 6.93 – 6.63 (m, 2H), 4.99 (p, J = 6.2 Hz, 1H), 1.61 (s, 6H), 1.16 (d, J = 6.3 Hz, 6H); ^{13}C NMR (101 MHz, DMSO-d_6) δ 195.5, 172.7, 159.0, 134.9, 132.1, 132.0, 131.6, 131.3, 128.2, 117.4, 117.4, 79.4, 69.4, 69.3, 25.6, 25.5, 21.7, 21.7. ^{19}F NMR (376 MHz, DMSO-d_6) δ -137.51. Mass Spectrometry: HRMS-ESI (*m/z*): Calcd for $C_{20}H_{21}BF_3O_4^-$ [M -K]$^-$, 393.1490. Found, 393.1490.

Gibberellic acid derivative (1ah)

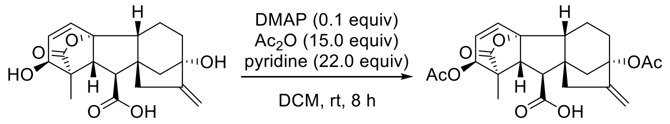

In a 100 mL round-bottom flask, the acetic anhydride (75.0 mmol, 7.675 g , 15.0 equiv) was added to the dry DCM (50 mL) solution of Gibberellic acid (5.0 mmol, 1.732 g), 4-(dimethylamino)pyridine (DMAP) (0.5 mmol, 61.8 mg, 0.1 equiv) and pyridine (110.0 mmol, 8.700 g, 22.0 equiv) under the N_2. The reaction was stirred for 8 hours at room temperature, and was quenched with water (30 mL), extracted three times with DCM (30 mL), the organic phase was washed twice with saturated brine (50 mL), and the organic phase was dried over anhydrous magnesium sulfate and concentrated in *vacuo*. The crude product 3,13-di-*O*-acetylgibberellic acid was used for next step directly.

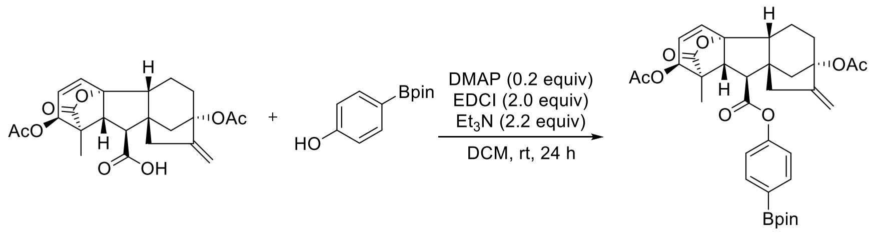

In a 25 mL round-bottom flask, the triethylamine (11.0 mmol, 1.113 g , 2.2 equiv) was added to the dry DCM (10 mL) solution of the crude product 3,13-di-*O*-acetylgibberellic acid, 4-

(dimethylamino)pyridine (DMAP) (1.0 mmol, 112.2 mg, 0.2 equiv), 4-(4,4,5,5-tetramethyl-1,3,2-dioxaborolan-2-yl)phenol (5.0 mmol, 1.100 g, 1.0 equiv) and *N*-(3-dimethylaminopropyl) -*N′*-ethylcarbodiimide hydrochloride (EDCI)(10.0 mmol, 1.917 g, 2.0 equiv) under the N_2. The reaction was stirred for 24 hours at room temperature, and was quenched with water (10 mL), extracted three times with DCM (10 mL), the organic phase was washed with saturated brine (30 mL), and the organic phase was dried over anhydrous magnesium sulfate and concentrated in *vacuo*. The residue was passed through a pad of silica gel, washed with *n*-hexane/EtOAc 3:1 (v/v), the filtrate was concentrated in *vacuo* and the crude product 3,13-di-*O*-acetylgibberellic acid, 4-(4,4,5,5-Tetramethyl-1,3,2-dioxaborolan-2-yl)phenyl ester was used for next step directly.

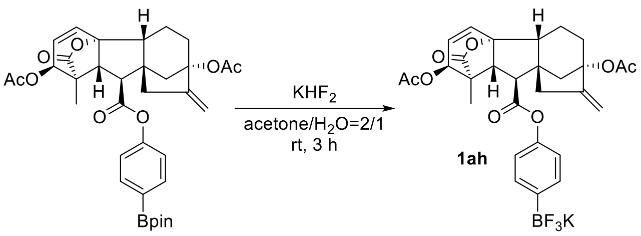

The crude product 3,13-di-*O*-acetylgibberellic acid, 4-(4,4,5,5-Tetramethyl-1,3,2-dioxaborolan-2-yl)phenyl ester (0.51 mmol, 0.323 g, 1.0 equiv) was dissolved in acetone (6.0 ml) and H_2O (3.0 mL), and the KHF_2 (0.201 g, 2.6 mmol, 5.0 equiv) was added to the mixture at room temperature for 3 h. Then the mixture was concentrated in *vacuo*. The residue was dissolved with hot acetone (20 mL), filtered and washed with hot acetone (20 mL) twice. The filtrate was concentrated in *vacuo* and was recrystallized using *n*-hexane and acetone, obtained a white solid Gibberellic acid derivative (**1ah**) (201 mg, 66%).

NMR Spectroscopy: ^{1}H NMR (400 MHz, DMSO-d_6) δ 7.35 (s, 2H), 6.81 (d, *J* = 7.7 Hz, 2H), 6.59 (d, *J* = 9.3 Hz, 1H), 5.84 (dd, *J* = 9.4, 3.7 Hz, 1H), 5.23 (d, *J* = 3.7 Hz, 1H), 5.14 (s, 1H), 5.02 (s, 1H), 3.26 (d, *J* = 11.1 Hz, 1H), 2.98 (d, *J* = 11.1 Hz, 1H), 2.43 – 2.31 (m, 3H), 2.27 – 2.21 (m, 1H), 2.15 – 2.10 (m, 1H), 2.07 (s, 3H), 1.98 (s, 3H), 1.81 – 1.69 (m, 3H), 1.13 (s, 3H).; ^{13}C NMR (101 MHz, DMSO-d_6) δ 177.1, 170.7, 170.3, 169.9, 153.9, 148.5, 135.4, 132.7, 132.6, 128.8, 119.8, 119.6, 113.8, 108.4, 90.5, 84.0, 69.9, 53.2, 52.1, 51.1, 51.1, 50.3, 50.2, 42.1, 36.5, 22.2, 21.0, 16.7, 14.6.; ^{19}F NMR (376 MHz, DMSO-d_6) δ -138.96. Mass Spectrometry: HRMS-ESI (*m/z*): Calcd for $C_{29}H_{29}BF_3O_8^-$ [M -K]$^-$, 573.1913. Found, 573.1918.

Potassium trifluoro(4-(2-(5-chloro-2-(3,5-dichlorophenoxy)phenoxy)-2-oxoethyl)phenyl)borane (1ai)

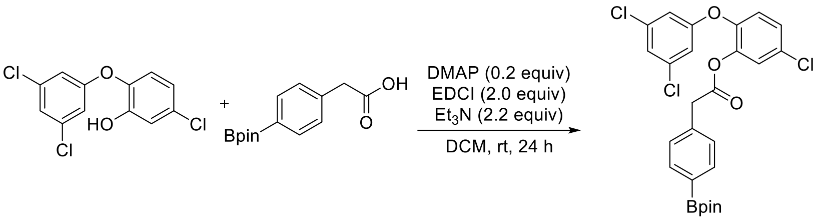

In a 25 mL round-bottom flask, the triethylamine (4.4 mmol, 0.445 g , 2.2 equiv) was added to the dry DCM (10 mL) solution of the triclosan (2.0 mmol, 0.579 g, 1.0 equiv), 4-(dimethylamino)pyridine (DMAP) (0.4 mmol, 48.0 mg, 0.2 equiv), 2-(4-(4,4,5,5-tetramethyl-1,3,2-dioxaborolan-2-yl)phenyl)acetic acid (2.0 mmol, 0.524 g, 1.0 equiv) and *N*-(3-dimethylaminopropyl) -*N'*-ethylcarbodiimide hydrochloride (EDCI)(4.0 mmol, 0.765 g, 2.0 equiv) under the N_2. The reaction was stirred for 24 hours at room temperature, and was quenched with water (10 mL), extracted three times with DCM (10 mL), the organic phase was washed with saturated brine (30 mL), and the organic phase was dried over anhydrous magnesium sulfate and concentrated in *vacuo*. The residue was passed through a pad of silica gel, washed with *n*-hexane/EtOAc 5:1 (v/v), the filtrate was concentrated in *vacuo* and the crude product 2-(4-(4,4,5,5-tetramethyl-1,3,2-dioxaborolan-2-yl)phenyl)acetic acid, 5-chloro-2-(2,4 dichlorophenoxy)phenyl ester was used for next step directly.

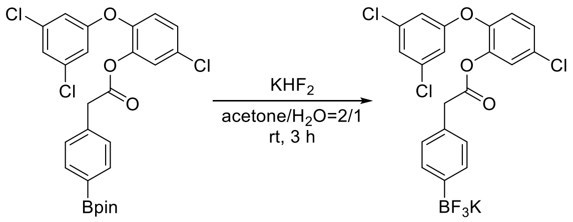

The crude product 2-(4-(4,4,5,5-tetramethyl-1,3,2-dioxaborolan-2-yl)phenyl)acetic acid, 5-chloro-2-(2,4 dichlorophenoxy)phenyl ester (0.87 mmol, 0.462 g, 1.0 equiv) was dissolved in acetone (6.0 ml) and H_2O (3.0 mL), and the KHF_2 (0.339 g, 4.34 mmol, 5.0 equiv) was added to the mixture at room temperature for 3 h. Then the mixture was concentrated in *vacuo*. The residue was dissolved with hot acetone (20 mL), filtered and washed with hot acetone (20 mL) twice. The filtrate was concentrated in *vacuo* and was recrystallized using *n*-hexane and acetone, obtained a white solid potassium trifluoro(4-(2-(5-chloro-2-(3,5-dichlorophenoxy)phenoxy)-2-

oxoethyl)phenyl)borane (**1ai**) (381 mg, 85%).

NMR Spectroscopy: ^{1}H NMR (400 MHz, DMSO-d_6) δ 7.80 (d, J = 2.5 Hz, 1H), 7.49 (d, J = 2.6 Hz, 1H), 7.41 – 7.35 (m, 2H), 7.24 (d, J = 7.4 Hz, 2H), 7.13 (d, J = 8.8 Hz, 1H), 6.98 – 6.90 (m, 3H), 3.72 (s, 2H). ^{13}C NMR (101 MHz, DMSO) δ 170.0, 151.3, 146.7, 142.2, 131.9, 130.6, 129.3, 128.9, 128.8, 127.8, 127.7, 125.0, 121.8, 120.7; ^{19}F NMR (376 MHz, DMSO) δ -138.99.

Mass Spectrometry: HRMS-ESI (m/z): Calcd for $C_{20}H_{12}BCl_3F_3O_3^-$ [M -K]$^-$, 472.9903. Found, 472.9903.

Deoxycholic acid derivative (1aj)

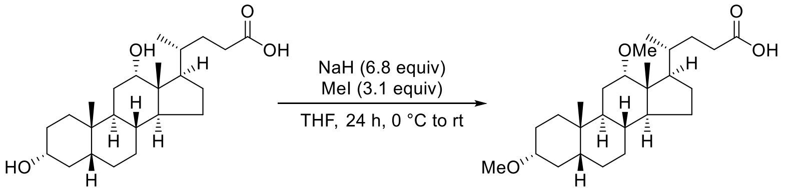

In a 100 mL round-bottom flask, sodium hydride (27.2 mmol, 1.088 g , 6.8 equiv) was added to the dry THF (48 mL) solution of Deoxycholic acid (4.0 mmol, 1.57 g), under the N_2 at 0 °C. The reaction was stirred for 30 minutes at 0 °C, and then MeI (12.5 mmol, 0.78 mL, 3.12 equiv) was added dropwise to the reaction at 0 °C . The reaction was stirred for 24 hours at room temperature, and was quenched with saturated aqueous ammonium chloride (30 mL), extracted three times with ethyl acetate (30 mL), the organic phase was washed with saturated brine (100 mL), and the organic phase was dried over anhydrous magnesium sulfate and concentrated in *vacuo*. The crude product (*3α,5β,12α*)-3,12-dimethoxycholan-24-oic acid was used for next step directly.

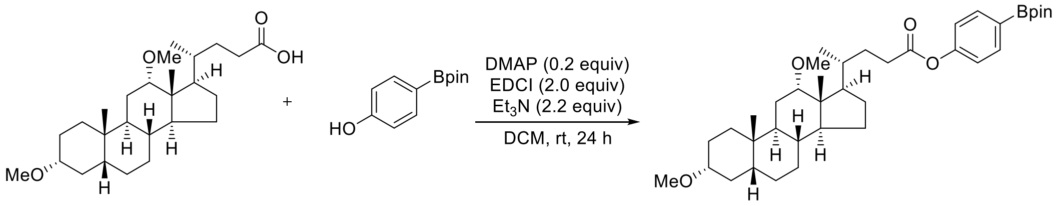

In a 25 mL round-bottom flask, the triethylamine (8.8 mmol, 0.890 g , 2.2 equiv) was added to the dry DCM (10 mL) solution of the crude product (*3α,5β,12α*)-3,12-dimethoxycholan-24-oic acid, 4-(dimethylamino)pyridine (DMAP) (0.8 mmol, 97.7 mg, 0.2 equiv), 4-(4,4,5,5-tetramethyl-1,3,2-dioxaborolan-2-yl)phenol (4.0 mmol, 0.880 g, 1.0 equiv) and *N*-(3-dimethylaminopropyl) -*N′*-ethylcarbodiimide hydrochloride (EDCI) (8.0 mmol, 1.533 g, 2.0

equiv) under the N_2. The reaction was stirred for 24 hours at room temperature, and was quenched with water (10 mL), extracted three times with DCM (10 mL), the organic phase was washed with saturated brine (30 mL), and the organic phase was dried over anhydrous magnesium sulfate and concentrated in *vacuo*. The residue was passed through a pad of silica gel, washed with *n*-hexane/EtOAc 5:1 (v/v), the filtrate was concentrated in *vacuo* and the crude product (*3α,5β,12α*)-3,12-dimethoxycholan-24-oic acid, 4-(4,4,5,5-Tetramethyl-1,3,2-dioxaborolan-2-yl)phenyl ester was used for next step directly.

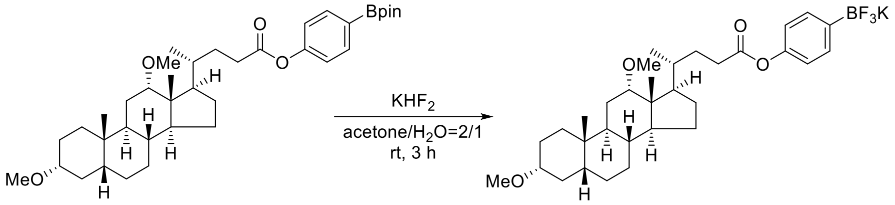

The crude product (*3α,5β,12α*)-3,12-dimethoxycholan-24-oic acid, 4-(4,4,5,5-Tetramethyl-1,3,2-dioxaborolan-2-yl)phenyl ester (1.0 mmol, 0.631 g, 1.0 equiv) was dissolved in acetone (6.0 ml) and H_2O (3.0 mL), and the KHF_2 (0.390 g, 5.0 mmol, 5.0 equiv) was added to the mixture at room temperature for 3 h. Then the mixture was concentrated in *vacuo*. The residue was dissolved with hot acetone (20 mL), filtered and washed with hot acetone (20 mL) twice. The filtrate was concentrated in *vacuo* and was recrystallized using *n*-hexane and acetone, obtained a white solid Deoxycholic acid derivative (**1aj**) (420 mg, 70%).

NMR Spectroscopy: ^1H NMR (400 MHz, DMSO-d_6) δ 7.32 (d, *J* = 7.8 Hz, 2H), 6.78 (d, *J* = 7.8 Hz, 2H), 3.40 (s, 1H), 3.15 – 3.03 (m, 1H), 2.61 – 2.46 (m, 2H), 2.47 – 2.36 (m, 1H), 1.91 – 1.69 (m, 7H), 1.68 – 1.57 (m, 1H), 1.57 – 1.47 (m, 4H), 1.33 (s, 5H), 1.28 – 1.11 (m, 5H), 1.07 (s, 3H), 1.10 – 0.98 (m, 3H), 0.93 (m, 4H), 0.89 (s, 3H), 0.66 (s, 3H). ^{13}C NMR (101 MHz, DMSO-d_6) δ 172.8, 149.0, 132.5, 119.8, 81.9, 79.9, 74.0, 55.6, 55.3, 49.1, 46.6, 46.5, 41.7, 35.9, 35.2, 35.0, 34.4, 33.6, 32.8, 31.3, 31.1, 27.4, 27.2, 26.9, 26.3, 25.4, 23.8, 23.5, 22.0, 17.7, 12.9.; ^{19}F NMR (376 MHz, DMSO-d_6) δ -138.94. Mass Spectrometry: HRMS-ESI (*m/z*): Calcd for $C_{32}H_{47}BF_3O_4^-$ [M -K]$^-$, 563.3525. Found, 563.3527.

Estrone derivative (1ak)

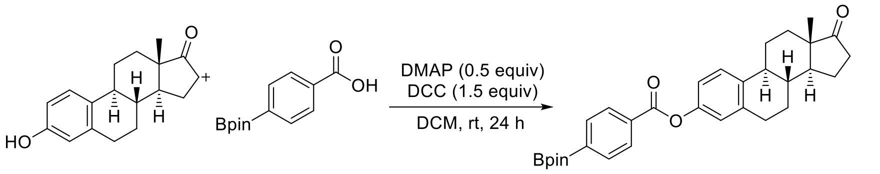

In a 50 mL round-bottom flask, the Estrone (4.1 mmol, 1.017 g , 1.0 equiv) was added to the dry DCM (25 mL) solution of 4-(dimethylamino)pyridine (DMAP) (2.05 mmol, 250.0 mg, 0.5 equiv), 2-(4-(4,4,5,5-tetramethyl-1,3,2-dioxaborolan-2-yl)phenyl)acetic acid (4.1 mmol, 1.011 g, 1.0 equiv) and *N*,*N′*-Dicyclohexylcarbodiimide (DCC) (6.1 mmol, 1.268 g, 1.5 equiv) under the N_2. The reaction was stirred for 24 hours at room temperature, and was quenched with water (20 mL), extracted three times with DCM (20 mL), the organic phase was washed with saturated brine (60 mL), and the organic phase was dried over anhydrous magnesium sulfate and concentrated in *vacuo*. The residue was passed through a pad of silica gel, washed with *n*-hexane/EtOAc 5:1 (v/v), the filtrate was concentrated in *vacuo* and the crude product Estrone, 4-(4,4,5,5-Tetramethyl-1,3,2-dioxaborolan-2-yl)benzoate was used for next step directly.

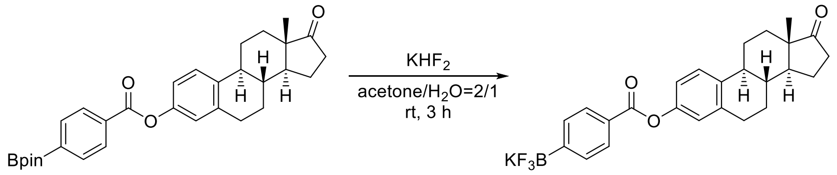

The crude product Estrone, 4-(4,4,5,5-Tetramethyl-1,3,2-dioxaborolan-2-yl)benzoate (1.1 mmol, 0.540 g, 1.0 equiv) was dissolved in acetone (6.0 ml) and H_2O (3.0 mL), and the KHF_2 (0.430 g, 5.5 mmol, 5.0 equiv) was added to the mixture at room temperature for 3 h. Then the mixture was concentrated in *vacuo*. The residue was dissolved with hot acetone (20 mL), filtered and washed with hot acetone (20 mL) twice. The filtrate was concentrated in *vacuo* and was recrystallized using *n*-hexane and acetone, obtained a white solid Estrone derivative (**1ak**) (380 mg, 73%).

NMR Spectroscopy: ^{1}H NMR (400 MHz, DMSO-d_6) δ 7.85 (d, *J* = 7.6 Hz, 2H), 7.51 (d, *J* = 7.7 Hz, 2H), 7.38 – 7.25 (m, 1H), 7.08 – 6.86 (m, 2H), 2.94 – 2.81 (m, 2H), 2.45 – 2.37 (m, 2H), 2.30 – 2.22 (m, 1H), 2.12 – 2.02 (m, 1H), 1.99 – 1.91 (m, 2H), 1.83 – 1.71 (m, 1H), 1.59 – 1.36 (m, 6H), 0.83 (s, 3H).; ^{13}C NMR (101 MHz, DMSO-d_6) δ 220.1, 166.0, 149.3, 138.3,

137.5, 132.1, 128.3, 126.8, 126.2, 125.7, 122.2, 119.6, 50.1, 47.8, 44.1, 38.0, 35.9, 31.8, 31.2, 29.4, 26.3, 25.8, 25.4, 21.6, 14,0.; ^{19}F NMR (376 MHz, DMSO-d_6) δ -139.93. Mass Spectrometry: HRMS-ESI (*m/z*): Calcd for $C_{25}H_{25}BF_3O_3^-$ [M -K]$^-$, 441.1854. Found, 441.1851.

Synthesis of Pd(II)-OCF_3 complex (5a)

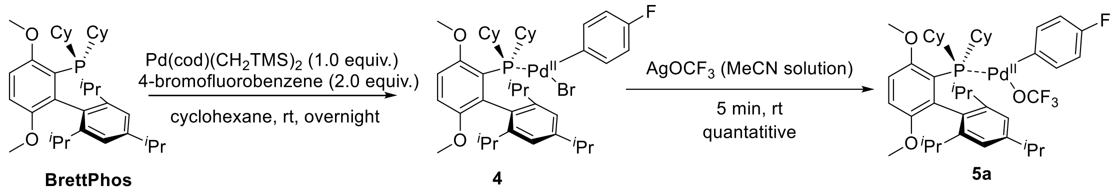

In a N_2 glovebox, to Brettphos (1.074g, 2.0 mmol, 1.0 equiv.) in a 25 mL round-bottom flask, cyclohexane was added until Brettphos dissolved. Then Pd(cod)(CH_2TMS)$_2$ (778.0 mg, 2.0 mmol, 1.0 equiv.) and 4-bromofluorbenzene (700.0 mg, 4.0 mmol, 2.0 equiv.) was added to the mixture. The mixture was stirred at room temperature overnight, then *n*-pentane was added to dilute the system. The system was cooled at -20 °C, filtered and washed three times with *n*-pentane to obtain Pd(II)complex **4** (1.03 g, 63%).

In a N_2 glovebox, to **4** (20.5 mg, 0.025mmol, 1.0 equiv.) in a 2.00 mL sealed vial tube, DCM (0.40 mL) and $AgOCF_3$ (17 μL, 2.0 mol/L in MeCN, 1.36 equiv.) was added. The mixture was stirred at room temperature for 5 minutes, the system was filtered and used *n*-pentane as poor solvent to recrystallization.

Synthesis of dicyclohexyldifluoro(2',4',6'-triisopropyl-3,6-dimethoxy-(1,1'-biphenyl)-2-yl)-λ^5-phosphane (7)

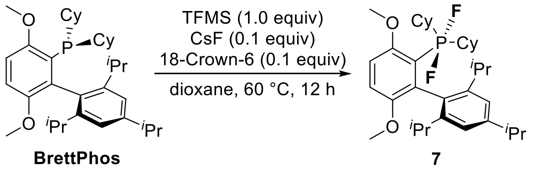

In a N_2 glovebox, to BrettPhos (268.4 mg, 0.5 mmol, 1.00 equiv.) CsF (7.6 mg, 0.05 mmol, 0.10 equiv.) and 18-crown-6 (13.2 mg, 0.05 mmol, 0.10 equiv.) in a 8.00 mL sealed vial tube were added 1,4-dioxane (4.00 mL) and TFMS (80 μL, 0.5 mmol, 1.00 equiv.) Then the sealed vial was taken outside the glovebox and the reaction mixture was stirred for 12 h at 60 °C. The

system was filtered and used *n*-pentane as poor solvent to recrystallization. The ^{19}F NMR yield of the **7** was determined by comparing the integration of the ^{19}F NMR resonance of **7** [δ -41.46 ppm (d, *J* = 681.3 Hz)] with that of phenyl trifluoromethyl sulfide (-41.27 ppm). (48% ^{19}F NMR Yield). The product was easily hydrolyzed, the process of filtration and recrystallization was carried out in a N_2 glovebox.

^{1}H NMR (400 MHz, $CDCl_3$) δ 7.01 (s, 2H), 6.93-6.86 (m, 1H), 6.84-6.74 (m, 1H), 3.86 (s, 3H), 3.55 (s, 3H), 2.93 (p, *J* = 6.9 Hz, 1H), 2.57 (p, *J* = 6.7 Hz, 2H), 2.30-1.87 (m, 2H), 1.85-1.67 (m, 3H), 1.72-1.64 (m, 1H), 1.53-1.41 (m, 7H), 1.35-0.99 (m, 19H), 0.89 (d, *J* = 6.8 Hz, 6H), 0.82-0.60 (m, 2H); ^{13}C NMR (101 MHz, $CDCl_3$) δ 152.0, 151.8, 151.2, 148.1, 147.8, 131.7, 131.7, 120.8, 110.8, 110.7, 109.9, 55.8, 54.8, 34.2, 31.0, 29.2, 29.2, 29.2, 29.1, 29.1, 28.5, 28.3, 28.3, 28.1, 27.1, 26.6, 25.3, 24.2, 22.7; ^{31}P NMR (162 MHz, $CDCl_3$) δ -26.08 (t, *J* = 681.6 Hz); ^{19}F NMR (376 MHz, $CDCl_3$) δ -44.69 (d, *J* = 681.3 Hz).

Synthesis of (5-(diphenylphosphaneyl)-9,9-dimethyl-9H-xanthen-4-yl)difluorodiphenyl-λ^{5}-phosphane (L5)

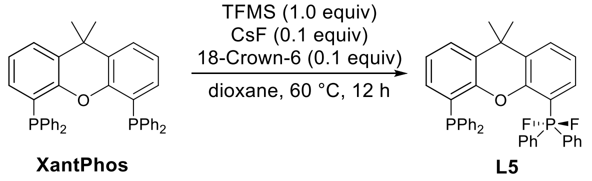

In a N_2 glovebox, to XantPhos (289.3 mg, 0.5 mmol, 1.00 equiv.) CsF (7.6 mg, 0.05 mmol, 0.10 equiv.) and 18-crown-6 (13.2 mg, 0.05 mmol, 0.10 equiv.) in a 8.00 mL sealed vial tube were added 1,4-dioxane (4.00 mL) and TFMS (80 μL, 0.5 mmol, 1.00 equiv.) Then the sealed vial was taken outside the glovebox and the reaction mixture was stirred for 12 h at 60 °C. The system was filtered and used *n*-pentane as poor solvent to recrystallization. The ^{19}F NMR yield of the **L5** was determined by comparing the integration of the ^{19}F NMR resonance of **L5** [δ -27.29 ppm (d, *J* = 655.1 Hz)] with that of phenyl trifluoromethyl sulfide (-41.27 ppm). (99% ^{19}F NMR Yield). The product was easily hydrolyzed, the process of filtration and recrystallization was carried out in a N_2 glovebox.

Supplementary Discussion

Effect of Pd catalyst on the reaction

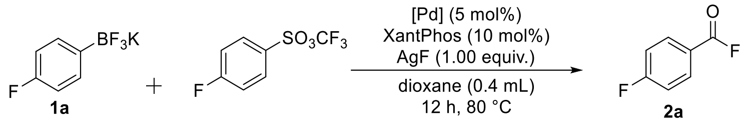

In a N_2 glovebox, to 4-fluorobenzenetrifluoroboric acid potassium salt (**1a**) (10.1 mg, 0.05 mmol, 1.00 equiv.) , Pd catalyst (0.0025 mmol, 0.05 equiv.), AgF (6.3 mg, 0.05 mmol, 1.00 equiv.) and XantPhos (2.8 mg, 0.005 mmol, 0.10 equiv.) in a 2.00 mL sealed vial tube were added 1,4-dioxane (0.40 mL), and TFMS (trifluoromethyl 4-fluorobenzenesulfonate) (28.0 µL, 0.175 mmol, 3.50 equiv.). Then the sealed vial was taken outside the glovebox and the reaction mixture was stirred for 12 h at 80 °C. The yield of 4-fluoro-Benzoyl fluoride (**2a**) was determined by comparing the integration of the ^{19}F NMR resonance of 4-fluoro-Benzoyl fluoride (-100.73 ppm) with that of 1-Fluoro-3-nitrobenzene (-112.0 ppm). Yields are reported in **Supplementary Table 1**.

Supplementary Table 1: Effect of Pd catalyst on the reaction

Catalyst (5mol%)	Yield[%] (^{19}F NMR)	Catalyst (5 mol%)	Yield[%] (^{19}F NMR)
$PdBr_2$	21	$Pd(MeCN)_2Cl_2$	72
$Pd(dba)_2$	6	$[PdCl(allyl)]_2$	2
$Pd(NO_3)_2$	trace	$Pd(PhCN)_2Cl_2$	54
$Pd(cod)Cl_2$	99	$[PdCl(cinnamyl)]_2$	25
$Pd(PPh_3)_4$	3	$Pd(OAc)_2$	4

Effect of solvent on the reaction

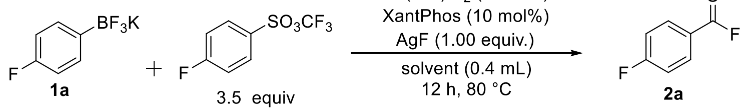

In a N_2 glovebox, to 4-fluorobenzenetrifluoroboric acid potassium salt (**1a**) (10.1 mg, 0.05 mmol, 1.00 equiv.) , $Pd(cod)Cl_2$ (0.7 mg, 0.0025 mmol, 0.05 equiv.), AgF(6.3 mg, 0.05 mmol, 1.00 equiv.) and Xantphos (2.8 mg, 0.005 mmol, 0.10 equiv.) in a 2.00 mL sealed vial tube

were added solvent (0.40 mL), and TFMS (trifluoromethyl 4-fluorobenzenesulfonate) (28.0 µL, 0.175 mmol, 3.50 equiv.). Then the sealed vial was taken outside the glovebox and the reaction mixture was stirred for 12 h at 80 °C. The yield of 4-fluoro-Benzoyl fluoride (**2a**) was determined by comparing the integration of the ^{19}F NMR resonance of 4-fluoro-Benzoyl fluoride (-100.73 ppm) with that of 1-Fluoro-3-nitrobenzene (-112.0 ppm). Yields are reported in **Supplementary Table 2**.

Supplementary Table 2: Effect of solvent on the reaction

Solvent	Yield[%] (^{19}F NMR)
dioxane	99
MeCN	0
DMA	trace
DMF	0
EA	80
THF	39
DMC	60
anisole	34

Effect of AgF amount on the reaction

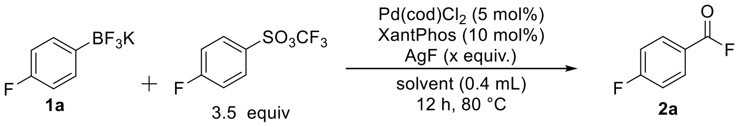

In a N_2 glovebox, to 4-fluorobenzenetrifluoroboric acid potassium salt (**1a**) (10.1 mg, 0.05 mmol, 1.00 equiv.), Pd(cod)Cl_2 (0.7 mg, 0.0025 mmol, 0.05 equiv.), AgF (x equiv.) and Xantphos (2.8 mg, 0.005 mmol, 0.10 equiv.) in a 2.00 mL sealed vial tube were added solvent (0.40 mL), and TFMS (trifluoromethyl 4-fluorobenzenesulfonate) (28.0 µL, 0.175 mmol, 3.50 equiv.). Then the sealed vial was taken outside the glovebox and the reaction mixture was stirred for 12 h at 80 °C. The yield of 4-fluoro-Benzoyl fluoride (**2a**) was determined by comparing the integration of the ^{19}F NMR resonance of 4-fluoro-Benzoyl fluoride (-100.73 ppm) with that of 1-Fluoro-3-nitrobenzene (-112.0 ppm). Yields are reported in **Supplementary Table 3**.

Supplementary Table 3: Effect of AgF amount on the reaction

Amount of AgF	Yield[%] (^{19}F NMR)
0 equiv.	0
0.1 equiv.	Trace
0.3 equiv.	59
0.5 equiv.	90
1.0 equiv.	99
1.5 equiv.	99
2.0 equiv.	77
3.0 equiv.	75

Effect of P ligand on the reaction

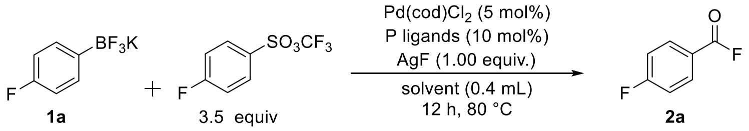

In a N_2 glovebox, to 4-fluorobenzenetrifluoroboric acid potassium salt (**1a**) (10.1 mg, 0.05 mmol, 1.00 equiv.), Pd(cod)Cl_2 (0.7 mg, 0.0025 mmol, 0.05 equiv.), AgF(6.3 mg, 0.05 mmol, 1.00 equiv.) and P ligand (0.10 equiv.) in a 2.00 mL sealed vial tube were added solvent (0.40 mL), and TFMS (trifluoromethyl 4-fluorobenzenesulfonate) (28.0 μL, 0.175 mmol, 3.50 equiv.). Then the sealed vial was taken outside the glovebox and the reaction mixture was stirred for 12 h at 80 °C. The yield of 4-fluoro-Benzoyl fluoride (**2a**) was determined by comparing the integration of the ^{19}F NMR resonance of 4-fluoro-Benzoyl fluoride (-100.73 ppm) with that of 1-Fluoro-3-nitrobenzene (-112.0 ppm). Yields are reported in **Supplementary Table 4**.

Supplementary Table 4: Effect of P ligand on the reaction

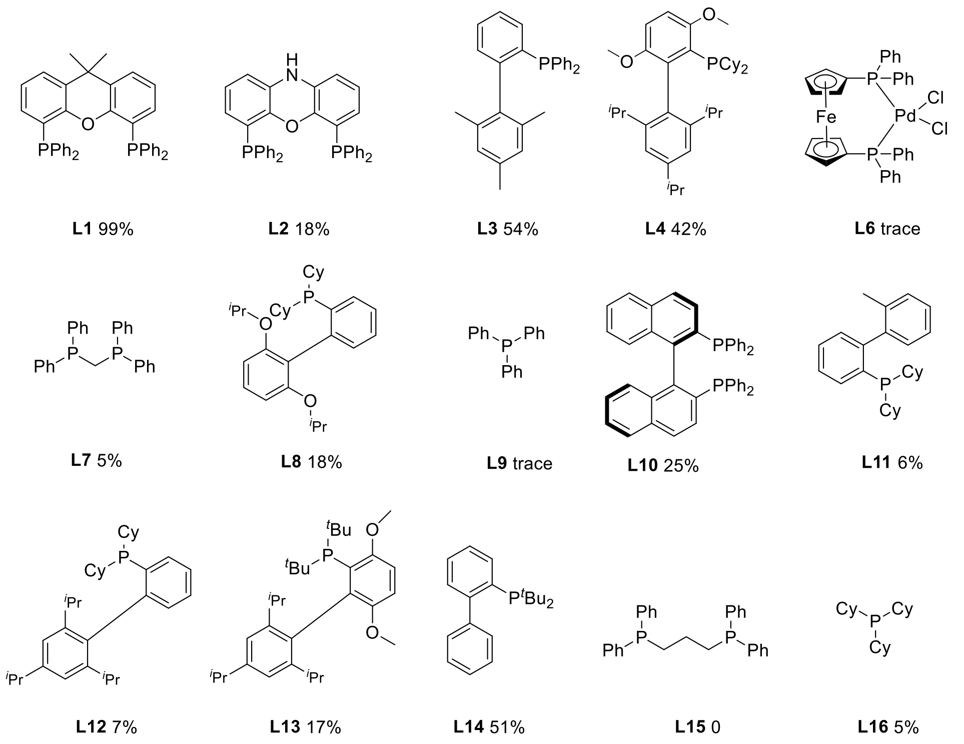

CO Detection Experiment

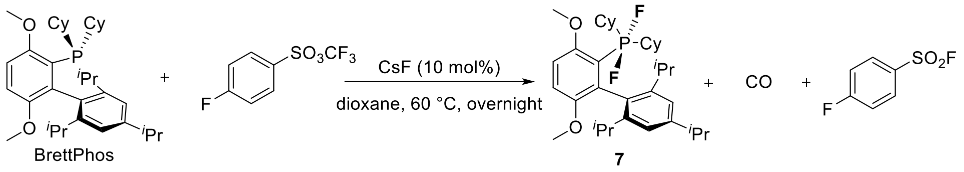

In a N_2 glovebox, to BrettPhos (0.1 mmol, 53.7 mg, 1.0 equiv.) in a 2.00 mL sealed vial tube were added 1,4-dioxane (0.40 mL), and TFMS (trifluoromethyl 4-fluorobenzenesulfonate) (16.0 µL, 0.10 mmol, 1.0 equiv.). Then the sealed vial was taken outside the glovebox and the reaction mixture was stirred for overnight at 60 °C. Then CO was detected by CO detection tube.

Supplementary Scheme 1: CO detection tube color rendering

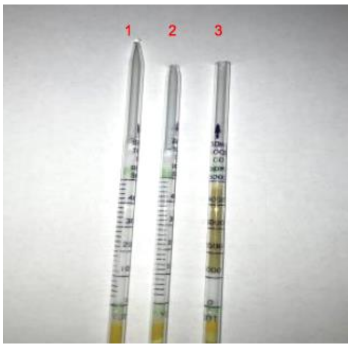

tube 1: original tube; tube 2: pass through COF_2 (diluted by Ar) ; tube 3: pass through the above reaction gas

Isotope Labelling Experiment

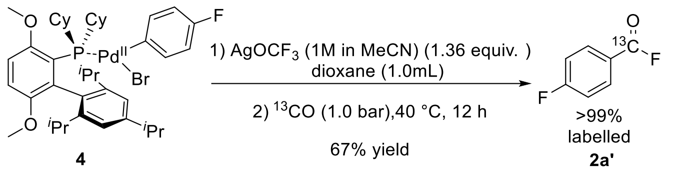

In a N_2 glovebox, to Pd(II)complex **4** (20.5mg, 0.025 mmol) in a 5.0 mL Schlenk tube, were added 1,4-dioxane (1.0 mL) and $AgOCF_3$ (1.0 mol/L in MeCN) (34 μL, 1.36 equiv), the reaction mixture was stirred for 5 minutes at room temperature. Then the sealed vial was taken outside the glovebox, ^{13}CO (1.0 bar) was replaced twice, the reaction mixture was stirred for 12 hours at 40 °C. The yield of [^{13}C]4-fluoro-Benzoyl fluoride (**2a'**) was determined by comparing the integration of the ^{19}F NMR resonance of [^{13}C]4-fluoro-Benzoyl fluoride (-100.73 ppm) with that of 1-Fluoro-3-nitrobenzene (-112.0 ppm).

[^{13}C]4-fluoro-Benzoyl fluoride (**2a'**) HRMS-EI (m/z): Calcd for $C_6{}^{13}CH_4F_2O$ $[M]^+$, 143.0264. Found, 143.0258.

Supplementary Scheme 2: ^{19}F NMR spectrum of **2a** and **2a'**

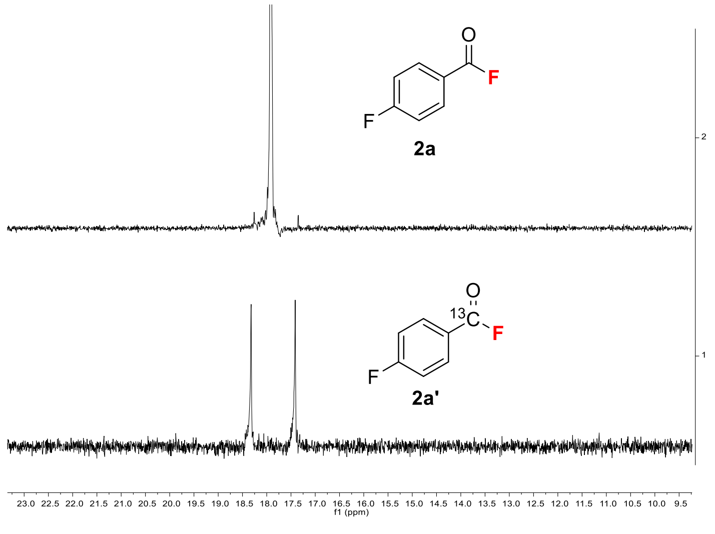

Supplementary Scheme 3: HRMS spectrum of **2a'**

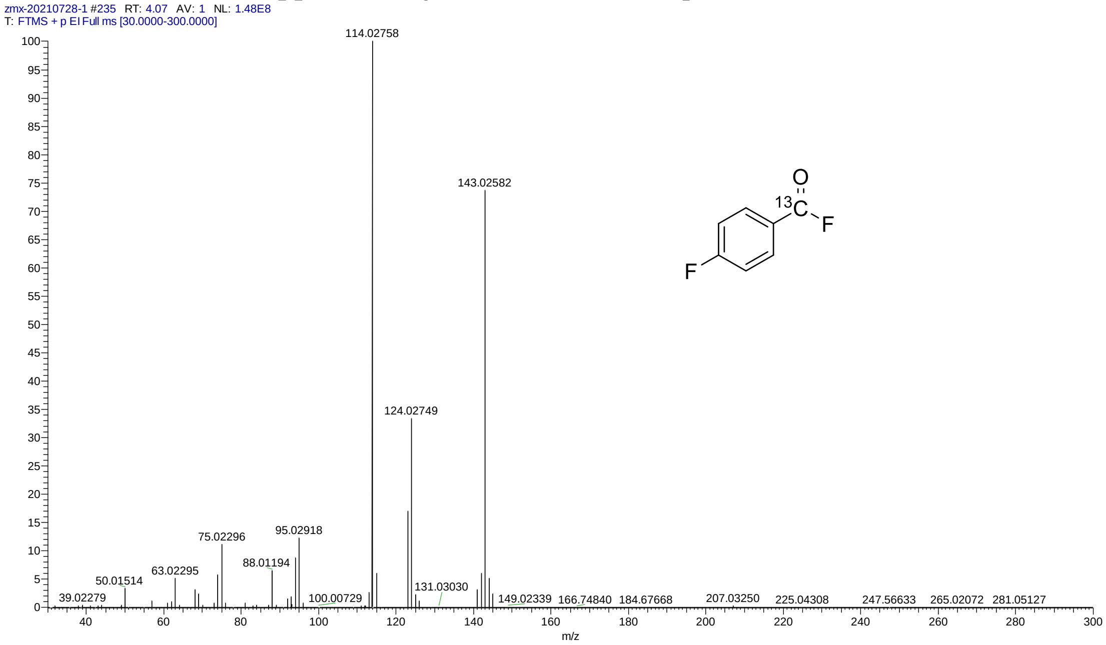

^{19}F NMR Monitoring Experiment

In a N_2 glovebox, to Pd(II)complex (**6**) (0.05 mmol, 39.4 mg, 1.0 equiv. and Tris(dimethylamino)sulfonium trimethylsilyldifluoride (TASF) (0.055 mmol, 15.1 mg, 1.1 equiv.) in a NMR tube. The stopper was tight, and then the NMR tube was taken outside the glovebox. The NMR tube was performed NMR scans to get the (t = 0) ^{19}F NMR spectrum. Then, TFMS (0.06 mmol, 9.6 μL, 1.2 equiv.) was injected into the NMR tube using micro sampler. Then, the (t = 353 s, 578 s, 912 s, 1284 s, 1672 s) ^{19}F NMR spectrum was obtained.

Computational studies

a.Computational Methods

All density functional theory (DFT) calculations were conducted with the Gaussian 09 software package[19]. Geometry optimizations of all the intermediates and transition states were performed in the gas phase at the B3LYP[20,21] level of theory with Grimme's D3 dispersion corrections[22],

and a mixed basis set, in which the pople's double-ζ basis set 6-31G(d) was adopted for H, B, C, O, F, P, and K atoms and SDD[23] was employed for Pd and Ag.

Based on the optimized structures, the zero-point energy (ZPE) and thermal energy corrections were calculated. The single-point energies were computed with M06L method (with the def2-TZVP[24] basis set for Pd, Ag, and 6-311+G (d, p) basis set for other atoms), including solvation energy corrections and Grimme's D3 dispersion corrections. The solvation energies were evaluated by a self-consistent reaction field (SCRF) using SMD model[25]. Intrinsic reaction coordinate (IRC)[26] calculations were carried out to confirm the transition states connecting the correct intermediates, reactants or products on the potential energy surface. The 3D diagrams of molecules were generated using CYLView[27].

b.The comparison of the direct reductive elimination to β-F elimination

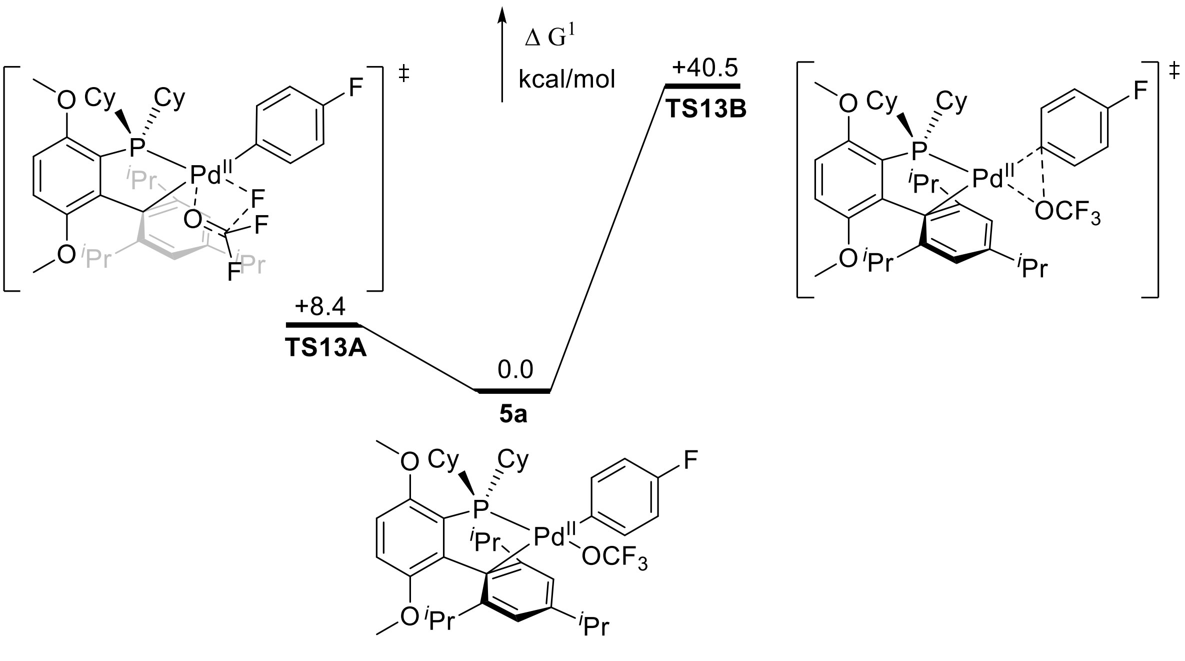

Supplementary Fig. 1. Comparison of energy barriers between two transition states **TS13A** and **TS13B**. Computed at the SMD(1,4-Dioxane)/M06L/6-311+G (d,p)/Def2-TZVP(Pd,Ag)// B3LYP-D3/6-31G(d)/SDD(Pd, Ag) .

c.Other Transition States

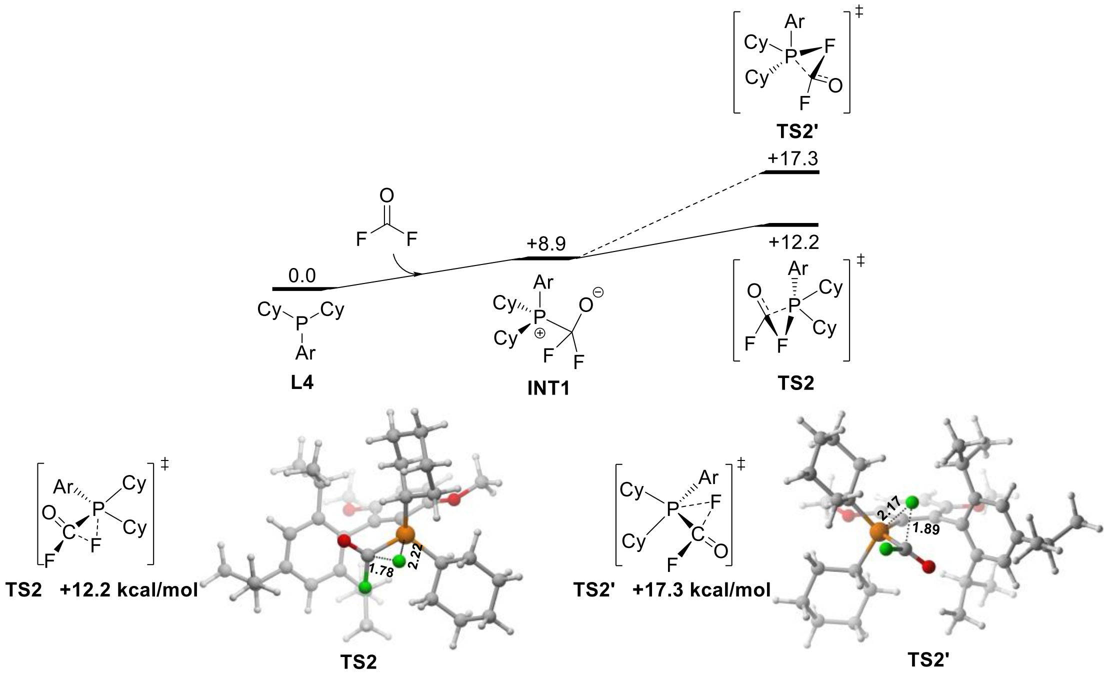

Supplementary Fig. 2. Other transition states of the F⁻ migration step. Take **L4** as zero point. **TS2** and **TS2'** are two transition states in which F⁻ migrates in different directions. In **TS2**, F⁻ migrates to the para position of the aryl group, while in **TS2'**, F⁻ migrates to the para position of the cyclohexyl group. The energy barrier of **TS2'** is 5.1 kcal/mol higher than that of **TS2**, probably due to the trans-effect contribution of aryl group or cyclohexyl group.

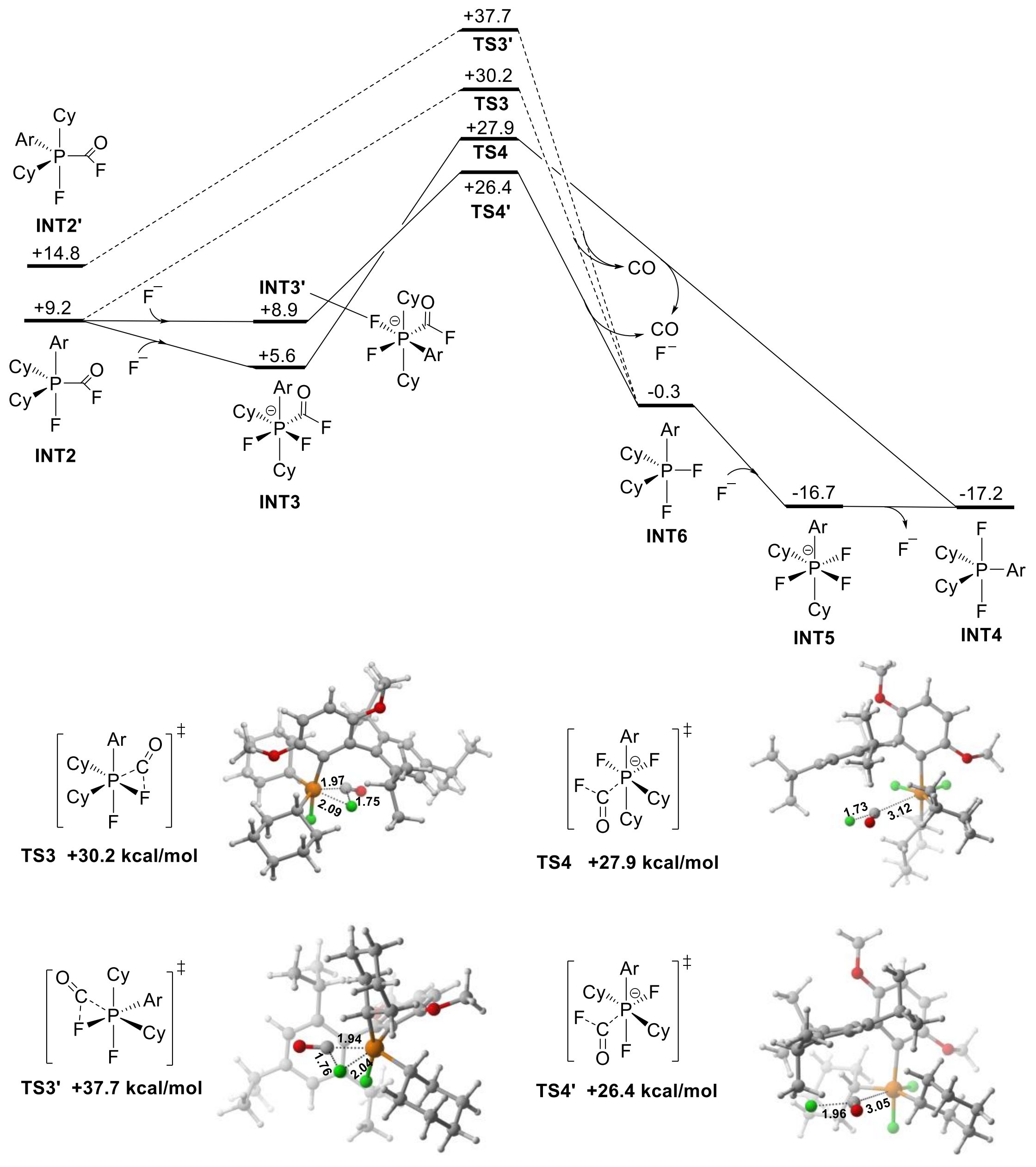

Supplementary Fig. 3. Other transition states of the CO release step. Take **L4** as zero point. We tried transition states with different configurations of the CO release step. Four representative models were found, in which the **TS3** and **TS3'** transition state structures are triangular. **TS4** can generate trans-difluoro intermediate **INT4**, and **TS3**, **TS3'** and **TS4'** can generate cis-difluoro intermediate **INT6**, which is thermodynamically unstable and can be converted to trans-difluoro intermediate **INT4**.

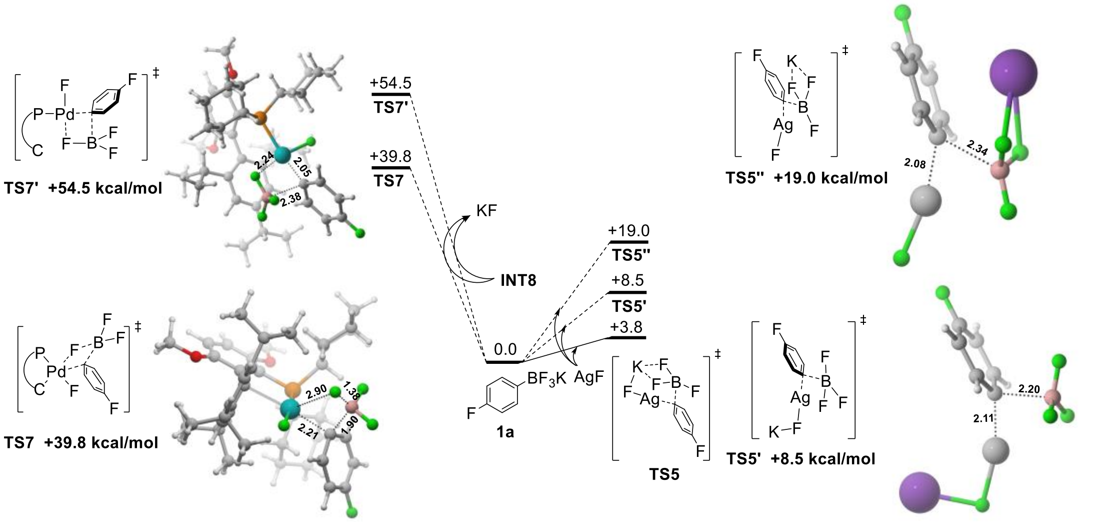

Supplementary Fig. 4. Other transition states of the transmetallation step. Take **1a** as zero point. We separately investigated the effect of K^+ on the energy barrier at different positions. It is found that the energy barrier is the lowest when K^+ interacts with AgF and BF_3. **TS7** and **TS7'** are transition states of transmetallation in the absence of AgF. In **TS7'**, Pd does not coordinate with C_{Ar} but with F in BF_3.

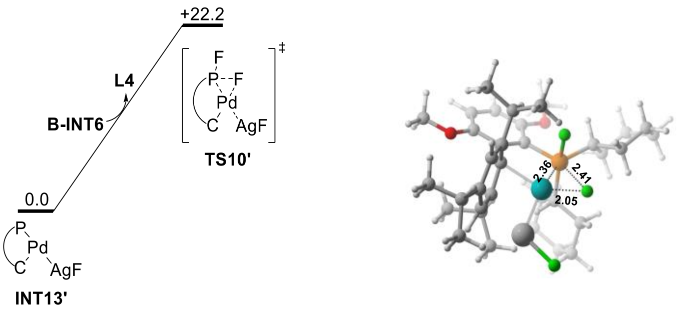

Supplementary Fig. 5. Other transition states of the oxidative addition step. Take **INT13'** as zero point. **TS10'** is the oxidative addition transition state with the participation of AgF. We found that with the participation of AgF, the energy barrier of oxidative addition did not decrease significantly, so AgF had little effect on the oxidative addition process, but the participation of AgF in the process could not be ruled out.

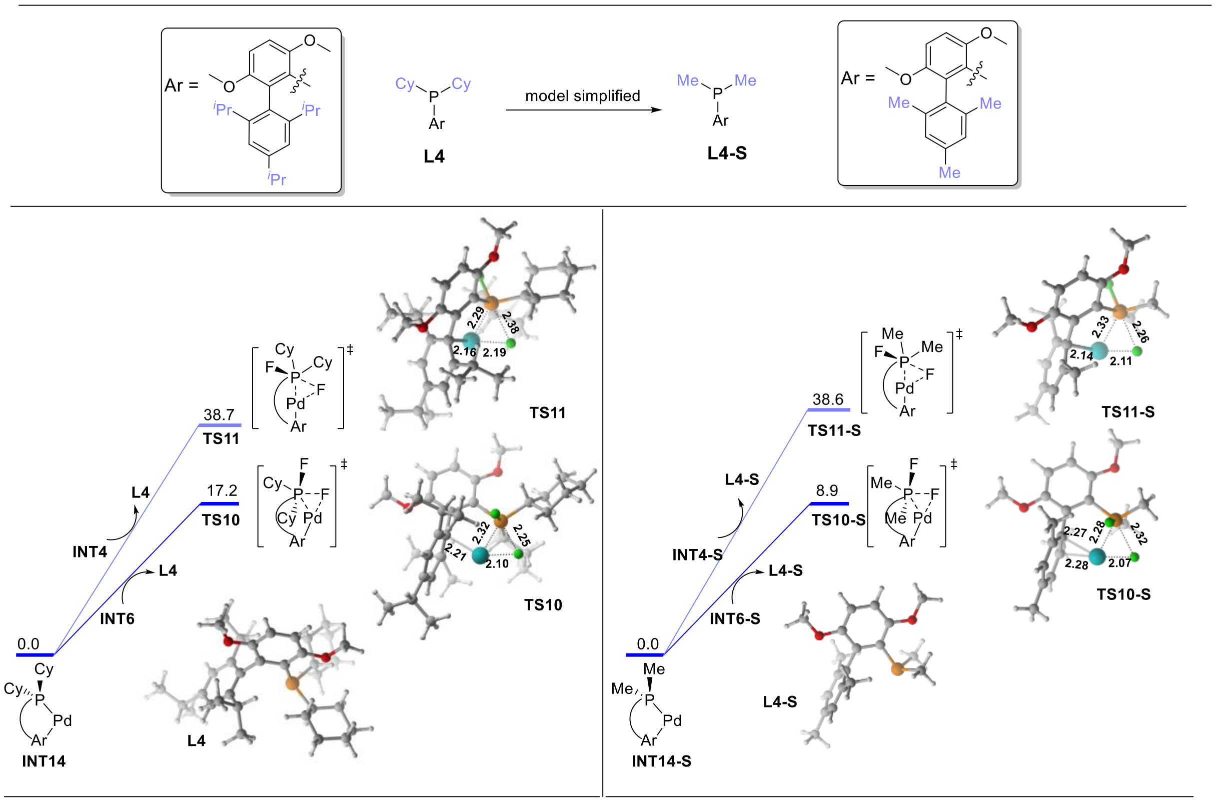

Supplementary Fig. 6. P-F oxidative addition with **INT14 as** zero point for full model and simplify model.

Supplementary Table 5. Relative energy differences of intermediate and transition state for full and simplified model.

	Full model		Simplify Model	
	**Intermediate relative energy difference**			
ΔΔG	**INT4**	-16.9	**INT4-S**	-17.3
(kcal/mol)	**INT6**	0.0	**INT6-S**	0.0
	**Transition state relative energy difference**			
$\Delta\Delta G^{\ddagger}$	**TS10**	0.0	**TS10-S**	0.0
(kcal/mol)	**TS11**	+4.6	**TS11-S**	+12.5

And if we set to zero-energy based on **INT14**, the energy profile would be as shown in Figure S6, suggesting the *trans*-difluoro P(V) (**INT4**) is inert for the next P-F oxidative addition. To ensure the rationality of the calculation, we also validated the reaction process using simplified model and obtained the same conclusion that *cis*-difluoro P(V) **INT6 (**or **INT6-S)** is the plausible active intermediate in the P-F oxidative addition.

d. Computed Energies of the Stationary Points

Supplementary Table 6. Zero-point correction (ZPE), electronic energies (E), enthalpies (H), and free energies (G) (in Hartree) of the structures calculated and imaginary frequency transition state at SMD(1,4-Dioxane)/M06L/6-311+G (d,p)/Def2-TZVP(Pd,Ag)// B3LYP-D3/6-31G(d)/SDD(Pd,Ag) theory.

	**ZPE**	**E**	**H**	**G**	**E(solvent)**
F^-	0.000000	-99.754089	-99.751728	-99.768247	-99.939404
BF_3	0.012543	-324.553919	-324.536934	-324.565844	-324.611110
CO	0.005032	-113.309455	-113.301119	-113.323562	-113.325182
COF_2	0.014114	-313.008512	-312.990148	-313.020233	-313.075804
KF	0.001035	-699.756262	-699.751663	-699.777321	-699.844979
AgF	0.001211	-246.830032	-246.825315	-246.852017	-246.961732
cod	0.181349	-312.046092	-311.856415	-311.896214	-312.069048
Pd(cod)F_2	0.188496	-639.703112	-639.502641	-639.551101	-639.863023
1a	0.095303	-1255.422505	-1255.315025	-1255.366642	-1255.550686
1a-AgF	0.097082	-1502.274888	-1502.161227	-1502.228430	-1502.539695
2a	0.094914	-544.076492	-543.972897	-544.014750	-544.175916
L4	0.818935	-1857.582416	-1856.722773	-1856.833959	-1857.667171
5a	0.922645	-2729.337604	-2728.360160	-2728.503120	-2729.639391
INT1	0.835333	-2170.602802	-2169.721800	-2169.844054	-2170.750739
INT2	0.836121	-2170.601486	-2169.720045	-2169.839862	-2170.753239
INT3	0.832068	-2270.575241	-2269.696428	-2269.818863	-2270.707326
INT4	0.827258	-2057.326701	-2056.456294	-2056.572119	-2057.448918
INT5	0.826452	-2157.225023	-2156.354372	-2156.471740	-2157.400459
INT6	0.826942	-2057.300092	-2056.429920	-2056.546137	-2057.421433
INT7	0.083720	-1177.707457	-1177.612128	-1177.664164	-1177.895460
INT8	0.826258	-2185.249651	-2184.377394	-2184.499394	-2185.481667
INT9	0.909514	-2663.175726	-2662.211633	-2662.353239	-2663.535717
INT10	0.909245	-2663.187514	-2662.223474	-2662.366158	-2663.546471
INT11	0.906516	-2416.292442	-2415.335073	-2415.467800	-2416.538647
INT12	0.915562	-2529.609726	-2528.641055	-2528.779621	-2529.871983
INT13	0.916559	-2529.650110	-2528.680762	-2528.819343	-2529.918159
INT14	0.819598	-1985.523750	-1984.660586	-1984.779395	-1985.705428
INT15	0.825926	-2185.204028	-2184.332729	-2184.452676	-2185.435690
INT16	0.825928	-2185.209160	-2184.337307	-2184.459591	-2185.445330
TS1	0.834816	-2170.602414	-2169.722508	-2169.843056	-2170.747156
TS2	0.835588	-2170.591631	-2169.711178	-2169.830503	-2170.747869
TS2'	0.834742	-2170.583415	-2169.703621	-2169.823384	-2170.738581
TS3	0.833964	-2170.562902	-2169.683698	-2169.804135	-2170.716922
TS3'	0.834164	-2170.551176	-2169.671880	-2169.791655	-2170.705727
TS4	0.829271	-2270.535289	-2269.658285	-2269.783286	-2270.667380

TS4'	0.831818	-2270.539933	-2269.661166	-2269.783011	-2270.674784
TS5	0.096656	-1502.288101	-1502.174509	-1502.239231	-1502.521379
TS5'	0.096135	-1502.271646	-1502.159342	-1502.223265	-1502.513403
TS5''	0.096135	-1502.254214	-1502.142227	-1502.203235	-1502.499307
TS6	0.908561	-2663.170305	-2662.207755	-2662.348889	-2663.527745
TS7	0.920583	-2740.835250	-2739.860279	-2739.999390	-2741.132642
TS7'	0.919716	-2740.819941	-2739.845177	-2739.988123	-2741.105200
TS8	0.914466	-2529.594463	-2528.627419	-2528.764667	-2529.864128
TS9	0.915608	-2529.631147	-2528.663382	-2528.800389	-2529.899152
TS10	0.825752	-2185.198904	-2184.328252	-2184.447303	-2185.433991
TS10'	0.827780	-2432.084244	-2431.207687	-2431.336472	-2432.442318
TS11	0.824448	-2185.186314	-2184.316786	-2184.436635	-2185.424729
TS12	0.825727	-2185.202308	-2184.331173	-2184.452383	-2185.437775
TS13A	0.921719	-2729.318560	-2728.342390	-2728.484076	-2729.625963
TS13B	0.921204	-2729.275513	-2728.300059	-2728.441491	-2729.574402

Crystallographic Data

X-ray Crystal Structure Data for Pd(II)-OCF3 complex (5a) (CCDC: 2099433)

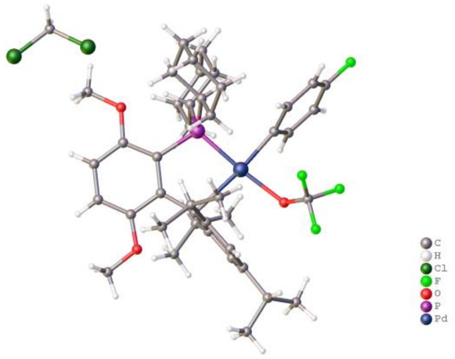

Supplementary Table 7. Crystal data and structure refinement for 5a

Identification code	**5a**
CCDC	2099433
Empirical formula	$C_{43}H_{59}Cl_2F_4O_3PPd$
Formula weight	908.17
Temperature/K	113.15
Crystal system	triclinic
Space group	P-1
a/Å	13.2645(5)
b/Å	13.8896(5)

c/Å	13.9372(6)
α/°	69.742(4)
β/°	84.274(3)
γ/°	64.005(4)
Volume/$Å^3$	2161.11(17)
Z	2
$\rho_{calc}g/cm^3$	1.396
μ/mm^{-1}	0.644
F(000)	944.0
Crystal size/mm^3	0.16 × 0.14 × 0.13
Radiation	MoKα (λ = 0.71073)
2Θ range for data collection/°	3.422 to 52.744
Index ranges	-16 ≤ h ≤ 16, -17 ≤ k ≤ 17, -17 ≤ l ≤ 17
Reflections collected	23322
Independent reflections	8807 [R_{int} = 0.0550, R_{sigma} = 0.0663]
Data/restraints/parameters	8807/1/495
Goodness-of-fit on F^2	1.058
Final R indexes [I>=2σ (I)]	R_1 = 0.0489, wR_2 = 0.1122
Final R indexes [all data]	R_1 = 0.0625, wR_2 = 0.1208
Largest diff. peak/hole / e $Å^{-3}$	1.37/-1.21

Supplementary Table 8. Fractional Atomic Coordinates (×10^4) and Equivalent Isotropic Displacement Parameters (Å^2×10^3) for 5a U_{eq} is defined as 1/3 of of the trace of the orthogonalised U_{IJ}tensor.

Atom	*x*	*y*	*z*	U(eq)
Pd1	3297.4(2)	6094.5(2)	7454.7(2)	17.61(9)
P1	1743.0(7)	5982.2(7)	7148.2(7)	17.11(19)
F1	5203(2)	6301(2)	6011.2(18)	45.5(6)
F2	5899(2)	6954(3)	6831(2)	59.8(8)
F3	6514(2)	5157(3)	7189(2)	64.3(9)
F4	6367(2)	1311.5(19)	7361.6(19)	46.4(7)
O1	4823.4(19)	6129(2)	7654.8(18)	22.6(5)
O2	-792(2)	6692(2)	7049(2)	30.8(6)
O3	349(2)	9541(2)	8166(2)	28.6(6)
C1	5536(3)	6125(4)	6990(3)	30.9(9)
C2	4207(3)	4546(3)	7349(3)	20.2(7)
C3	4476(3)	4319(3)	6437(3)	22.0(8)
C4	5207(3)	3228(3)	6435(3)	25.2(8)
C5	5650(3)	2386(3)	7353(3)	29.6(9)
C6	5433(3)	2572(3)	8275(3)	31.2(9)

C7	4709(3)	3660(3)	8261(3)	26.7(8)
C8	1675(3)	6001(3)	5822(3)	18.9(7)
C9	1993(3)	6900(3)	5035(3)	20.9(7)
C10	2164(3)	6723(3)	3996(3)	26.5(8)
C11	1136(3)	6723(3)	3602(3)	29.3(9)
C12	806(3)	5848(3)	4393(3)	29.8(9)
C13	614(3)	6050(3)	5426(3)	24.2(8)
C14	1443(3)	4763(3)	7946(3)	21.7(7)
C15	2079(3)	3669(3)	7695(3)	29.7(9)
C16	1624(4)	2805(3)	8306(3)	32.5(9)
C17	1722(4)	2560(4)	9450(3)	43.6(11)
C18	1147(5)	3649(4)	9704(3)	46.1(12)
C19	1579(4)	4529(3)	9088(3)	33.0(9)
C20	575(3)	7223(3)	7364(2)	17.2(7)
C21	-568(3)	7441(3)	7315(3)	22.0(8)
C22	-1395(3)	8352(3)	7548(3)	25.2(8)
C23	-1109(3)	9065(3)	7832(3)	25.0(8)
C24	-4(3)	8878(3)	7890(3)	22.9(8)
C25	856(3)	7956(3)	7650(2)	17.4(7)
C26	-1917(3)	6833(4)	7037(4)	38.5(10)
C27	-476(4)	10437(3)	8477(3)	37.0(10)
C28	2028(3)	7858(3)	7727(3)	18.4(7)
C29	2377(3)	8566(3)	6887(3)	19.4(7)
C30	3257(3)	8782(3)	7057(3)	22.4(8)
C31	3796(3)	8366(3)	8012(3)	24.5(8)
C32	3450(3)	7676(3)	8830(3)	23.9(8)
C33	2585(3)	7415(3)	8715(3)	21.0(7)
C34	1772(3)	9136(3)	5826(3)	19.8(7)
C35	924(3)	10365(3)	5666(3)	29.1(9)
C36	2586(3)	9097(3)	4966(3)	28.5(8)
C37	4710(3)	8705(3)	8130(3)	29.6(9)
C38	4191(4)	9802(4)	8402(4)	50.2(13)
C39	5670(3)	7781(4)	8893(3)	34.1(9)
C40	2201(3)	6718(3)	9646(3)	25.4(8)
C41	1657(4)	7384(4)	10382(3)	38.1(10)
C42	3165(3)	5572(3)	10208(3)	34.1(9)
Cl1	-2236.4(13)	7670.6(13)	4240.1(12)	67.9(4)
Cl2	-4540.0(17)	8986(2)	4506(2)	161.7(14)
C43	-3516(6)	7734(6)	4348(5)	80.6(19)

Supplementary Table 9. Anisotropic Displacement Parameters (Å^2×10^3) for 5a The Anisotropic displacement factor exponent takes the form: -2π^2[h^{2}a*^{2}U$_{11}$+2hka*b*U$_{12}$+…].

Atom	U_{11}	U_{22}	U_{33}	U_{23}	U_{13}	U_{12}
Pd1	13.66(14)	17.17(14)	20.13(15)	-7.81(10)	1.04(10)	-3.90(10)
P1	14.9(4)	17.4(4)	18.4(4)	-7.4(3)	2.5(3)	-5.8(3)
F1	52.1(16)	69.3(18)	27.3(13)	-12.1(12)	4.2(11)	-40.6(15)
F2	55.5(18)	91(2)	73(2)	-48.7(18)	29.6(15)	-56.3(18)
F3	32.2(15)	76(2)	57.0(18)	-33.6(16)	4.3(13)	9.0(14)
F4	42.9(15)	25.7(12)	49.8(16)	-17.4(11)	5.3(12)	6.2(11)
O1	17.5(12)	25.2(13)	27.8(13)	-11.7(11)	0.3(9)	-9.2(10)
O2	15.5(13)	40.6(16)	44.1(17)	-22.3(13)	5.7(11)	-13.7(12)
O3	29.3(14)	21.5(13)	34.7(15)	-17.6(12)	4.9(12)	-5.2(11)
C1	20.8(19)	41(2)	33(2)	-17.4(18)	2.2(13)	-11.7(17)
C2	14.2(16)	22.4(18)	23.5(18)	-8.4(14)	2.6(13)	-7.2(14)
C3	14.7(17)	24.6(19)	25.0(19)	-10.4(15)	5.4(14)	-6.3(14)
C4	19.4(18)	30(2)	26(2)	-15.2(16)	7.2(15)	-7.6(16)
C5	19.5(19)	20.3(19)	41(2)	-15.0(17)	6.1(16)	0.6(15)
C6	26(2)	23(2)	32(2)	-4.9(16)	1.3(16)	-2.9(16)
C7	26(2)	27(2)	22.1(19)	-10.6(15)	1.7(15)	-4.8(16)
C8	19.3(17)	18.5(17)	19.6(17)	-8.8(14)	3.3(13)	-7.4(14)
C9	20.5(18)	20.0(17)	21.6(18)	-6.1(14)	0.5(14)	-8.7(15)
C10	28(2)	28(2)	21.6(19)	-5.6(16)	3.4(15)	-13.8(17)
C11	34(2)	33(2)	23(2)	-11.8(16)	0.7(16)	-14.5(18)
C12	37(2)	34(2)	26(2)	-13.8(17)	0.3(17)	-19.0(18)
C13	26(2)	27.5(19)	24.5(19)	-10.2(16)	2.5(15)	-15.9(16)
C14	22.4(18)	24.3(18)	22.5(18)	-11.0(15)	6.4(14)	-12.2(15)
C15	35(2)	22.3(19)	29(2)	-7.7(16)	5.9(17)	-11.9(17)
C16	43(2)	23(2)	35(2)	-9.5(17)	7.0(18)	-18.5(18)
C17	62(3)	33(2)	36(2)	-1.9(19)	4(2)	-29(2)
C18	75(4)	47(3)	25(2)	-9(2)	13(2)	-38(3)
C19	45(2)	33(2)	23(2)	-9.0(17)	6.3(17)	-20.5(19)
C20	14.9(16)	17.7(17)	14.8(16)	-4.2(13)	1.3(13)	-4.5(13)
C21	21.7(18)	24.7(18)	18.1(18)	-5.1(14)	3.4(14)	-10.9(15)
C22	14.4(17)	29(2)	25.7(19)	-7.6(16)	5.8(14)	-6.2(15)
C23	18.6(18)	22.2(18)	22.6(19)	-5.7(15)	3.6(14)	-0.7(15)
C24	24.3(19)	18.4(17)	17.3(17)	-5.2(14)	1.9(14)	-2.3(15)
C25	14.6(16)	16.5(16)	15.0(16)	-4.3(13)	2.9(13)	-2.7(13)
C26	22(2)	55(3)	52(3)	-31(2)	8.6(19)	-20(2)
C27	44(3)	22(2)	44(3)	-21.5(19)	10(2)	-6.5(18)

C28	18.3(17)	14.8(16)	20.8(18)	-8.9(14)	0.4(13)	-3.6(14)
C29	16.3(17)	17.5(17)	21.9(18)	-8.2(14)	1.8(13)	-4.1(14)
C30	20.7(18)	18.9(17)	26.2(19)	-6.7(15)	0.0(14)	-7.6(15)
C31	22.5(19)	20.7(18)	30(2)	-11.2(15)	-1.2(15)	-6.7(15)
C32	23.4(19)	22.4(18)	20.8(18)	-9.1(15)	-7.4(14)	-2.4(15)
C33	19.5(18)	18.3(17)	22.0(18)	-9.6(14)	-0.6(14)	-3.0(14)
C34	19.6(18)	16.4(17)	22.3(18)	-3.5(14)	-0.2(14)	-8.8(14)
C35	27(2)	21.1(19)	30(2)	-1.4(16)	-8.0(16)	-5.4(16)
C36	30(2)	31(2)	22.4(19)	-5.8(16)	4.4(16)	-14.9(17)
C37	29(2)	30(2)	31(2)	-8.8(17)	-4.4(16)	-14.4(17)
C38	44(3)	33(2)	80(4)	-27(2)	-10(2)	-14(2)
C39	25(2)	40(2)	35(2)	-8.2(19)	-3.7(17)	-14.9(18)
C40	30(2)	25.2(19)	19.2(18)	-8.1(15)	0.8(15)	-9.8(16)
C41	47(3)	34(2)	28(2)	-14.2(18)	10.4(19)	-11(2)
C42	40(2)	28(2)	25(2)	-4.8(17)	0.8(17)	-10.5(18)
Cl1	70.5(10)	71.3(9)	71.9(10)	-29.7(8)	1.8(7)	-34.6(8)
Cl2	61.6(12)	111.5(16)	187(3)	46.3(16)	43.2(14)	-9.8(11)
C43	90(5)	89(5)	63(4)	-28(4)	1(3)	-36(4)

Supplementary Table 10. Bond Lengths for 5a

Atom	Atom	Length/Å	Atom	Atom	Length/Å
Pd1	P1	2.2275(9)	C14	C19	1.523(5)
Pd1	O1	2.094(2)	C15	C16	1.535(5)
Pd1	C2	2.003(3)	C16	C17	1.516(6)
Pd1	C28	2.425(3)	C17	C18	1.517(6)
P1	C8	1.851(3)	C18	C19	1.532(5)
P1	C14	1.860(4)	C20	C21	1.414(5)
P1	C20	1.840(3)	C20	C25	1.405(5)
F1	C1	1.382(4)	C21	C22	1.381(5)
F2	C1	1.377(5)	C22	C23	1.383(5)
F3	C1	1.362(5)	C23	C24	1.378(5)
F4	C5	1.370(4)	C24	C25	1.412(5)
O1	C1	1.255(4)	C25	C28	1.512(5)
O2	C21	1.366(4)	C28	C29	1.431(5)
O2	C26	1.418(4)	C28	C33	1.427(5)
O3	C24	1.369(4)	C29	C30	1.386(5)
O3	C27	1.420(4)	C29	C34	1.525(5)
C2	C3	1.390(5)	C30	C31	1.383(5)
C2	C7	1.393(5)	C31	C32	1.397(5)

C3	C4	1.397(5)	C31	C37	1.520(5)
C4	C5	1.364(5)	C32	C33	1.385(5)
C5	C6	1.376(5)	C33	C40	1.521(5)
C6	C7	1.386(5)	C34	C35	1.528(5)
C8	C9	1.536(5)	C34	C36	1.531(5)
C8	C13	1.528(5)	C37	C38	1.538(6)
C9	C10	1.530(5)	C37	C39	1.514(5)
C10	C11	1.520(5)	C40	C41	1.535(5)
C11	C12	1.525(5)	C40	C42	1.528(5)
C12	C13	1.537(5)	Cl1	C43	1.655(7)
C14	C15	1.525(5)	Cl2	C43	1.747(7)

Supplementary Table 11. Bond Angles for 5a

Atom	Atom	Atom	Angle/°	Atom	Atom	Atom	Angle/°
P1	Pd1	C28	85.32(8)	C16	C17	C18	111.1(4)
O1	Pd1	P1	175.17(7)	C17	C18	C19	111.9(4)
O1	Pd1	C28	98.80(10)	C14	C19	C18	110.1(3)
C2	Pd1	P1	89.02(10)	C21	C20	P1	124.0(3)
C2	Pd1	O1	87.13(12)	C25	C20	P1	117.2(2)
C2	Pd1	C28	172.01(12)	C25	C20	C21	118.8(3)
C8	P1	Pd1	110.34(11)	O2	C21	C20	116.4(3)
C8	P1	C14	104.20(15)	O2	C21	C22	122.8(3)
C14	P1	Pd1	121.18(12)	C22	C21	C20	120.8(3)
C20	P1	Pd1	105.23(11)	C21	C22	C23	120.0(3)
C20	P1	C8	111.99(15)	C24	C23	C22	120.9(3)
C20	P1	C14	103.80(15)	O3	C24	C23	124.5(3)
C1	O1	Pd1	124.6(2)	O3	C24	C25	115.3(3)
C21	O2	C26	118.9(3)	C23	C24	C25	120.2(3)
C24	O3	C27	117.4(3)	C20	C25	C24	119.4(3)
F2	C1	F1	102.7(3)	C20	C25	C28	125.7(3)
F3	C1	F1	102.6(3)	C24	C25	C28	114.9(3)
F3	C1	F2	102.8(3)	C25	C28	Pd1	106.3(2)
O1	C1	F1	116.9(3)	C29	C28	Pd1	95.1(2)
O1	C1	F2	113.8(3)	C29	C28	C25	118.1(3)
O1	C1	F3	116.1(4)	C33	C28	Pd1	88.4(2)
C3	C2	Pd1	125.0(3)	C33	C28	C25	119.1(3)
C3	C2	C7	118.1(3)	C33	C28	C29	118.9(3)
C7	C2	Pd1	116.5(3)	C28	C29	C34	121.9(3)
C2	C3	C4	121.0(3)	C30	C29	C28	118.8(3)

C5	C4	C3	118.4(3)	C30	C29	C34	119.2(3)
F4	C5	C6	118.1(3)	C31	C30	C29	122.8(3)
C4	C5	F4	118.9(3)	C30	C31	C32	118.1(3)
C4	C5	C6	122.9(3)	C30	C31	C37	118.7(3)
C5	C6	C7	117.8(4)	C32	C31	C37	123.2(3)
C6	C7	C2	121.8(3)	C33	C32	C31	122.3(3)
C9	C8	P1	112.8(2)	C28	C33	C40	121.0(3)
C13	C8	P1	118.4(2)	C32	C33	C28	119.1(3)
C13	C8	C9	109.8(3)	C32	C33	C40	119.8(3)
C10	C9	C8	110.9(3)	C29	C34	C35	111.2(3)
C11	C10	C9	111.8(3)	C29	C34	C36	112.5(3)
C10	C11	C12	110.8(3)	C35	C34	C36	109.9(3)
C11	C12	C13	111.2(3)	C31	C37	C38	109.9(3)
C8	C13	C12	110.1(3)	C39	C37	C31	114.2(3)
C15	C14	P1	115.4(2)	C39	C37	C38	110.8(3)
C19	C14	P1	112.2(2)	C33	C40	C41	110.8(3)
C19	C14	C15	110.7(3)	C33	C40	C42	111.9(3)
C14	C15	C16	109.1(3)	C42	C40	C41	110.2(3)
C17	C16	C15	111.7(3)	Cl1	C43	Cl2	114.2(4)

Supplementary Table 12. Torsion Angles for 5a

A	B	C	D	Angle/°	A	B	C	D	Angle/°
Pd1	P1	C8	C9	-43.9(3)	C19	C14	C15	C16	59.1(4)
Pd1	P1	C8	C13	-174.0(2)	C20	P1	C8	C9	73.0(3)
Pd1	P1	C14	C15	-78.8(3)	C20	P1	C8	C13	-57.2(3)
Pd1	P1	C14	C19	49.2(3)	C20	P1	C14	C15	163.5(3)
Pd1	P1	C20	C21	-175.9(3)	C20	P1	C14	C19	-68.5(3)
Pd1	P1	C20	C25	0.4(3)	C20	C21	C22	C23	0.1(5)
Pd1	O1	C1	F1	11.2(5)	C20	C25	C28	Pd1	-6.3(4)
Pd1	O1	C1	F2	130.7(3)	C20	C25	C28	C29	98.7(4)
Pd1	O1	C1	F3	-110.2(3)	C20	C25	C28	C33	-103.7(4)
Pd1	C2	C3	C4	173.6(3)	C21	C20	C25	C24	0.6(5)
Pd1	C2	C7	C6	-174.5(3)	C21	C20	C25	C28	-179.1(3)
Pd1	C28	C29	C30	-89.9(3)	C21	C22	C23	C24	-0.2(5)
Pd1	C28	C29	C34	93.3(3)	C22	C23	C24	O3	-179.9(3)
Pd1	C28	C33	C32	94.4(3)	C22	C23	C24	C25	0.5(5)
Pd1	C28	C33	C40	-88.9(3)	C23	C24	C25	C20	-0.7(5)
P1	C8	C9	C10	168.2(2)	C23	C24	C25	C28	179.0(3)
P1	C8	C13	C12	-170.1(2)	C24	C25	C28	Pd1	173.9(2)

P1	C14	C15	C16	-172.1(3)	C24	C25	C28	C29	-81.0(4)
P1	C14	C19	C18	171.1(3)	C24	C25	C28	C33	76.5(4)
P1	C20	C21	O2	-2.7(4)	C25	C20	C21	O2	-179.0(3)
P1	C20	C21	C22	176.0(3)	C25	C20	C21	C22	-0.3(5)
P1	C20	C25	C24	-175.9(3)	C25	C28	C29	C30	158.6(3)
P1	C20	C25	C28	4.4(4)	C25	C28	C29	C34	-18.2(5)
F4	C5	C6	C7	179.9(3)	C25	C28	C33	C32	-157.8(3)
O2	C21	C22	C23	178.7(3)	C25	C28	C33	C40	18.9(5)
O3	C24	C25	C20	179.6(3)	C26	O2	C21	C20	176.8(3)
O3	C24	C25	C28	-0.6(4)	C26	O2	C21	C22	-1.8(5)
C2	C3	C4	C5	0.2(5)	C27	O3	C24	C23	4.3(5)
C3	C2	C7	C6	-1.7(6)	C27	O3	C24	C25	-176.1(3)
C3	C4	C5	F4	179.9(3)	C28	C29	C30	C31	-1.6(5)
C3	C4	C5	C6	-1.9(6)	C28	C29	C34	C35	97.2(4)
C4	C5	C6	C7	1.7(6)	C28	C29	C34	C36	-139.0(3)
C5	C6	C7	C2	0.2(6)	C28	C33	C40	C41	-110.1(4)
C7	C2	C3	C4	1.5(5)	C28	C33	C40	C42	126.5(4)
C8	P1	C14	C15	46.2(3)	C29	C28	C33	C32	-0.4(5)
C8	P1	C14	C19	174.2(3)	C29	C28	C33	C40	176.2(3)
C8	P1	C20	C21	64.2(3)	C29	C30	C31	C32	1.6(5)
C8	P1	C20	C25	-119.5(3)	C29	C30	C31	C37	-176.7(3)
C8	C9	C10	C11	55.8(4)	C30	C29	C34	C35	-79.6(4)
C9	C8	C13	C12	58.4(4)	C30	C29	C34	C36	44.2(4)
C9	C10	C11	C12	-54.6(4)	C30	C31	C32	C33	-0.9(5)
C10	C11	C12	C13	55.7(4)	C30	C31	C37	C38	89.8(4)
C11	C12	C13	C8	-58.0(4)	C30	C31	C37	C39	-145.0(4)
C13	C8	C9	C10	-57.5(4)	C31	C32	C33	C28	0.4(5)
C14	P1	C8	C9	-175.5(2)	C31	C32	C33	C40	-176.3(3)
C14	P1	C8	C13	54.4(3)	C32	C31	C37	C38	-88.3(4)
C14	P1	C20	C21	-47.6(3)	C32	C31	C37	C39	36.9(5)
C14	P1	C20	C25	128.7(3)	C32	C33	C40	C41	66.6(4)
C14	C15	C16	C17	-57.6(5)	C32	C33	C40	C42	-56.8(4)
C15	C14	C19	C18	-58.4(4)	C33	C28	C29	C30	1.0(5)
C15	C16	C17	C18	55.2(5)	C33	C28	C29	C34	-175.8(3)
C16	C17	C18	C19	-54.0(5)	C34	C29	C30	C31	175.3(3)
C17	C18	C19	C14	55.6(5)	C37	C31	C32	C33	177.2(3)

Supplementary Table 13. Hydrogen Atom Coordinates (Å×10^4) and Isotropic Displacement Parameters (Å^2×10^3) for 5a

Atom	x	y	z	U(eq)
H3	4157.25	4915.39	5804.74	26
H4	5390.59	3076.03	5809.78	30
H6	5769.55	1972.79	8901.42	37
H7	4549.7	3806.43	8889.59	32
H8	2287.02	5253.96	5818.05	23
H9A	1389.67	7668.93	4955.6	25
H9B	2693.19	6849.27	5285.37	25
H10A	2325.67	7336.79	3493.54	32
H10B	2821.57	5988.59	4061.91	32
H11A	1299.5	6543.73	2959.21	35
H11B	501.47	7488.82	3448.95	35
H12A	1407.82	5073.76	4489.48	36
H12B	109.57	5894.87	4137.91	36
H13A	-19.99	6803.98	5341.64	29
H13B	417.71	5462.04	5929.24	29
H14	629.11	5005.75	7797.23	26
H15A	2891.46	3352.81	7876.43	36
H15B	1980.07	3832.22	6952.2	36
H16A	825.55	3106.08	8084.57	39
H16B	2048.32	2087.39	8158.51	39
H17A	2525.34	2174.15	9688.08	52
H17B	1374.06	2041.82	9814.41	52
H18A	1280.62	3471.17	10444.77	55
H18B	326.55	3976.37	9555.13	55
H19A	1151.64	5244.4	9238.9	40
H19B	2380.99	4239.88	9289.71	40
H22	-2160.27	8489.07	7512.63	30
H23	-1682.38	9691.94	7989.24	30
H26A	-2392.39	7590.81	6564.91	58
H26B	-2187.3	6752.74	7728.18	58
H26C	-1947.87	6251.99	6810.67	58
H27A	-1041.44	10971.67	7911.2	55
H27B	-116.59	10835.83	8664.93	55
H27C	-838.33	10120.07	9068.96	55
H30	3499.84	9236.64	6494.25	27
H32	3822.21	7374.83	9487.29	29
H34	1345.51	8709.19	5782.32	24
H35A	362.32	10377.36	6180.57	44
H35B	549.78	10710.68	4979.12	44

H35C	1315.27	10796.66	5734.73	44
H36A	2872.92	9662.41	4876.02	43
H36B	2192.25	9268.42	4327	43
H36C	3214.65	8336.7	5144.58	43
H37	5032.99	8883.21	7447.38	35
H38A	3614.66	10407.81	7868.72	75
H38B	4778.86	10034.18	8447.57	75
H38C	3848.31	9664.27	9061.92	75
H39A	5421.37	7703	9591.09	51
H39B	6302.43	7982.51	8806.47	51
H39C	5906.59	7057.58	8776.23	51
H40	1619.1	6565.2	9402.29	31
H41A	2216.28	7527.99	10645.04	57
H41B	1374.75	6939.11	10955.11	57
H41C	1031.32	8110.64	10016.76	57
H42A	3509.38	5157.4	9728.82	51
H42B	2874.38	5125.16	10769.92	51
H42C	3729.21	5698.75	10484.38	51
H43A	-3500.55	7078.25	4942.01	97
H43B	-3733.14	7659.65	3727.02	97

X-ray Crystal Structure Data for dicyclohexyldifluoro(2',4',6'-triisopropyl-3,6-dimethoxy-(1,1'-biphenyl)-2-yl)-λ^5-phosphane (7) (CCDC: 2099395)

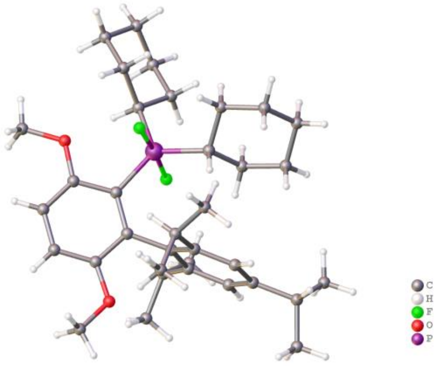

Supplementary Table 14. Crystal data and structure refinement for 7

Identification code	**7**
CCDC	2099395
Empirical formula	$C_{35}H_{53}F_2O_2P$
Formula weight	574.74
Temperature/K	113.15

Crystal system	monoclinic
Space group	$P2_1/n$
a/Å	10.6000(4)
b/Å	22.0535(7)
c/Å	14.1767(6)
α/°	90
β/°	95.291(3)
γ/°	90
Volume/Å^3	3299.9(2)
Z	4
ρ_{calc}g/cm^3	1.157
μ/mm^{-1}	0.123
F(000)	1248.0
Crystal size/mm^3	0.24 × 0.2 × 0.17
Radiation	MoKα (λ = 0.71073)
2Θ range for data collection/°	3.694 to 67.21
Index ranges	-15 ≤ h ≤ 14, -32 ≤ k ≤ 32, -21 ≤ l ≤ 21
Reflections collected	40012
Independent reflections	11924 [R_{int} = 0.0781, R_{sigma} = 0.0825]
Data/restraints/parameters	11924/0/370
Goodness-of-fit on F^2	1.044
Final R indexes [I>=2σ (I)]	R_1 = 0.0635, wR_2 = 0.1300
Final R indexes [all data]	R_1 = 0.1081, wR_2 = 0.1579
Largest diff. peak/hole / e Å^{-3}	0.43/-0.41

Supplementary Table 15. Fractional Atomic Coordinates (×10^4) and Equivalent Isotropic Displacement Parameters (Å^2×10^3) for 7 U_{eq} is defined as 1/3 of of the trace of the orthogonalised U_{IJ}tensor.

Atom	*x*	*y*	*z*	U(eq)
P1	7561.8(4)	3943.4(2)	4133.2(3)	20.19(10)
F1	6233.6(9)	3844.3(4)	4688.5(7)	24.5(2)
F2	8787.4(9)	4093.1(4)	3504.1(7)	26.1(2)
O1	6857.4(13)	5049.8(6)	5055.7(8)	31.9(3)
O2	5353.2(11)	5118.5(5)	1229.9(8)	24.7(3)
C1	6713.8(18)	5559.9(8)	5650.8(13)	31.4(4)
C2	6433.0(15)	5094.5(7)	4114.1(11)	21.4(3)
C3	6733.9(14)	4598.9(7)	3539.4(11)	19.2(3)
C4	6365.8(14)	4622.8(7)	2562.1(11)	17.6(3)
C5	5685.1(14)	5132.7(7)	2193.2(11)	19.4(3)
C6	5377.3(15)	5605.6(7)	2770.0(12)	23.3(3)

C7	5762.7(15)	5588.3(7)	3733.4(12)	23.2(3)
C8	4487.3(17)	5570.0(8)	844.5(13)	27.9(4)
C9	8600.2(16)	4060.6(8)	5237.4(12)	24.6(3)
C10	7983.7(18)	3968.0(9)	6169.0(12)	31.6(4)
C11	8888(2)	4174.4(10)	7009.3(14)	42.2(5)
C12	10148(2)	3838.9(10)	7051.4(15)	45.9(6)
C13	10750.4(19)	3908.3(10)	6122.6(16)	41.7(5)
C14	9855.0(17)	3706.2(8)	5271.9(14)	31.5(4)
C15	7399.3(16)	3149.6(7)	3702.0(12)	24.7(3)
C16	7159.3(17)	2705.1(8)	4510.8(13)	28.3(4)
C17	6876.7(19)	2063.9(8)	4125.1(15)	34.8(4)
C18	7954(2)	1833.1(8)	3589.3(15)	39.0(5)
C19	8199(2)	2264.6(9)	2786.1(15)	40.1(5)
C20	8470.5(19)	2912.6(8)	3145.4(13)	31.9(4)
C21	6607.9(14)	4126.0(7)	1872.2(10)	16.6(3)
C22	7707.1(14)	4138.3(7)	1383.3(11)	19.1(3)
C23	7879.6(15)	3682.7(8)	723.2(12)	24.1(3)
C24	7009.5(16)	3215.7(7)	532.7(12)	24.1(3)
C25	5916.8(15)	3224.3(7)	1003.0(11)	22.4(3)
C26	5683.6(14)	3673.3(7)	1656.5(11)	19.6(3)
C27	8679.7(15)	4645.2(7)	1525.2(12)	23.5(3)
C28	10045.0(16)	4406.2(9)	1607.0(14)	30.5(4)
C29	8479.1(18)	5108.7(8)	714.8(14)	32.7(4)
C30	7220.9(18)	2719.1(8)	-177.6(14)	34.5(4)
C31	8509(2)	2413.1(10)	18.1(16)	45.4(5)
C32	7042(3)	2956.1(13)	-1181.6(17)	77.6(10)
C33	4414.2(15)	3687.1(9)	2080.6(13)	28.0(4)
C34	3960(2)	3064.9(12)	2381.7(18)	55.2(7)
C35	3395.9(19)	3956.6(11)	1391(2)	58.2(7)

Supplementary Table 16. Anisotropic Displacement Parameters (Å^2×10^3) for 7 The Anisotropic displacement factor exponent takes the form: -2π^2[h^{2}a*^{2}U$_{11}$+2hka*b*U$_{12}$+…].

Atom	U$_{11}$	U$_{22}$	U$_{33}$	U$_{23}$	U$_{13}$	U$_{12}$
P1	20.0(2)	22.60(19)	17.6(2)	0.50(15)	-0.34(15)	3.96(15)
F1	22.1(5)	27.9(5)	23.8(5)	1.1(4)	3.3(4)	2.4(4)
F2	22.7(5)	31.6(5)	24.5(5)	3.9(4)	4.6(4)	6.6(4)
O1	46.3(8)	31.2(6)	16.8(6)	-7.1(5)	-4.2(5)	14.4(6)
O2	31.6(6)	25.5(6)	16.0(6)	0.7(4)	-3.4(5)	10.5(5)

C1	37.2(10)	34.6(9)	21.4(9)	-11.4(7)	-2.7(7)	6.5(8)
C2	23.5(8)	24.8(7)	15.6(7)	-3.0(6)	0.7(6)	3.1(6)
C3	18.3(7)	21.0(7)	18.4(8)	-1.0(6)	1.7(6)	3.6(5)
C4	15.3(7)	20.2(7)	17.6(7)	-0.5(5)	3.1(5)	1.0(5)
C5	20.1(7)	21.2(7)	16.7(7)	0.0(6)	0.9(6)	0.8(5)
C6	24.5(8)	20.2(7)	25.0(9)	-0.6(6)	0.6(6)	4.4(6)
C7	26.4(8)	21.5(7)	21.6(8)	-4.7(6)	1.7(6)	3.7(6)
C8	33.7(9)	25.3(8)	23.1(9)	2.0(6)	-5.4(7)	7.6(7)
C9	24.7(8)	26.7(8)	21.2(8)	0.7(6)	-3.5(7)	0.5(6)
C10	36.6(10)	35.9(10)	21.2(9)	1.3(7)	-3.1(8)	0.1(8)
C11	62.3(14)	39.1(11)	22.5(10)	0.1(8)	-9.5(9)	-1.4(10)
C12	54.9(14)	41.7(12)	35.7(12)	4.4(9)	-25.2(10)	-3.8(10)
C13	31.2(10)	42.9(11)	47.1(13)	1.7(9)	-17.8(9)	-2.1(8)
C14	26.9(9)	31.0(9)	34.5(10)	-0.1(8)	-9.1(8)	2.6(7)
C15	27.8(8)	25.0(8)	20.2(8)	-0.2(6)	-2.9(7)	5.6(6)
C16	31.1(9)	24.3(8)	28.5(9)	1.9(7)	-2.3(7)	-0.2(7)
C17	38.7(10)	23.8(8)	39.8(11)	-0.4(8)	-7.4(9)	0.4(7)
C18	47.1(12)	23.3(9)	44.6(12)	-0.9(8)	-6.9(10)	6.9(8)
C19	55.8(13)	30.1(10)	33.7(11)	-5.3(8)	-0.4(9)	16.3(9)
C20	42.2(11)	26.1(8)	27.9(10)	0.8(7)	6.3(8)	12.6(7)
C21	17.6(7)	19.1(6)	13.1(7)	0.0(5)	1.0(5)	3.3(5)
C22	19.2(7)	20.6(7)	17.4(8)	0.0(6)	2.0(6)	1.0(5)
C23	22.9(8)	26.9(8)	23.6(9)	-4.9(6)	7.9(6)	1.7(6)
C24	26.4(8)	23.2(8)	23.2(9)	-6.3(6)	4.9(7)	2.1(6)
C25	24.8(8)	21.3(7)	20.6(8)	-1.8(6)	0.0(6)	-1.3(6)
C26	19.7(7)	23.3(7)	15.5(7)	1.2(6)	0.3(6)	0.1(6)
C27	21.6(8)	25.3(8)	24.4(8)	-2.8(6)	6.2(6)	-4.3(6)
C28	21.9(8)	36.9(10)	33.2(10)	-4.2(8)	5.2(7)	-4.6(7)
C29	34.1(10)	29.0(9)	36.0(11)	4.4(8)	8.1(8)	-6.5(7)
C30	35.6(10)	30.1(9)	39.2(11)	-16.2(8)	11.2(8)	-0.4(7)
C31	58.2(14)	36.3(11)	41.5(12)	-10.9(9)	3.2(10)	20.4(10)
C32	118(3)	76.9(19)	34.1(13)	-26.4(13)	-13.6(15)	60.1(18)
C33	18.3(8)	41.6(10)	24.4(9)	-12.4(7)	4.4(6)	-6.3(7)
C34	30.1(11)	76.1(17)	60.4(16)	38.2(13)	9.1(10)	-5.0(11)
C35	19.3(10)	56.0(14)	100(2)	31.2(14)	8.6(11)	6.3(9)

Supplementary Table 17. Bond Lengths for 7

Atom	Atom	Length/Å	Atom	Atom	Length/Å
P1	F1	1.6894(10)	C15	C16	1.547(2)

P1	F2	1.6752(10)	C15	C20	1.533(2)
P1	C3	1.8526(15)	C16	C17	1.536(2)
P1	C9	1.8469(17)	C17	C18	1.517(3)
P1	C15	1.8571(17)	C18	C19	1.524(3)
O1	C1	1.423(2)	C19	C20	1.535(3)
O1	C2	1.3723(19)	C21	C22	1.410(2)
O2	C5	1.3788(18)	C21	C26	1.412(2)
O2	C8	1.4284(18)	C22	C23	1.396(2)
C2	C3	1.417(2)	C22	C27	1.522(2)
C2	C7	1.383(2)	C23	C24	1.392(2)
C3	C4	1.405(2)	C24	C25	1.389(2)
C4	C5	1.410(2)	C24	C30	1.518(2)
C4	C21	1.506(2)	C25	C26	1.394(2)
C5	C6	1.383(2)	C26	C33	1.524(2)
C6	C7	1.390(2)	C27	C28	1.535(2)
C9	C10	1.540(3)	C27	C29	1.538(2)
C9	C14	1.540(2)	C30	C31	1.525(3)
C10	C11	1.528(3)	C30	C32	1.512(3)
C11	C12	1.523(3)	C33	C34	1.528(3)
C12	C13	1.523(3)	C33	C35	1.510(3)
C13	C14	1.531(3)			

Supplementary Table 18. Bond Angles for 7

Atom	Atom	Atom	Angle/°	Atom	Atom	Atom	Angle/°
F1	P1	C3	86.13(6)	C13	C14	C9	110.38(16)
F1	P1	C9	94.60(7)	C16	C15	P1	111.70(12)
F1	P1	C15	88.60(7)	C20	C15	P1	116.11(13)
F2	P1	F1	173.81(5)	C20	C15	C16	110.23(14)
F2	P1	C3	87.84(6)	C17	C16	C15	111.07(15)
F2	P1	C9	89.59(7)	C18	C17	C16	110.86(16)
F2	P1	C15	93.64(7)	C17	C18	C19	110.64(16)
C3	P1	C15	124.08(7)	C18	C19	C20	111.89(16)
C9	P1	C3	119.75(7)	C15	C20	C19	111.29(17)
C9	P1	C15	116.16(8)	C22	C21	C4	120.22(13)
C2	O1	C1	118.48(13)	C22	C21	C26	119.69(14)
C5	O2	C8	117.34(12)	C26	C21	C4	119.89(13)
O1	C2	C3	115.65(13)	C21	C22	C27	121.99(14)
O1	C2	C7	123.14(14)	C23	C22	C21	118.74(14)
C7	C2	C3	121.20(14)	C23	C22	C27	119.23(14)

C2	C3	P1	117.58(11)	C24	C23	C22	122.47(15)
C4	C3	P1	123.64(11)	C23	C24	C30	121.78(15)
C4	C3	C2	118.76(14)	C25	C24	C23	117.62(14)
C3	C4	C5	118.78(14)	C25	C24	C30	120.59(15)
C3	C4	C21	124.36(13)	C24	C25	C26	122.39(15)
C5	C4	C21	116.81(13)	C21	C26	C33	121.37(14)
O2	C5	C4	115.10(13)	C25	C26	C21	118.96(14)
O2	C5	C6	123.39(14)	C25	C26	C33	119.58(14)
C6	C5	C4	121.51(14)	C22	C27	C28	112.35(14)
C5	C6	C7	119.78(14)	C22	C27	C29	110.14(14)
C2	C7	C6	119.94(15)	C28	C27	C29	110.45(14)
C10	C9	P1	116.20(12)	C24	C30	C31	112.51(16)
C14	C9	P1	113.67(12)	C32	C30	C24	111.20(17)
C14	C9	C10	109.82(15)	C32	C30	C31	110.90(18)
C11	C10	C9	110.10(16)	C26	C33	C34	113.89(16)
C12	C11	C10	111.76(17)	C35	C33	C26	110.89(16)
C13	C12	C11	110.68(17)	C35	C33	C34	108.03(16)
C12	C13	C14	111.91(17)				

Supplementary Table 19. Torsion Angles for 7

A	B	C	D	Angle/°	A	B	C	D	Angle/°
P1	C3	C4	C5	176.92(11)	C9	P1	C3	C2	-28.42(16)
P1	C3	C4	C21	-0.8(2)	C9	P1	C3	C4	153.18(13)
P1	C9	C10	C11	-171.24(13)	C9	P1	C15	C16	46.25(15)
P1	C9	C14	C13	170.41(14)	C9	P1	C15	C20	-81.30(14)
P1	C15	C16	C17	173.53(12)	C9	C10	C11	C12	-57.5(2)
P1	C15	C20	C19	-177.23(12)	C10	C9	C14	C13	-57.5(2)
F1	P1	C3	C2	64.67(13)	C10	C11	C12	C13	55.5(2)
F1	P1	C3	C4	-113.73(14)	C11	C12	C13	C14	-54.9(2)
F1	P1	C9	C10	5.76(14)	C12	C13	C14	C9	56.4(2)
F1	P1	C9	C14	134.69(13)	C14	C9	C10	C11	58.0(2)
F1	P1	C15	C16	-48.19(12)	C15	P1	C3	C2	150.35(12)
F1	P1	C15	C20	-175.74(13)	C15	P1	C3	C4	-28.05(17)
F2	P1	C3	C2	-116.71(13)	C15	P1	C9	C10	-84.94(15)
F2	P1	C3	C4	64.89(13)	C15	P1	C9	C14	43.99(16)
F2	P1	C9	C10	-178.80(13)	C15	C16	C17	C18	57.4(2)
F2	P1	C9	C14	-49.87(13)	C16	C15	C20	C19	54.49(19)
F2	P1	C15	C16	137.58(12)	C16	C17	C18	C19	-57.1(2)
F2	P1	C15	C20	10.03(13)	C17	C18	C19	C20	56.2(2)

O1	C2	C3	P1	3.6(2)	C18	C19	C20	C15	-55.3(2)
O1	C2	C3	C4	-177.91(14)	C20	C15	C16	C17	-55.81(19)
O1	C2	C7	C6	179.15(15)	C21	C4	C5	O2	-1.7(2)
O2	C5	C6	C7	-179.15(15)	C21	C4	C5	C6	177.95(14)
C1	O1	C2	C3	172.99(15)	C21	C22	C23	C24	-0.3(2)
C1	O1	C2	C7	-6.5(2)	C21	C22	C27	C28	136.73(16)
C2	C3	C4	C5	-1.5(2)	C21	C22	C27	C29	-99.69(18)
C2	C3	C4	C21	-179.15(14)	C21	C26	C33	C34	-139.51(17)
C3	P1	C9	C10	93.93(14)	C21	C26	C33	C35	98.4(2)
C3	P1	C9	C14	-137.15(13)	C22	C21	C26	C25	3.9(2)
C3	P1	C15	C16	-132.56(12)	C22	C21	C26	C33	-172.63(15)
C3	P1	C15	C20	99.89(14)	C22	C23	C24	C25	2.1(3)
C3	C2	C7	C6	-0.3(3)	C22	C23	C24	C30	-179.15(17)
C3	C4	C5	O2	-179.58(13)	C23	C22	C27	C28	-45.8(2)
C3	C4	C5	C6	0.1(2)	C23	C22	C27	C29	77.82(19)
C3	C4	C21	C22	-92.77(19)	C23	C24	C25	C26	-1.0(2)
C3	C4	C21	C26	92.37(19)	C23	C24	C30	C31	52.7(2)
C4	C5	C6	C7	1.2(2)	C23	C24	C30	C32	-72.4(3)
C4	C21	C22	C23	-177.69(14)	C24	C25	C26	C21	-2.0(2)
C4	C21	C22	C27	-0.2(2)	C24	C25	C26	C33	174.58(16)
C4	C21	C26	C25	178.82(14)	C25	C24	C30	C31	-128.59(19)
C4	C21	C26	C33	2.3(2)	C25	C24	C30	C32	106.3(2)
C5	C4	C21	C22	89.49(18)	C25	C26	C33	C34	43.9(2)
C5	C4	C21	C26	-85.37(18)	C25	C26	C33	C35	-78.1(2)
C5	C6	C7	C2	-1.1(2)	C26	C21	C22	C23	-2.8(2)
C7	C2	C3	P1	-176.87(13)	C26	C21	C22	C27	174.70(14)
C7	C2	C3	C4	1.6(2)	C27	C22	C23	C24	-177.85(15)
C8	O2	C5	C4	169.78(14)	C30	C24	C25	C26	-179.69(16)
C8	O2	C5	C6	-9.9(2)					

Supplementary Table 20. Hydrogen Atom Coordinates (Å×10^4) and Isotropic Displacement Parameters (Å^2×10^3) for 7.

Atom	*x*	*y*	*z*	U(eq)
H1A	7148.28	5910.4	5405.2	47
H1B	7082.85	5468.13	6294.32	47
H1C	5811.2	5652.6	5662.98	47
H6	4904.04	5941.15	2508.51	28
H7	5565.71	5915.55	4130.21	28
H8A	4273.29	5491.36	168.26	42

H8B	4879.56	5971.25	926.48	42
H8C	3714.76	5557.31	1174.19	42
H9	8846.26	4498.21	5232.83	29
H10A	7187.32	4203.98	6150.77	38
H10B	7774.04	3534.17	6243.03	38
H11A	9042.61	4615.34	6956.71	51
H11B	8490.22	4102.41	7604.09	51
H12A	10728.51	4002.51	7577.45	55
H12B	10009.56	3403.51	7176.95	55
H13A	11535.36	3663.16	6149.65	50
H13B	10985.37	4338.12	6039.33	50
H14A	9682.91	3266.6	5320.55	38
H14B	10261.32	3777.01	4680.21	38
H15	6615.42	3140.17	3252.02	30
H16A	7914.7	2693.37	4976.44	34
H16B	6433.55	2850.79	4840.72	34
H17A	6752.79	1786.24	4657.51	42
H17B	6083.98	2069.46	3697.33	42
H18A	7739.04	1426.24	3326.68	47
H18B	8731.75	1795.33	4029.27	47
H19A	7450	2268.83	2314.23	48
H19B	8931.29	2115.85	2466.51	48
H20A	9276.42	2917.75	3558.12	38
H20B	8567.23	3183.34	2599.72	38
H23	8618.42	3691.72	391.73	29
H25	5306.11	2912.7	874.65	27
H27	8542.06	4858.06	2130.03	28
H28A	10157.39	4111.26	2125.07	46
H28B	10634.71	4744.58	1736.79	46
H28C	10214.73	4209.97	1011.48	46
H29A	8601.38	4909.59	111.99	49
H29B	9091.31	5440.02	824.29	49
H29C	7616.93	5271.92	691.72	49
H30	6559.45	2402	-114.71	41
H31A	9180.65	2705.41	-79.94	68
H31B	8564.72	2069.38	-414.06	68
H31C	8608.19	2268.06	674.17	68
H32A	6216.48	3156.87	-1287.66	116
H32B	7078.87	2617.64	-1626.24	116
H32C	7715.16	3247.33	-1281.77	116

H33	4507.55	3952.4	2654.83	34
H34A	3782.53	2808.43	1820.59	83
H34B	3186.18	3112.21	2703.51	83
H34C	4619.04	2875.05	2814.04	83
H35A	3649.2	4364.92	1211.06	87
H35B	2599.11	3978.96	1688.73	87
H35C	3278.72	3700.73	824.02	87

X-ray Crystal Structure Data for (5-(diphenylphosphaneyl)-9,9-dimethyl-9H-xanthen-4-yl)difluorodiphenyl-λ^5-phosphane (L5) (CCDC: 2243163)

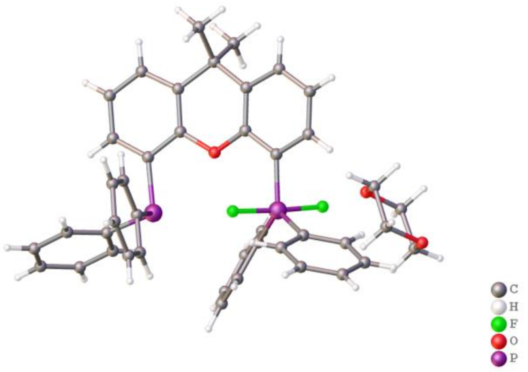

Supplementary Table 21. Crystal data and structure refinement for L5.

Identification code	**L5**
CCDC	2243163
Empirical formula	$C_{43}H_{40}F_2O_3P_2$
Formula weight	704.69
Temperature/K	113.15
Crystal system	triclinic
Space group	P-1
a/Å	10.4105(2)
b/Å	11.0943(2)
c/Å	17.6362(4)
α/°	84.572(2)
β/°	82.783(2)
γ/°	62.129(2)
Volume/Å^3	1784.89(7)
Z	2
ρ_{calc}g/cm^3	1.311
μ/mm^{-1}	0.173
F(000)	740.0

Crystal size/mm^3	0.26 × 0.24 × 0.22
Radiation	MoKα (λ = 0.71073)
2Θ range for data collection/°	4.156 to 57.398
Index ranges	-14 ≤ h ≤ 14, -14 ≤ k ≤ 14, -23 ≤ l ≤ 23
Reflections collected	30588
Independent reflections	8987 [R_{int} = 0.0407, R_{sigma} = 0.0416]
Data/restraints/parameters	8987/0/453
Goodness-of-fit on F^2	1.022
Final R indexes [I>=2σ (I)]	R_1 = 0.0496, wR_2 = 0.1170
Final R indexes [all data]	R_1 = 0.0658, wR_2 = 0.1261
Largest diff. peak/hole / e Å^{-3}	0.47/-0.39

Supplementary Table 22. Fractional Atomic Coordinates (×10^4) and Equivalent Isotropic Displacement Parameters (Å^2×10^3) for L5. U_{eq} is defined as 1/3 of of the trace of the orthogonalised U_{IJ} tensor.

Atom	*x*	*y*	*z*	U(eq)
P1	3536.0(5)	3658.8(4)	4255.3(2)	19.84(10)
P2	2353.5(5)	5355.8(4)	1906.8(2)	20.63(10)
F1	2119.8(10)	4107.0(9)	2376.3(5)	23.1(2)
F2	2713.7(11)	6582.9(10)	1440.1(6)	27.0(2)
O1	4982.5(13)	3348.8(12)	2735.7(6)	24.7(3)
C1	5977.5(17)	2405.4(17)	3212.9(9)	20.3(3)
C2	5465.3(17)	2471.3(16)	3991.1(9)	19.4(3)
C3	6421.0(18)	1557.7(17)	4501.3(10)	22.7(3)
C4	7815.4(19)	598.1(18)	4245.6(10)	25.4(4)
C5	8269.9(18)	534.0(18)	3471.6(10)	24.7(4)
C6	7365.5(17)	1449.0(17)	2935.9(9)	21.3(3)
C7	7917.8(18)	1424.4(19)	2092.7(10)	26.1(4)
C8	6651.2(18)	2331.3(17)	1619.7(10)	23.5(3)
C9	5291.4(18)	3237.6(17)	1954.7(9)	21.7(3)
C10	4154.4(18)	4123.4(17)	1513.9(9)	22.0(3)
C11	4397(2)	4070.5(18)	721.8(10)	27.0(4)
C12	5735(2)	3164.6(19)	375.3(10)	31.0(4)
C13	6842(2)	2314.2(19)	822.1(10)	28.5(4)
C14	8657(3)	-45(2)	1821.0(12)	45.8(6)
C15	9028(2)	1995(3)	1999.7(12)	43.8(5)
C16	2725.0(18)	2579.5(17)	4049.1(9)	21.4(3)
C17	3539.1(19)	1242.7(18)	3811.3(10)	26.3(4)
C18	2859(2)	497(2)	3640.8(11)	32.5(4)

C19	1352(2)	1070(2)	3719.4(11)	35.3(5)
C20	529(2)	2388(2)	3947.3(11)	35.5(5)
C21	1204.1(19)	3154(2)	4106.3(10)	28.9(4)
C22	3448.1(17)	3492.7(17)	5306.8(9)	21.7(3)
C23	3353.6(19)	2420.3(19)	5749.3(10)	26.9(4)
C24	3261(2)	2404(2)	6541.3(11)	33.0(4)
C25	3250(2)	3462(2)	6904.0(11)	37.2(5)
C26	3337(2)	4536(2)	6475.1(11)	37.4(5)
C27	3429(2)	4556.4(19)	5681.3(11)	29.6(4)
C28	825.1(19)	5796.4(18)	1344.1(10)	26.2(4)
C29	448(2)	6852(2)	784.2(11)	36.4(4)
C30	-729(3)	7193(2)	364.2(13)	48.0(6)
C31	-1539(2)	6497(3)	492.6(12)	46.4(6)
C32	-1182(2)	5458(3)	1040.3(13)	45.8(5)
C33	-8(2)	5099(2)	1468.5(12)	35.6(4)
C34	1995.8(18)	6217.7(16)	2785.2(9)	20.9(3)
C35	754.2(18)	6431.5(17)	3272.6(10)	24.0(3)
C36	484(2)	7086.6(18)	3952.7(10)	28.5(4)
C37	1462(2)	7483.8(18)	4161.6(10)	30.1(4)
C38	2700(2)	7261.8(18)	3681.6(11)	30.1(4)
C39	2960(2)	6653.2(17)	2990.4(10)	25.8(4)
O2	2560.4(17)	10697.3(15)	995.3(9)	43.9(4)
O3	4998.9(18)	9065.2(18)	1810.5(10)	53.5(4)
C40	2907(2)	9337(2)	1243.0(15)	42.8(5)
C41	4522(2)	8501(2)	1289.6(14)	42.8(5)
C42	4641(3)	10428(3)	1591.4(19)	65.1(8)
C43	3040(3)	11275(2)	1513.5(15)	51.3(6)

Supplementary Table 23. Anisotropic Displacement Parameters (Å^2×10^3) for L5. The Anisotropic displacement factor exponent takes the form: -2π^2[$h^2a^{*2}U_{11}$+2hka*b*U_{12}+…].

Atom	U_{11}	U_{22}	U_{33}	U_{23}	U_{13}	U_{12}
P1	19.6(2)	17.0(2)	19.9(2)	-0.79(15)	-2.36(16)	-5.80(17)
P2	23.0(2)	18.7(2)	18.4(2)	0.54(16)	-3.34(16)	-8.01(17)
F1	24.9(5)	20.2(5)	24.2(5)	-1.0(4)	-2.8(4)	-10.2(4)
F2	34.0(6)	22.2(5)	23.8(5)	2.5(4)	-2.1(4)	-12.8(4)
O1	21.8(6)	24.9(6)	18.1(6)	-1.3(5)	-1.6(5)	-3.0(5)
C1	19.4(7)	18.7(8)	23.2(8)	-0.4(6)	-4.0(6)	-8.7(6)
C2	17.4(7)	18.5(8)	23.1(8)	-1.4(6)	-2.2(6)	-8.8(6)

C3	23.8(8)	23.8(9)	21.8(8)	0.5(6)	-4.7(6)	-11.8(7)
C4	22.7(8)	23.1(9)	29.0(9)	2.6(7)	-8.6(7)	-8.7(7)
C5	17.5(7)	21.7(9)	33.1(9)	-2.3(7)	-3.8(7)	-6.9(7)
C6	18.2(7)	21.3(8)	25.1(8)	-2.8(6)	0.0(6)	-10.0(7)
C7	18.9(8)	27.7(9)	25.0(8)	-3.1(7)	1.4(7)	-5.7(7)
C8	21.8(8)	21.6(9)	24.8(8)	-3.1(7)	1.7(6)	-8.7(7)
C9	23.9(8)	20.4(8)	20.2(8)	-1.7(6)	-1.5(6)	-9.7(7)
C10	22.6(8)	19.0(8)	22.7(8)	-2.6(6)	-1.5(6)	-8.0(7)
C11	31.3(9)	25.9(9)	20.4(8)	-0.2(7)	-3.5(7)	-10.2(8)
C12	39.5(10)	29.4(10)	19.2(8)	-3.5(7)	1.8(7)	-12.3(8)
C13	27.5(9)	26.8(10)	26.1(9)	-4.9(7)	4.9(7)	-9.3(8)
C14	46.6(13)	31.8(11)	33.1(11)	-9.0(9)	-4.1(9)	5.0(9)
C15	29.8(10)	71.3(16)	34.0(11)	6.3(10)	0.9(8)	-28.8(11)
C16	22.6(8)	23.1(9)	17.2(7)	2.6(6)	-4.0(6)	-9.6(7)
C17	25.1(8)	25.9(9)	28.8(9)	0.0(7)	-4.6(7)	-12.3(7)
C18	40.1(11)	31.6(10)	33.4(10)	-0.2(8)	-7.9(8)	-22.0(9)
C19	41.2(11)	47.1(12)	33.2(10)	6.8(9)	-12.3(9)	-32.7(10)
C20	24.6(9)	50.6(13)	34.2(10)	9.7(9)	-8.5(8)	-20.3(9)
C21	23.3(8)	32.4(10)	26.7(9)	3.1(7)	-3.7(7)	-9.6(8)
C22	18.8(7)	22.1(9)	19.7(8)	-2.7(6)	-1.6(6)	-5.6(6)
C23	24.6(8)	26.5(9)	26.3(9)	-0.2(7)	-2.4(7)	-9.2(7)
C24	26.5(9)	40.9(11)	26.2(9)	7.9(8)	-3.4(7)	-12.5(8)
C25	28.0(9)	53.0(13)	19.9(9)	-4.8(8)	-2.5(7)	-9.0(9)
C26	38.4(11)	37.8(11)	30.7(10)	-13.5(8)	-6.5(8)	-10.2(9)
C27	31.0(9)	25.3(9)	29.7(9)	-3.1(7)	-5.1(8)	-9.7(8)
C28	22.9(8)	28.0(9)	20.3(8)	-5.7(7)	-3.2(7)	-4.4(7)
C29	37.7(11)	31.0(11)	33.2(10)	0.7(8)	-11.0(9)	-8.3(9)
C30	47.6(13)	42.9(13)	34.0(11)	3.9(9)	-18.9(10)	-2.1(10)
C31	27.7(10)	59.2(15)	37.0(11)	-15.1(10)	-13.0(9)	-2.4(10)
C32	29.2(10)	62.5(15)	46.2(12)	-9.0(11)	-8.4(9)	-19.2(10)
C33	29.1(10)	42.6(12)	33.8(10)	0.0(9)	-6.5(8)	-14.9(9)
C34	23.3(8)	15.4(8)	19.1(7)	1.3(6)	-3.8(6)	-4.9(6)
C35	22.1(8)	21.1(9)	24.5(8)	-0.4(7)	-4.6(7)	-6.0(7)
C36	24.4(8)	24.1(9)	25.4(9)	-2.8(7)	-1.7(7)	-1.3(7)
C37	37.2(10)	18.2(9)	26.6(9)	-4.1(7)	-7.8(8)	-4.2(8)
C38	38.9(10)	21.6(9)	31.9(9)	-0.2(7)	-10.4(8)	-14.3(8)
C39	28.6(9)	21.4(9)	27.1(9)	2.7(7)	-4.5(7)	-11.5(7)
O2	50.3(9)	31.0(8)	42.8(8)	2.2(6)	-18.9(7)	-9.6(7)
O3	46.2(9)	56.8(11)	59.9(10)	11.8(8)	-27.1(8)	-22.9(8)
C40	35.9(11)	29.5(11)	63.0(15)	-5.3(10)	-13.3(10)	-12.3(9)

C41	34.1(11)	33.7(12)	55.0(14)	0.9(10)	-8.3(10)	-10.5(9)
C42	68.6(18)	69.0(19)	84(2)	2.3(15)	-21.9(15)	-51.1(16)
C43	68.9(17)	34.7(13)	58.5(15)	-2.7(11)	-14.3(13)	-28.4(12)

Supplementary Table 24. Bond Lengths for L5.

Atom	Atom	Length/Å	Atom	Atom	Length/Å
P1	C2	1.8422(16)	C17	C18	1.388(2)
P1	C16	1.8395(17)	C18	C19	1.384(3)
P1	C22	1.8411(17)	C19	C20	1.374(3)
P2	F1	1.6469(10)	C20	C21	1.396(3)
P2	F2	1.6908(11)	C22	C23	1.396(2)
P2	C10	1.8222(17)	C22	C27	1.397(2)
P2	C28	1.8258(17)	C23	C24	1.387(3)
P2	C34	1.8043(16)	C24	C25	1.384(3)
O1	C1	1.3839(19)	C25	C26	1.383(3)
O1	C9	1.3783(19)	C26	C27	1.390(3)
C1	C2	1.404(2)	C28	C29	1.396(3)
C1	C6	1.393(2)	C28	C33	1.395(3)
C2	C3	1.392(2)	C29	C30	1.391(3)
C3	C4	1.388(2)	C30	C31	1.373(4)
C4	C5	1.385(2)	C31	C32	1.371(3)
C5	C6	1.399(2)	C32	C33	1.395(3)
C6	C7	1.524(2)	C34	C35	1.392(2)
C7	C8	1.524(2)	C34	C39	1.396(2)
C7	C14	1.537(3)	C35	C36	1.391(2)
C7	C15	1.543(3)	C36	C37	1.383(3)
C8	C9	1.391(2)	C37	C38	1.381(3)
C8	C13	1.396(2)	C38	C39	1.385(2)
C9	C10	1.404(2)	O2	C40	1.416(3)
C10	C11	1.390(2)	O2	C43	1.421(3)
C11	C12	1.385(3)	O3	C41	1.413(3)
C12	C13	1.382(3)	O3	C42	1.405(3)
C16	C17	1.395(2)	C40	C41	1.502(3)
C16	C21	1.397(2)	C42	C43	1.500(4)

Supplementary Table 25. Bond Angles for L5.

Atom	Atom	Atom	Angle/°	Atom	Atom	Atom	Angle/°

C16	P1	C2	98.44(7)	C12	C11	C10	120.61(17)
C16	P1	C22	101.04(7)	C13	C12	C11	119.60(16)
C22	P1	C2	101.41(7)	C12	C13	C8	121.89(17)
F1	P2	F2	176.06(6)	C17	C16	P1	123.81(13)
F1	P2	C10	89.89(7)	C17	C16	C21	118.30(16)
F1	P2	C28	91.98(7)	C21	C16	P1	117.84(14)
F1	P2	C34	90.64(6)	C18	C17	C16	120.99(17)
F2	P2	C10	87.10(7)	C19	C18	C17	119.92(19)
F2	P2	C28	91.66(7)	C20	C19	C18	120.05(17)
F2	P2	C34	88.98(7)	C19	C20	C21	120.37(18)
C10	P2	C28	117.13(8)	C20	C21	C16	120.34(18)
C34	P2	C10	124.64(8)	C23	C22	P1	125.18(13)
C34	P2	C28	118.17(8)	C23	C22	C27	118.39(16)
C9	O1	C1	119.27(13)	C27	C22	P1	116.40(13)
O1	C1	C2	114.78(14)	C24	C23	C22	120.75(17)
O1	C1	C6	122.25(14)	C25	C24	C23	120.19(18)
C6	C1	C2	122.95(15)	C26	C25	C24	119.88(17)
C1	C2	P1	117.79(12)	C25	C26	C27	120.10(18)
C3	C2	P1	124.48(13)	C26	C27	C22	120.68(18)
C3	C2	C1	117.52(15)	C29	C28	P2	120.87(15)
C4	C3	C2	121.01(16)	C33	C28	P2	120.90(14)
C5	C4	C3	119.95(16)	C33	C28	C29	118.22(17)
C4	C5	C6	121.36(16)	C30	C29	C28	120.6(2)
C1	C6	C5	117.15(15)	C31	C30	C29	120.6(2)
C1	C6	C7	122.11(15)	C32	C31	C30	119.57(19)
C5	C6	C7	120.69(15)	C31	C32	C33	120.8(2)
C6	C7	C8	109.86(14)	C32	C33	C28	120.2(2)
C6	C7	C14	110.07(16)	C35	C34	P2	119.62(13)
C6	C7	C15	108.09(15)	C35	C34	C39	119.44(15)
C8	C7	C14	110.13(15)	C39	C34	P2	120.94(13)
C8	C7	C15	108.76(16)	C36	C35	C34	119.65(16)
C14	C7	C15	109.89(17)	C37	C36	C35	120.59(17)
C9	C8	C7	122.10(15)	C38	C37	C36	119.77(16)
C9	C8	C13	117.44(16)	C37	C38	C39	120.33(17)
C13	C8	C7	120.41(15)	C38	C39	C34	120.17(17)
O1	C9	C8	122.47(15)	C40	O2	C43	109.37(16)
O1	C9	C10	115.71(14)	C42	O3	C41	110.34(18)
C8	C9	C10	121.80(15)	O2	C40	C41	110.52(18)
C9	C10	P2	124.54(13)	O3	C41	C40	110.13(18)
C11	C10	P2	116.82(13)	O3	C42	C43	111.9(2)

C11	C10	C9	118.64(16)	O2	C43	C42	111.0(2)

Supplementary Table 26. Torsion Angles for L5.

A	B	C	D	Angle/°	A	B	C	D	Angle/°
P1	C2	C3	C4	173.23(13)	C9	C10	C11	C12	-0.2(3)
P1	C16	C17	C18	-177.88(14)	C10	P2	C28	C29	-87.89(16)
P1	C16	C21	C20	179.21(14)	C10	P2	C28	C33	92.91(16)
P1	C22	C23	C24	-178.59(14)	C10	P2	C34	C35	-140.89(13)
P1	C22	C27	C26	178.79(15)	C10	P2	C34	C39	37.98(17)
P2	C10	C11	C12	179.92(14)	C10	C11	C12	C13	-0.5(3)
P2	C28	C29	C30	-179.20(16)	C11	C12	C13	C8	0.4(3)
P2	C28	C33	C32	179.25(16)	C13	C8	C9	O1	-179.55(15)
P2	C34	C35	C36	179.51(13)	C13	C8	C9	C10	-1.1(3)
P2	C34	C39	C38	-177.29(13)	C14	C7	C8	C9	133.66(18)
F1	P2	C10	C9	-59.01(15)	C14	C7	C8	C13	-48.9(2)
F1	P2	C10	C11	120.89(14)	C15	C7	C8	C9	-105.87(19)
F1	P2	C28	C29	-178.78(15)	C15	C7	C8	C13	71.6(2)
F1	P2	C28	C33	2.02(15)	C16	P1	C2	C1	81.79(13)
F1	P2	C34	C35	-50.58(13)	C16	P1	C2	C3	-92.90(14)
F1	P2	C34	C39	128.29(14)	C16	P1	C22	C23	22.28(16)
F2	P2	C10	C9	118.45(15)	C16	P1	C22	C27	-155.59(14)
F2	P2	C10	C11	-61.66(14)	C16	C17	C18	C19	-1.2(3)
F2	P2	C28	C29	-0.30(15)	C17	C16	C21	C20	1.6(3)
F2	P2	C28	C33	-179.50(15)	C17	C18	C19	C20	1.7(3)
F2	P2	C34	C35	133.34(13)	C18	C19	C20	C21	-0.5(3)
F2	P2	C34	C39	-47.78(14)	C19	C20	C21	C16	-1.2(3)
O1	C1	C2	P1	5.59(19)	C21	C16	C17	C18	-0.4(3)
O1	C1	C2	C3	-179.34(14)	C22	P1	C2	C1	-175.06(12)
O1	C1	C6	C5	-179.12(14)	C22	P1	C2	C3	10.25(15)
O1	C1	C6	C7	3.5(2)	C22	P1	C16	C17	-100.12(15)
O1	C9	C10	P2	-0.6(2)	C22	P1	C16	C21	82.43(14)
O1	C9	C10	C11	179.56(15)	C22	C23	C24	C25	0.5(3)
C1	O1	C9	C8	-9.2(2)	C23	C22	C27	C26	0.8(3)
C1	O1	C9	C10	172.26(14)	C23	C24	C25	C26	-0.2(3)
C1	C2	C3	C4	-1.5(2)	C24	C25	C26	C27	0.2(3)
C1	C6	C7	C8	-13.0(2)	C25	C26	C27	C22	-0.5(3)
C1	C6	C7	C14	-134.40(18)	C27	C22	C23	C24	-0.8(3)
C1	C6	C7	C15	105.57(19)	C28	P2	C10	C9	-151.17(14)
C2	P1	C16	C17	3.34(16)	C28	P2	C10	C11	28.72(17)

C2	P1	C16	C21	-174.10(13)	C28	P2	C34	C35	42.00(16)
C2	P1	C22	C23	-78.79(15)	C28	P2	C34	C39	-139.12(14)
C2	P1	C22	C27	103.34(14)	C28	C29	C30	C31	0.0(3)
C2	C1	C6	C5	-0.6(2)	C29	C28	C33	C32	0.0(3)
C2	C1	C6	C7	-177.97(15)	C29	C30	C31	C32	0.0(3)
C2	C3	C4	C5	-0.5(3)	C30	C31	C32	C33	0.0(3)
C3	C4	C5	C6	2.0(3)	C31	C32	C33	C28	-0.1(3)
C4	C5	C6	C1	-1.5(2)	C33	C28	C29	C30	0.0(3)
C4	C5	C6	C7	175.98(16)	C34	P2	C10	C9	31.70(18)
C5	C6	C7	C8	169.74(15)	C34	P2	C10	C11	-148.41(13)
C5	C6	C7	C14	48.3(2)	C34	P2	C28	C29	89.44(16)
C5	C6	C7	C15	-71.7(2)	C34	P2	C28	C33	-89.76(17)
C6	C1	C2	P1	-173.05(12)	C34	C35	C36	C37	-2.3(3)
C6	C1	C2	C3	2.0(2)	C35	C34	C39	C38	1.6(2)
C6	C7	C8	C9	12.3(2)	C35	C36	C37	C38	1.8(3)
C6	C7	C8	C13	-170.26(16)	C36	C37	C38	C39	0.5(3)
C7	C8	C9	O1	-2.0(3)	C37	C38	C39	C34	-2.1(3)
C7	C8	C9	C10	176.41(15)	C39	C34	C35	C36	0.6(2)
C7	C8	C13	C12	-177.20(17)	O2	C40	C41	O3	59.8(3)
C8	C9	C10	P2	-179.06(13)	O3	C42	C43	O2	-55.6(3)
C8	C9	C10	C11	1.0(3)	C40	O2	C43	C42	56.4(3)
C9	O1	C1	C2	-170.17(14)	C41	O3	C42	C43	55.8(3)
C9	O1	C1	C6	8.5(2)	C42	O3	C41	C40	-57.4(3)
C9	C8	C13	C12	0.4(3)	C43	O2	C40	C41	-58.7(3)

Supplementary Table 27. Hydrogen Atom Coordinates (Å×10^4) and Isotropic Displacement Parameters (Å^2×10^3) for L5.

Atom	*x*	*y*	*z*	U(eq)
H3	6114.02	1591.54	5032.89	27
H4	8457.68	-14.39	4601.41	30
H5	9215.39	-145.22	3300.68	30
H11	3638.16	4661.41	415.21	32
H12	5891.7	3128.08	-166.23	37
H13	7758.6	1700.16	579.63	34
H14A	9474.47	-617.31	2125.11	69
H14B	9019.31	-52.28	1280.16	69
H14C	7947.6	-403.38	1884.35	69
H15A	8553.02	2928.05	2179.3	66
H15B	9387.92	2000.43	1459.19	66

H15C	9848.08	1419.17	2301.55	66
H17	4574.52	837.14	3765.53	32
H18	3427.25	-406.6	3470.25	39
H19	885.87	551.25	3615.71	42
H20	-506.07	2780.45	3996.89	43
H21	626.71	4070.27	4254.27	35
H23	3352.77	1693.19	5505.17	32
H24	3204.54	1664.85	6835.63	40
H25	3183.03	3450.68	7446.61	45
H26	3332.89	5261.66	6723.53	45
H27	3480.56	5301	5390.46	36
H29	999.12	7340.85	689.35	44
H30	-974.31	7913.48	-14.98	58
H31	-2341.76	6735.2	203.81	56
H32	-1742.29	4975.88	1128.99	55
H33	226.36	4376.79	1846.42	43
H35	95.39	6131.6	3141.36	29
H36	-381.35	7262.85	4276.56	34
H37	1283.76	7907.93	4633.64	36
H38	3376.99	7527.85	3826.31	36
H39	3796.69	6531.53	2654.87	31
H40A	2581.82	8929.05	880.7	51
H40B	2385.99	9331.9	1752.1	51
H41A	4749.5	7551.87	1459.1	51
H41B	5043.15	8481.65	777.4	51
H42A	5195.9	10455.63	1096.76	78
H42B	4932.94	10822.12	1977.82	78
H43A	2488.07	11336.66	2020.57	62
H43B	2841.64	12210.81	1327.3	62

^{1}H NMR, ^{13}C NMR, ^{19}F NMR and ^{31}P NMR Spectra

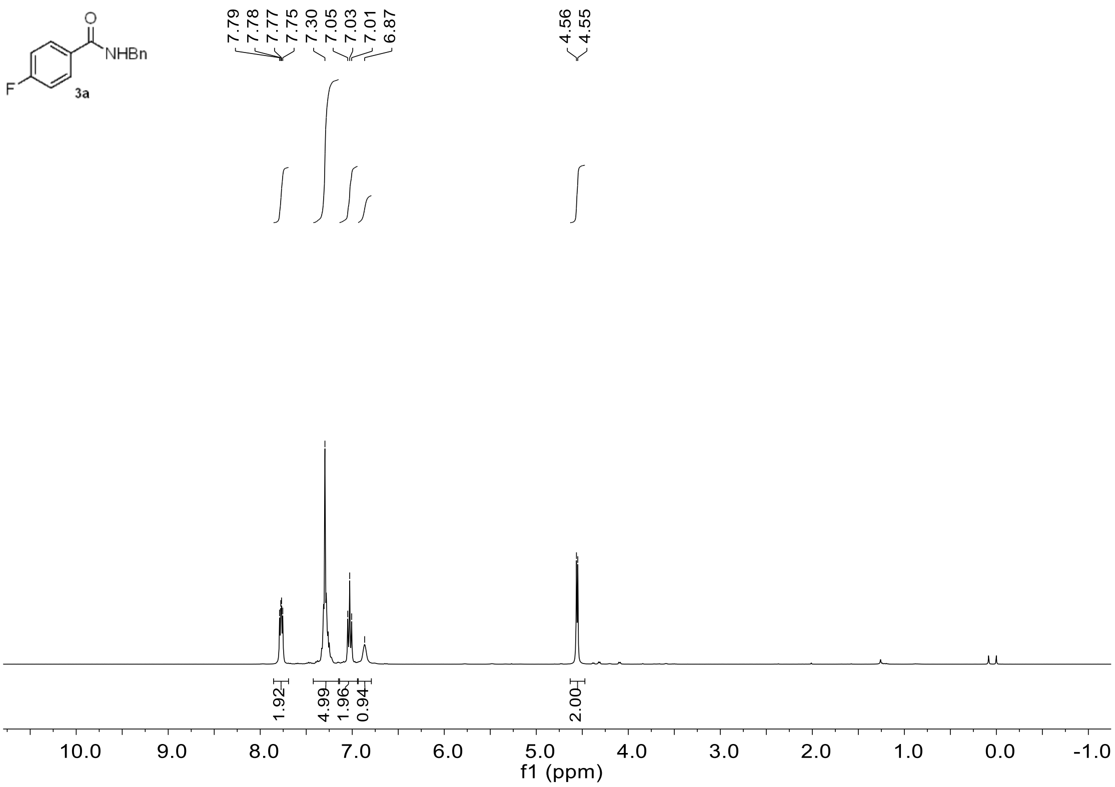

Supplementary Fig. 7. ^{1}H NMR spectrum (400 MHz, $CDCl_3$) of **3a**

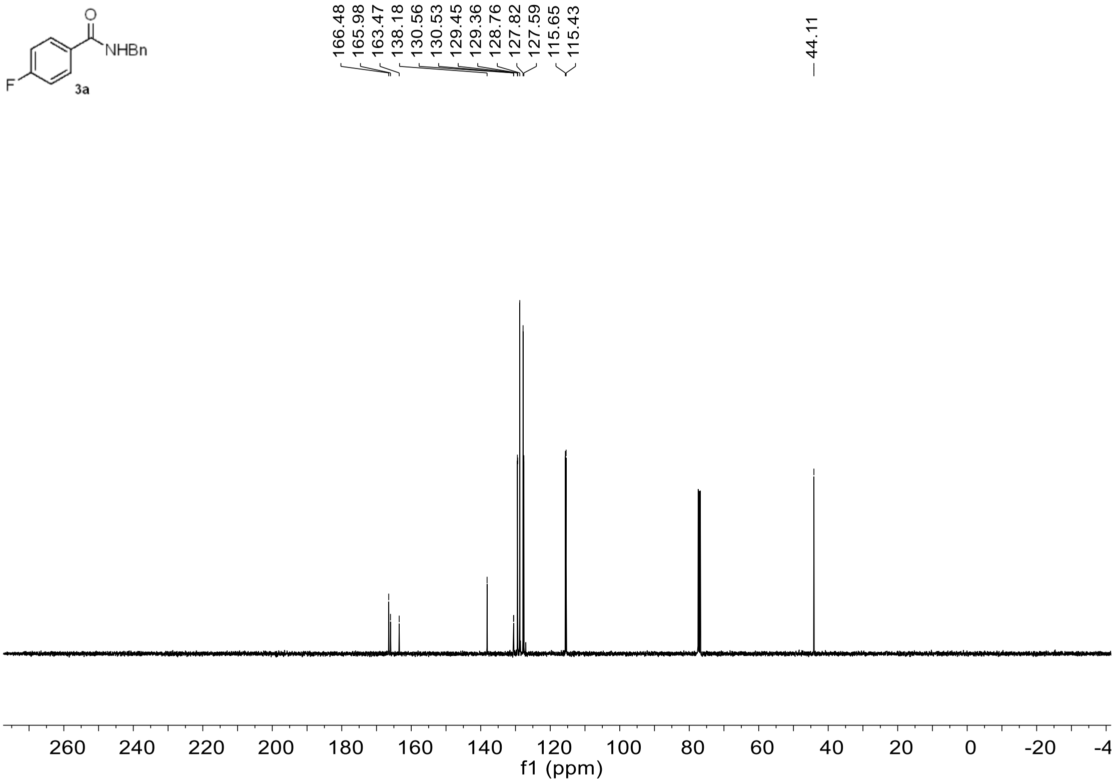

Supplementary Fig. 8. ^{13}C NMR spectrum (101 MHz, $CDCl_3$) of **3a**

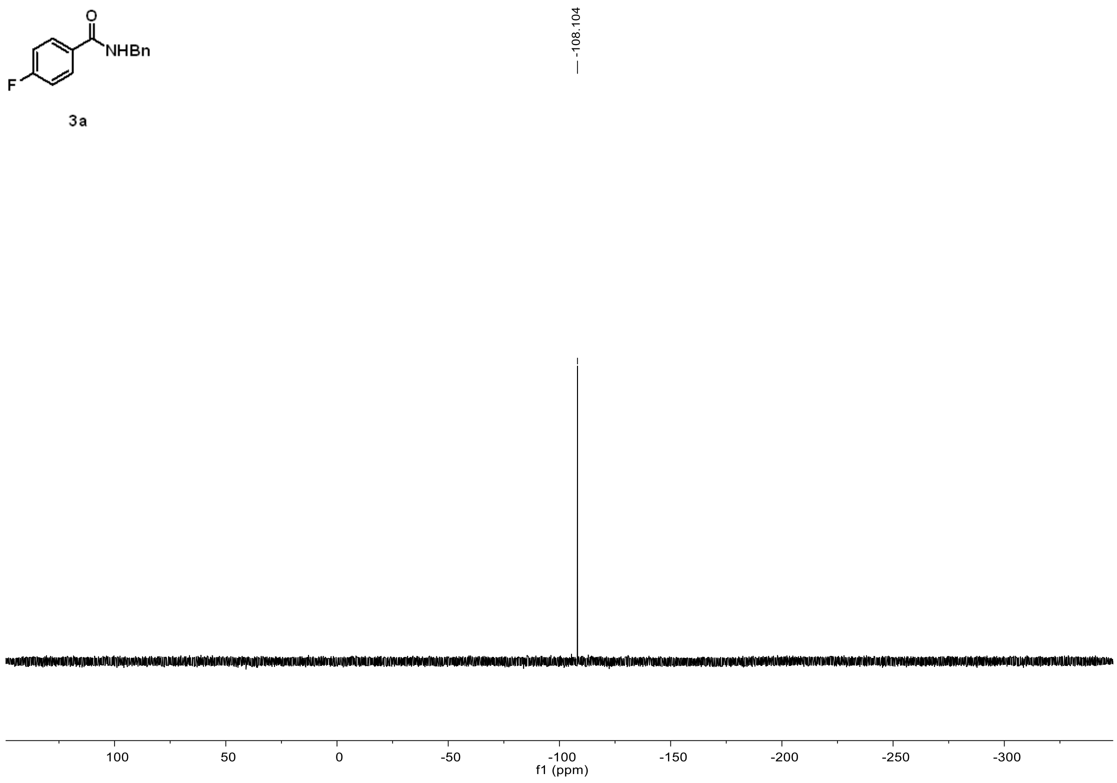

Supplementary Fig. 9. ^{19}F NMR spectrum (376 MHz, $CDCl_3$) of **3a**

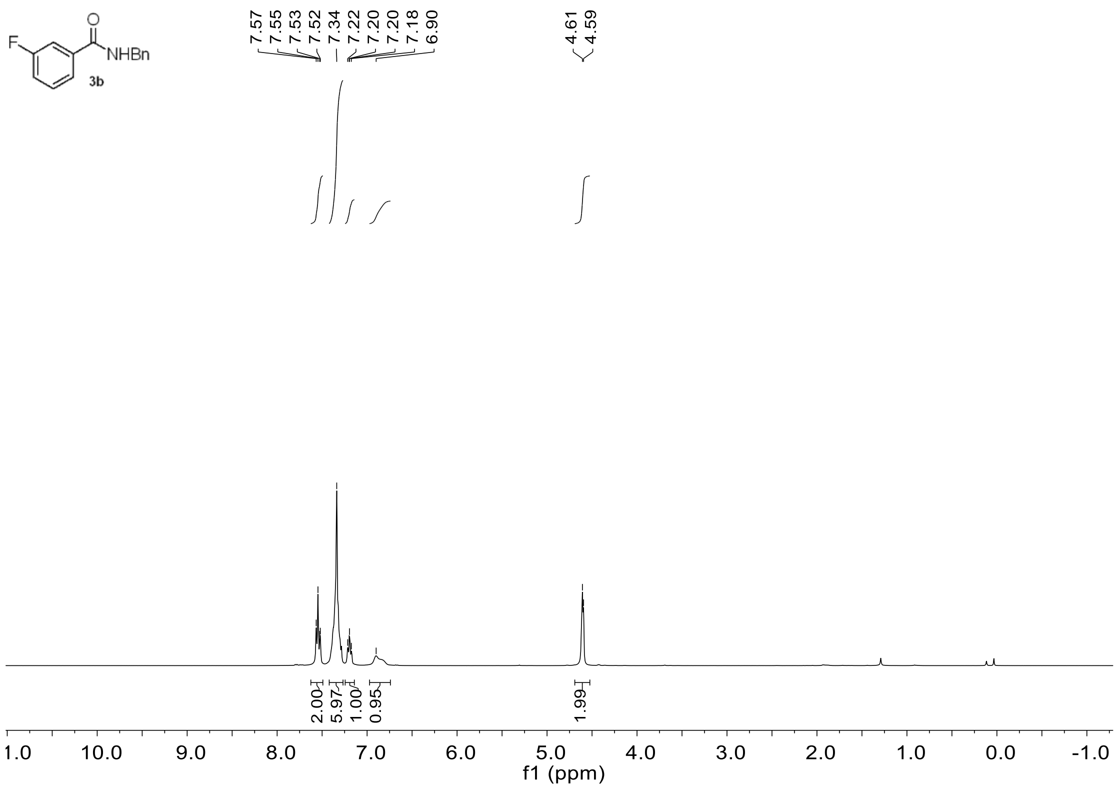

Supplementary Fig. 10. ^{1}H NMR spectrum (400 MHz, $CDCl_3$) of **3b**

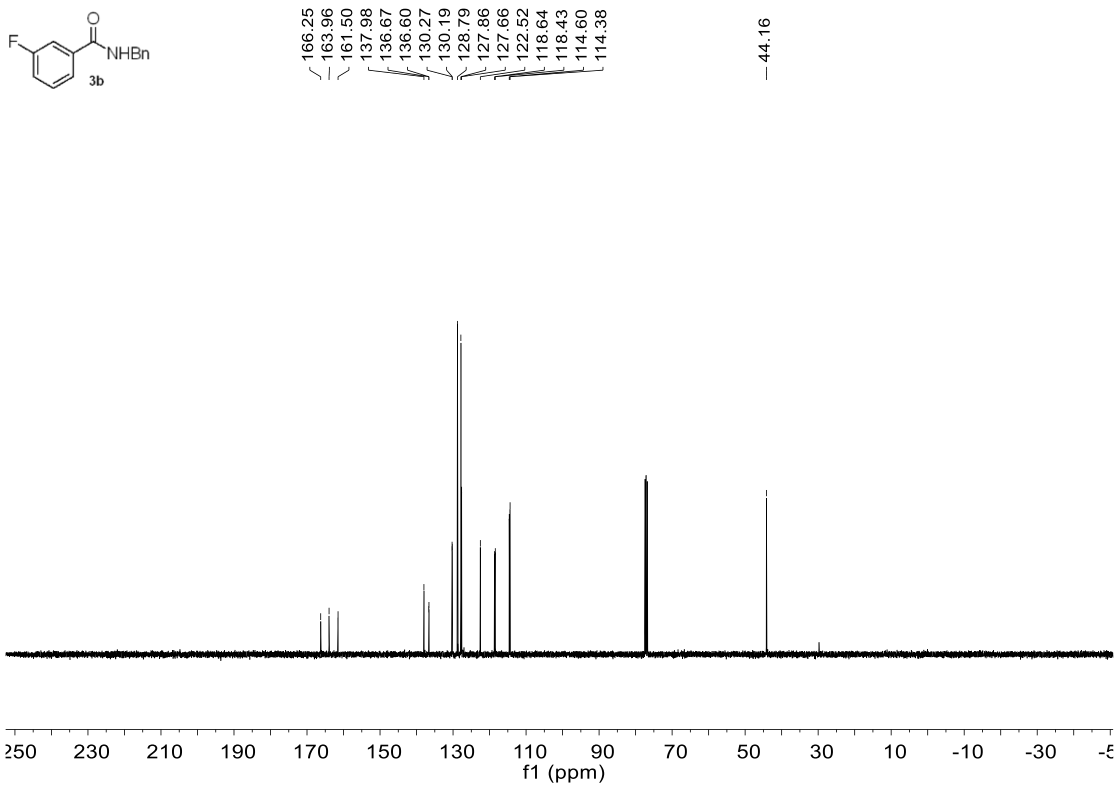

Supplementary Fig. 11. ^{13}C NMR spectrum (101 MHz, $CDCl_3$) of **3b**

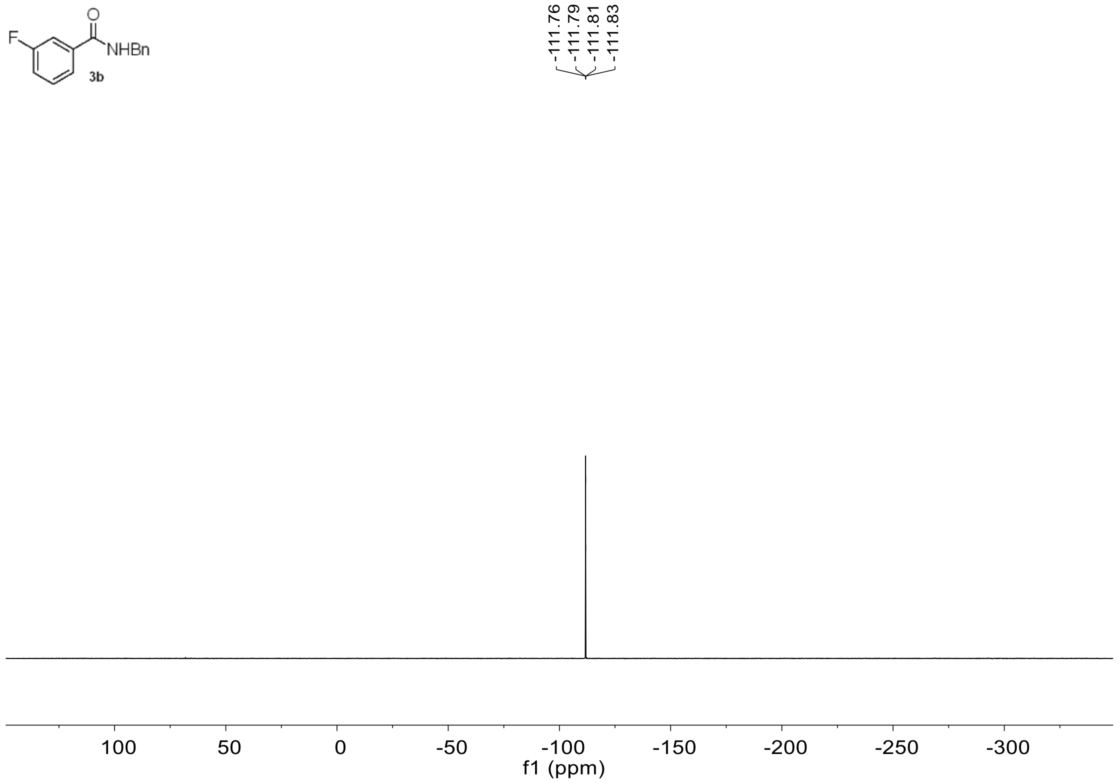

Supplementary Fig. 12. ^{19}F NMR spectrum (376 MHz, $CDCl_3$) of **3b**

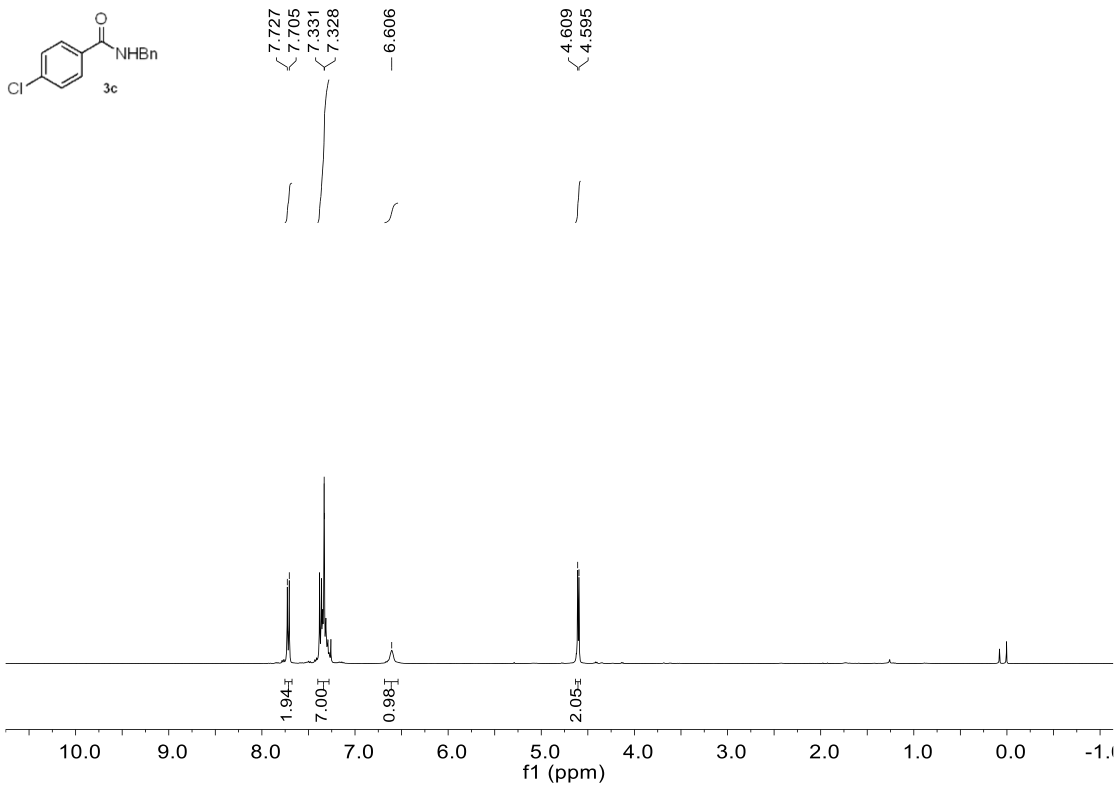

Supplementary Fig. 13. ^{1}H NMR spectrum (400 MHz, $CDCl_3$) of **3c**

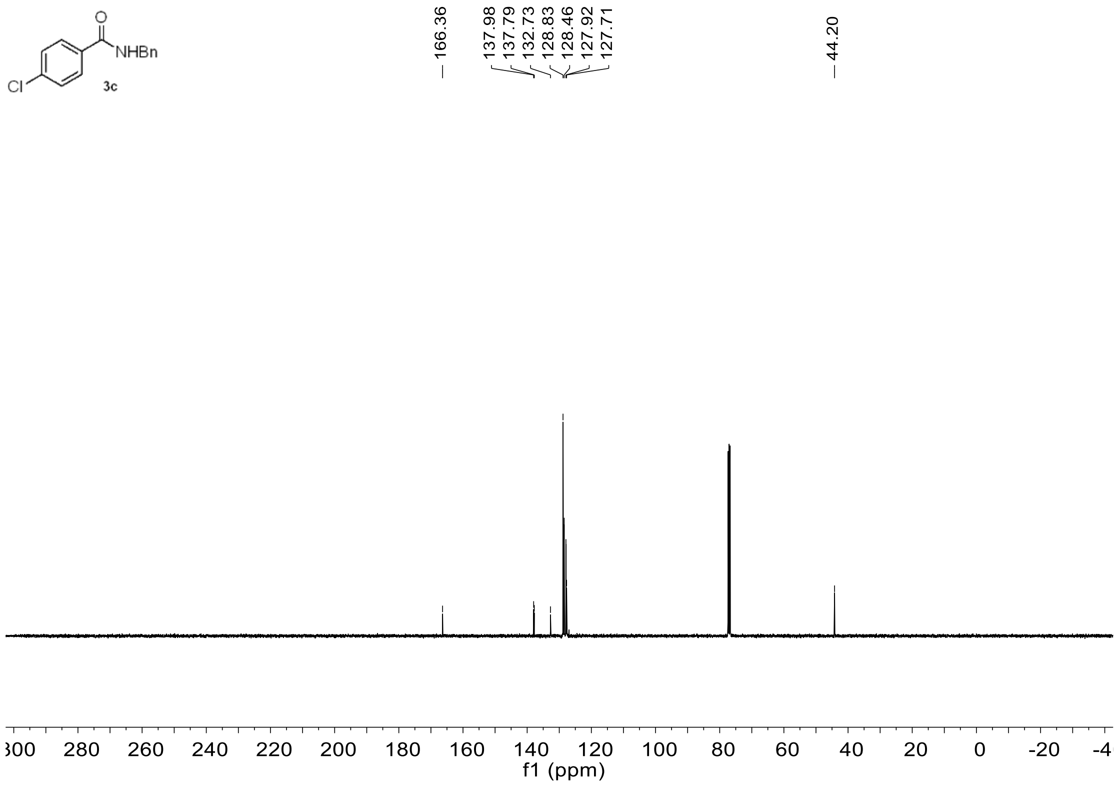

Supplementary Fig. 14. ^{13}C NMR spectrum (101 MHz, $CDCl_3$) of **3c**

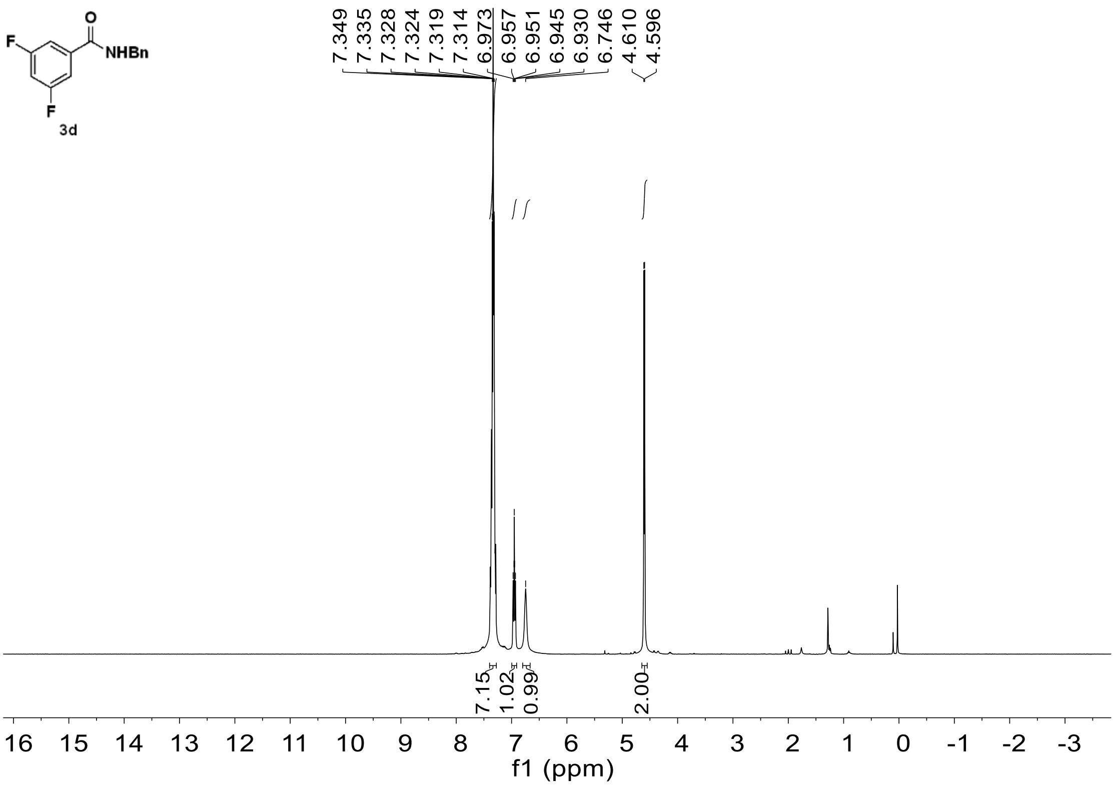

Supplementary Fig. 15. ^{1}H NMR spectrum (400 MHz, $CDCl_3$) of **3d**

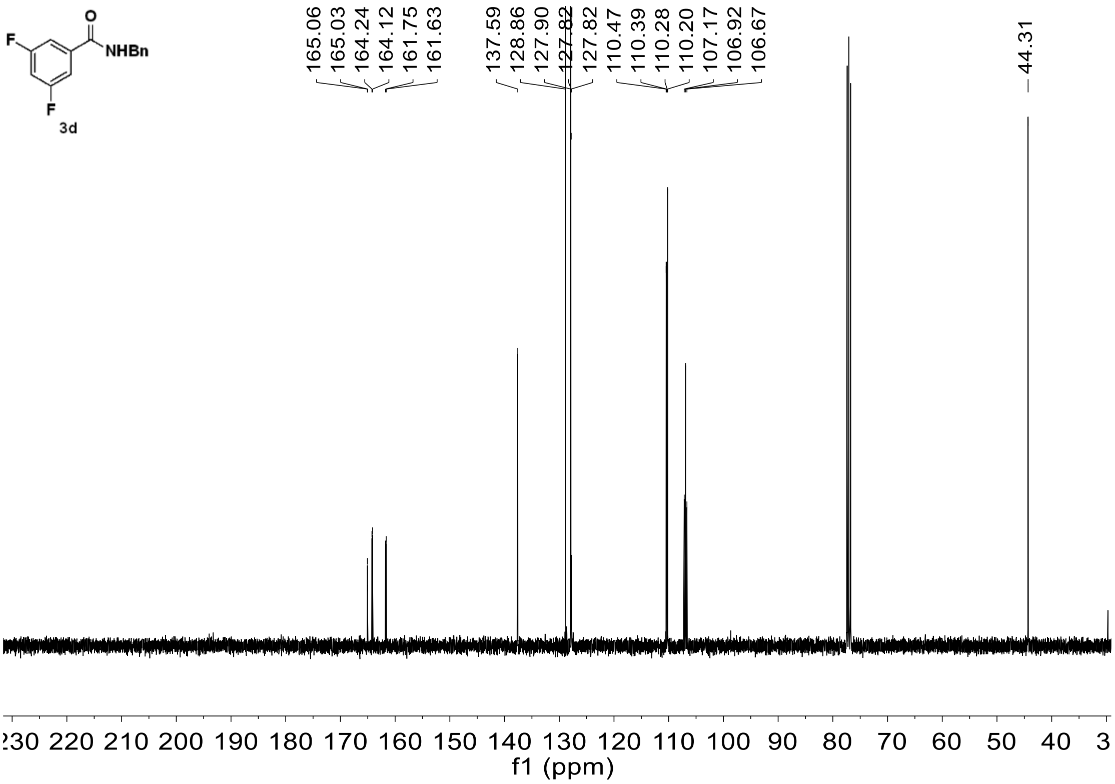

Supplementary Fig. 16. ^{13}C NMR spectrum (101 MHz, $CDCl_3$) of **3d**

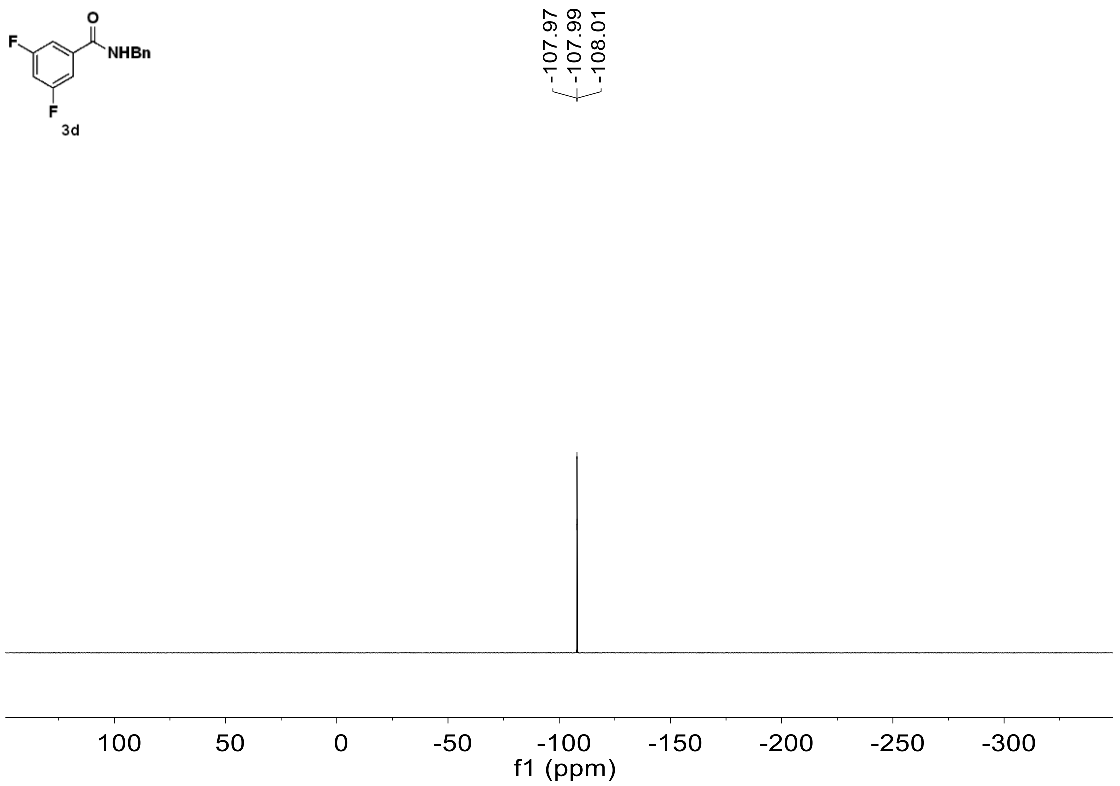

Supplementary Fig. 17. ^{19}F NMR spectrum (376 MHz, $CDCl_3$) of **3d**

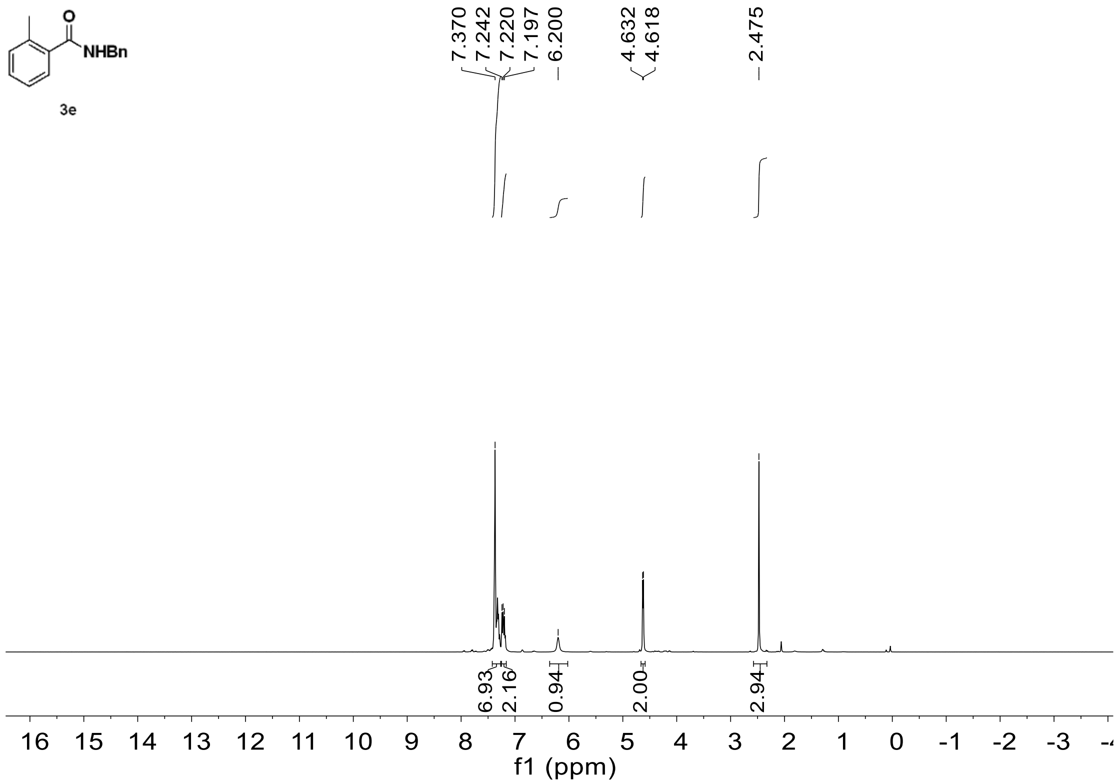

Supplementary Fig. 18. ^{1}H NMR spectrum (400 MHz, $CDCl_3$) of **3e**

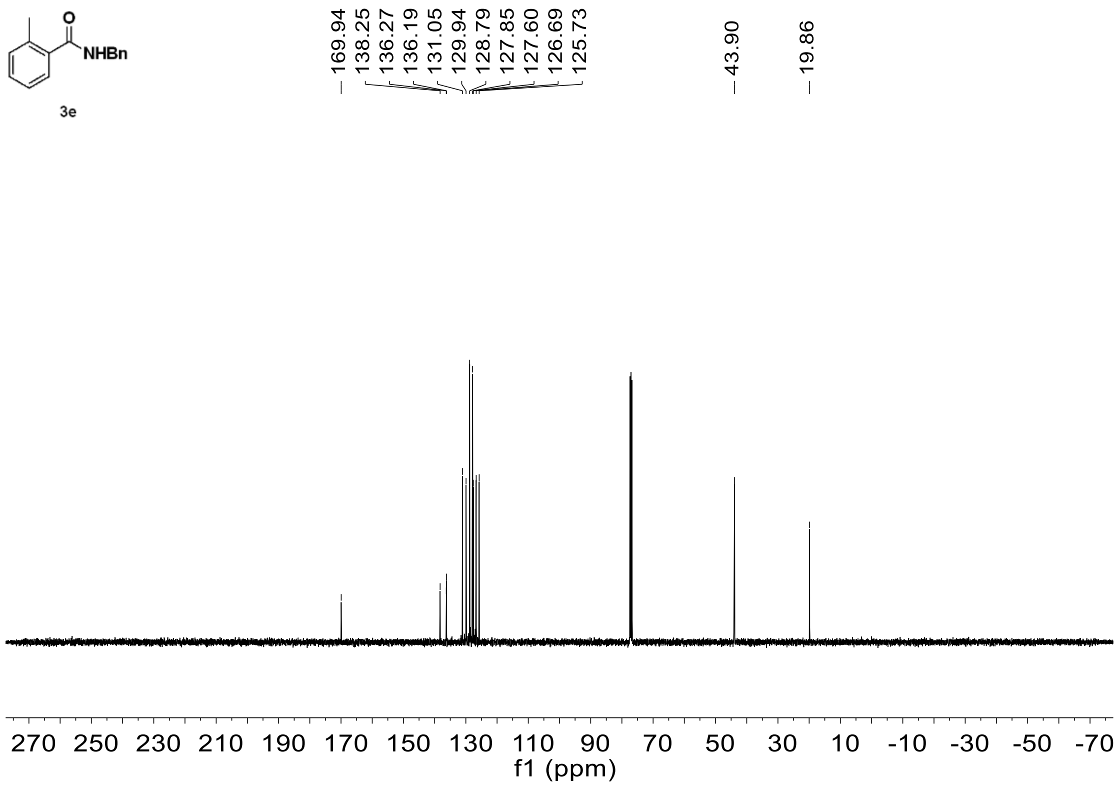

Supplementary Fig. 19. ^{13}C NMR spectrum (101 MHz, $CDCl_3$) of **3e**

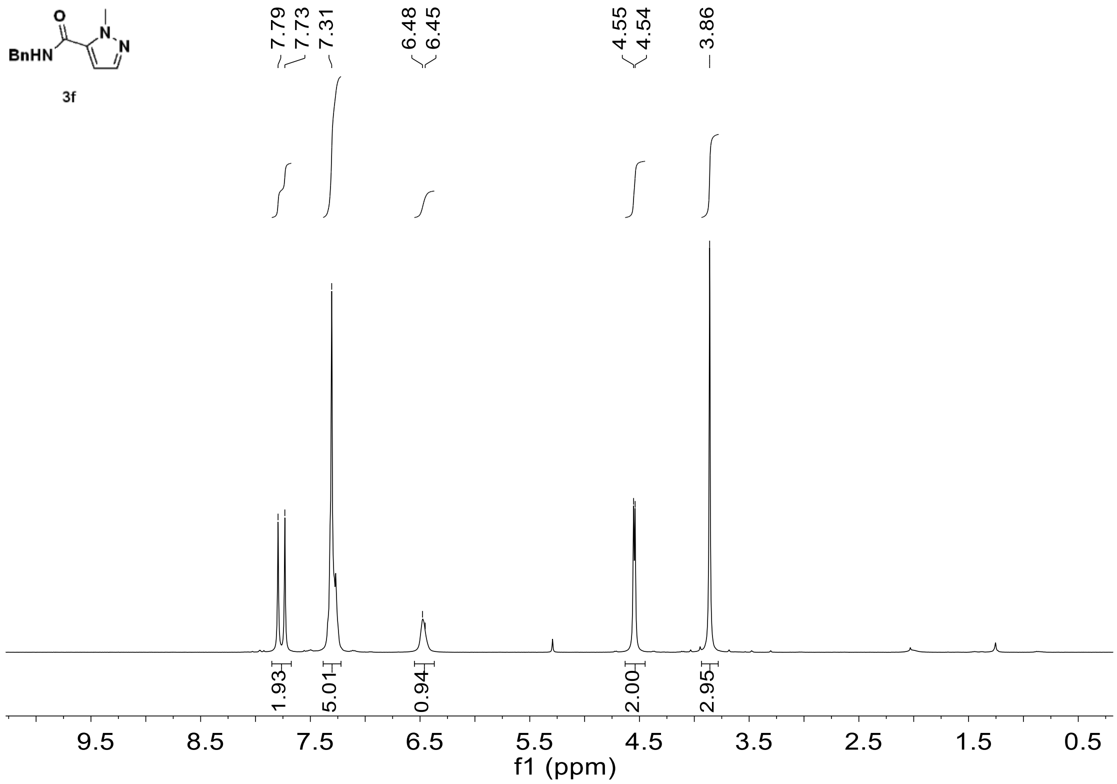

Supplementary Fig. 20. ^{1}H NMR spectrum (400 MHz, $CDCl_3$) of **3f**

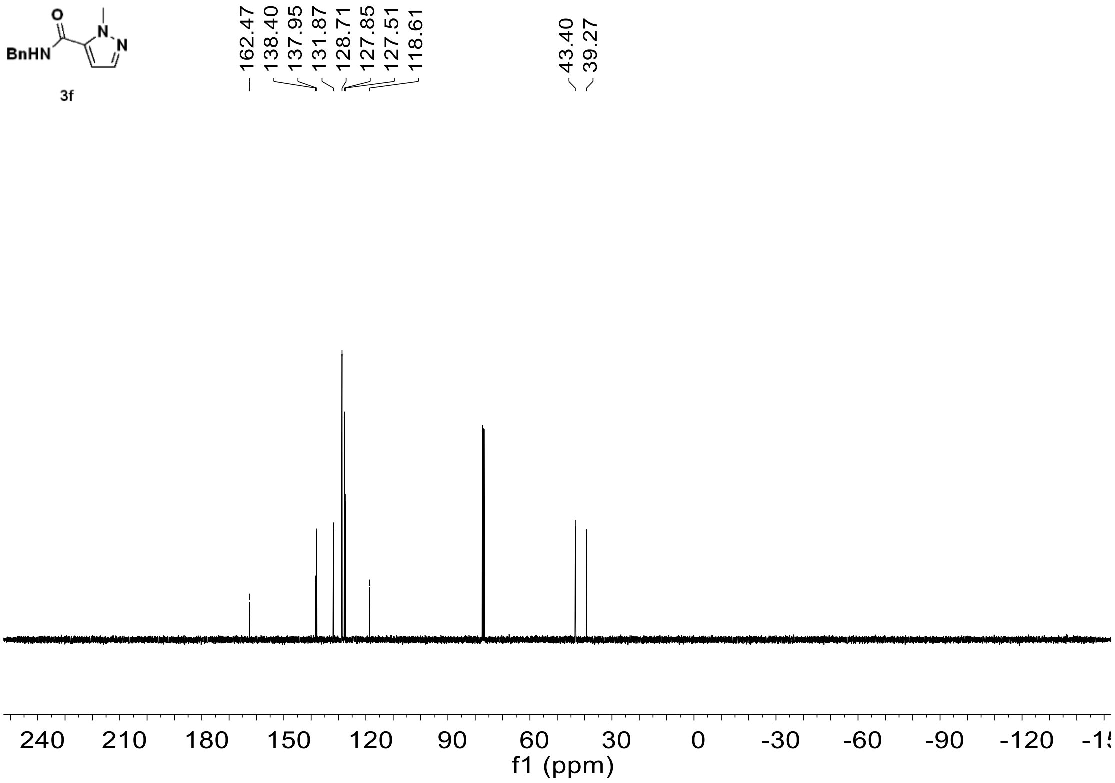

Supplementary Fig. 21. ^{13}C NMR spectrum (101 MHz, $CDCl_3$) of **3f**

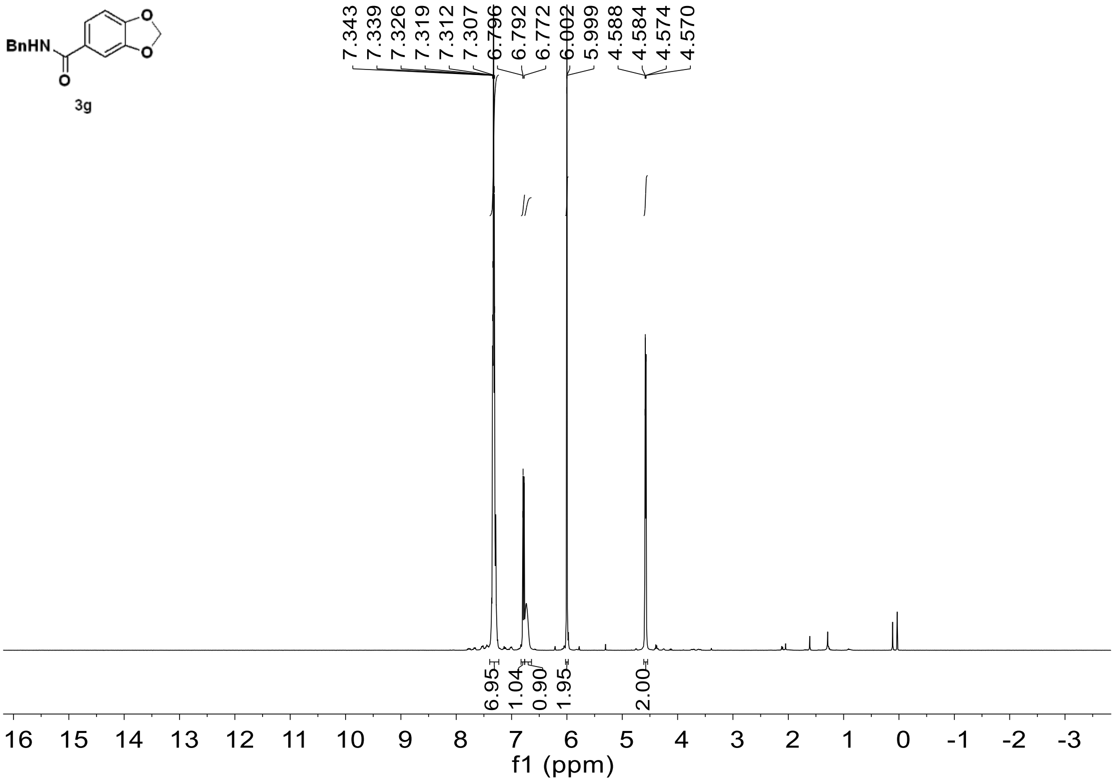

Supplementary Fig. 22. ^{1}H NMR spectrum (400 MHz, $CDCl_3$) of **3g**

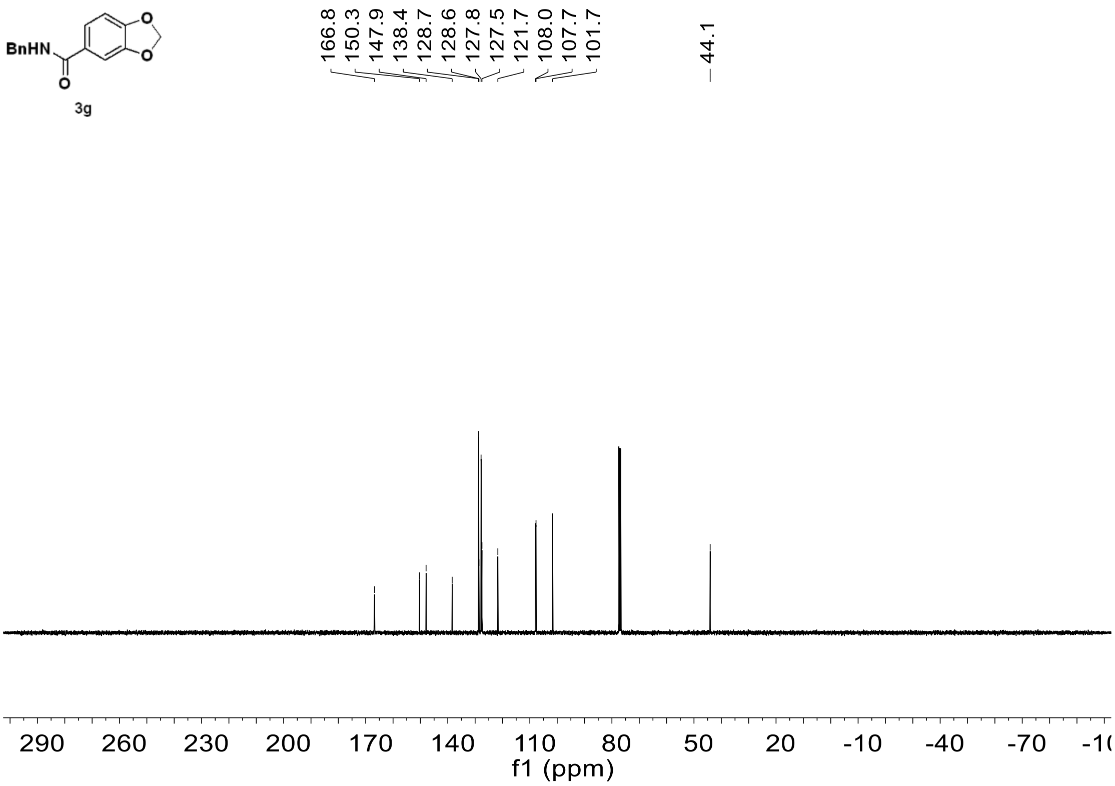

Supplementary Fig. 23. ^{13}C NMR spectrum (101 MHz, $CDCl_3$) of **3g**

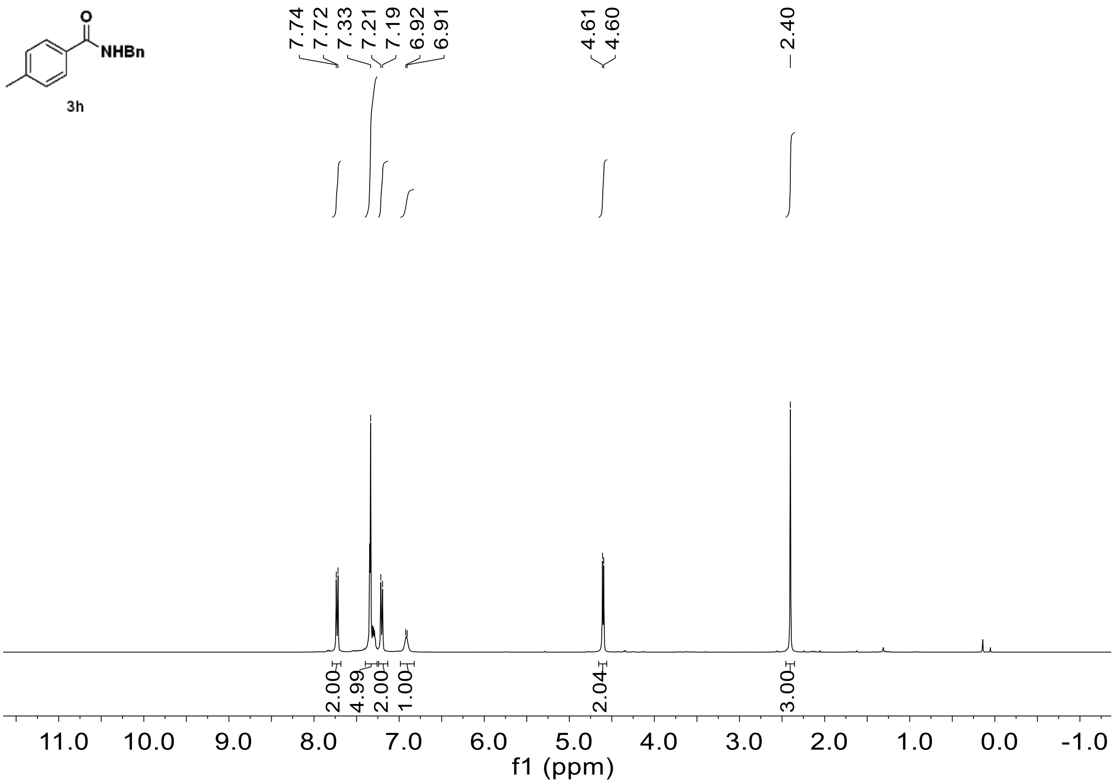

Supplementary Fig. 24. ^{1}H NMR spectrum (400 MHz, $CDCl_3$) of **3h**

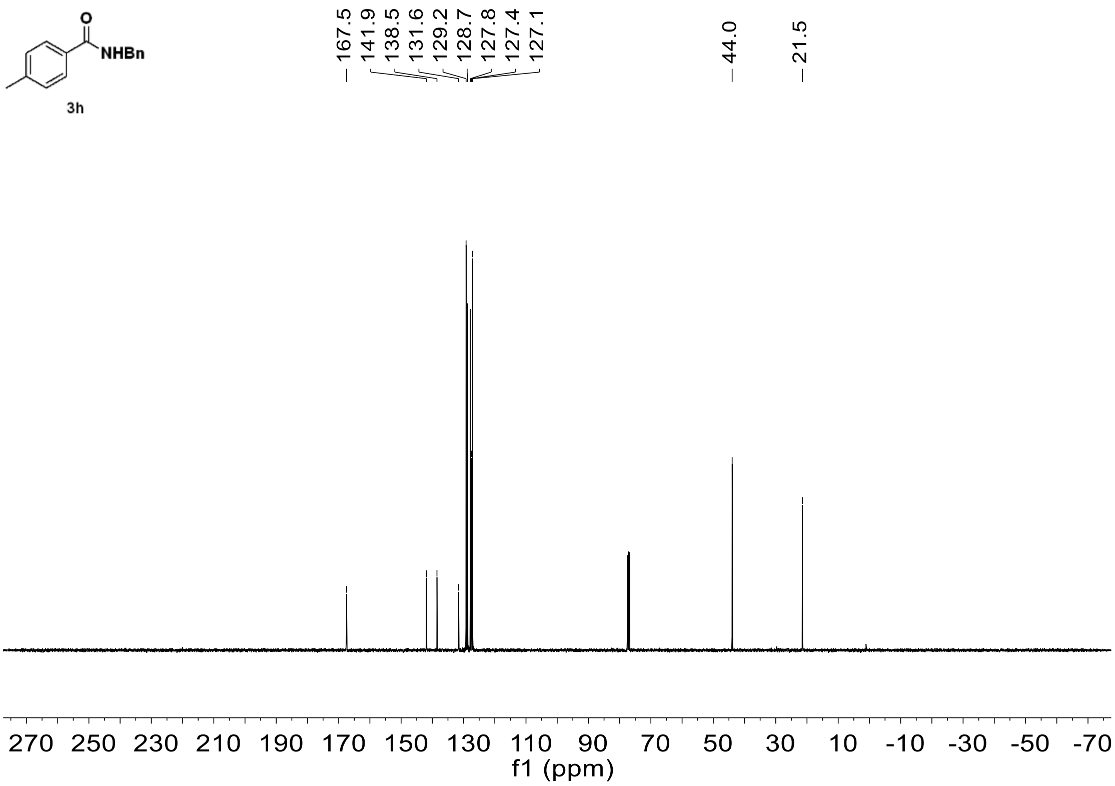

Supplementary Fig. 25. ^{13}C NMR spectrum (101 MHz, $CDCl_3$) of **3h**

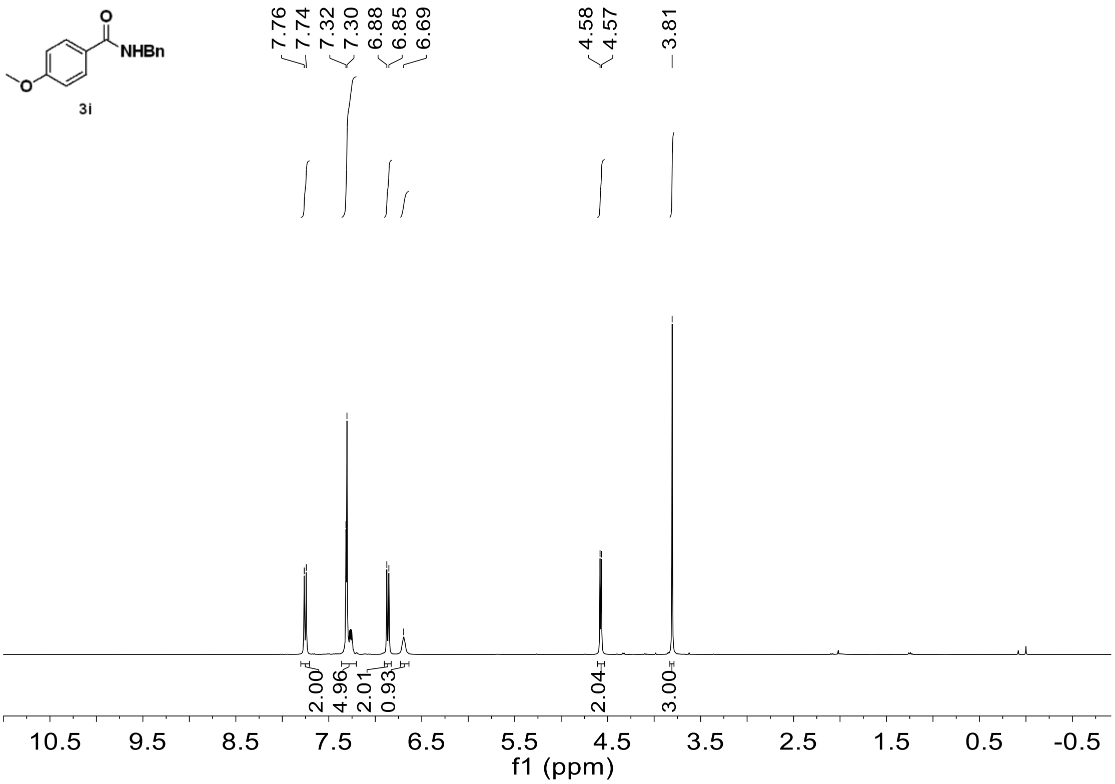

Supplementary Fig. 26. ^{1}H NMR spectrum (400 MHz, $CDCl_3$) of **3i**

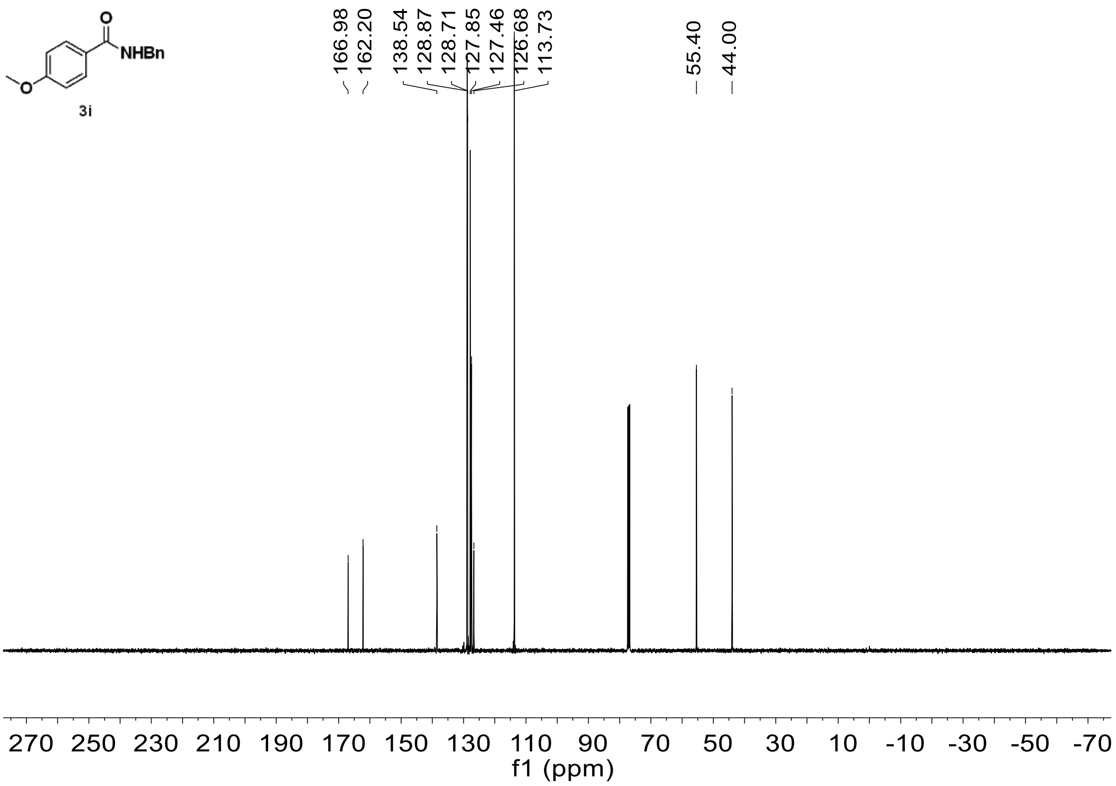

Supplementary Fig. 27. ^{13}C NMR spectrum (101 MHz, $CDCl_3$) of **3i**

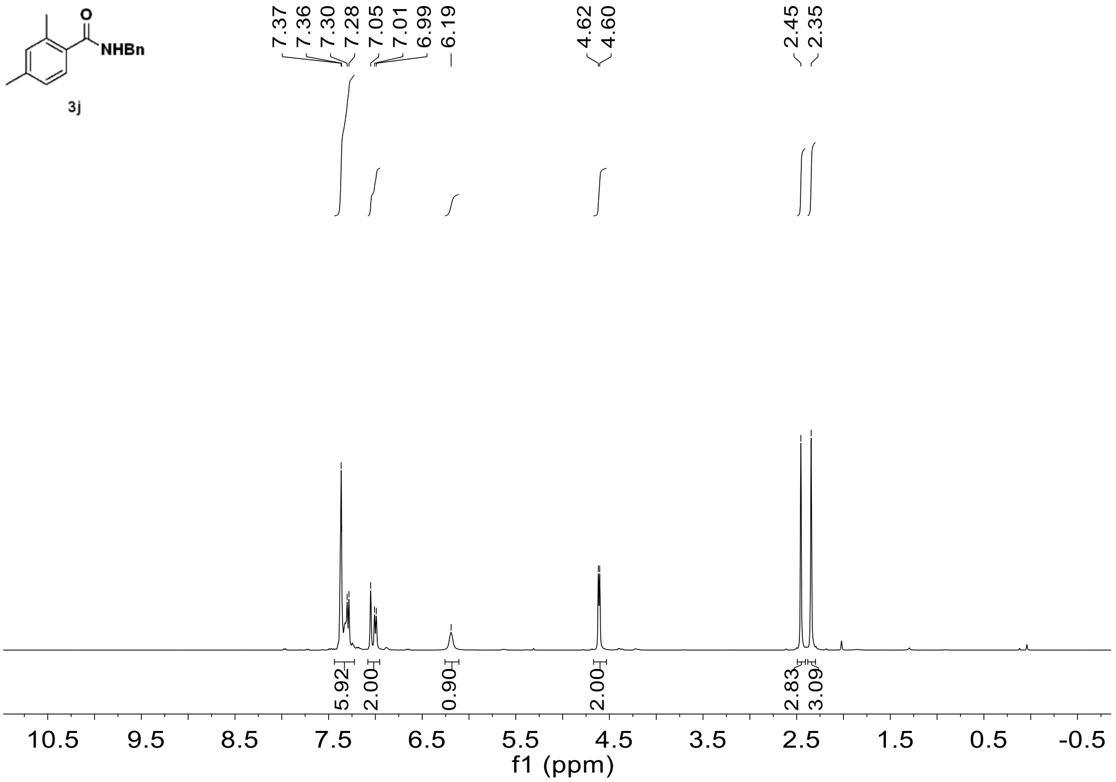

Supplementary Fig. 28. ^{1}H NMR spectrum (400 MHz, $CDCl_3$) of **3j**

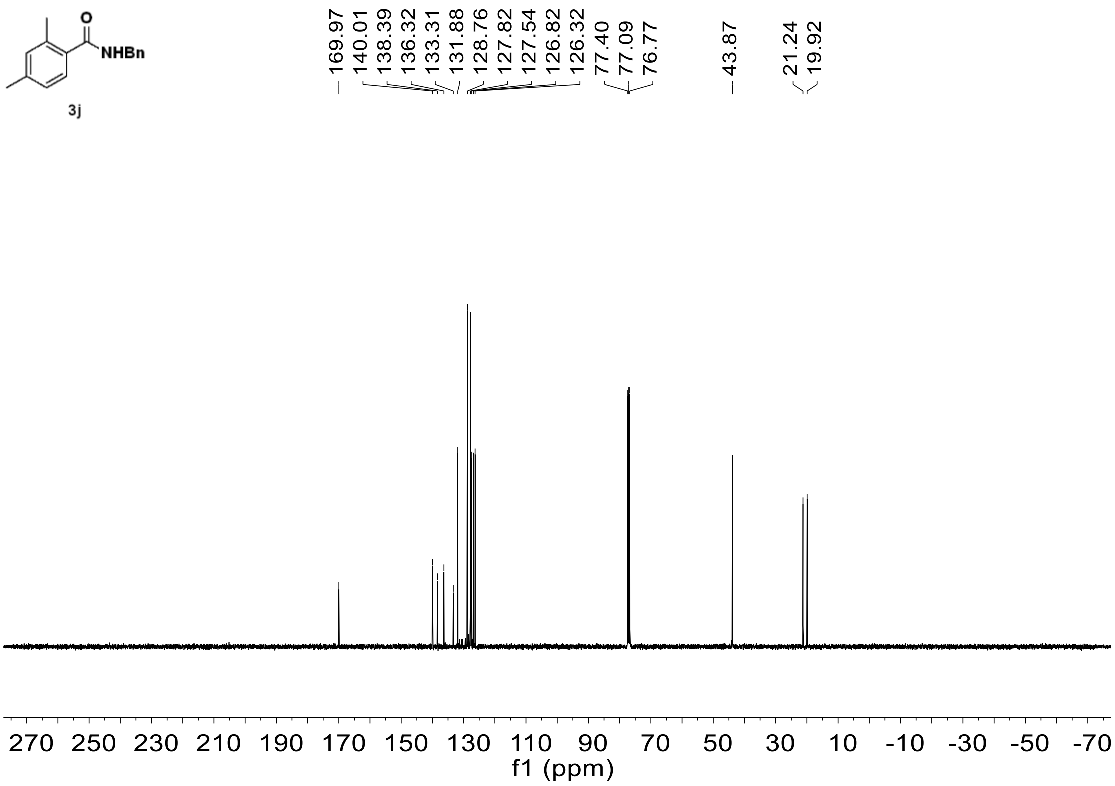

Supplementary Fig. 29. ^{13}C NMR spectrum (101 MHz, $CDCl_3$) of **3j**

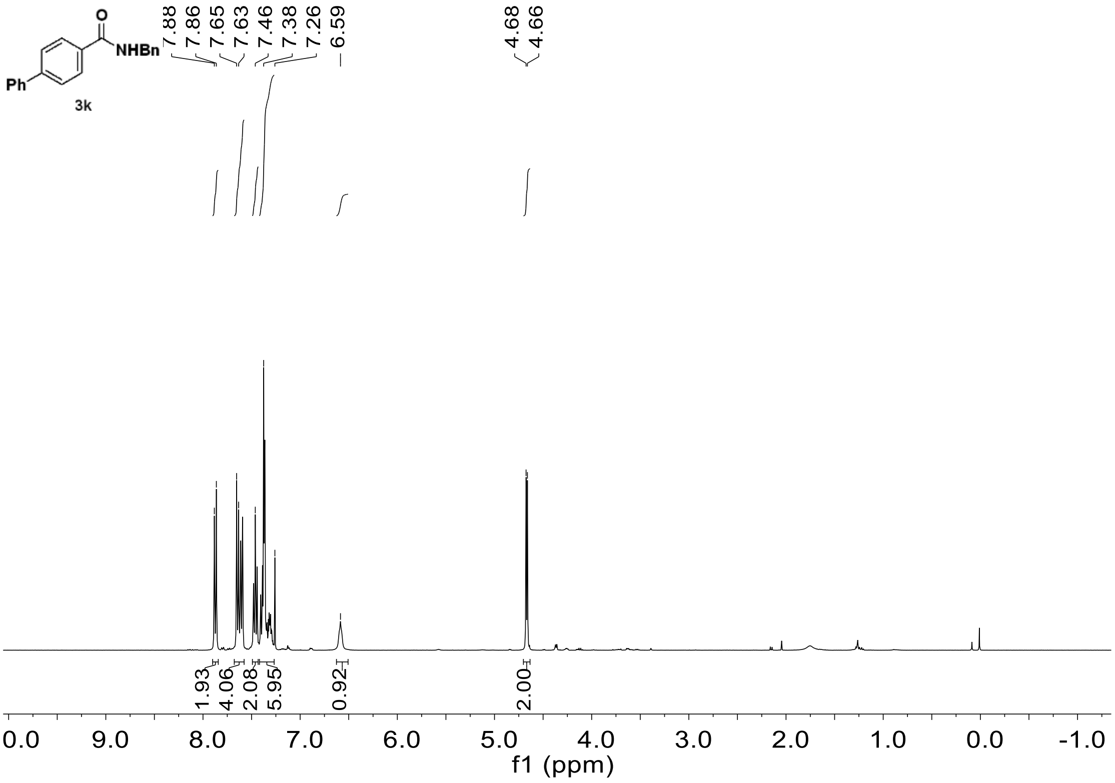

Supplementary Fig. 30. ^{1}H NMR spectrum (400 MHz, $CDCl_3$) of **3k**

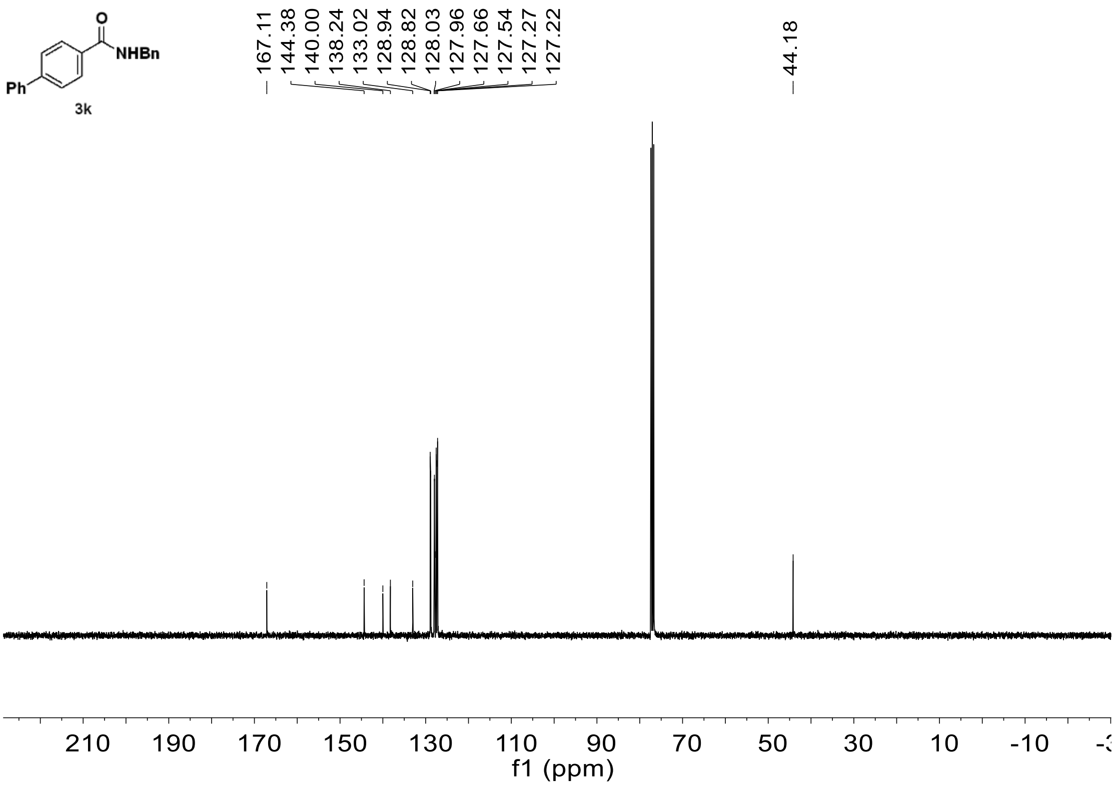

Supplementary Fig. 31. ^{13}C NMR spectrum (101 MHz, $CDCl_3$) of **3k**

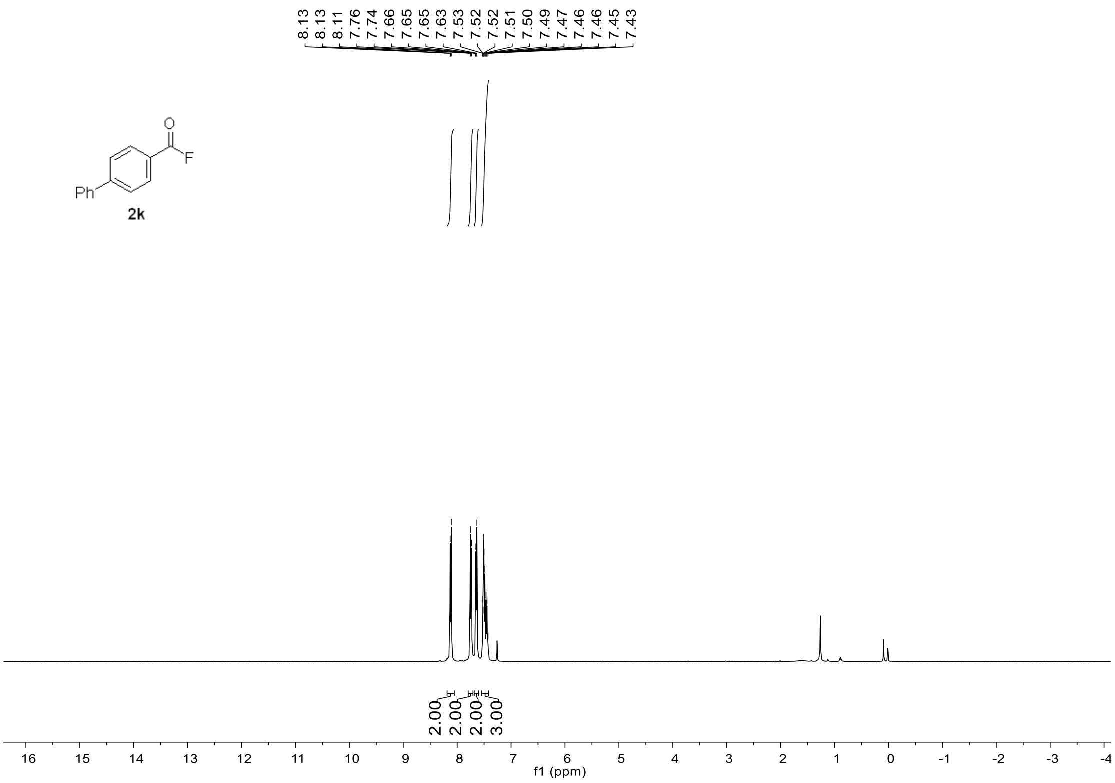

Supplementary Fig. 32. ^{1}H NMR spectrum (400 MHz, $CDCl_3$) of **2k**

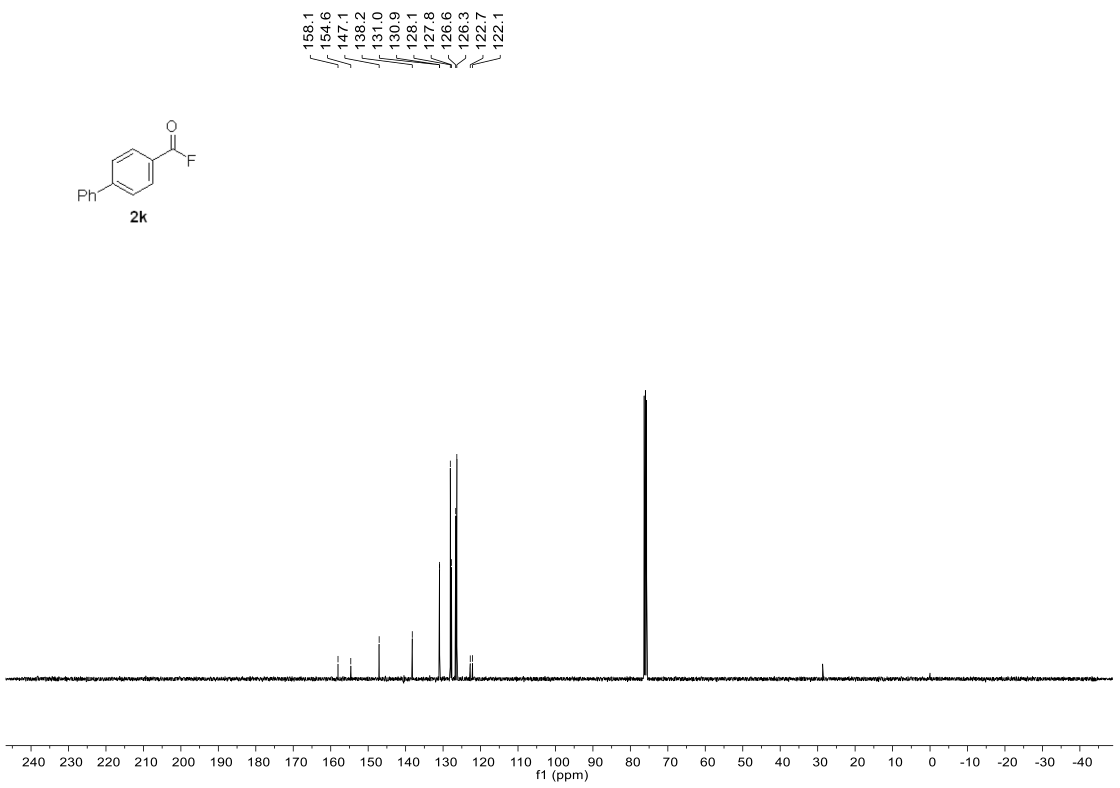

Supplementary Fig. 33. ^{13}C NMR spectrum (101 MHz, $CDCl_3$) of **2k**

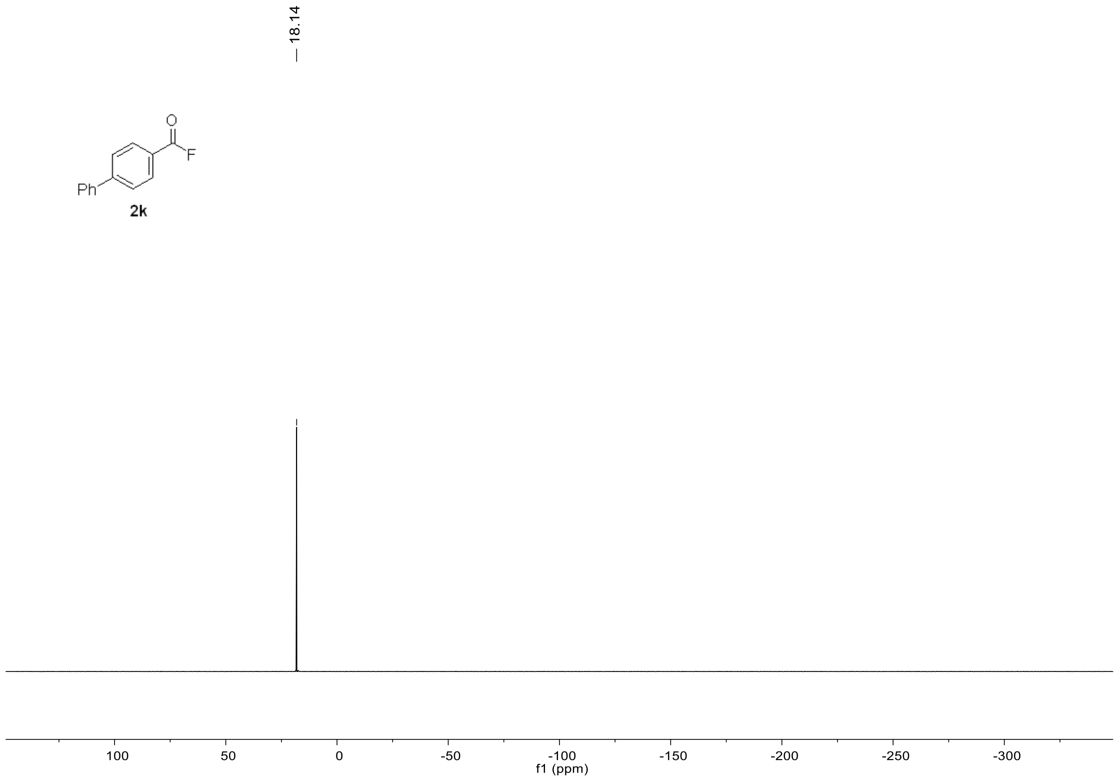

Supplementary Fig. 34. ^{19}F NMR spectrum (376 MHz, $CDCl_3$) of **2k**

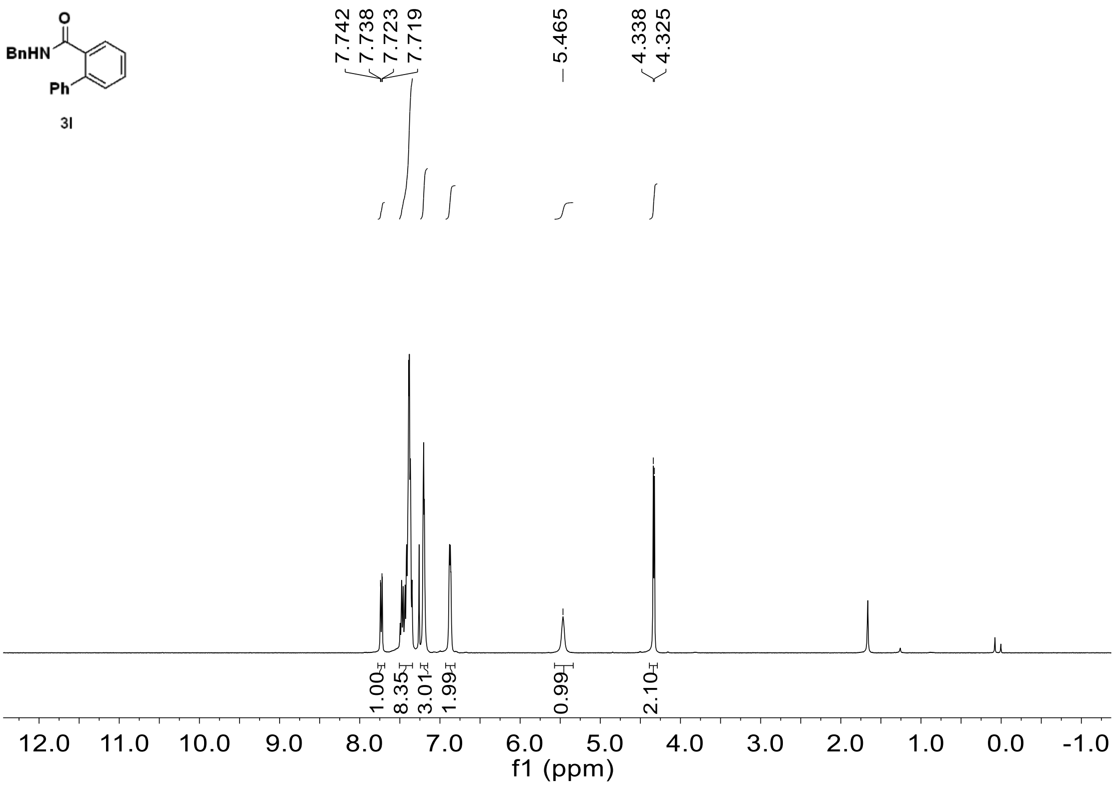

Supplementary Fig. 35. ^{1}H NMR spectrum (400 MHz, $CDCl_3$) of **3l**

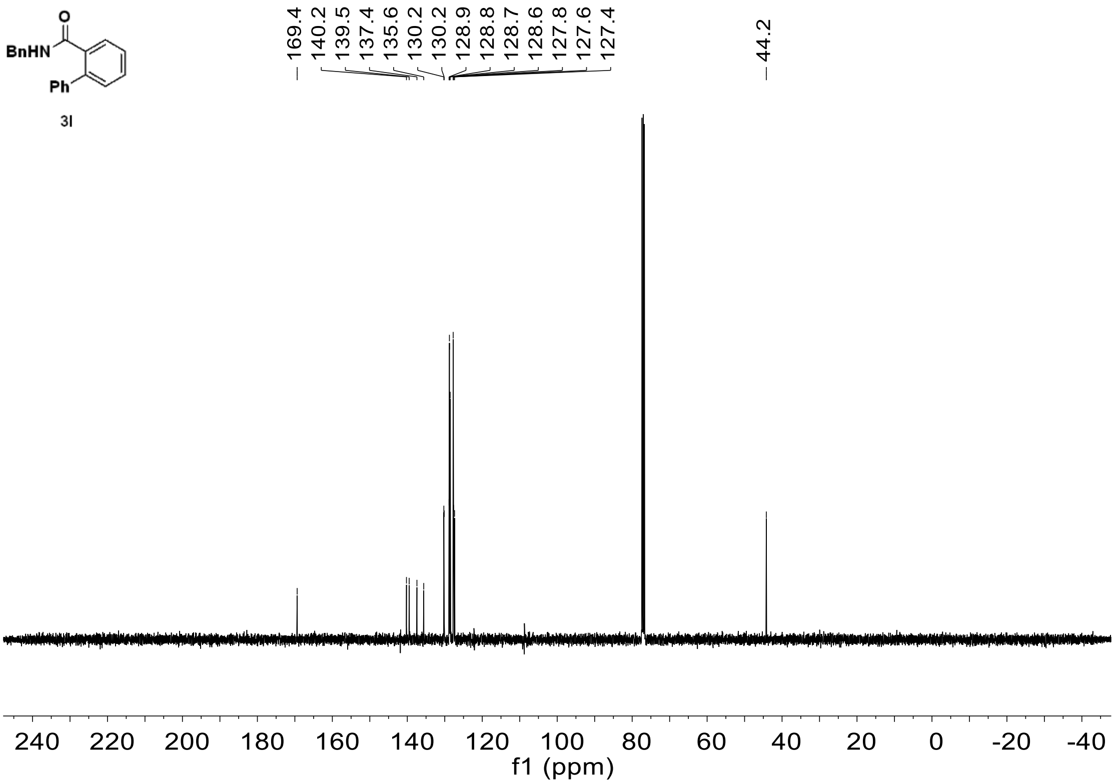

Supplementary Fig. 36. ^{13}C NMR spectrum (101 MHz, $CDCl_3$) of **3l**

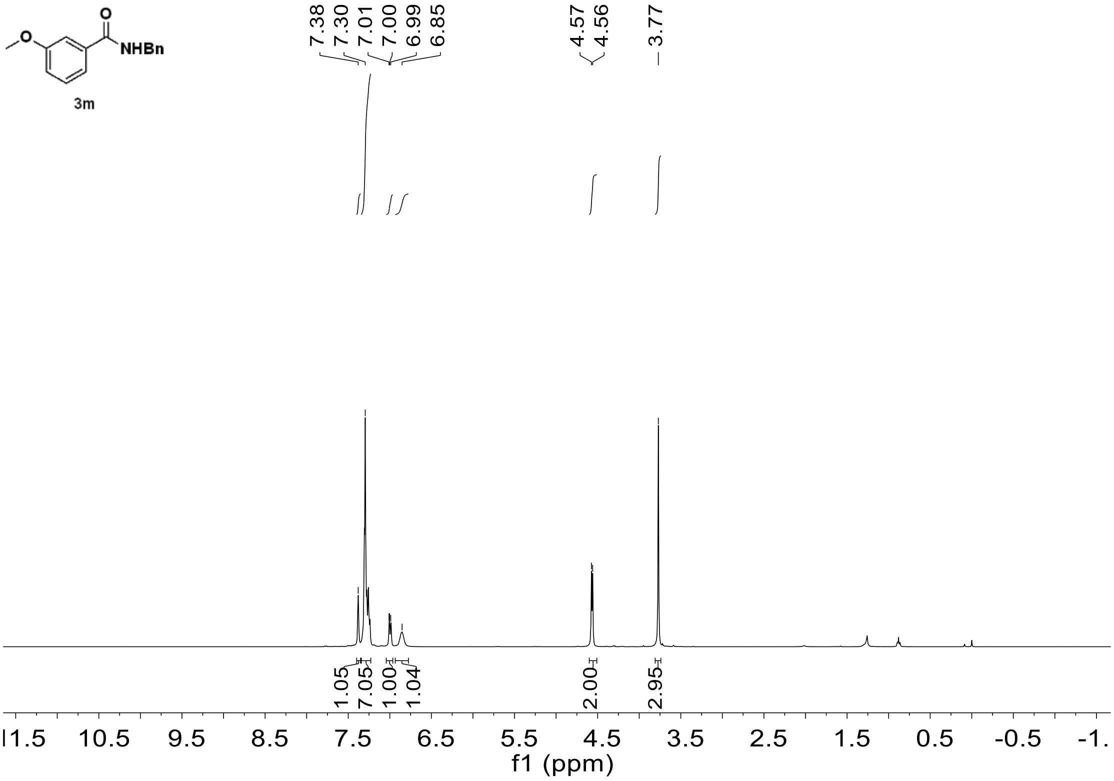

Supplementary Fig. 37. ^{1}H NMR spectrum (400 MHz, $CDCl_3$) of **3m**

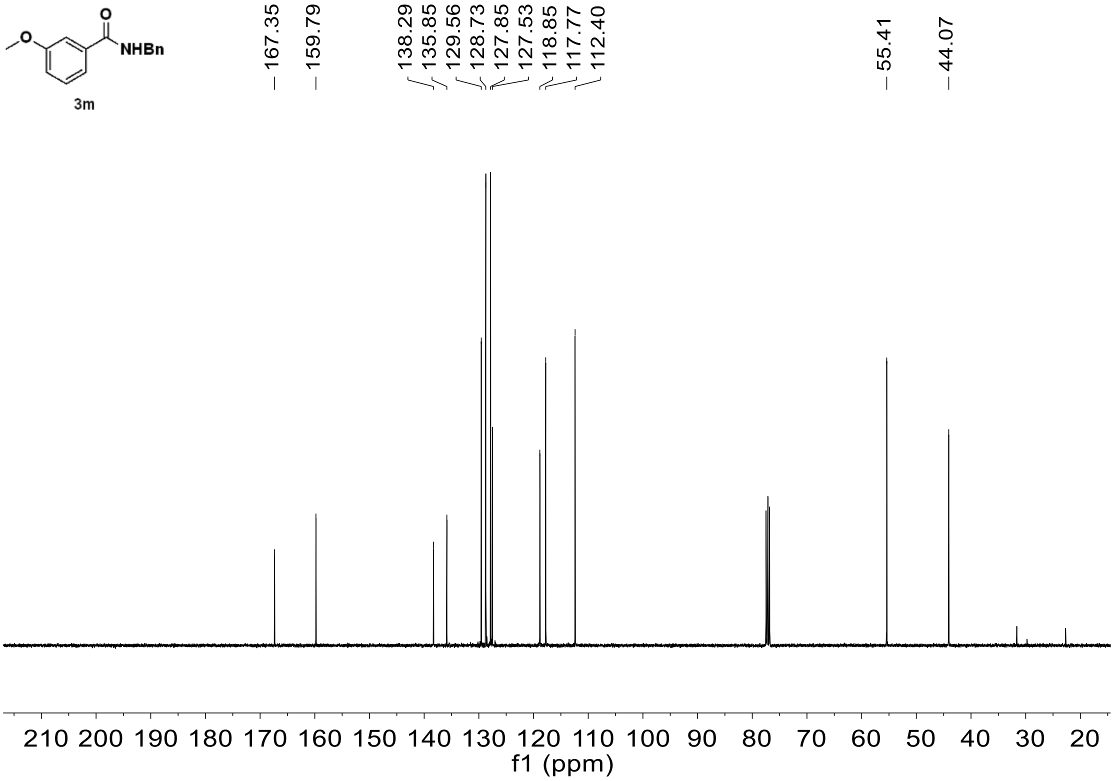

Supplementary Fig. 38. ^{13}C NMR spectrum (101 MHz, $CDCl_3$) of **3m**

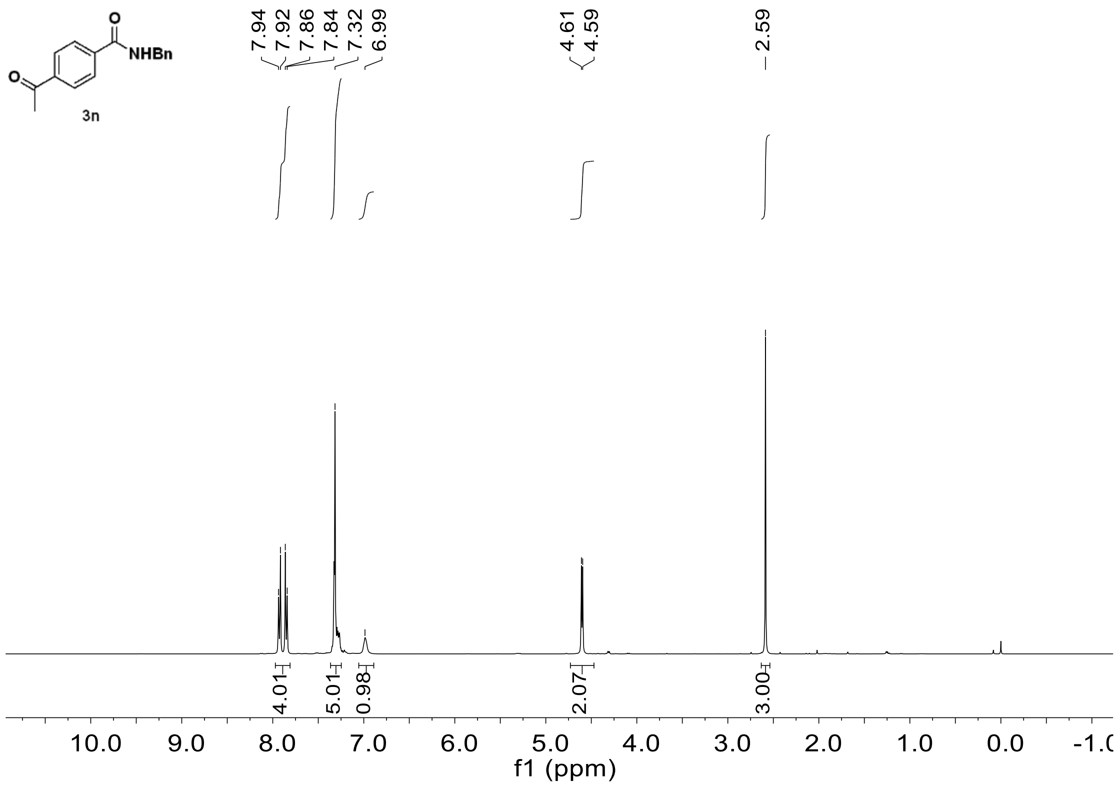

Supplementary Fig. 39. ^{1}H NMR spectrum (400 MHz, $CDCl_3$) of **3n**

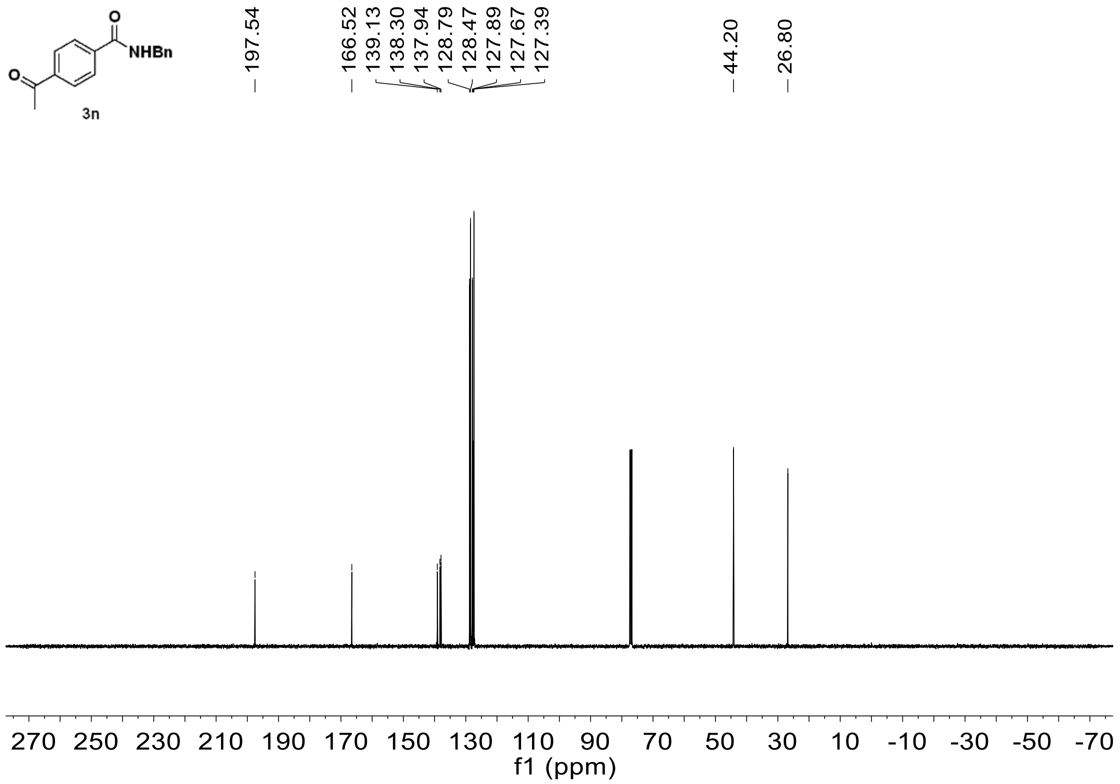

Supplementary Fig. 40. ^{13}C NMR spectrum (101 MHz, $CDCl_3$) of **3n**

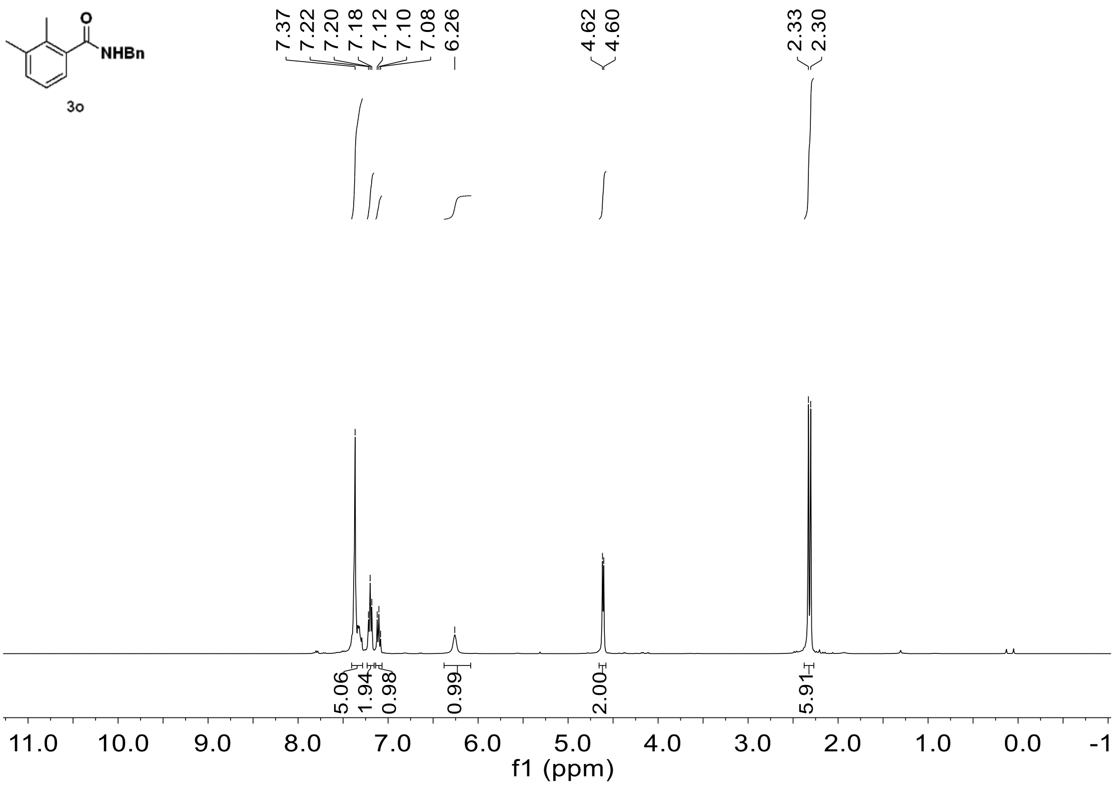

Supplementary Fig. 41. ^{1}H NMR spectrum (400 MHz, $CDCl_3$) of **3o**

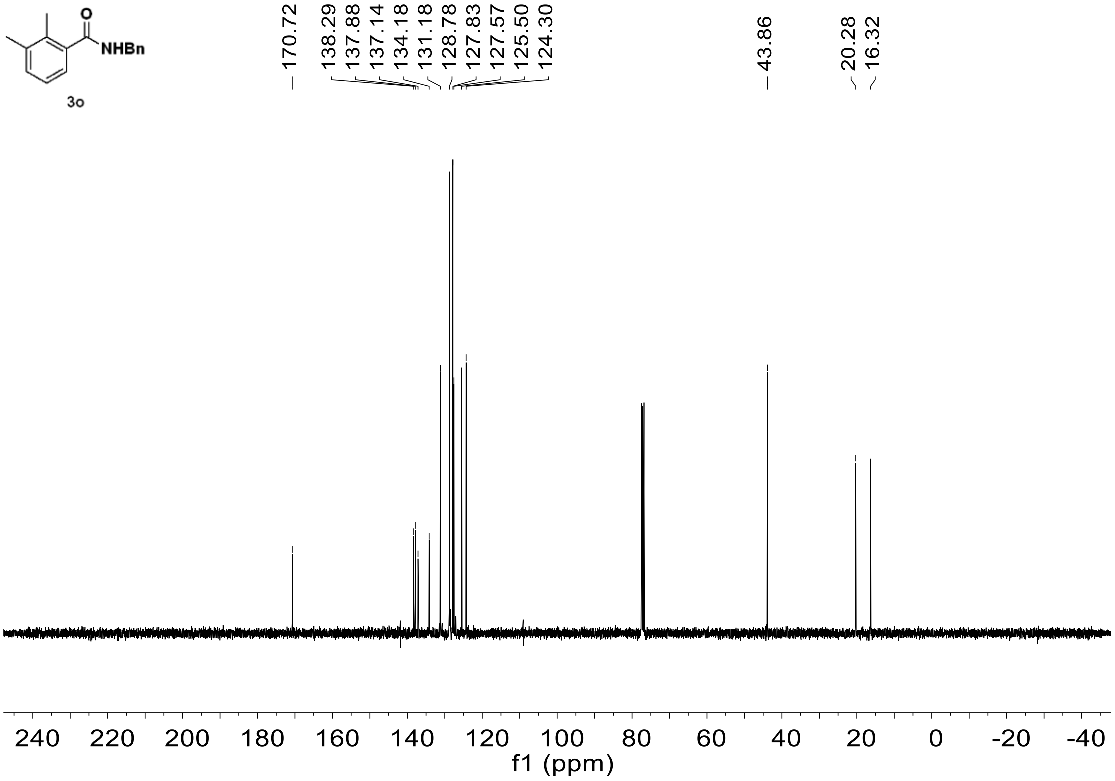

Supplementary Fig. 42. ^{13}C NMR spectrum (101 MHz, $CDCl_3$) of **3o**

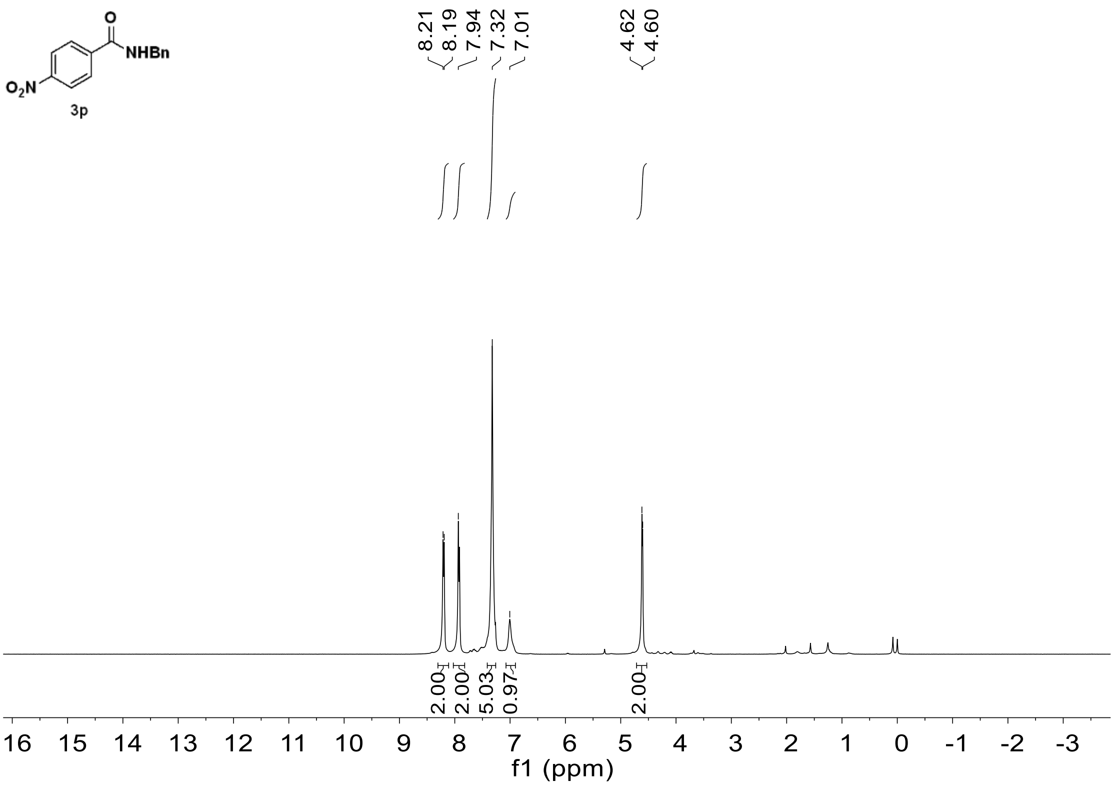

Supplementary Fig. 43. ^{1}H NMR spectrum (400 MHz, $CDCl_3$) of **3p**

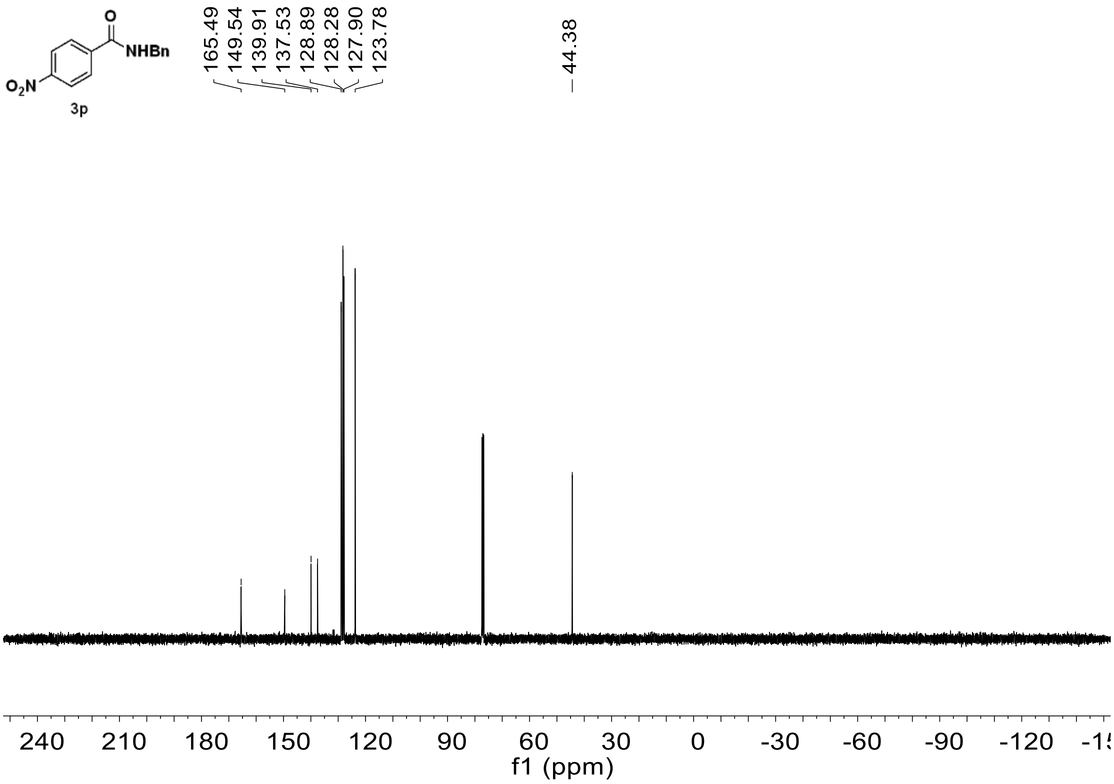

Supplementary Fig. 44. ^{13}C NMR spectrum (101 MHz, $CDCl_3$) of **3p**

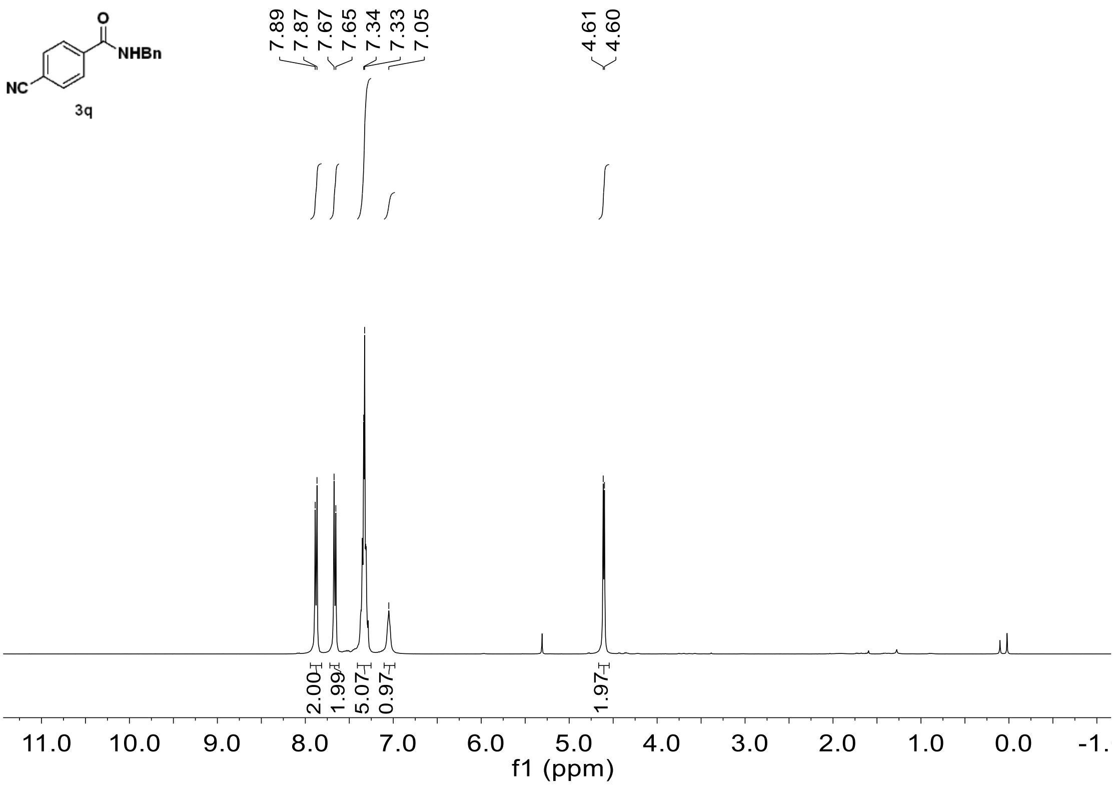

Supplementary Fig. 45. ^{1}H NMR spectrum (400 MHz, $CDCl_3$) of **3q**

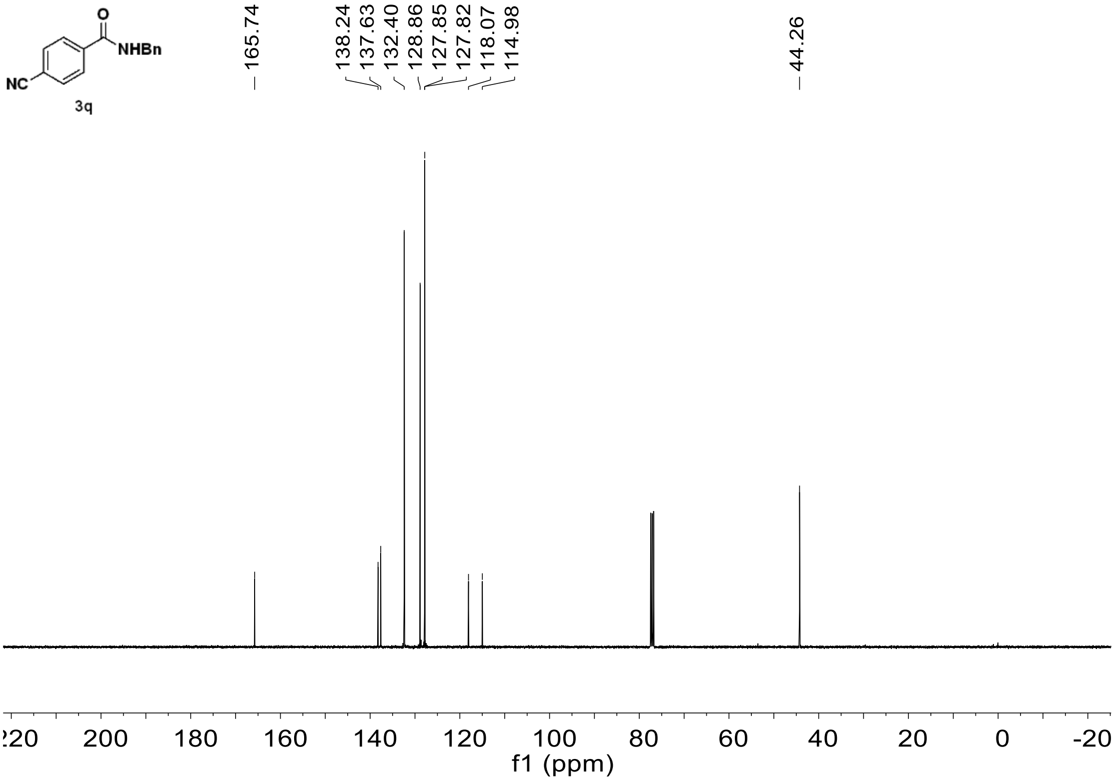

Supplementary Fig. 46. ^{13}C NMR spectrum (101 MHz, $CDCl_3$) of **3q**

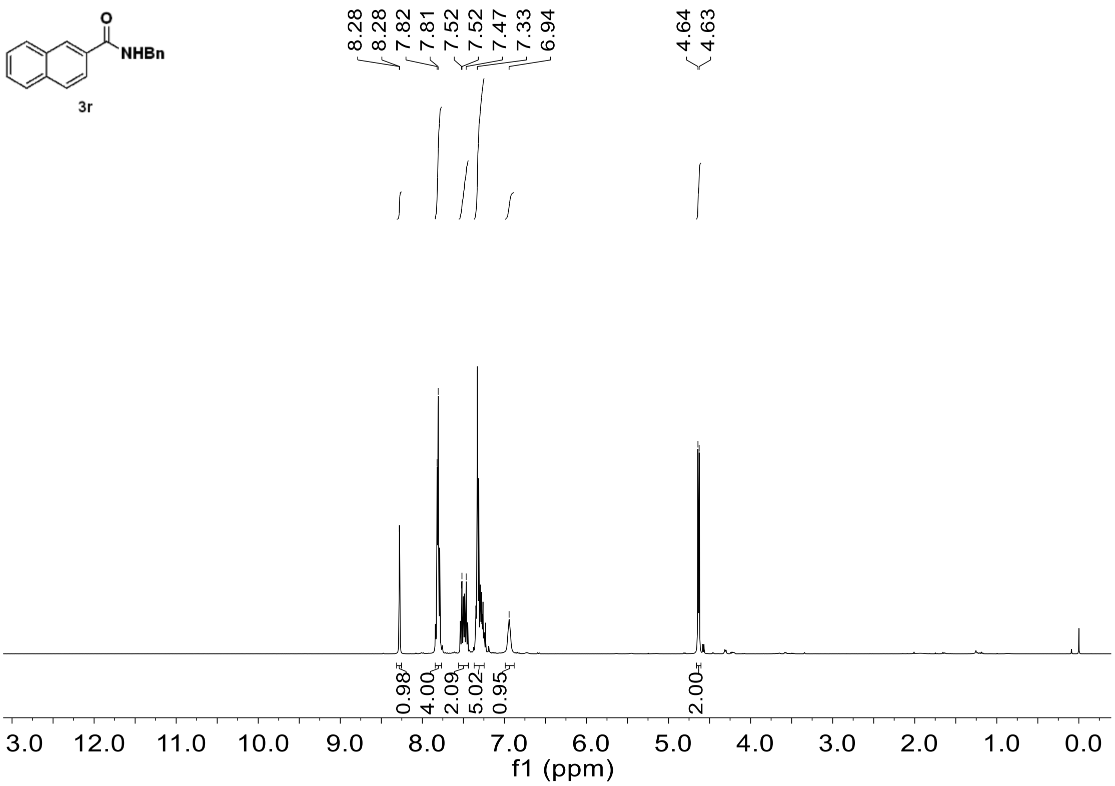

Supplementary Fig. 47. ^{1}H NMR spectrum (400 MHz, $CDCl_3$) of **3r**

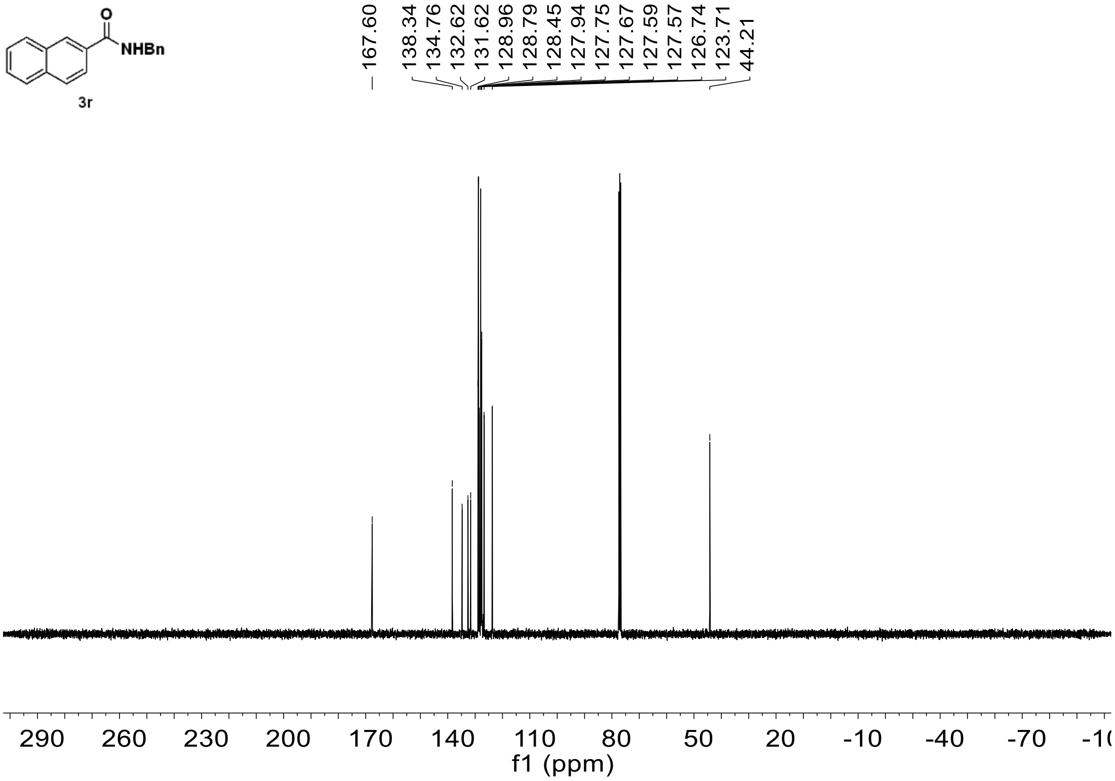

Supplementary Fig. 48. ^{13}C NMR spectrum (101 MHz, $CDCl_3$) of **3r**

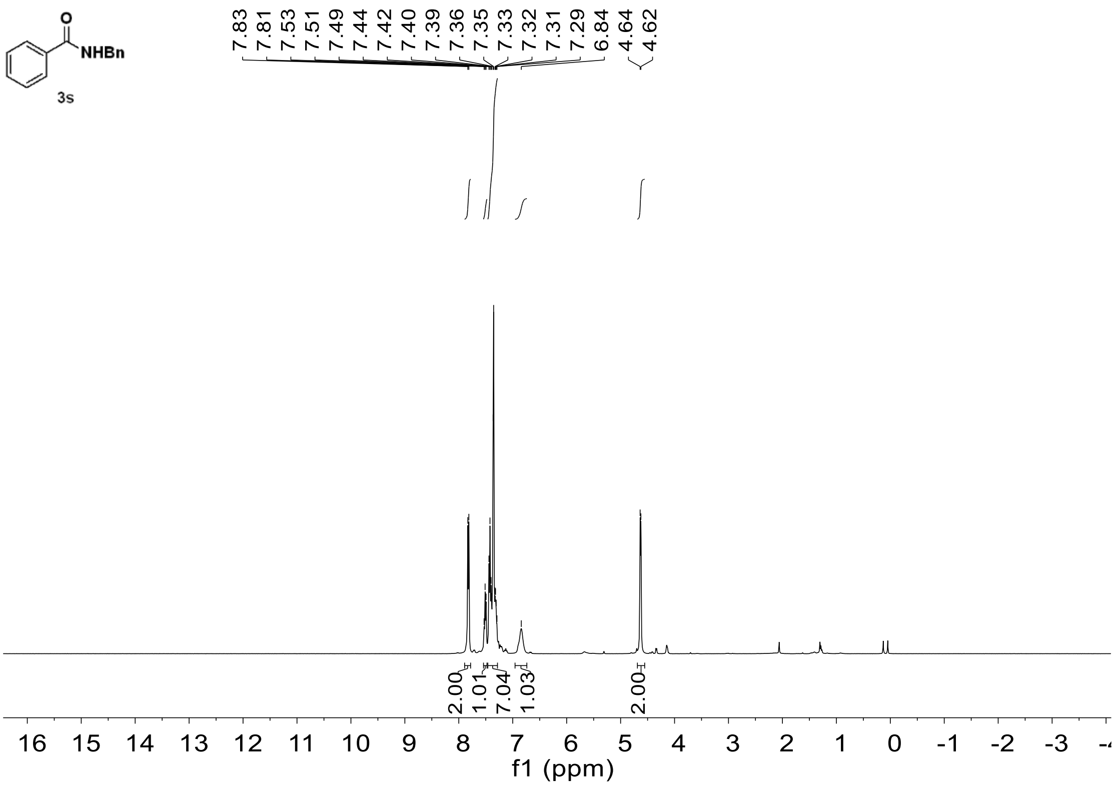

Supplementary Fig. 49. ^{1}H NMR spectrum (400 MHz, $CDCl_3$) of **3s**

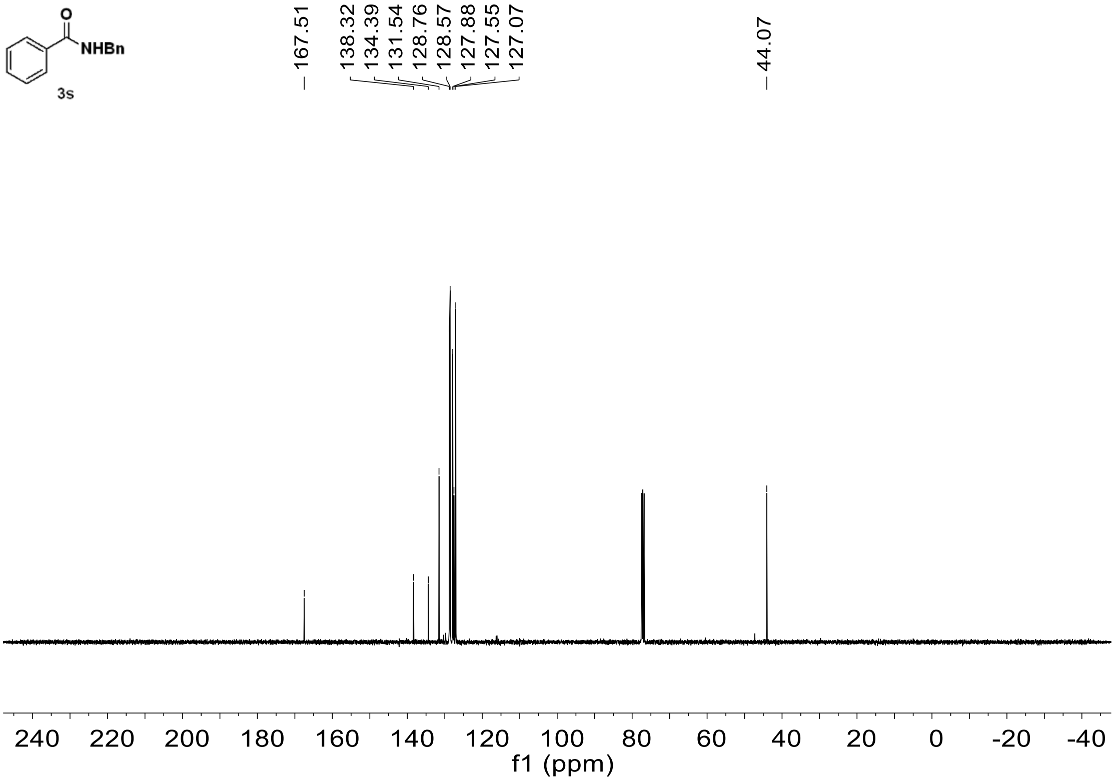

Supplementary Fig. 50. ^{13}C NMR spectrum (101 MHz, $CDCl_3$) of **3s**

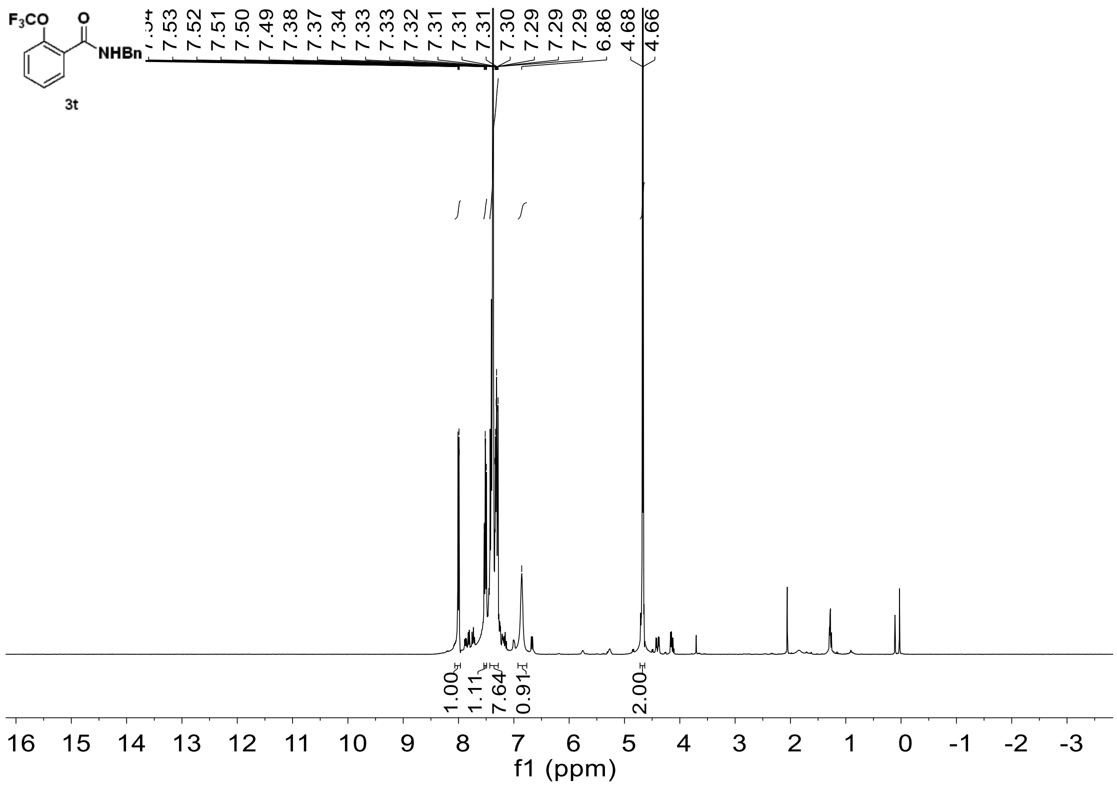

Supplementary Fig. 51. ^{1}H NMR spectrum (400 MHz, $CDCl_3$) of **3t**

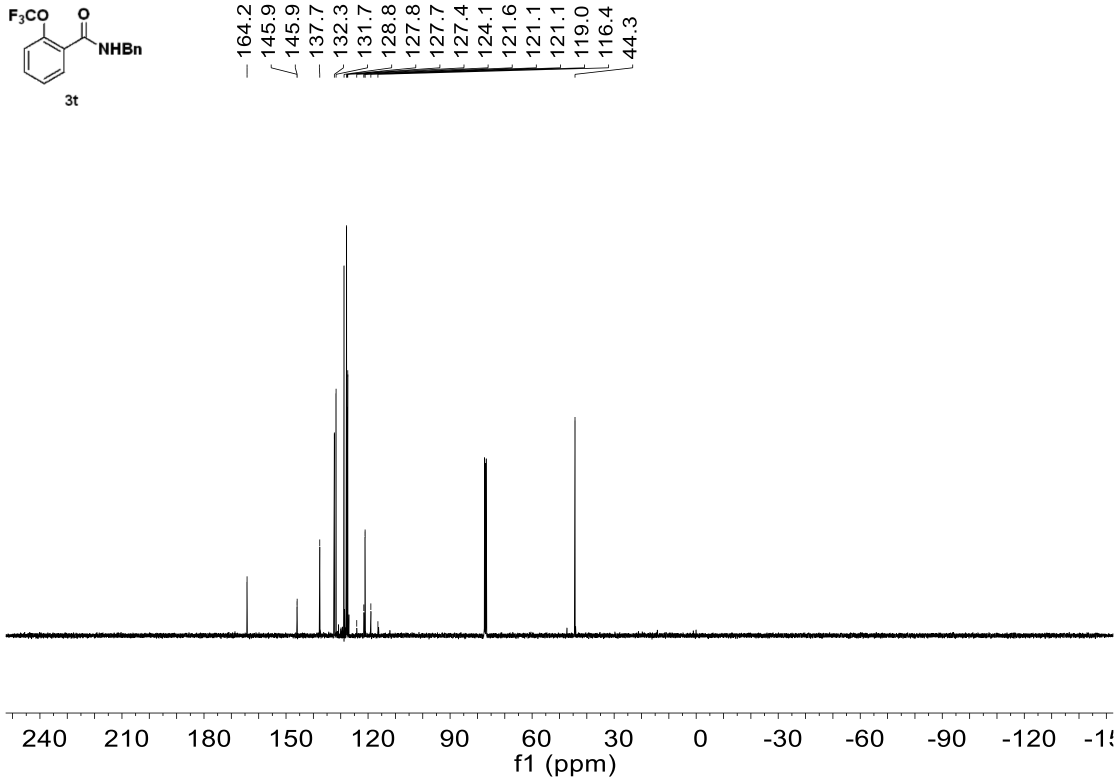

Supplementary Fig. 52. ^{13}C NMR spectrum (101 MHz, $CDCl_3$) of **3t**

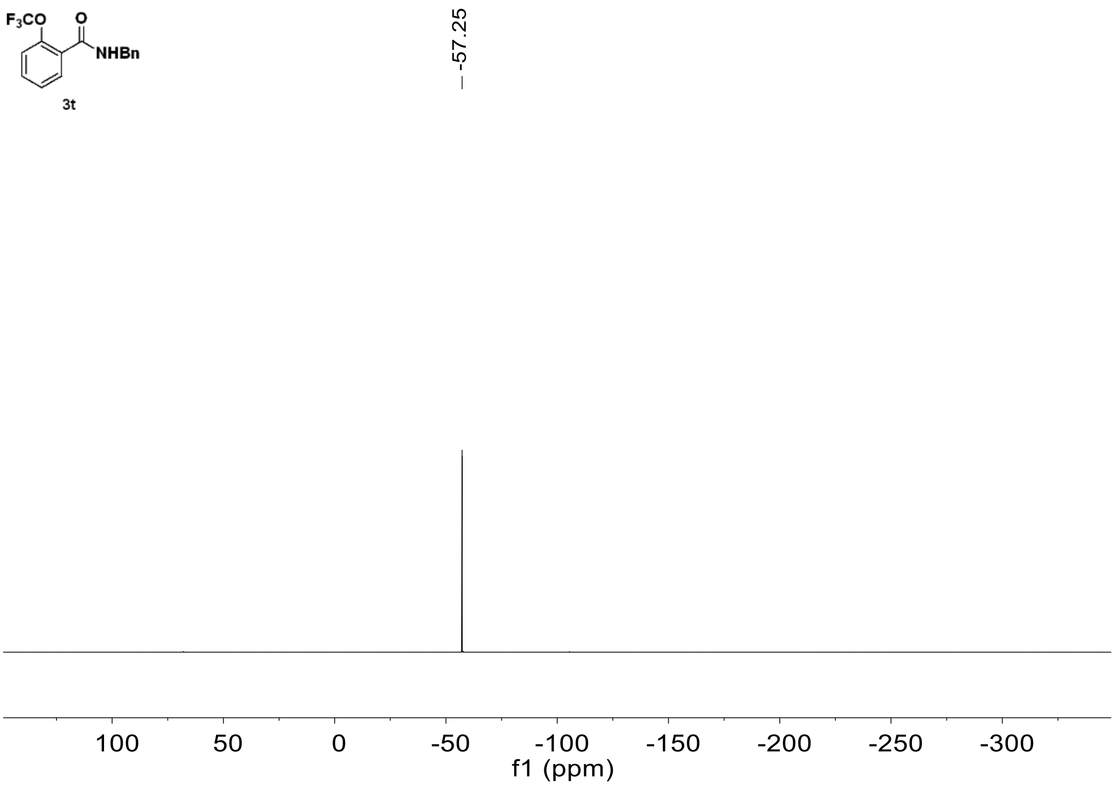

Supplementary Fig. 53. ^{19}F NMR spectrum (376 MHz, $CDCl_3$) of **3t**

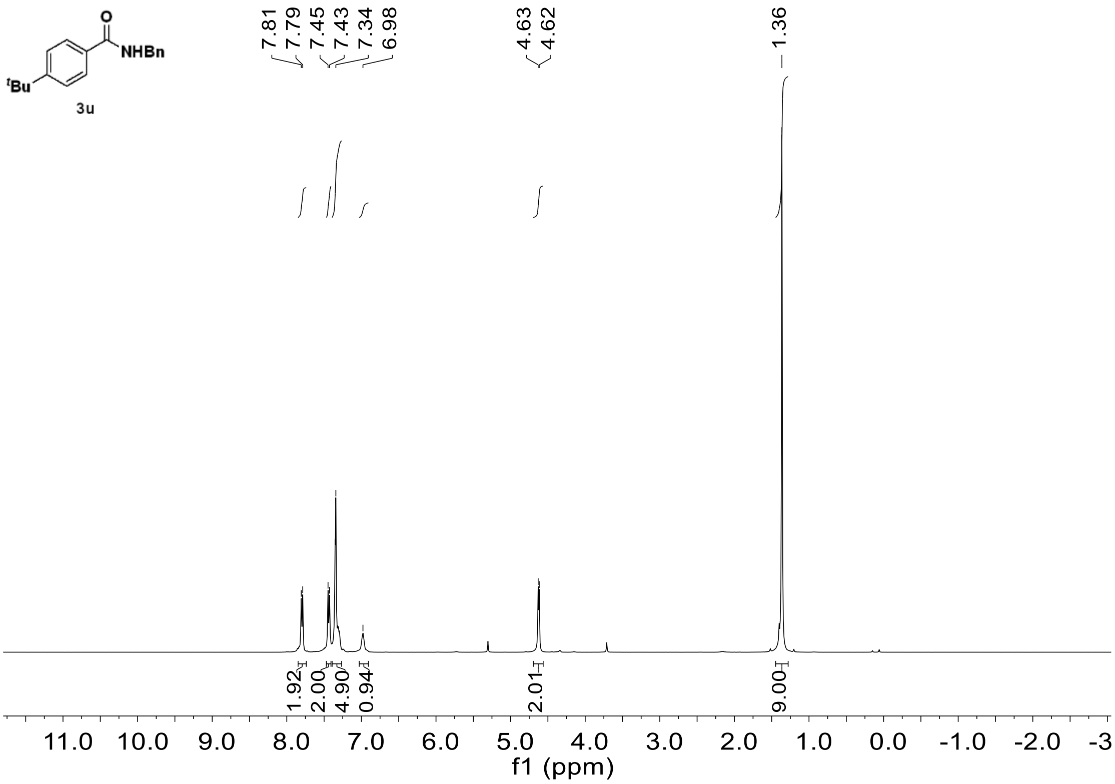

Supplementary Fig. 54. ^{1}H NMR spectrum (400 MHz, $CDCl_3$) of **3u**

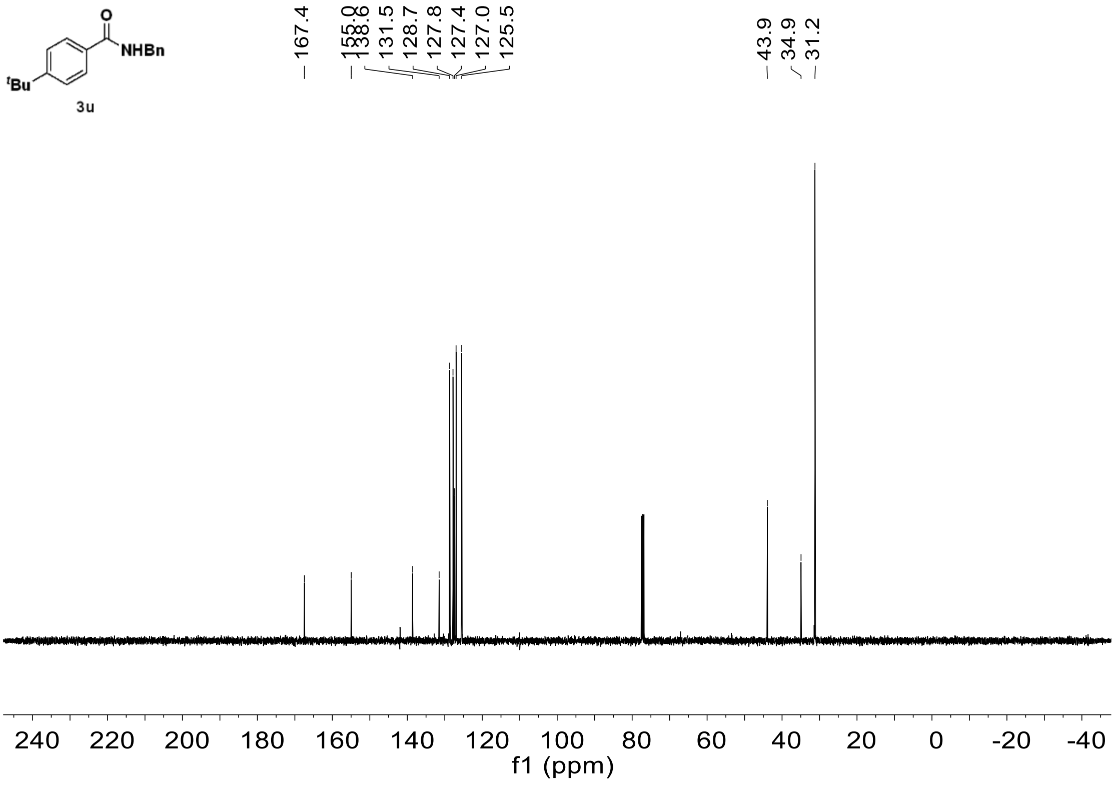

Supplementary Fig. 55. ^{13}C NMR spectrum (101 MHz, CDCl$_3$) of **3u**

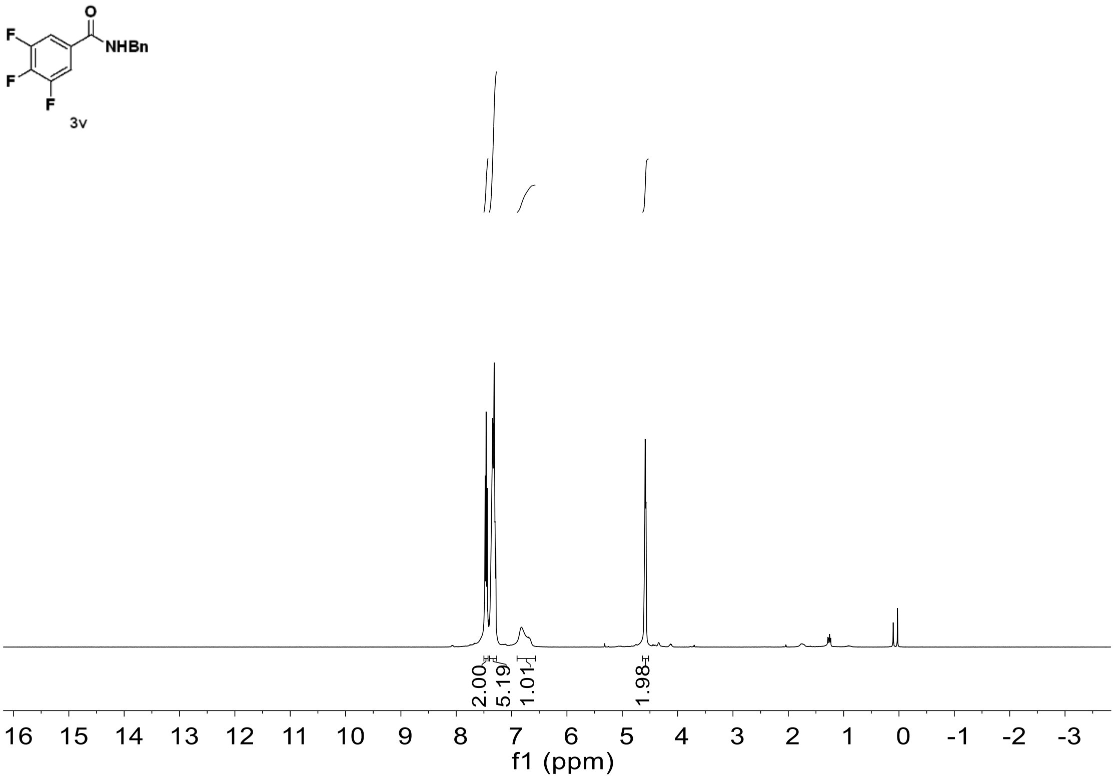

Supplementary Fig. 56. ^{1}H NMR spectrum (400 MHz, CDCl$_3$) of **3v**

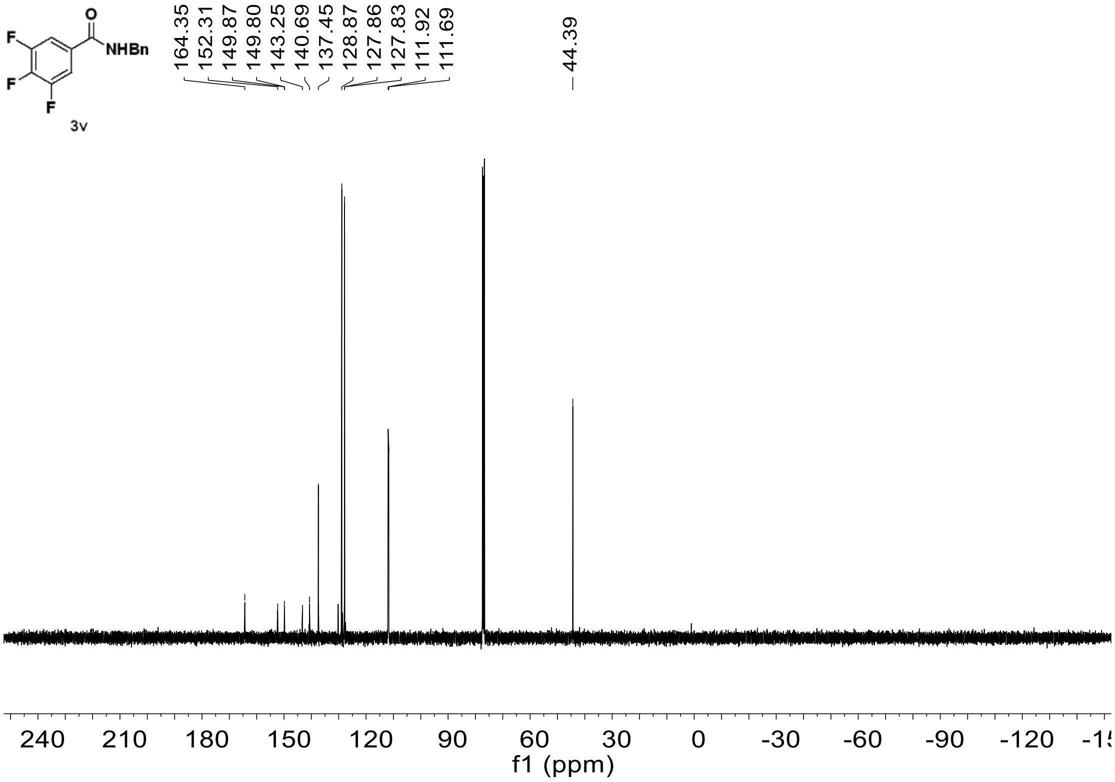

Supplementary Fig. 57. ^{13}C NMR spectrum (101 MHz, $CDCl_3$) of **3v**

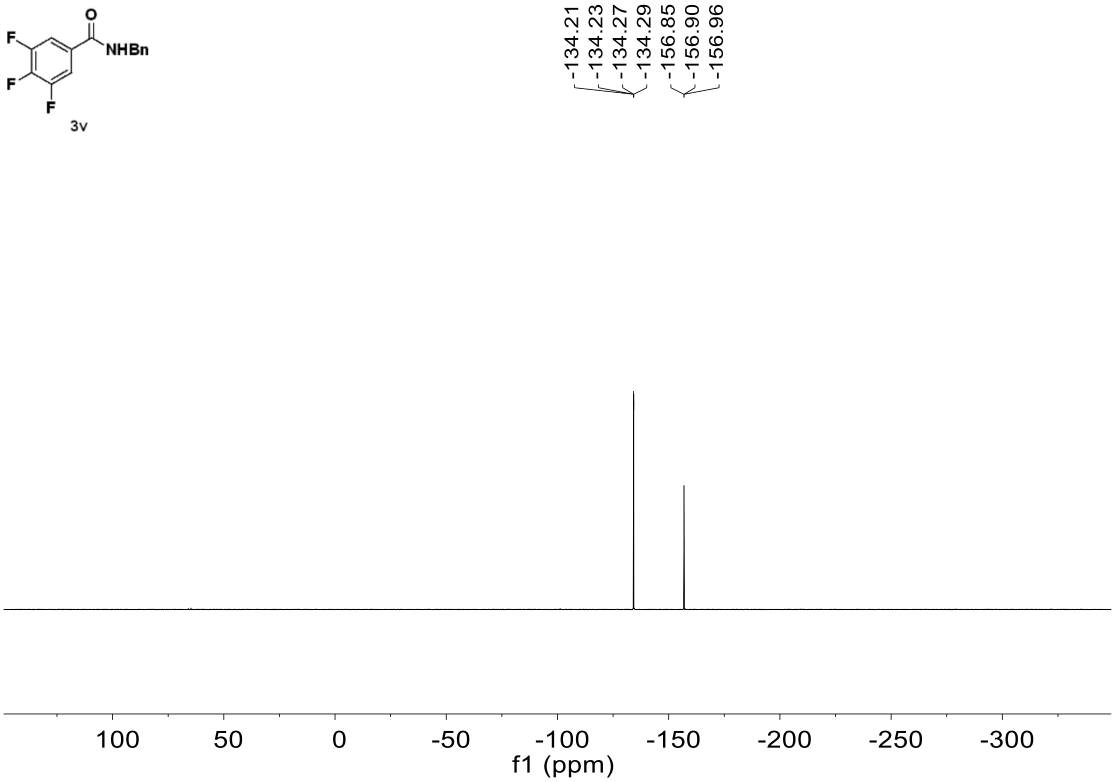

Supplementary Fig. 58. ^{19}F NMR spectrum (376 MHz, $CDCl_3$) of **3v**

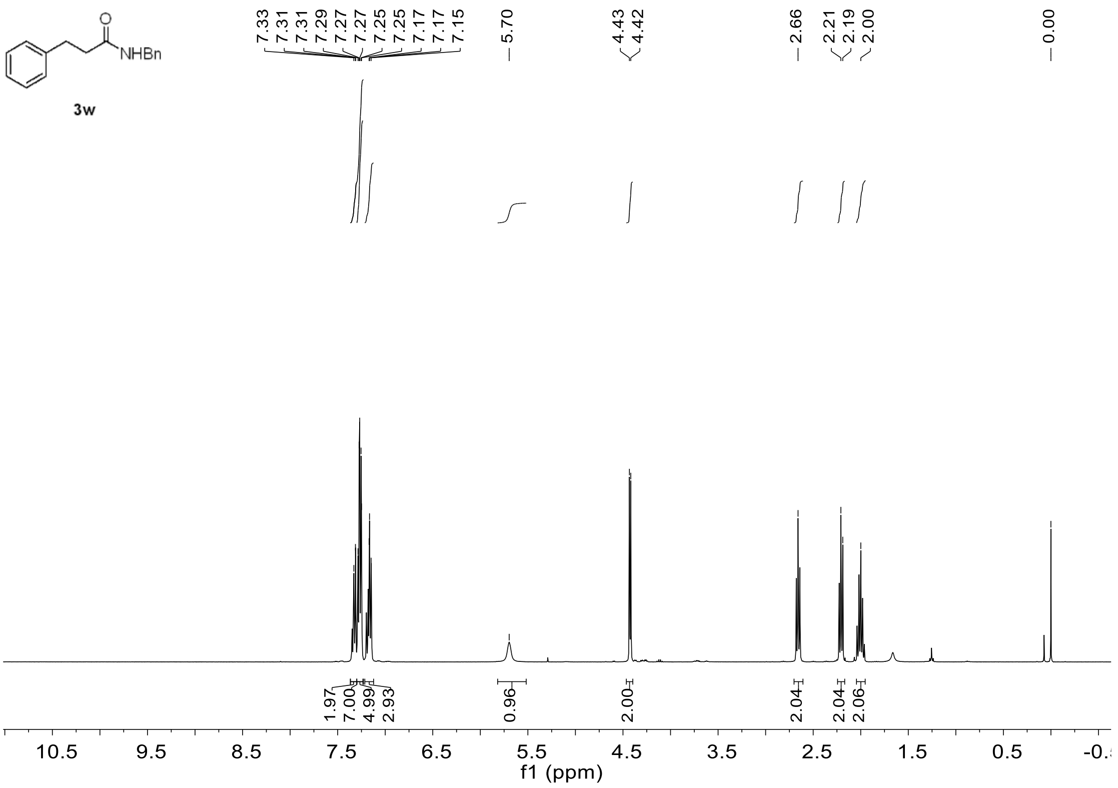

Supplementary Fig. 59. ^{1}H NMR spectrum (400 MHz, $CDCl_3$) of **3w**

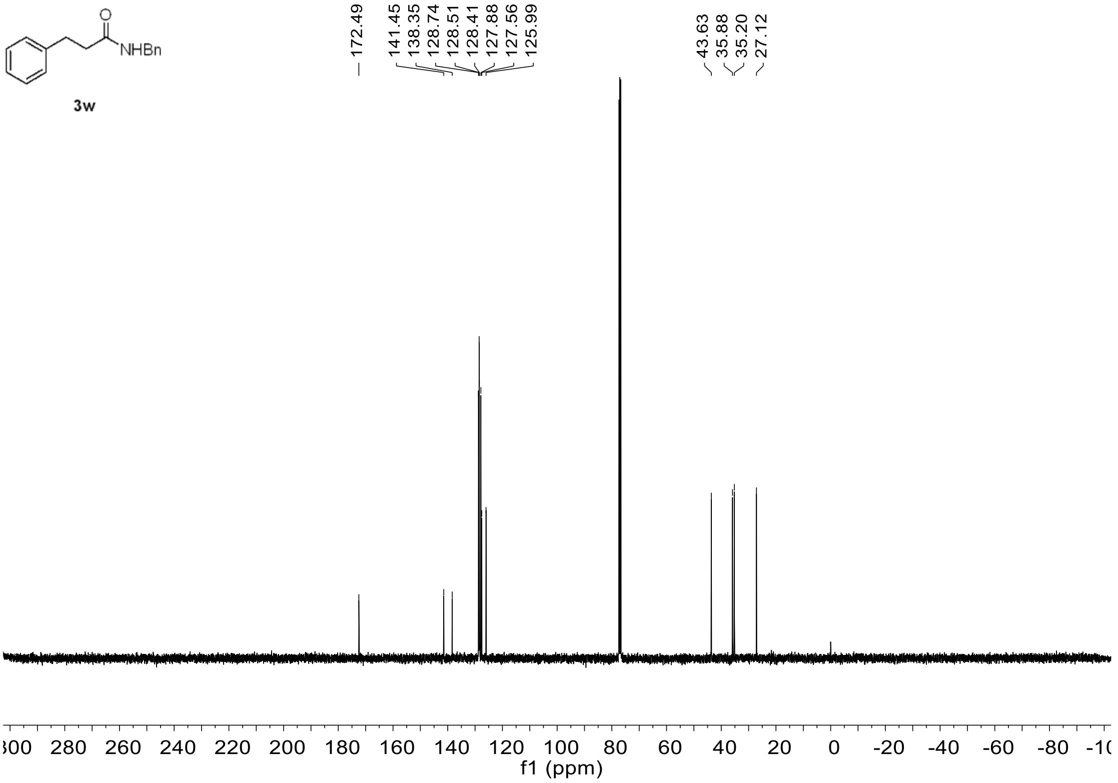

Supplementary Fig. 60. ^{13}C NMR spectrum (101 MHz, $CDCl_3$) of **3w**

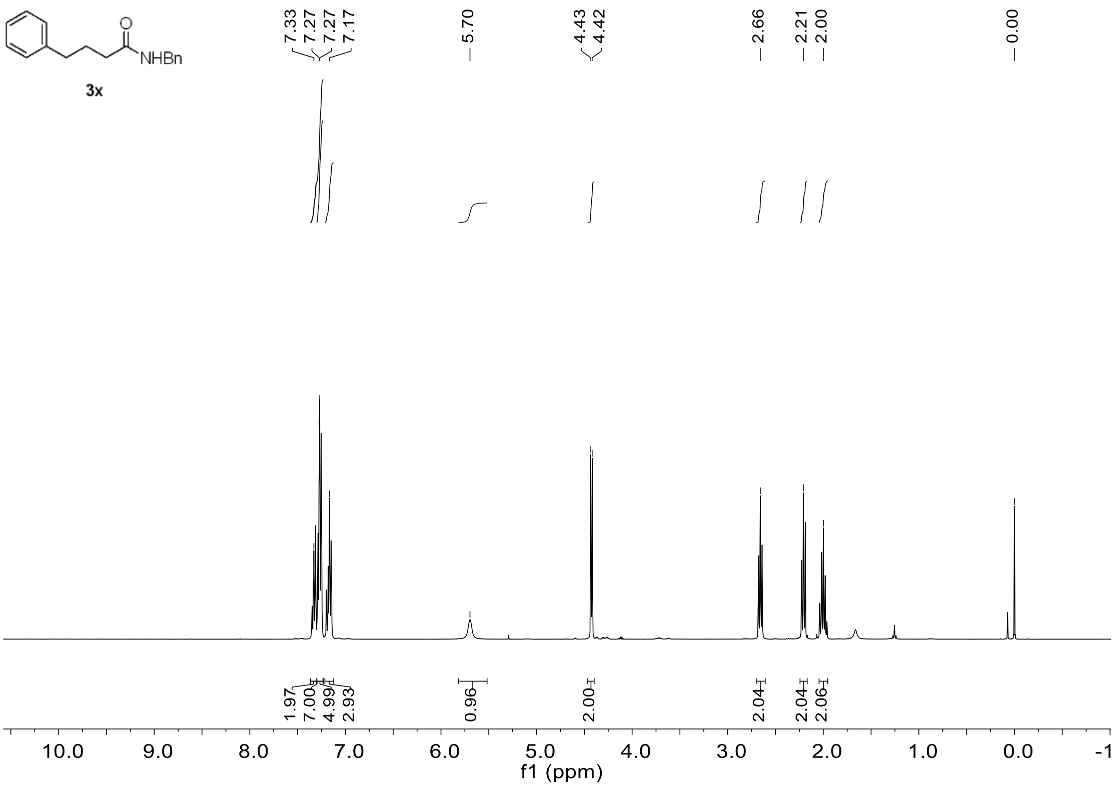

Supplementary Fig. 61. ^{1}H NMR spectrum (400 MHz, $CDCl_3$) of **3x**

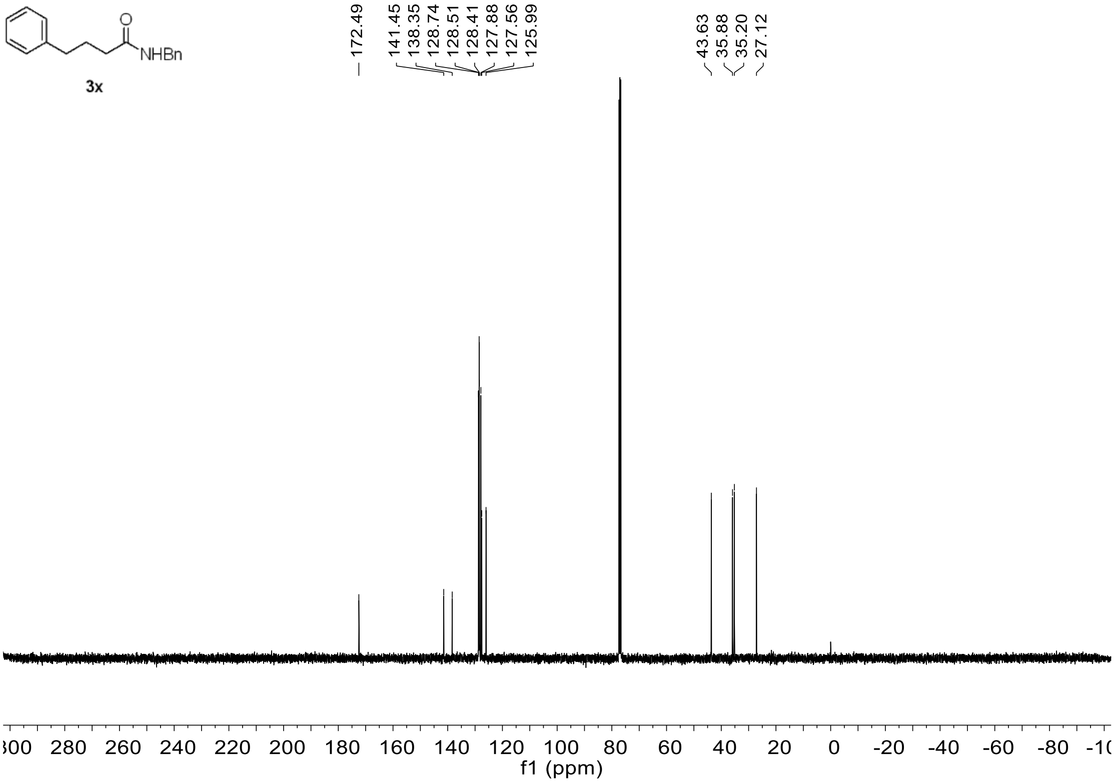

Supplementary Fig. 62. ^{13}C NMR spectrum (101 MHz, $CDCl_3$) of **3x**

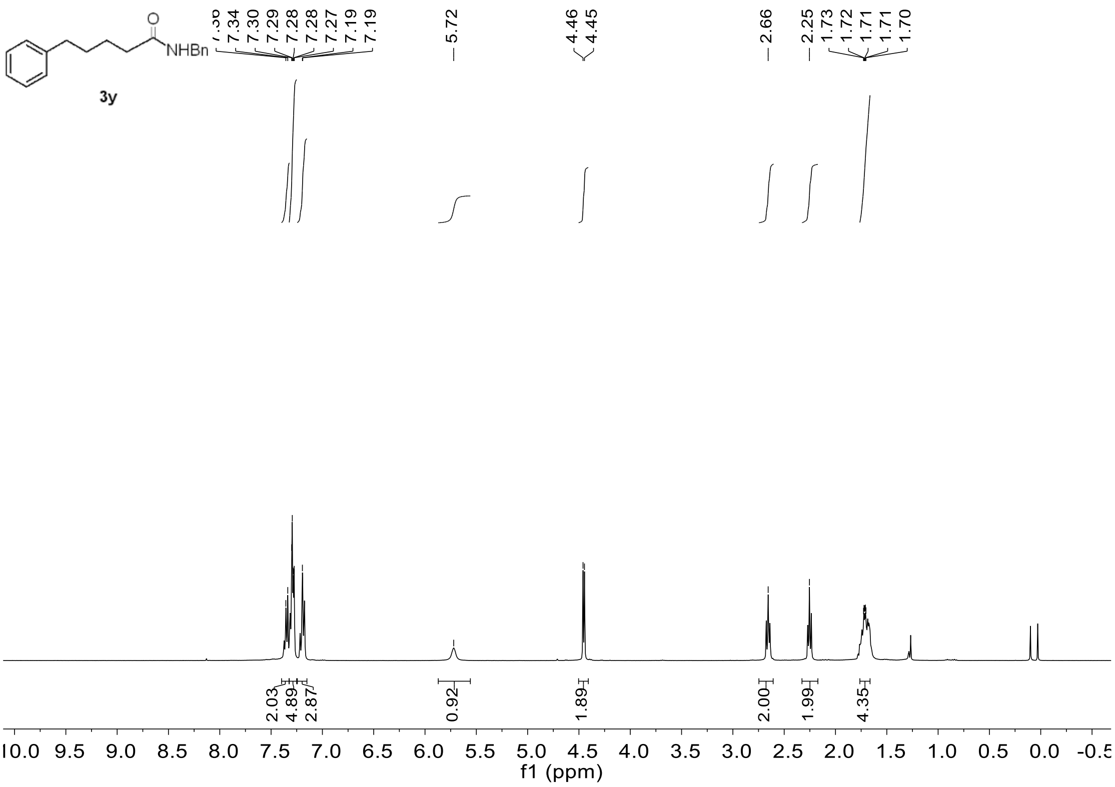

Supplementary Fig. 63. ^{1}H NMR spectrum (400 MHz, $CDCl_3$) of **3y**

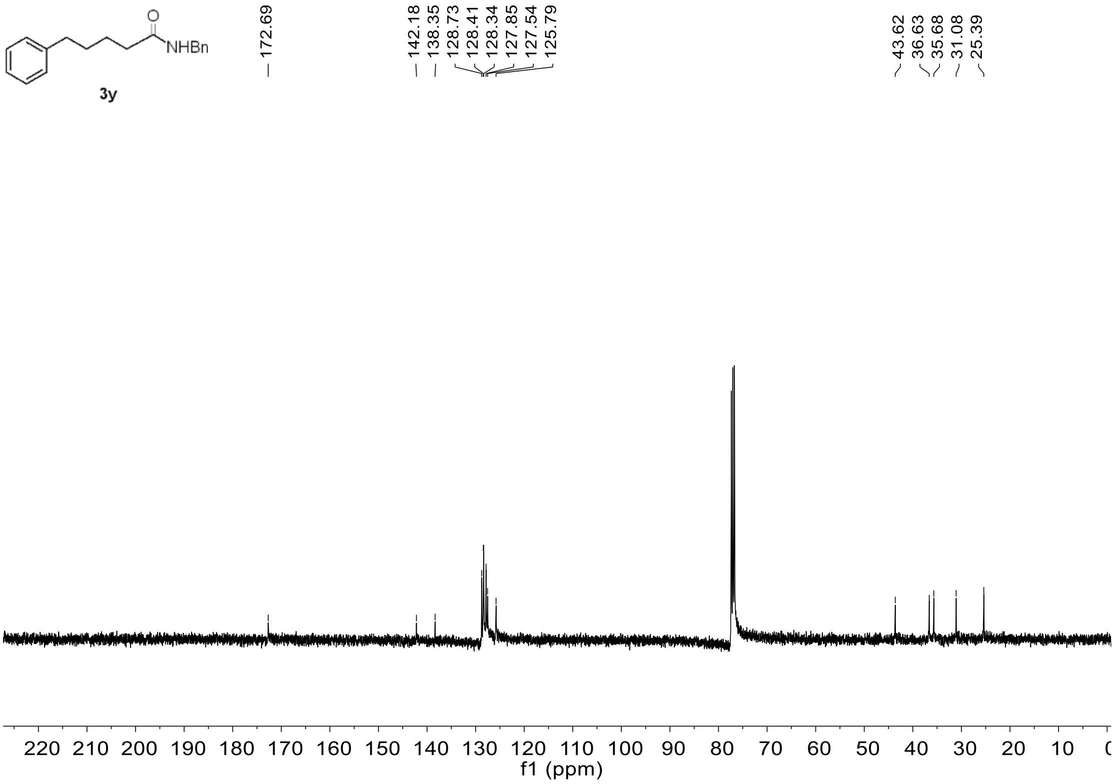

Supplementary Fig. 64. ^{13}C NMR spectrum (101 MHz, $CDCl_3$) of **3y**

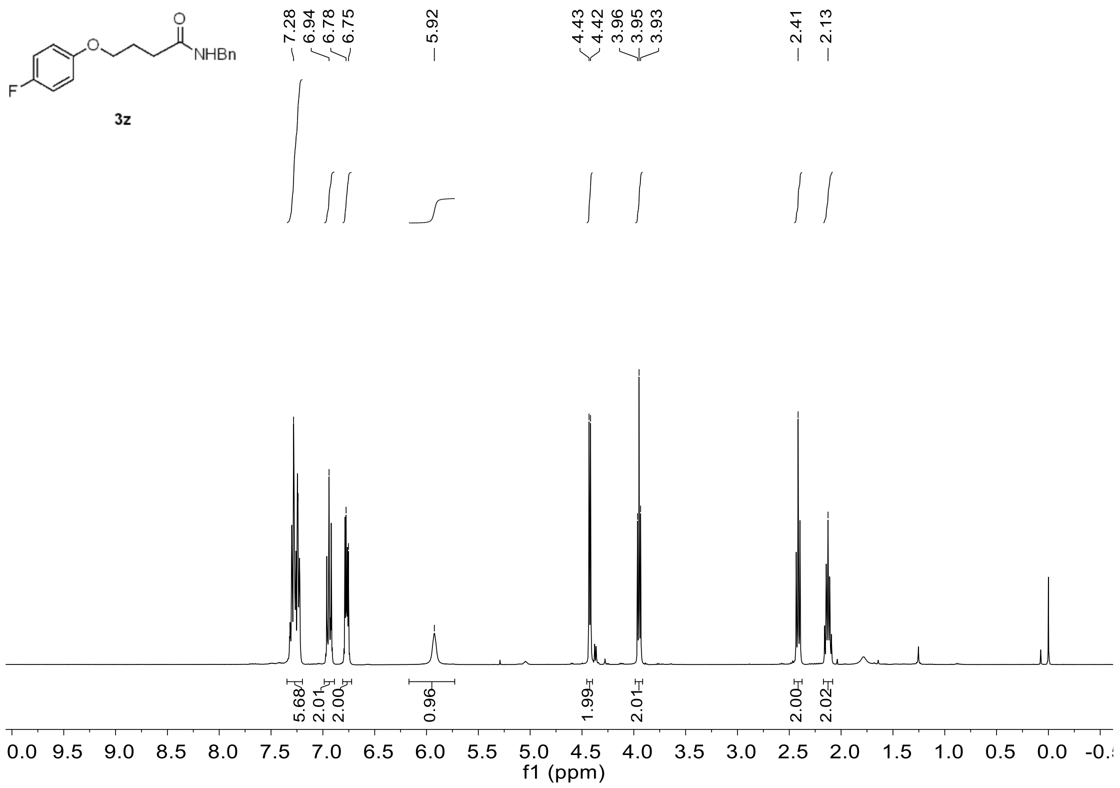

Supplementary Fig. 65. ^{1}H NMR spectrum (400 MHz, $CDCl_3$) of **3z**

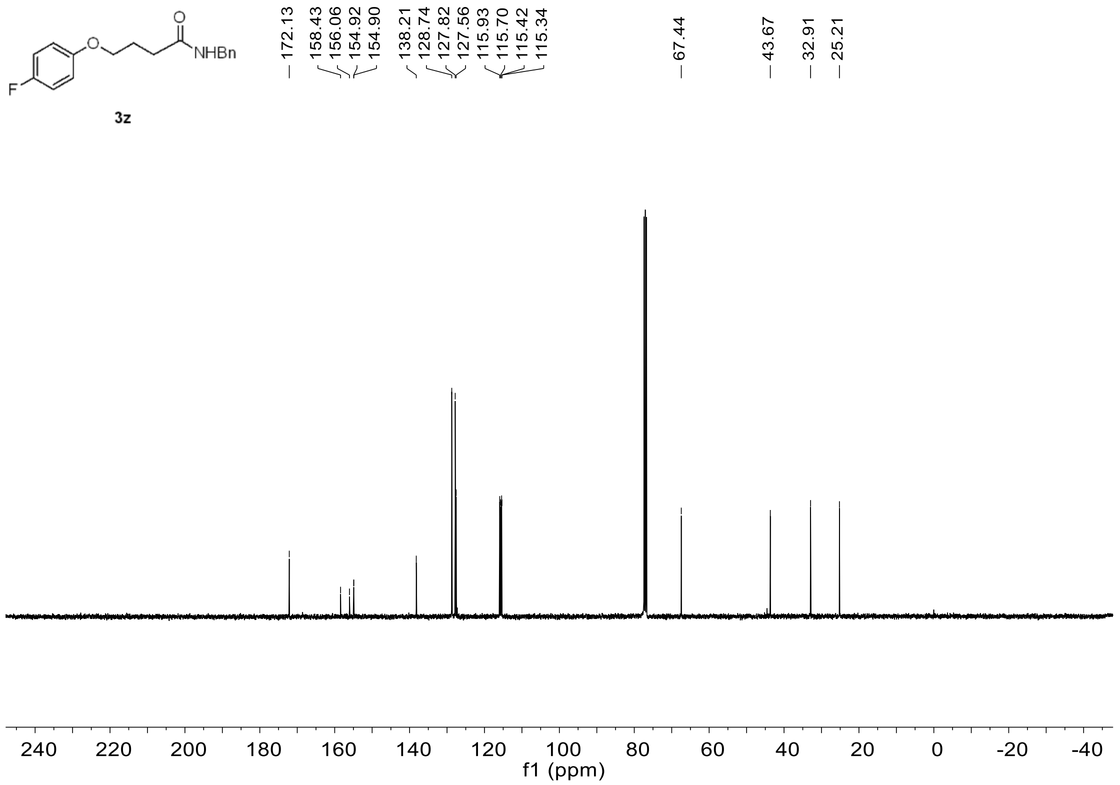

Supplementary Fig. 66. ^{13}C NMR spectrum (101 MHz, $CDCl_3$) of **3z**

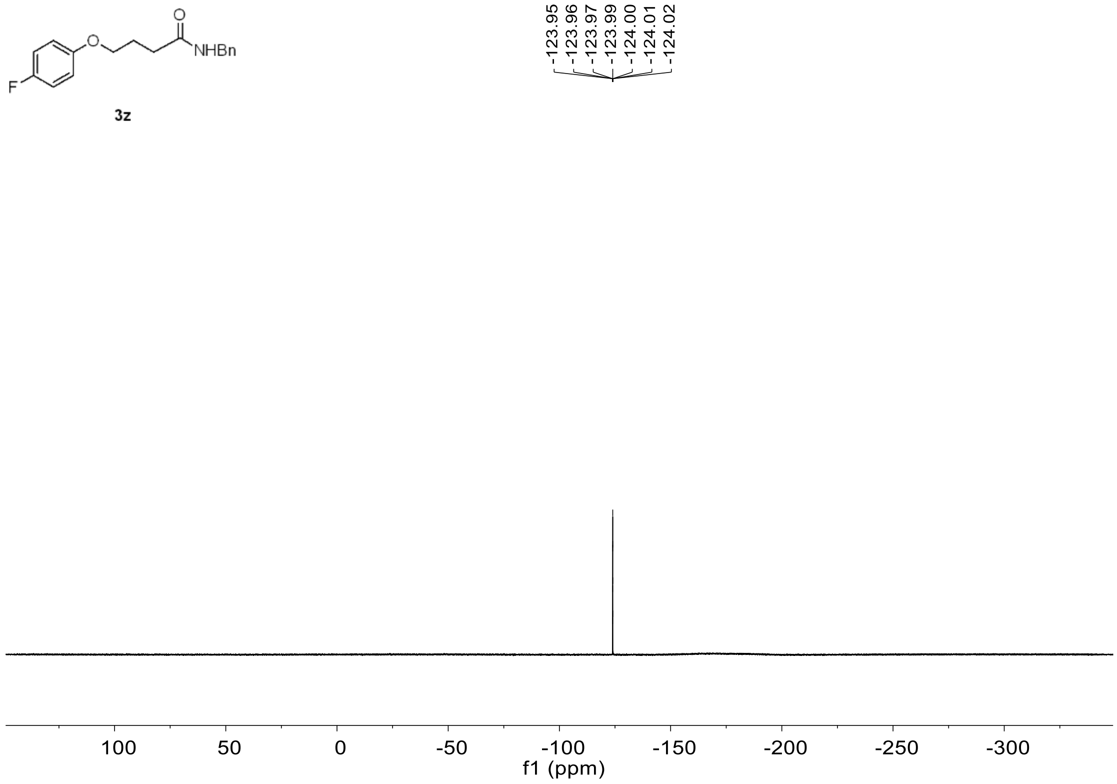

Supplementary Fig. 67. ^{19}F NMR spectrum (376 MHz, $CDCl_3$) of **3z**

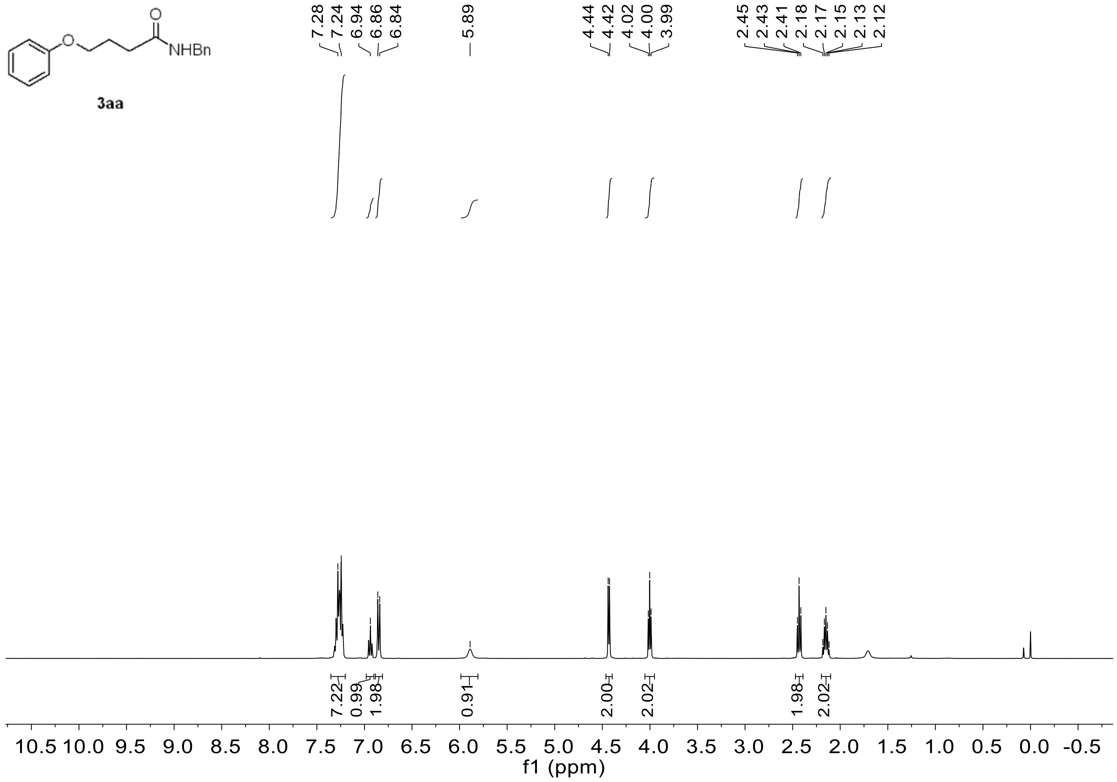

Supplementary Fig. 68. ^{1}H NMR spectrum (400 MHz, $CDCl_3$) of **3aa**

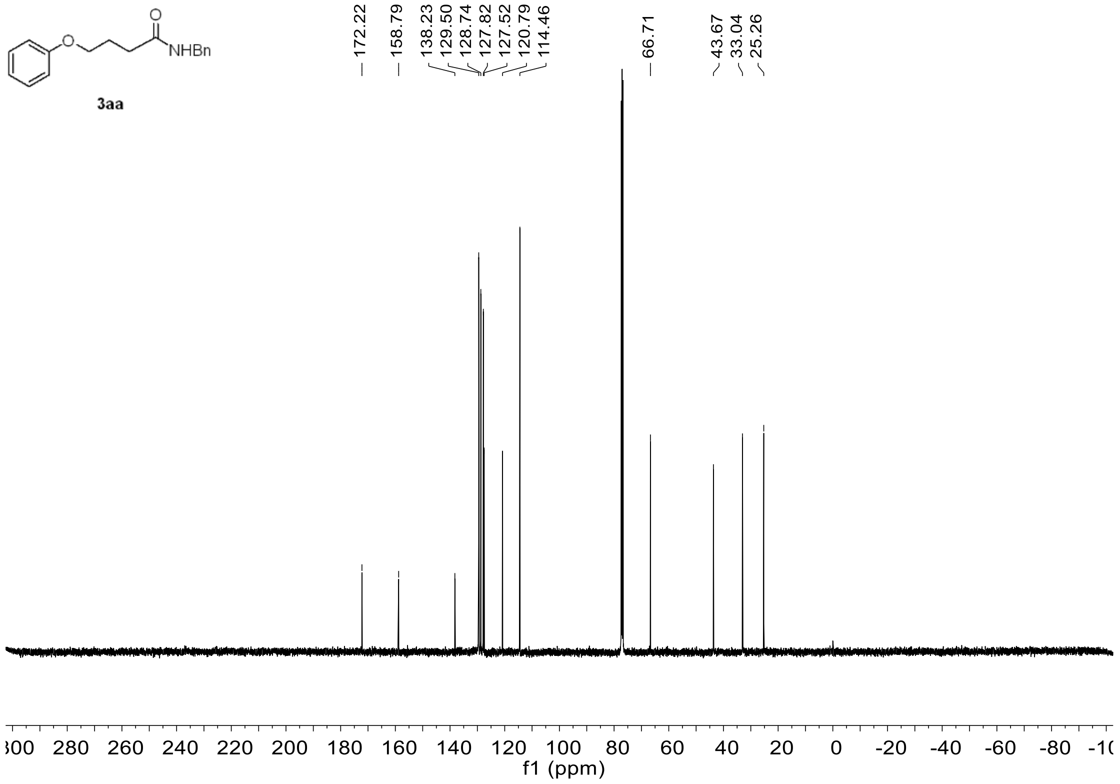

Supplementary Fig. 69. ^{13}C NMR spectrum (101 MHz, $CDCl_3$) of **3aa**

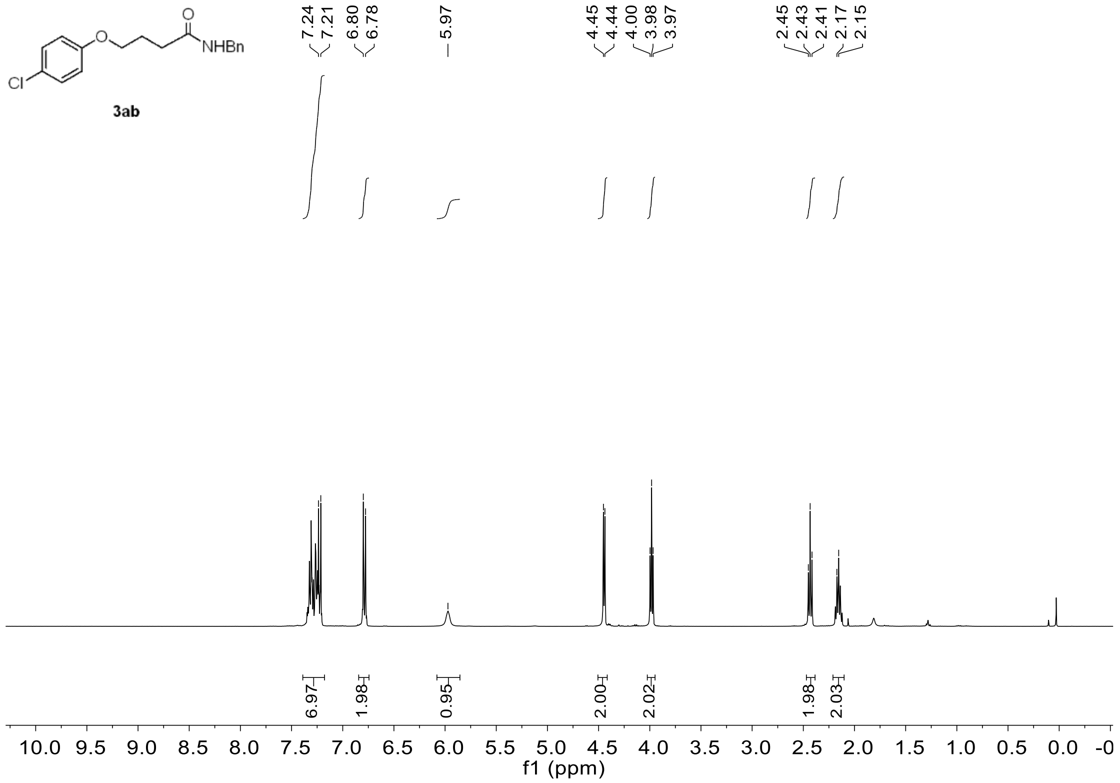

Supplementary Fig. 70. ^{1}H NMR spectrum (400 MHz, $CDCl_3$) of **3ab**

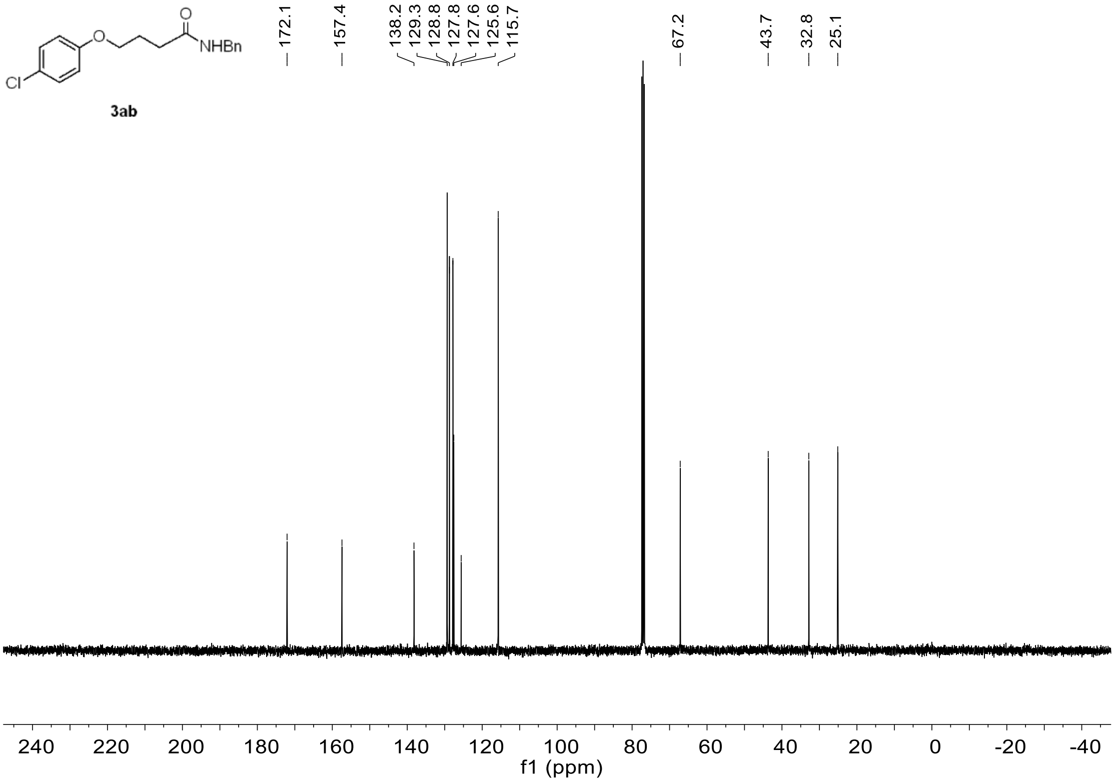

Supplementary Fig. 71. ^{13}C NMR spectrum (101 MHz, $CDCl_3$) of **3ab**

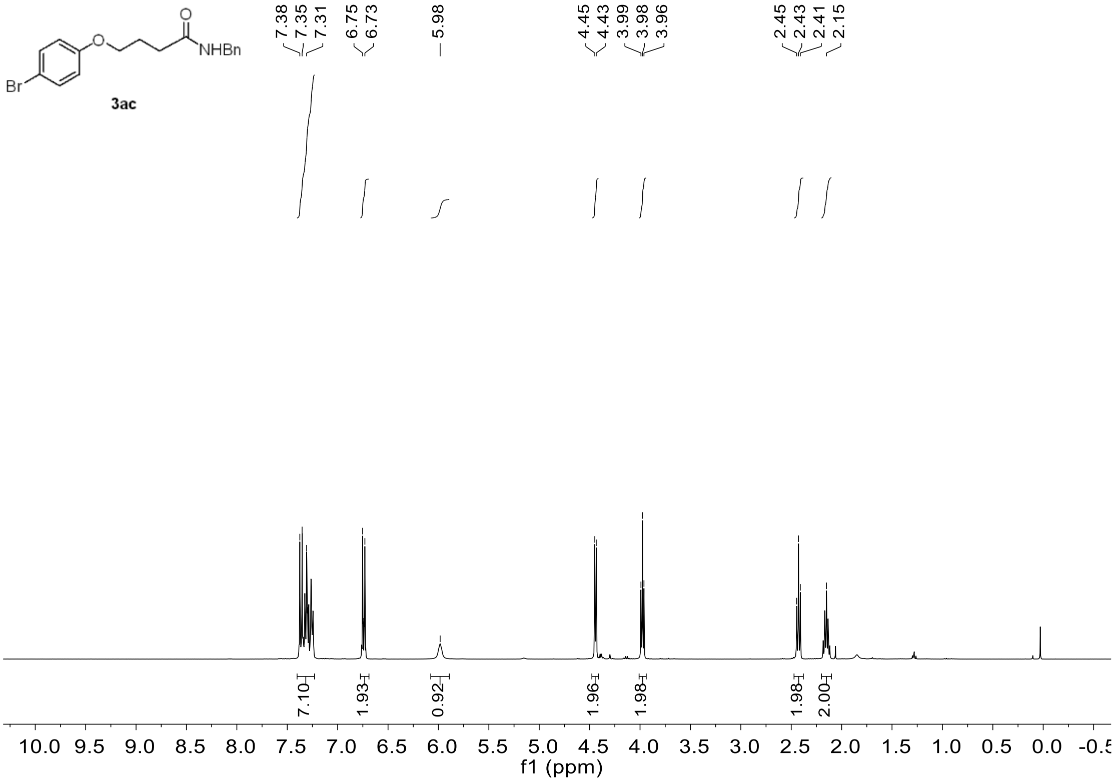

Supplementary Fig. 72. ^{1}H NMR spectrum (400 MHz, $CDCl_3$) of **3ac**

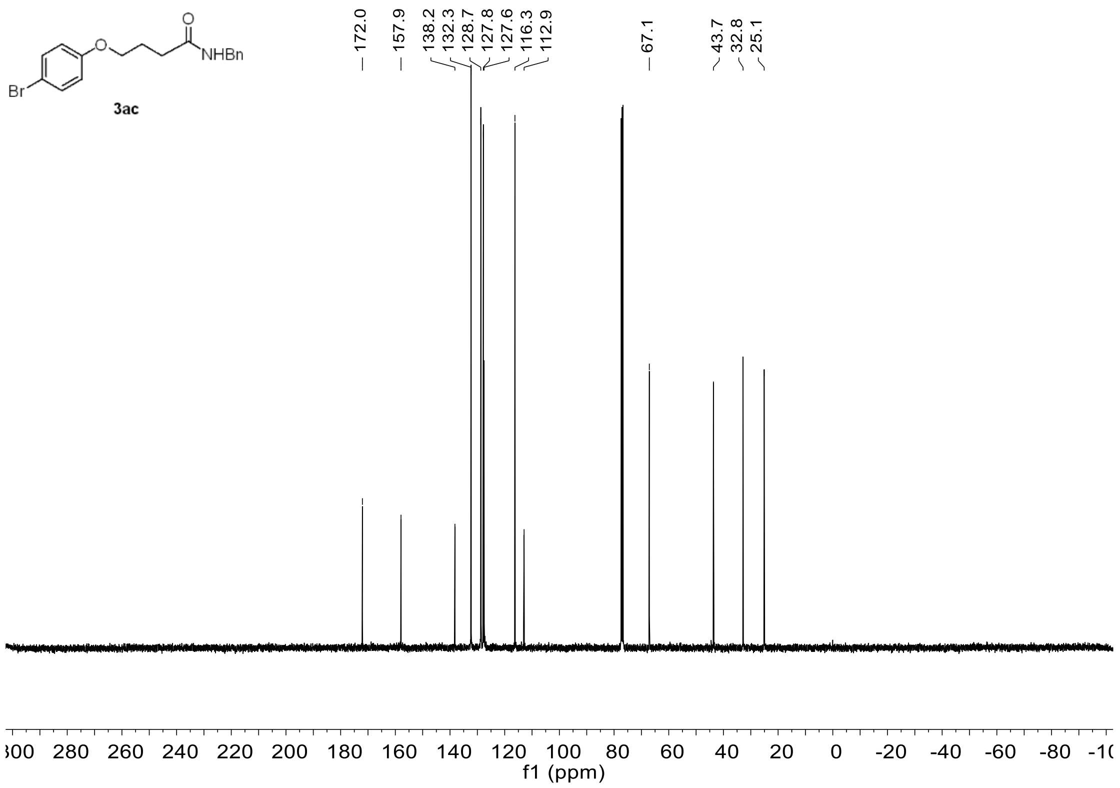

Supplementary Fig. 73. ^{13}C NMR spectrum (101 MHz, $CDCl_3$) of **3ac**

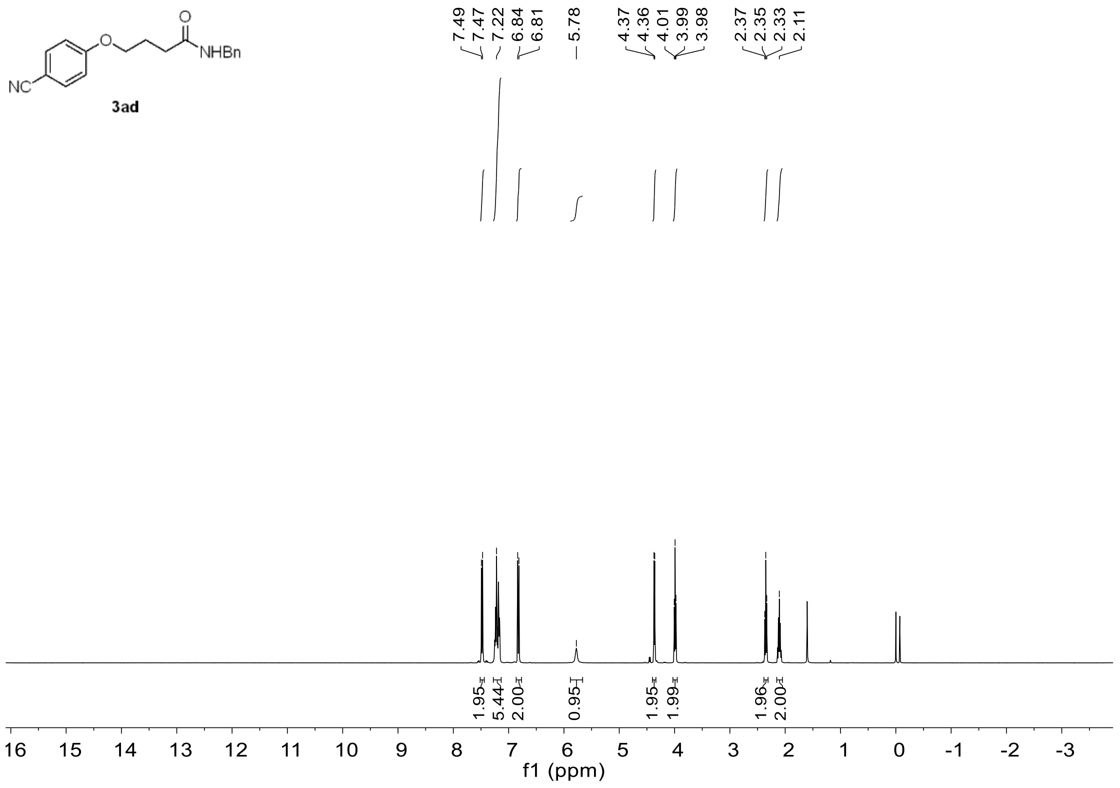

Supplementary Fig. 74. ^{1}H NMR spectrum (400 MHz, $CDCl_3$) of **3ad**

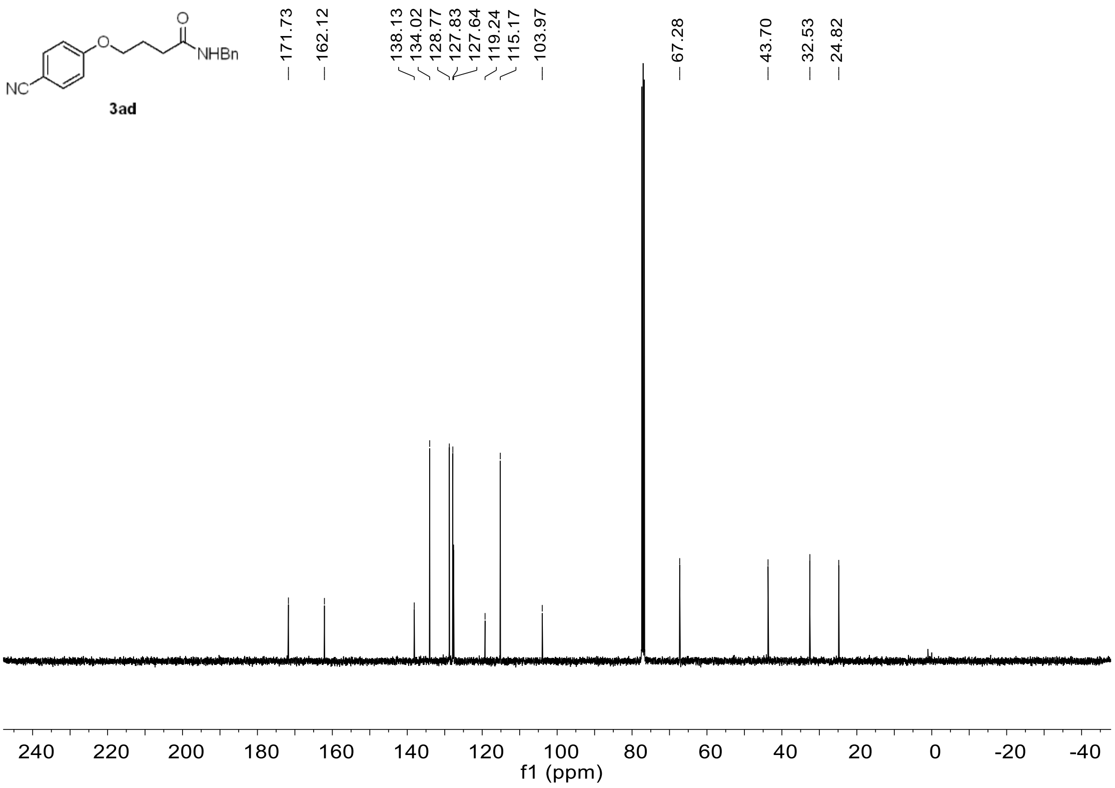

Supplementary Fig. 75. ^{13}C NMR spectrum (101 MHz, $CDCl_3$) of **3ad**

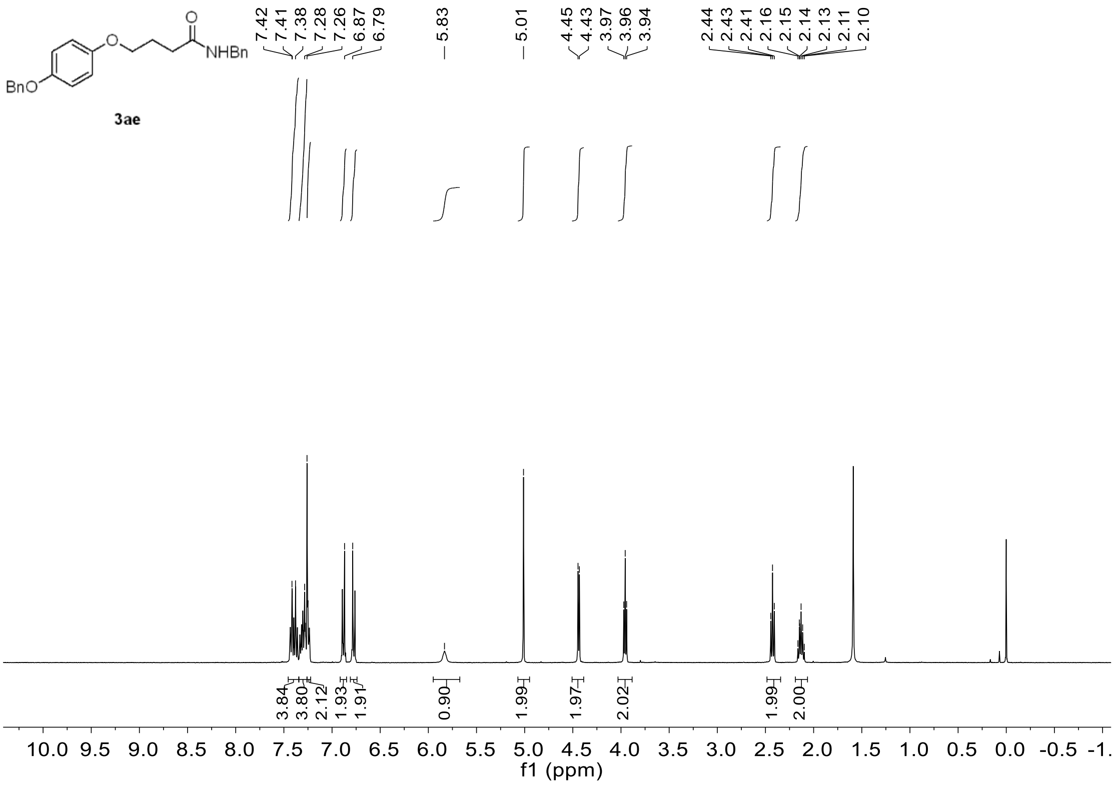

Supplementary Fig. 76. ^{1}H NMR spectrum (400 MHz, $CDCl_3$) of **3ae**

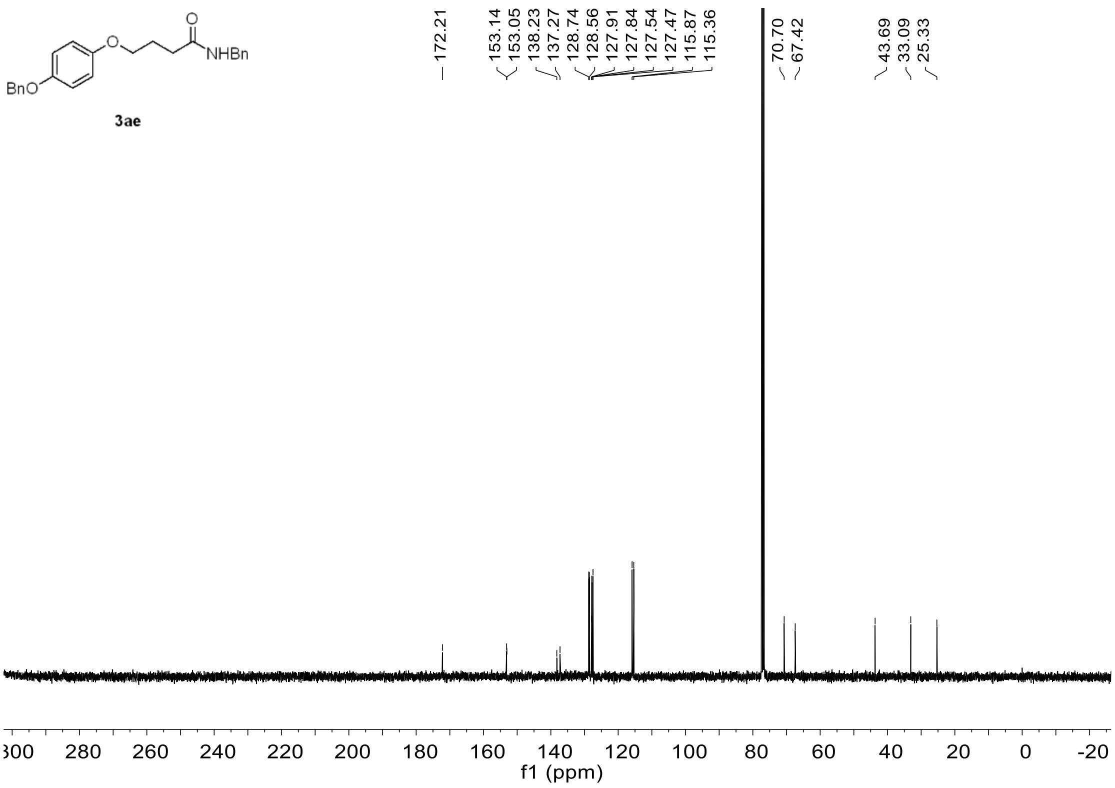

Supplementary Fig. 77. ^{13}C NMR spectrum (101 MHz, $CDCl_3$) of **3ae**

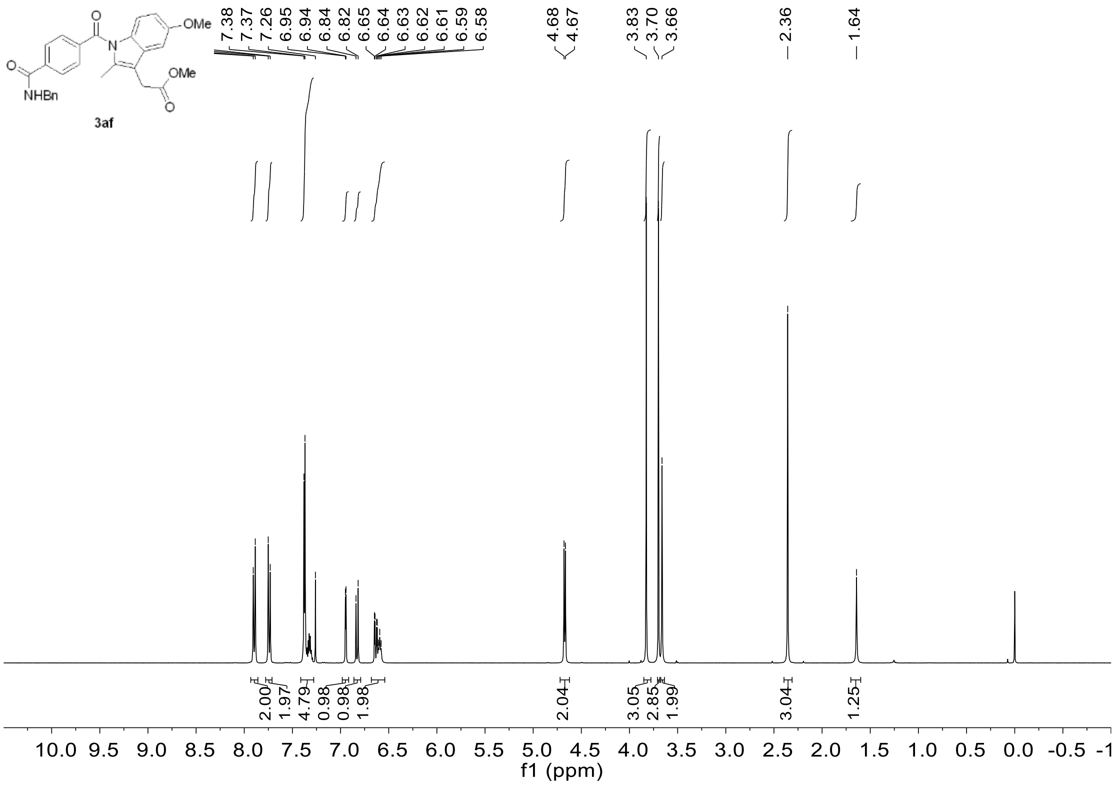

Supplementary Fig. 78. ^{1}H NMR spectrum (400 MHz, $CDCl_3$) of **3af**

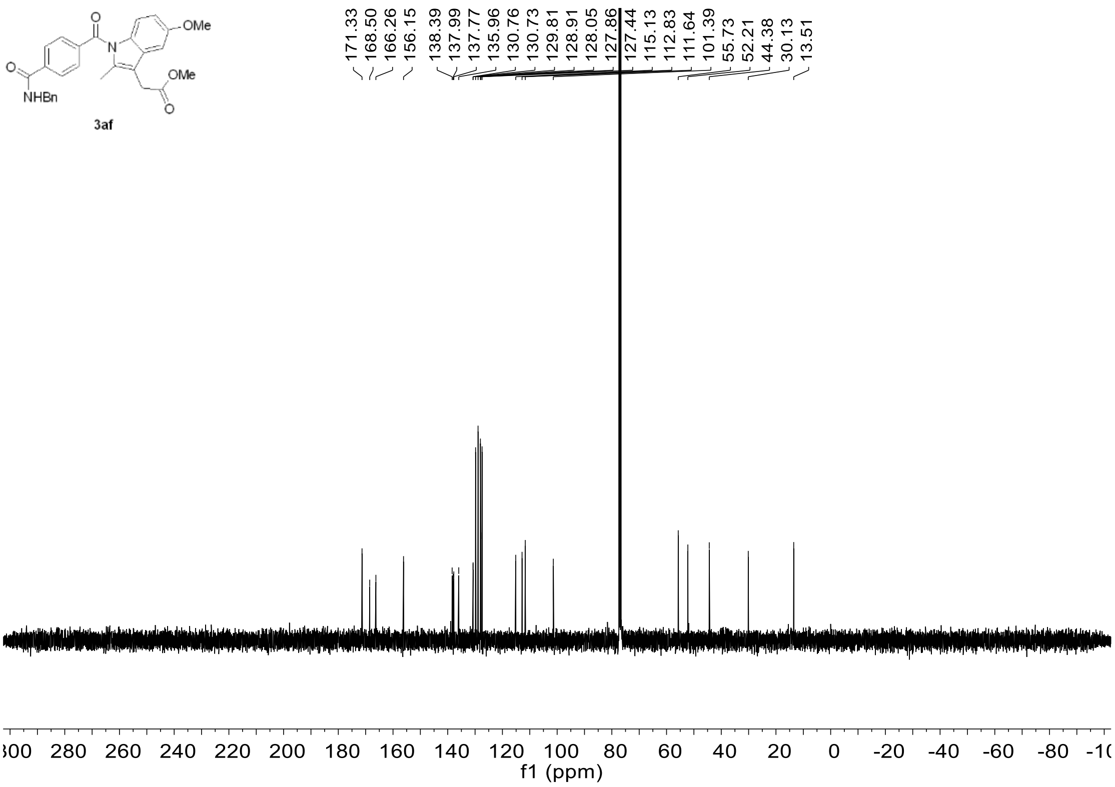

Supplementary Fig. 79. ^{13}C NMR spectrum (101 MHz, $CDCl_3$) of **3af**

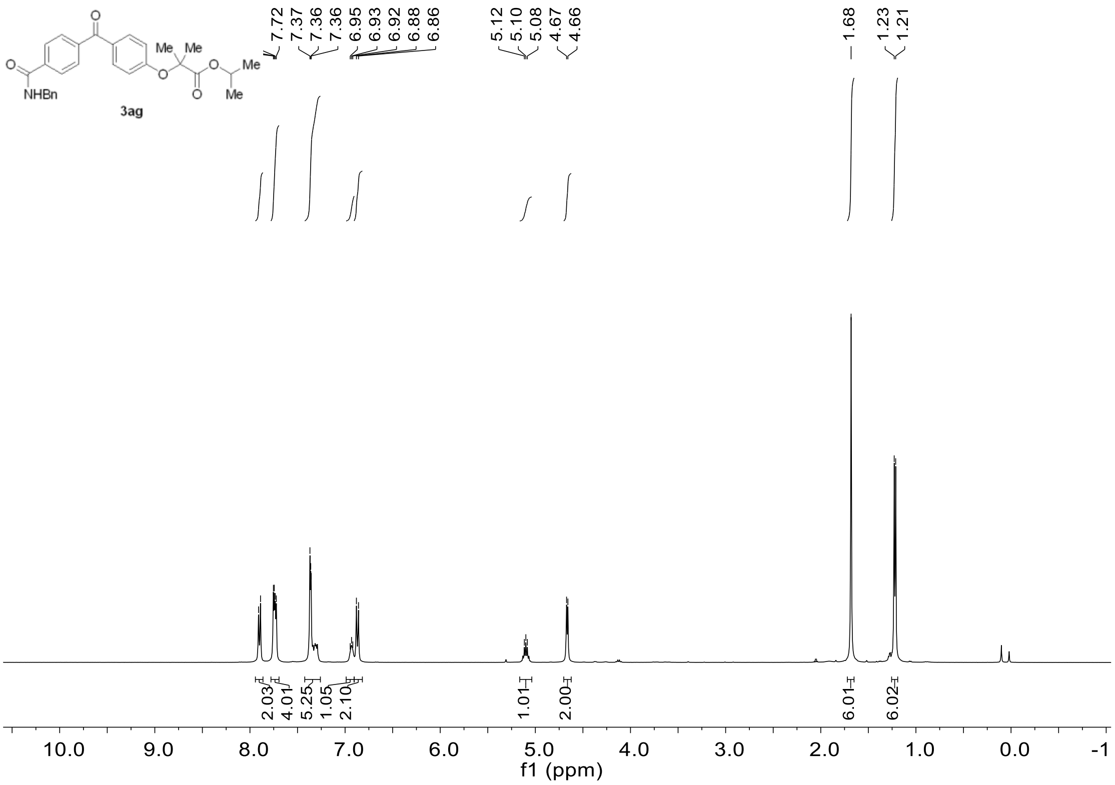

Supplementary Fig. 80. ^{1}H NMR spectrum (400 MHz, $CDCl_3$) of **3ag**

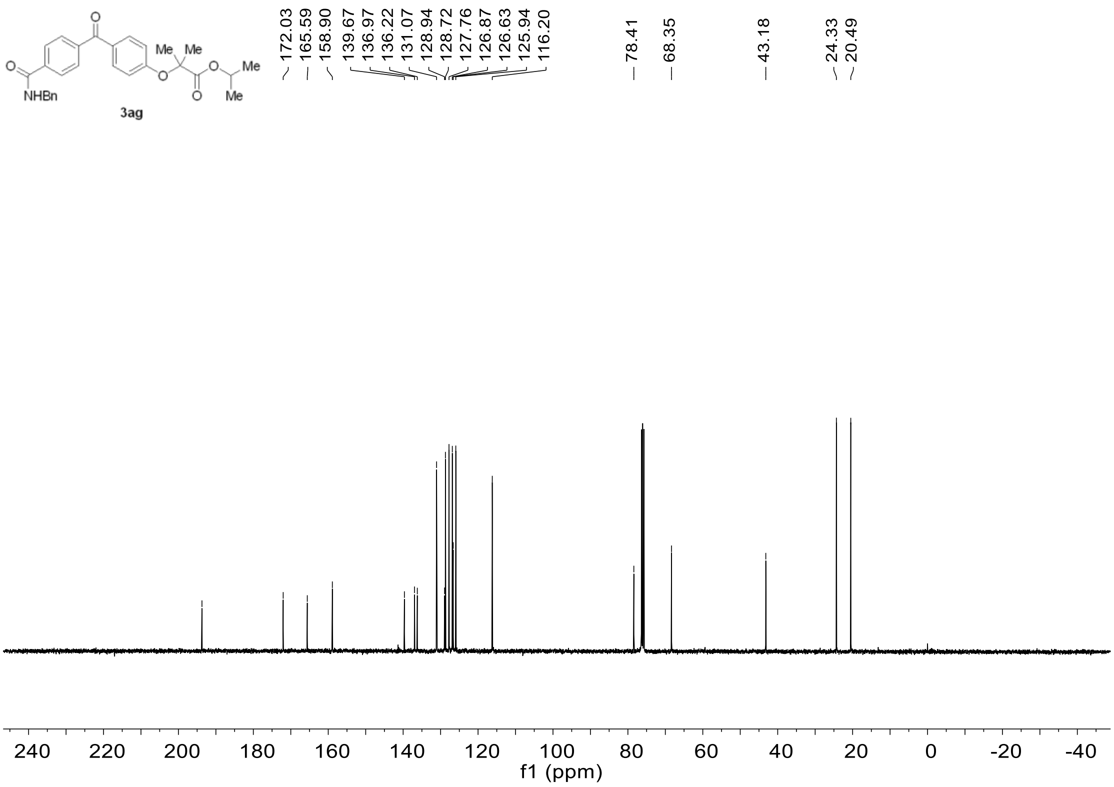

Supplementary Fig. 81. ^{13}C NMR spectrum (101 MHz, $CDCl_3$) of **3ag**

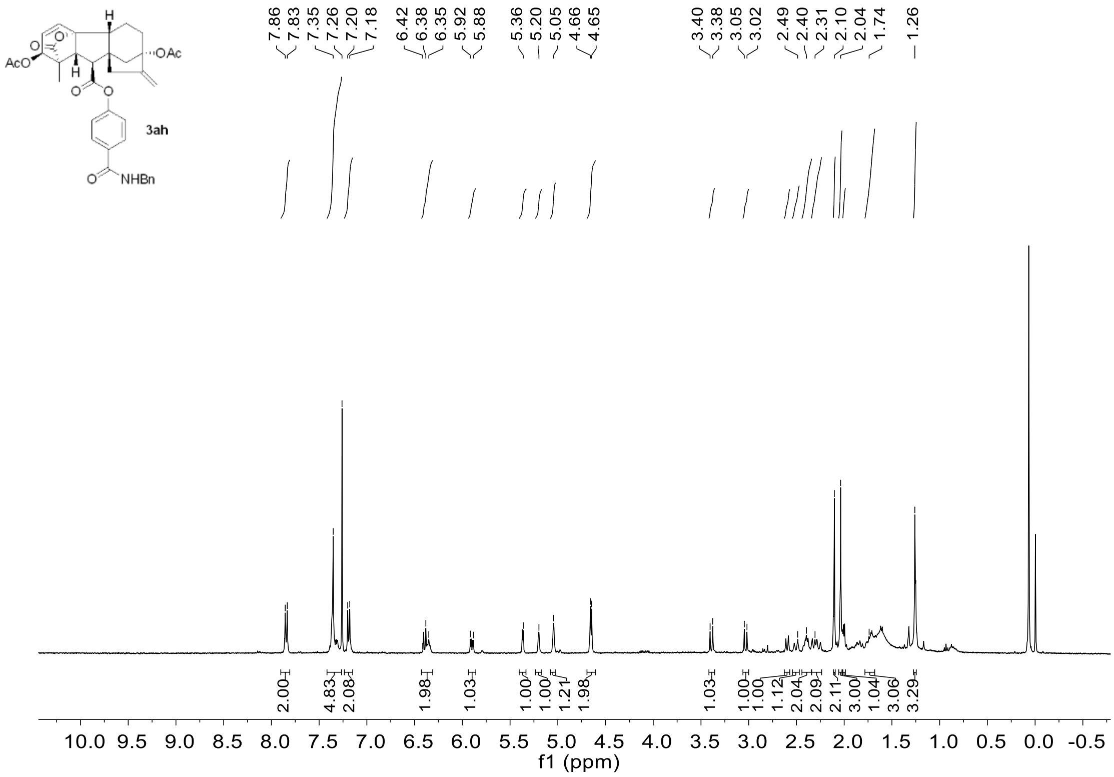

Supplementary Fig. 82. ^{1}H NMR spectrum (400 MHz, $CDCl_3$) of **3ah**

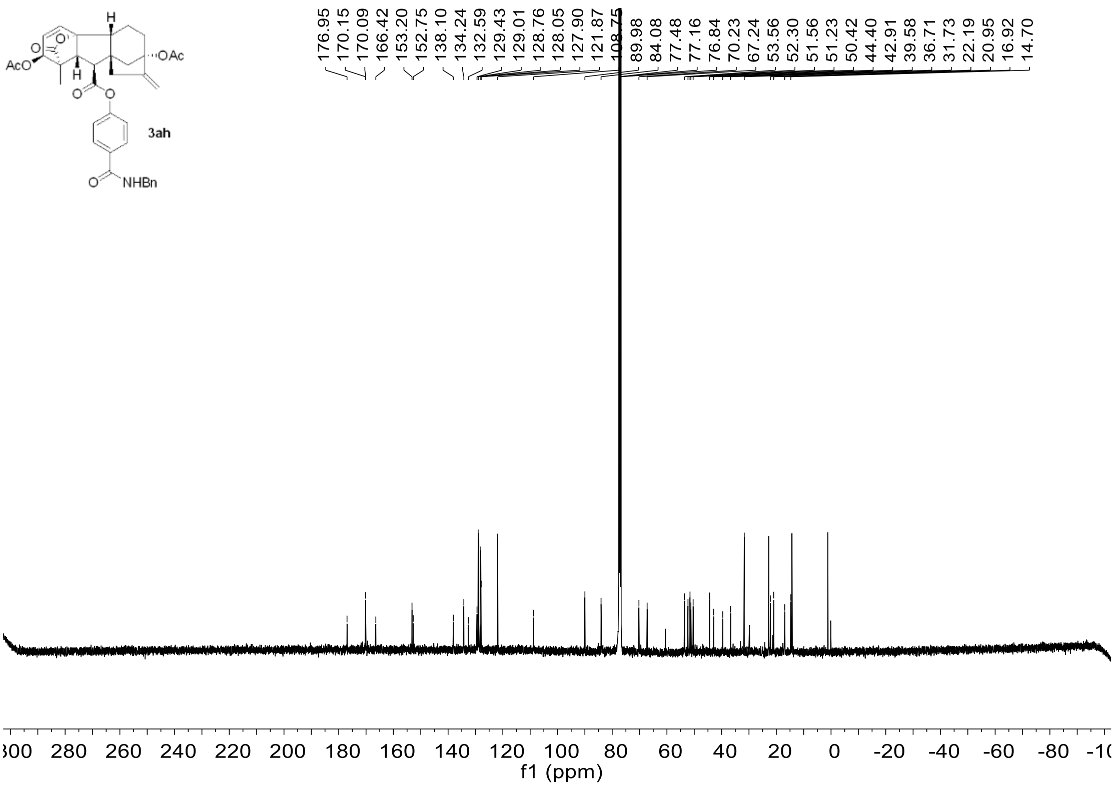

Supplementary Fig. 83. ^{13}C NMR spectrum (101 MHz, $CDCl_3$) of **3ah**

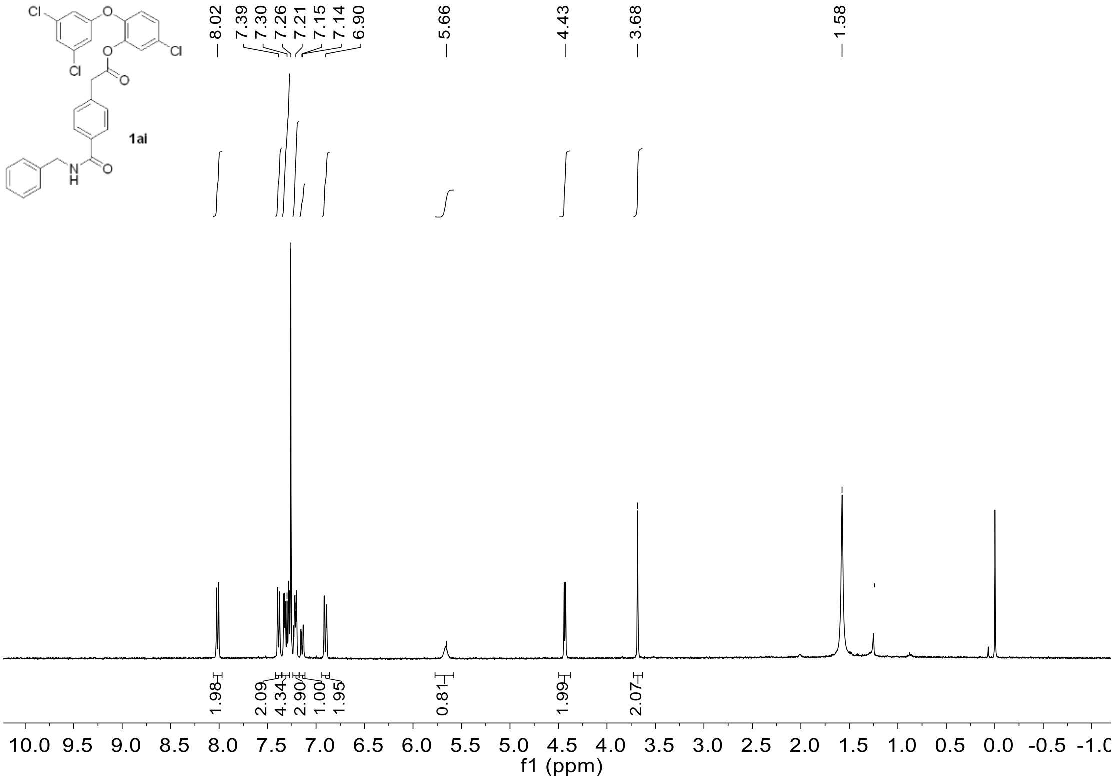

Supplementary Fig. 84. ^{1}H NMR spectrum (400 MHz, $CDCl_3$) of **3ai**

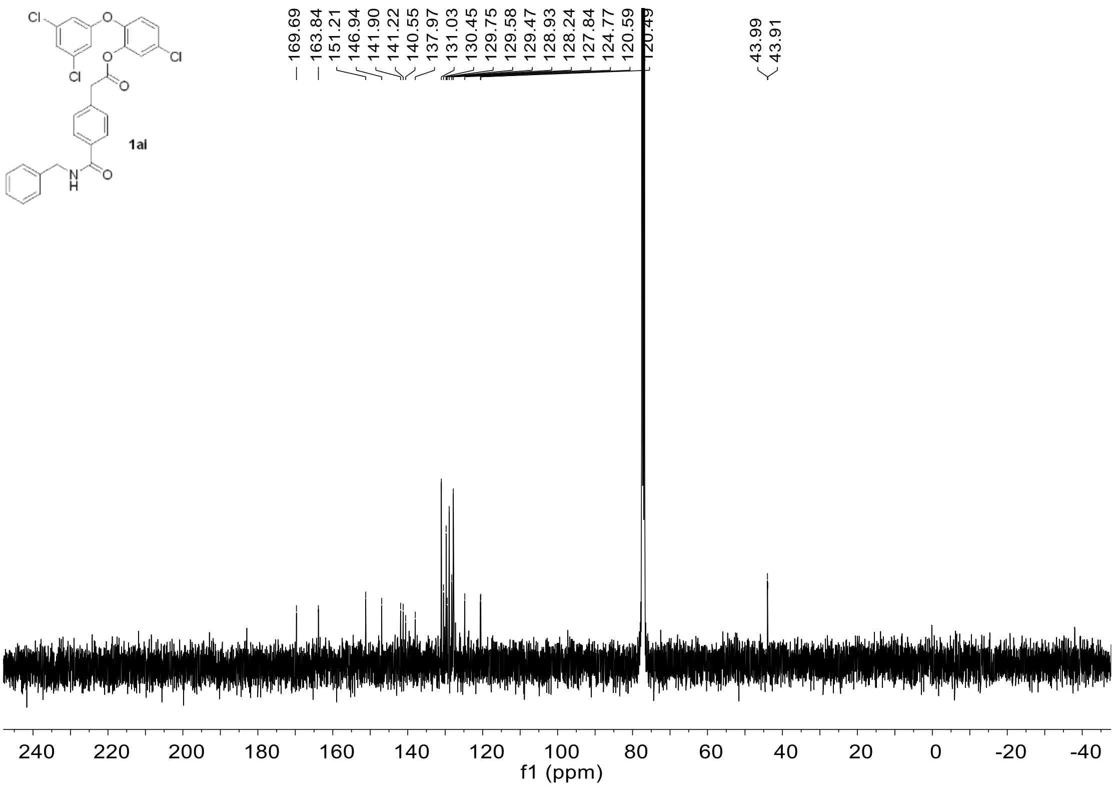

Supplementary Fig. 85. ^{13}C NMR spectrum (101 MHz, $CDCl_3$) of **3ai**

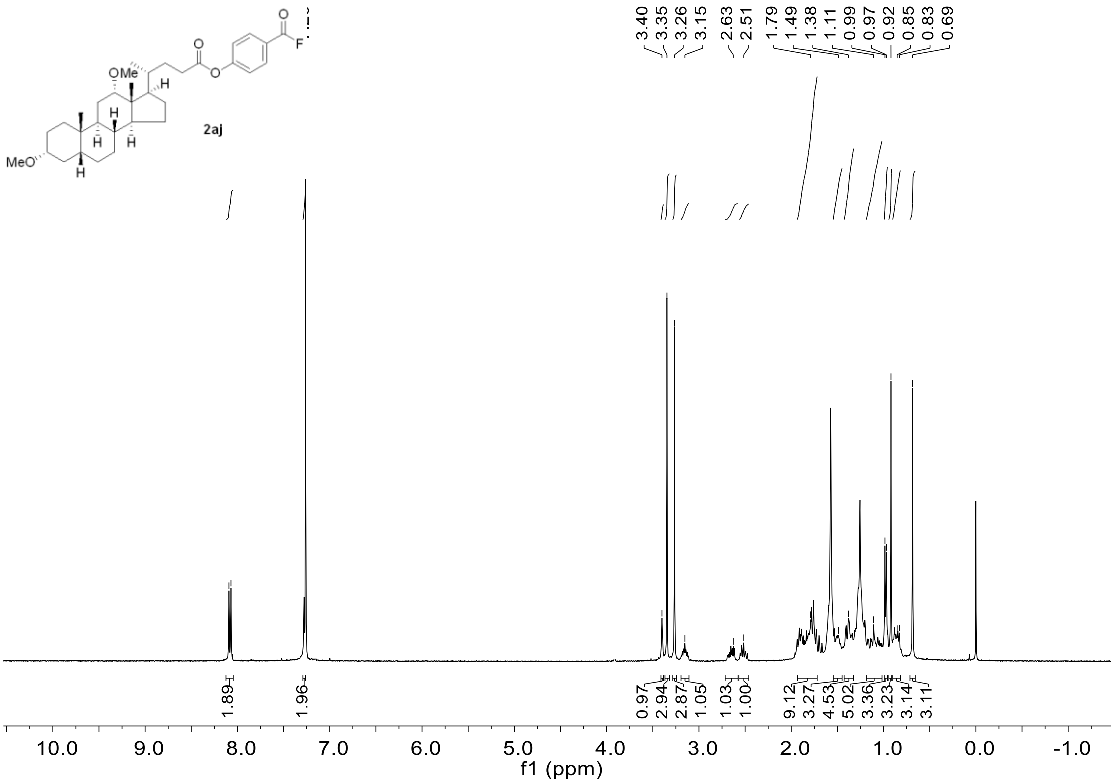

Supplementary Fig. 86. ^{1}H NMR spectrum (400 MHz, $CDCl_3$) of **2aj**

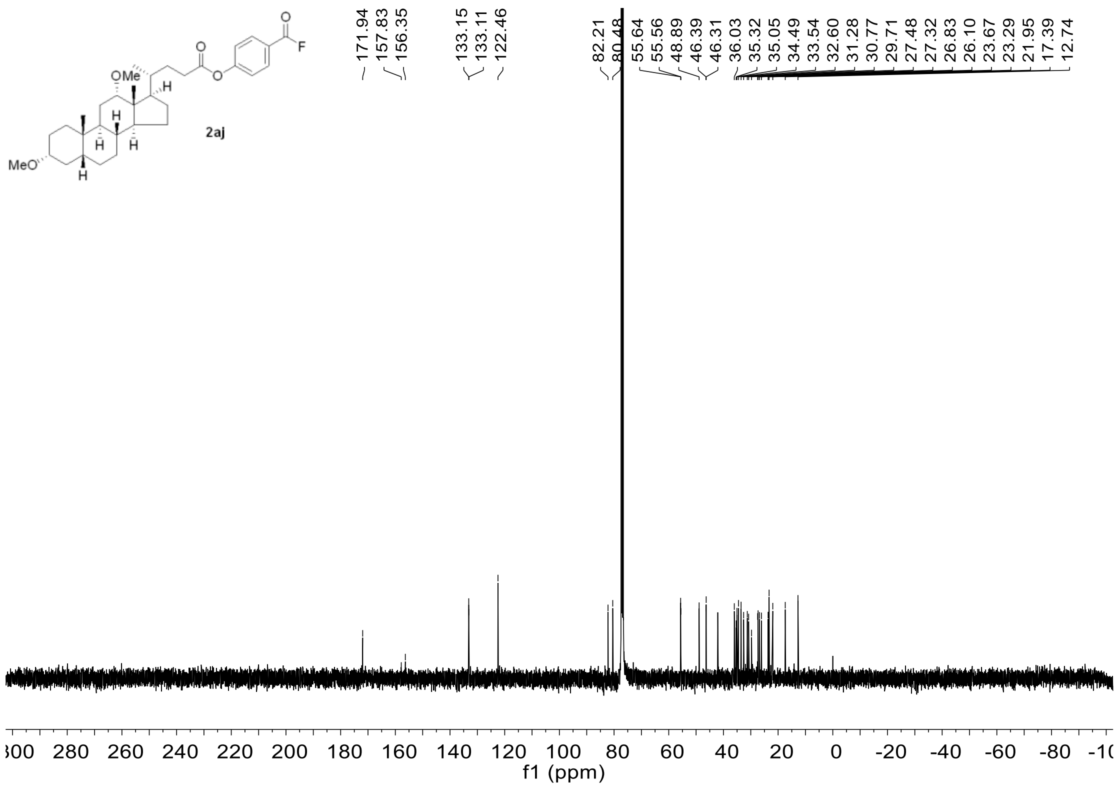

Supplementary Fig. 87. ^{13}C NMR spectrum (101 MHz, $CDCl_3$) of **2aj**

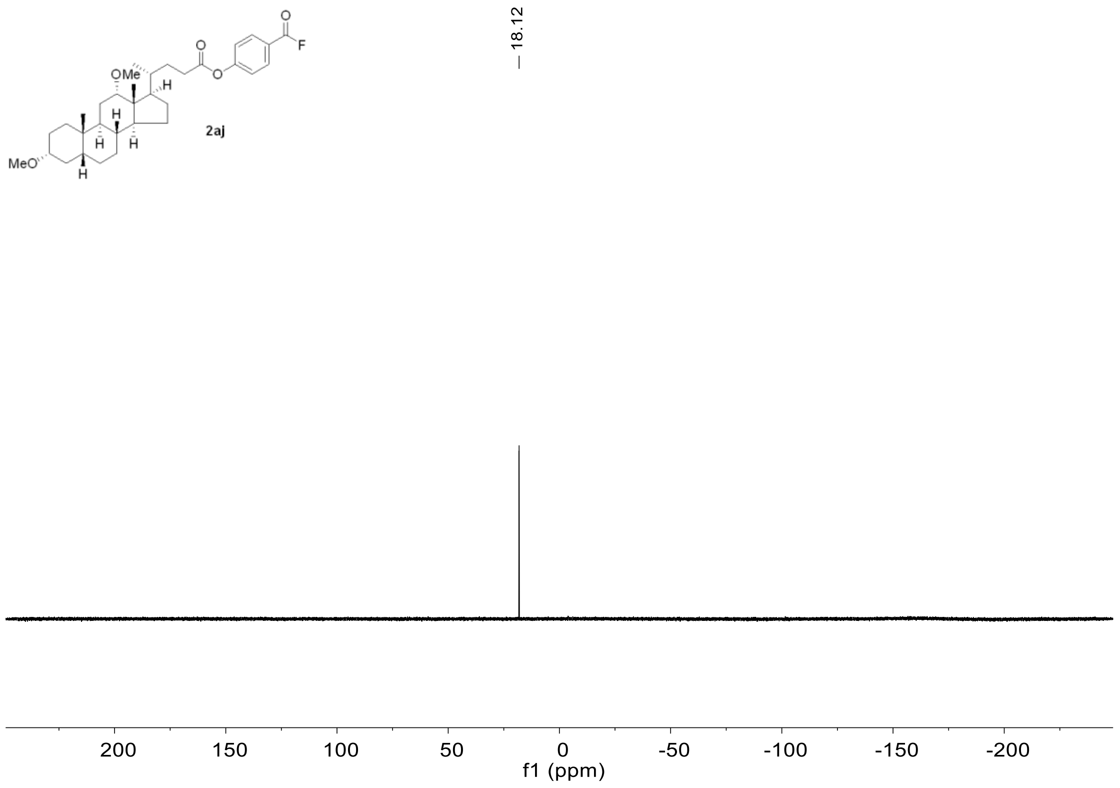

Supplementary Fig. 88. ^{19}F NMR spectrum (376 MHz, $CDCl_3$) of **2aj**

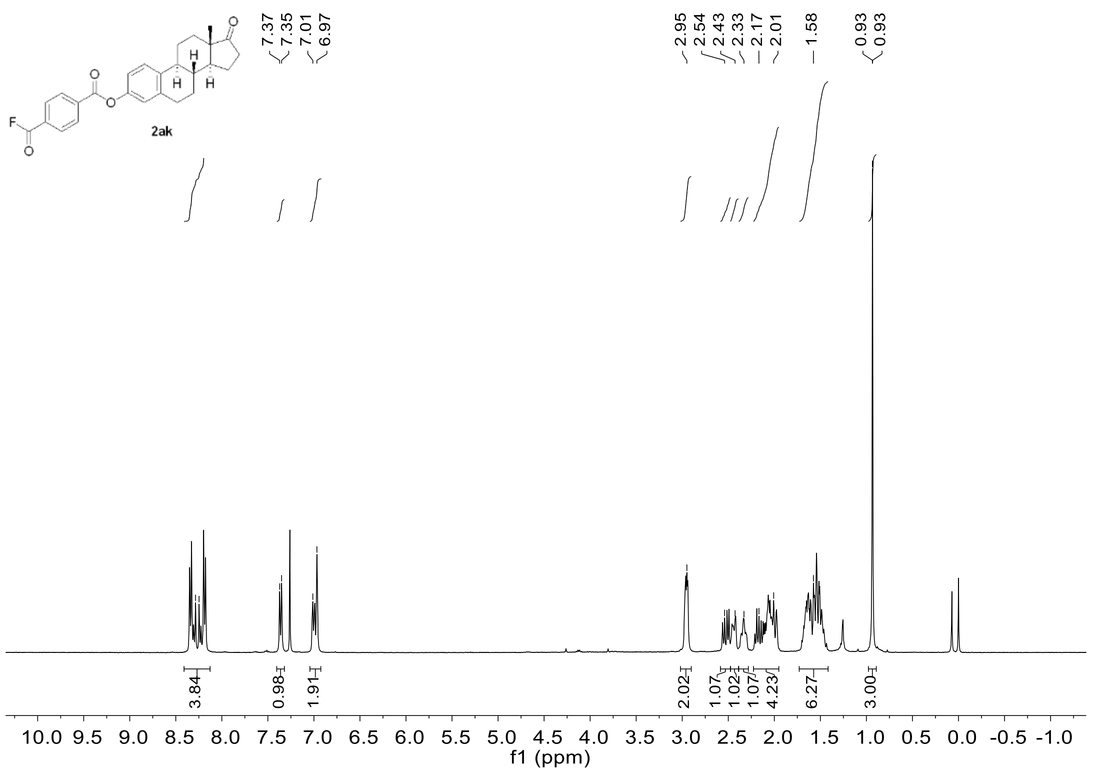

Supplementary Fig. 89. ^{1}H NMR spectrum (400 MHz, $CDCl_3$) of **2ak**

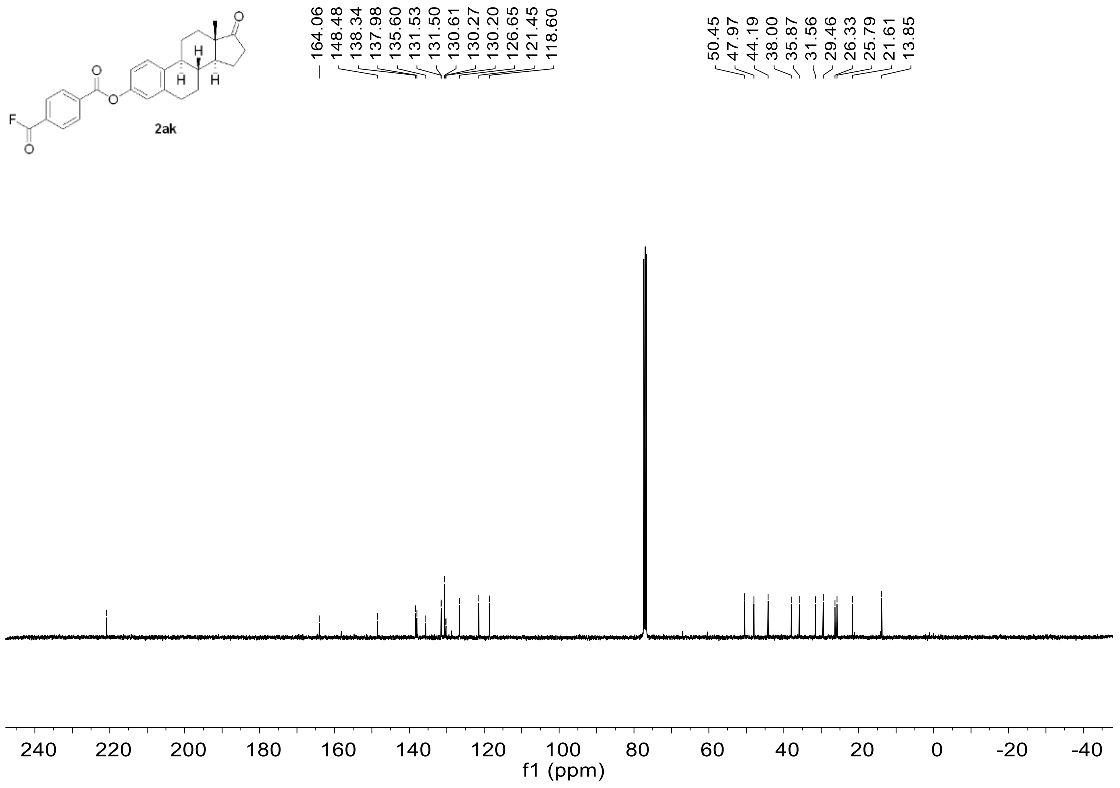

Supplementary Fig. 90. ^{13}C NMR spectrum (101 MHz, $CDCl_3$) of **2ak**

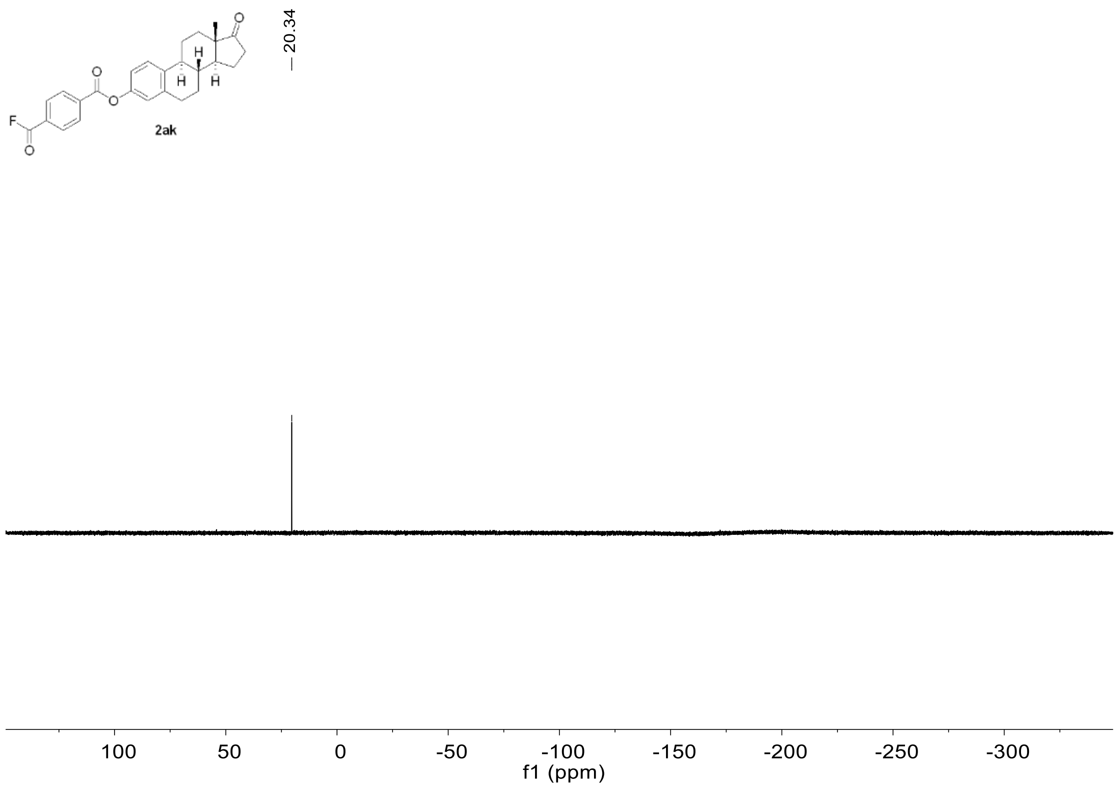

Supplementary Fig. 91. ^{19}F NMR spectrum (376 MHz, $CDCl_3$) of **2ak**

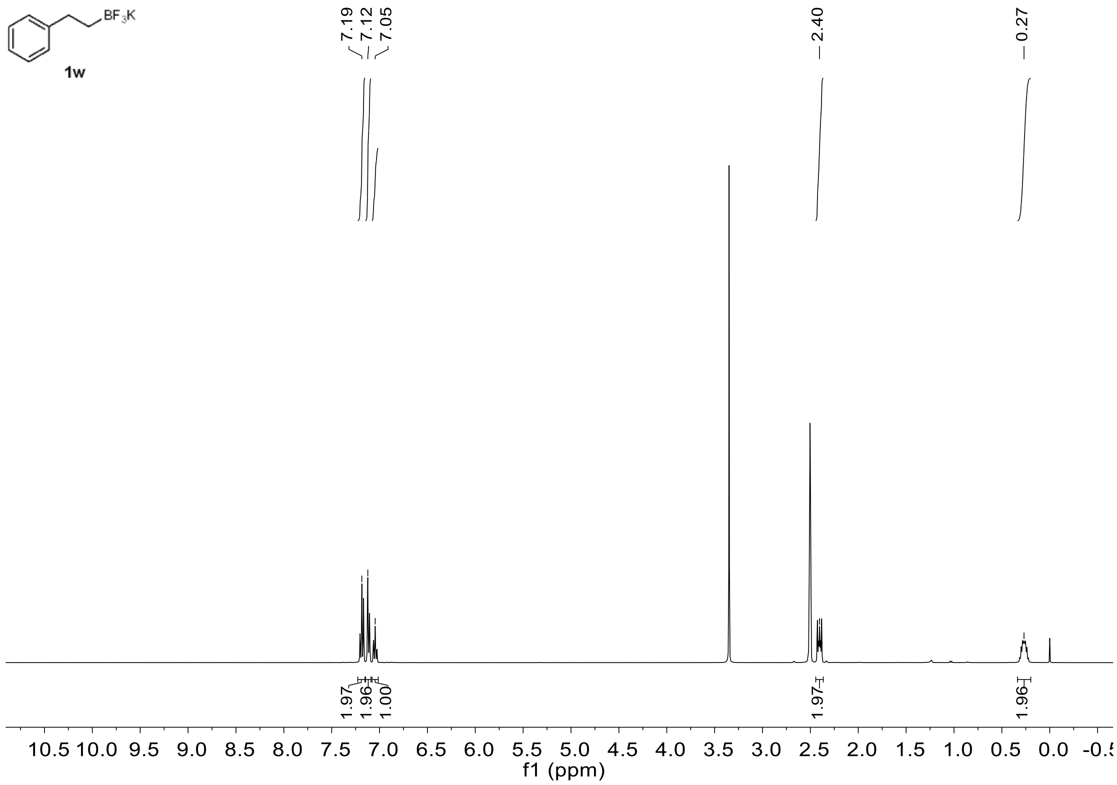

Supplementary Fig. 92. ^{1}H NMR spectrum (400 MHz, DMSO-d_6) of **1w**

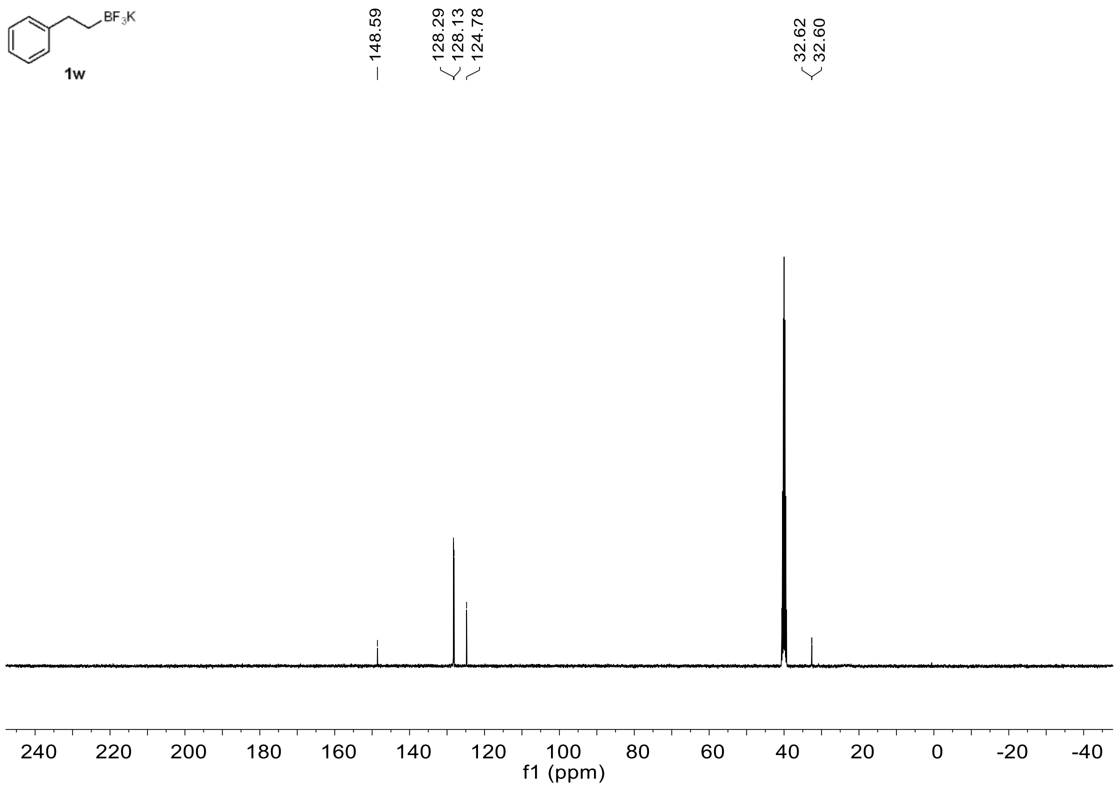

Supplementary Fig. 93. ^{13}C NMR spectrum (101 MHz, DMSO-d_6) of **1w**

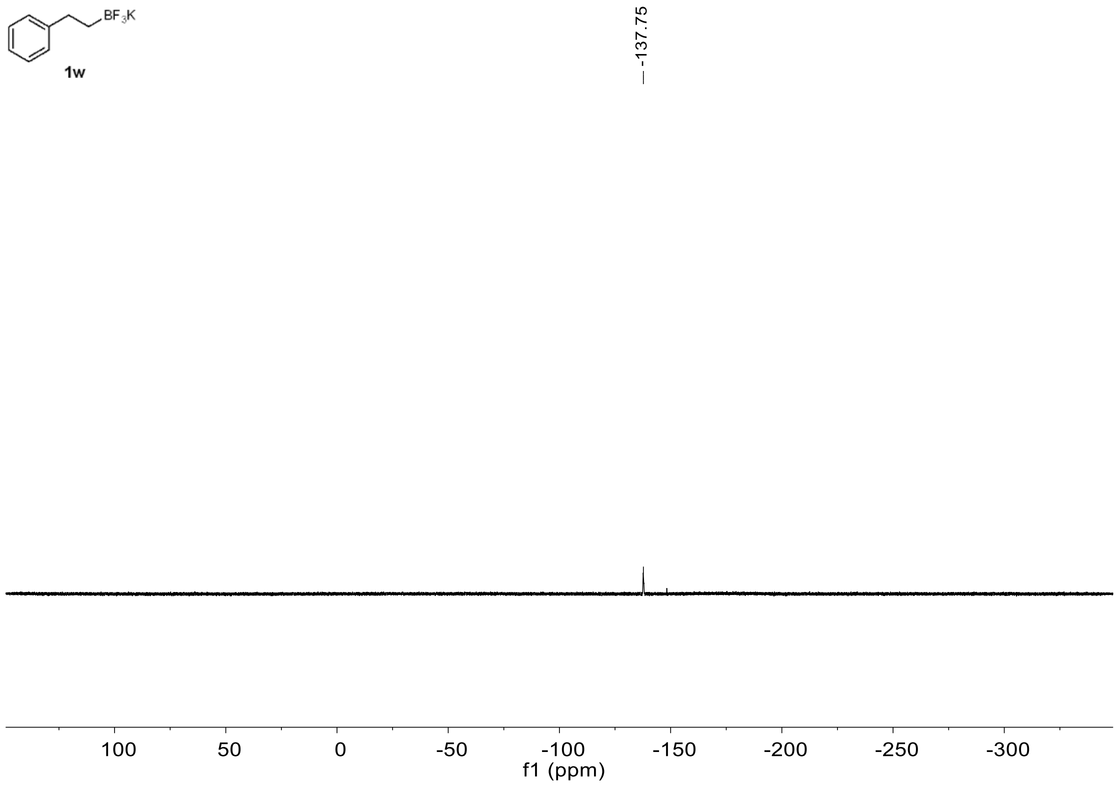

Supplementary Fig. 94. ^{19}F NMR spectrum (376 MHz, DMSO-d_6) of **1w**

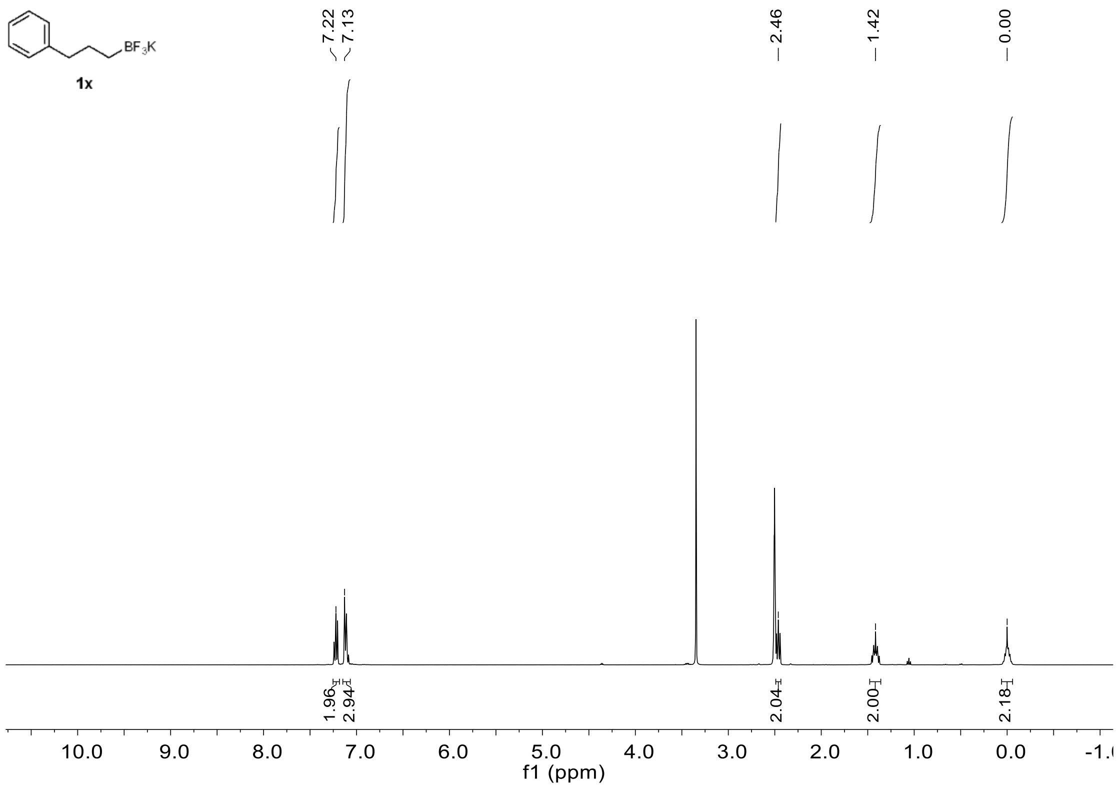

Supplementary Fig. 95. ^{1}H NMR spectrum (400 MHz, DMSO-d_6) of **1x**

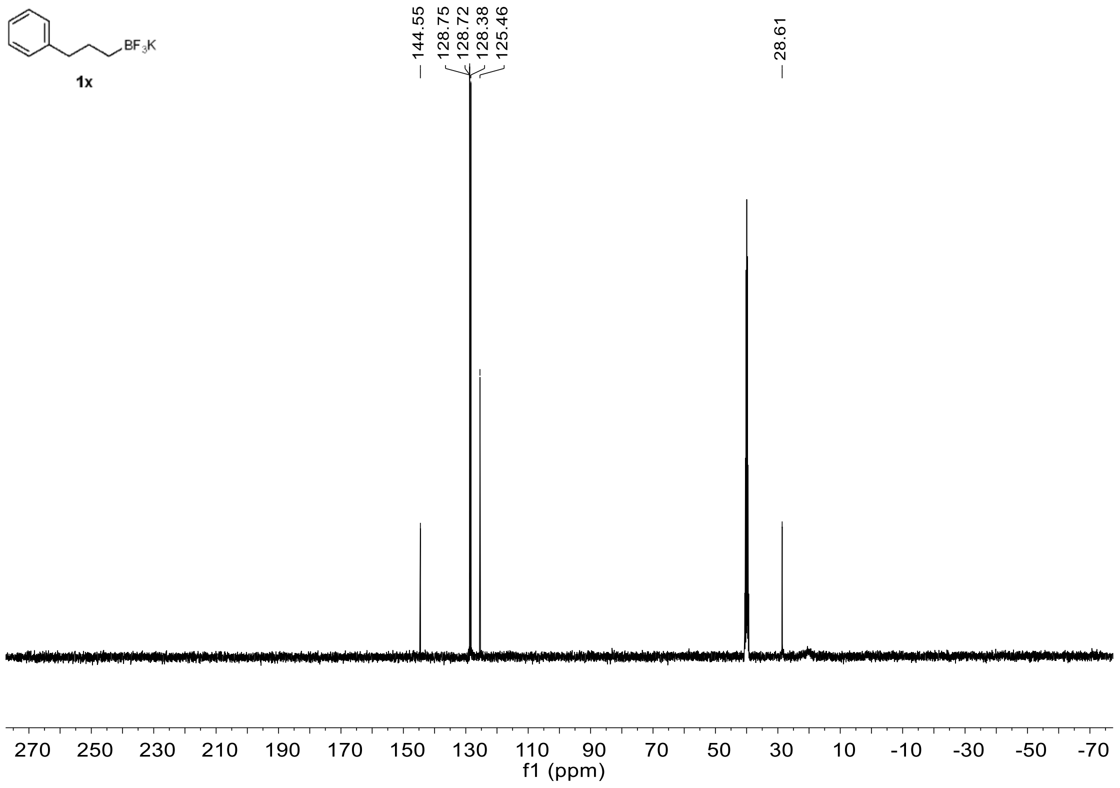

Supplementary Fig. 96. ^{13}C NMR spectrum (101 MHz, DMSO-d_6) of **1x**

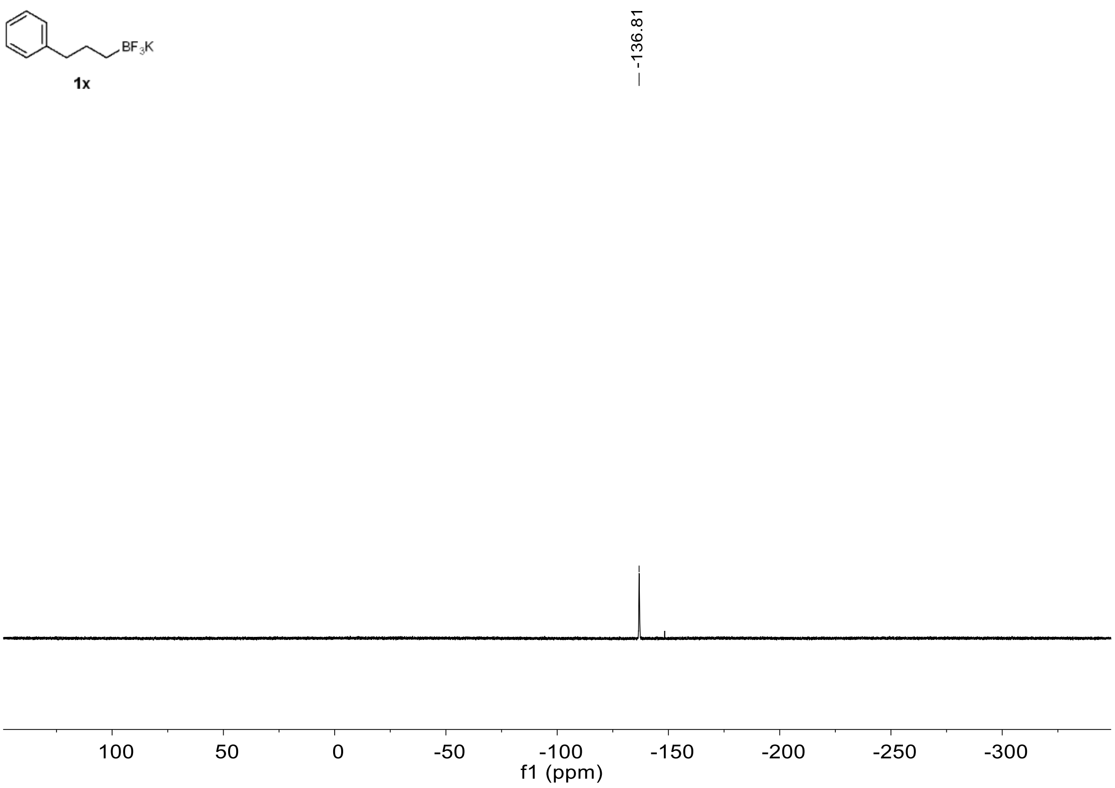

Supplementary Fig. 97. ^{19}F NMR spectrum (376 MHz, DMSO-d_6) of **1x**

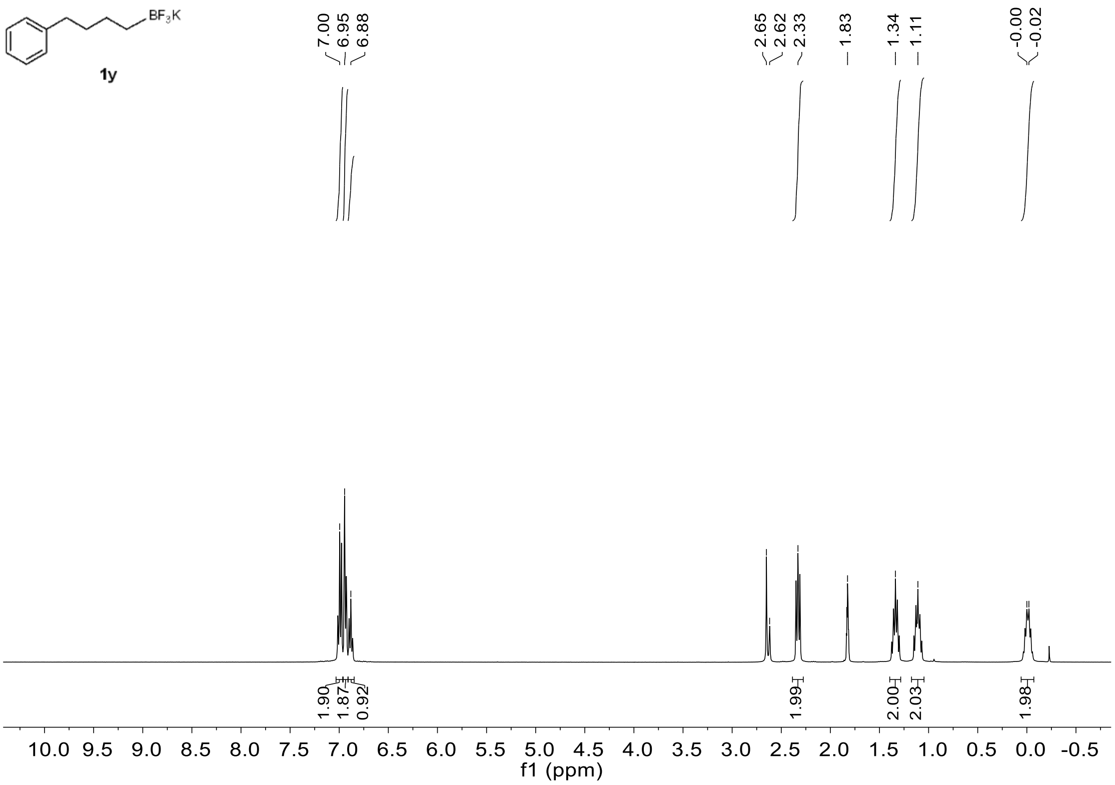

Supplementary Fig. 98. ^{1}H NMR spectrum (400 MHz, Acetone-d_6) of **1y**

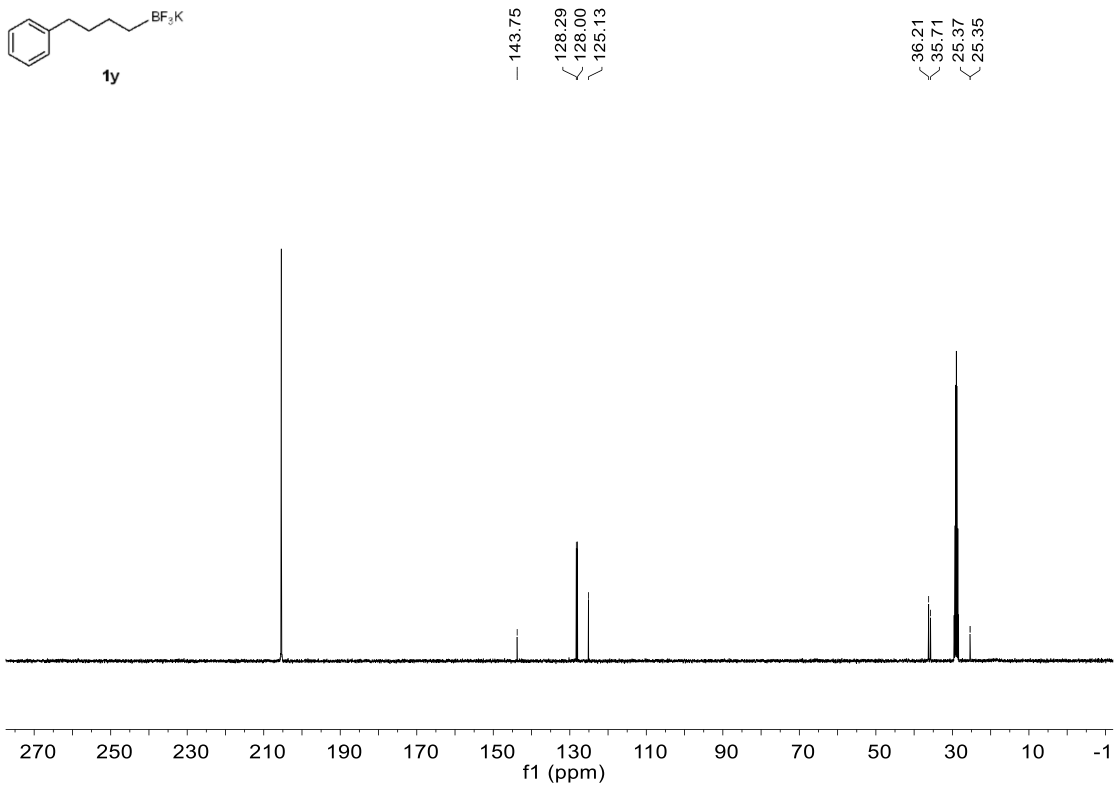

Supplementary Fig. 99. ^{13}C NMR spectrum (101 MHz, Acetone-d_6) of **1y**

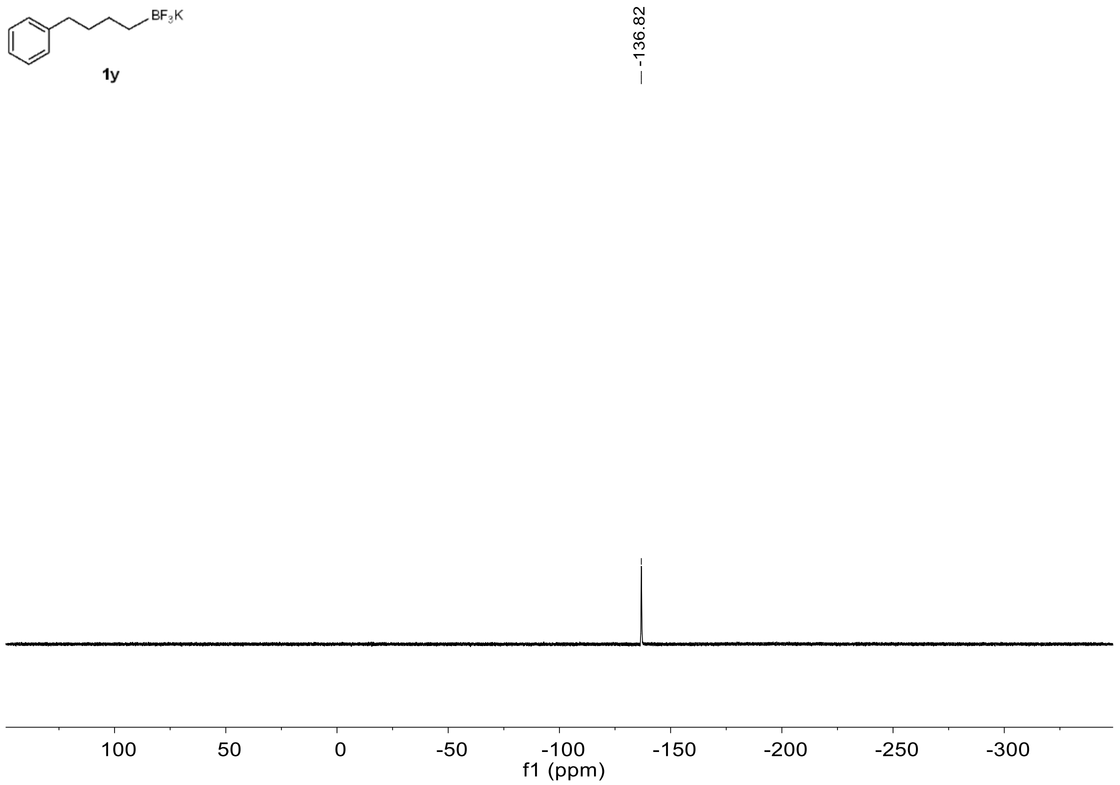

Supplementary Fig. 100. ^{19}F NMR spectrum (376 MHz, DMSO-d_6) of **1y**

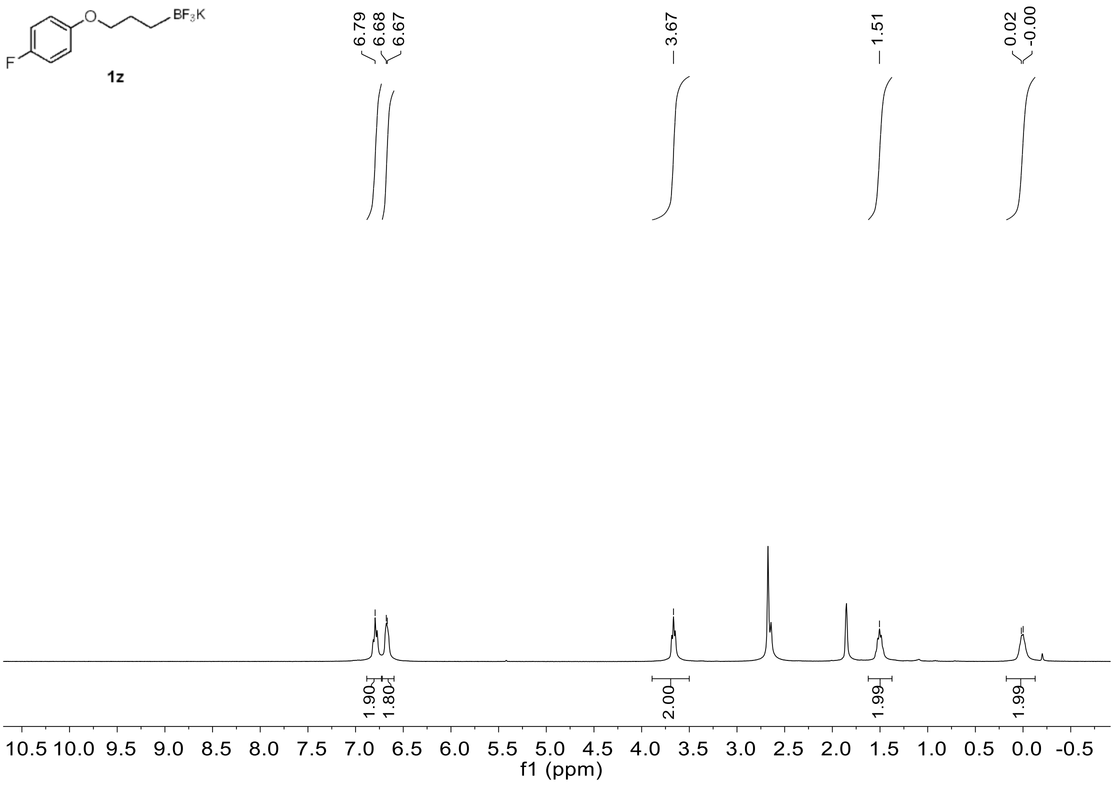

Supplementary Fig. 101. ^{1}H NMR spectrum (400 MHz, Acetone-d_6) of **1z**

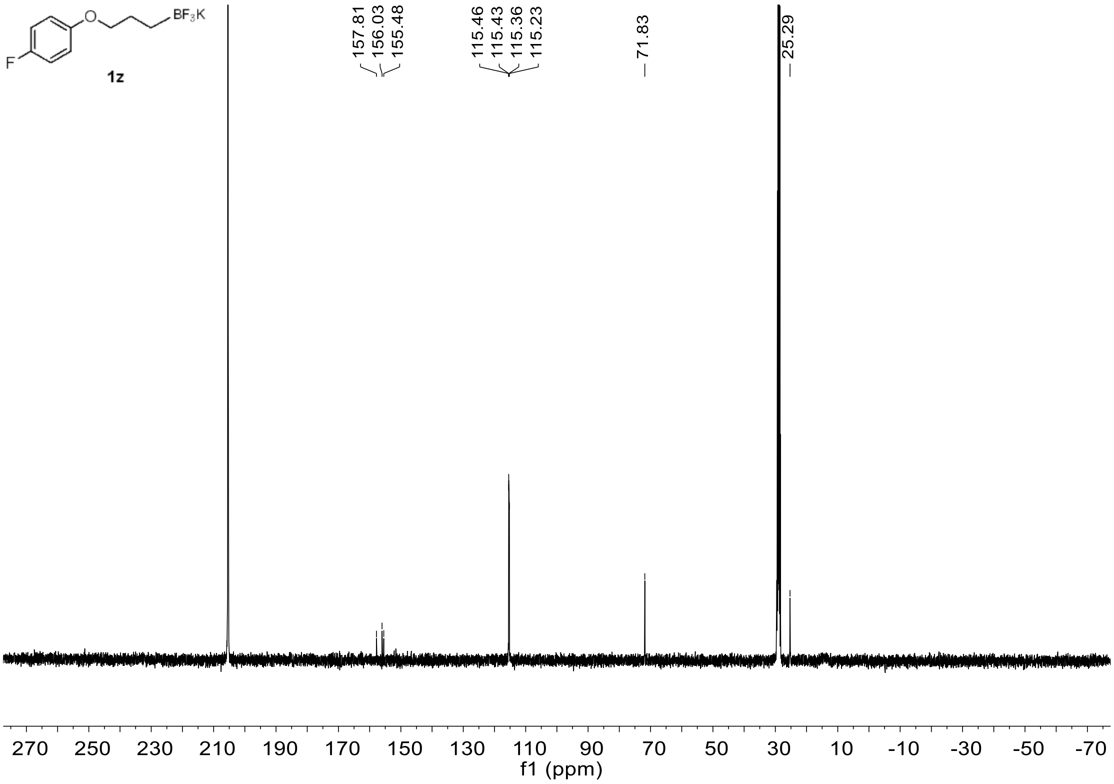

Supplementary Fig. 102. ^{13}C NMR spectrum (101 MHz, Acetone-d_6) of **1z**

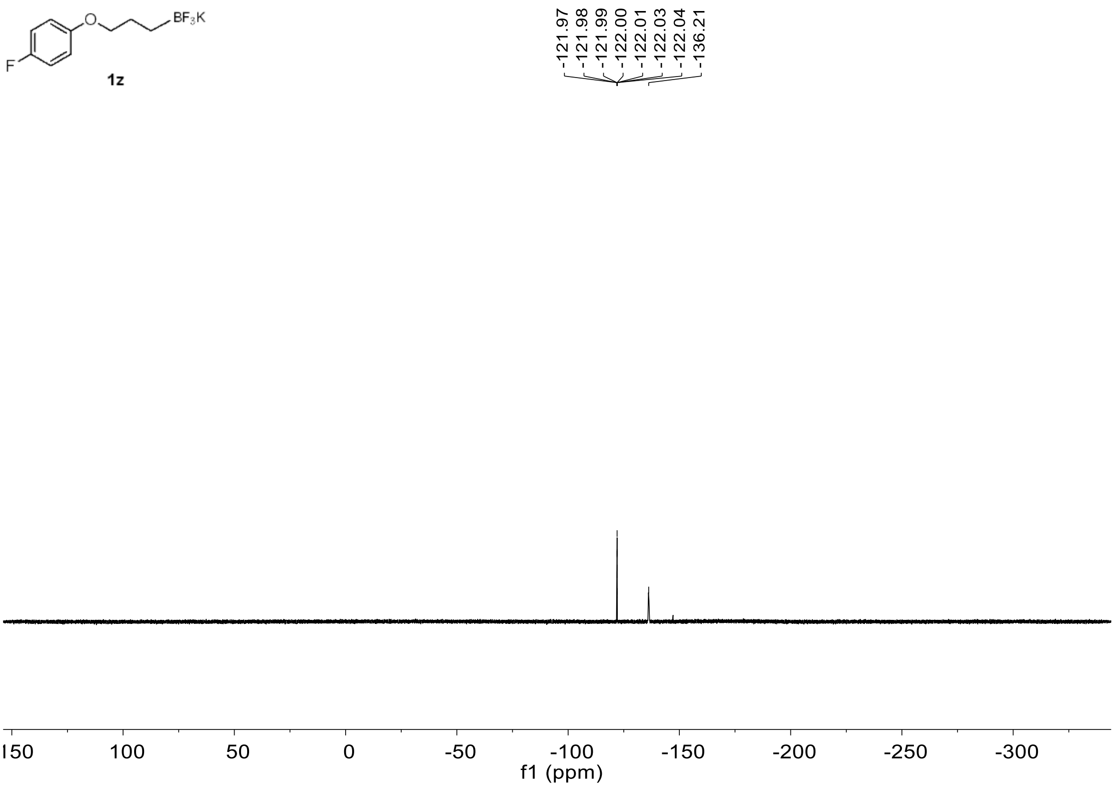

Supplementary Fig. 103. ^{19}F NMR spectrum (376 MHz, Acetone-d_6) of **1z**

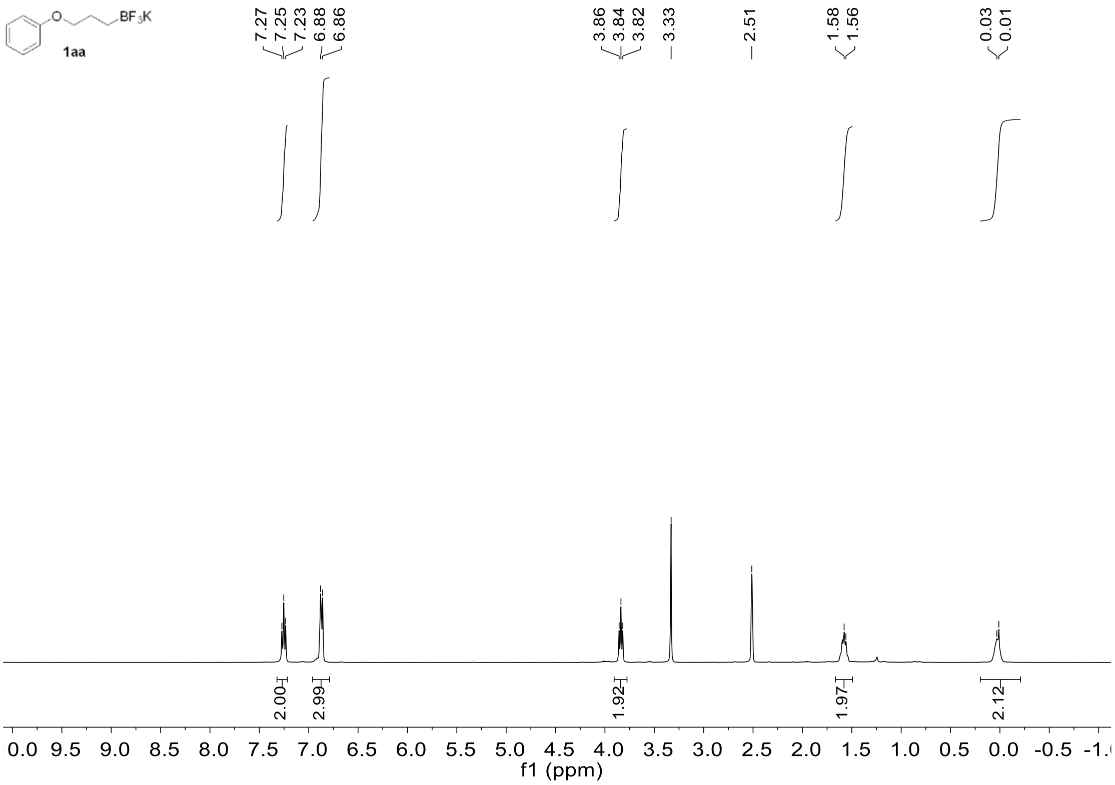

Supplementary Fig. 104. ^{1}H NMR (400 MHz, DMSO-d_6) of **1aa**

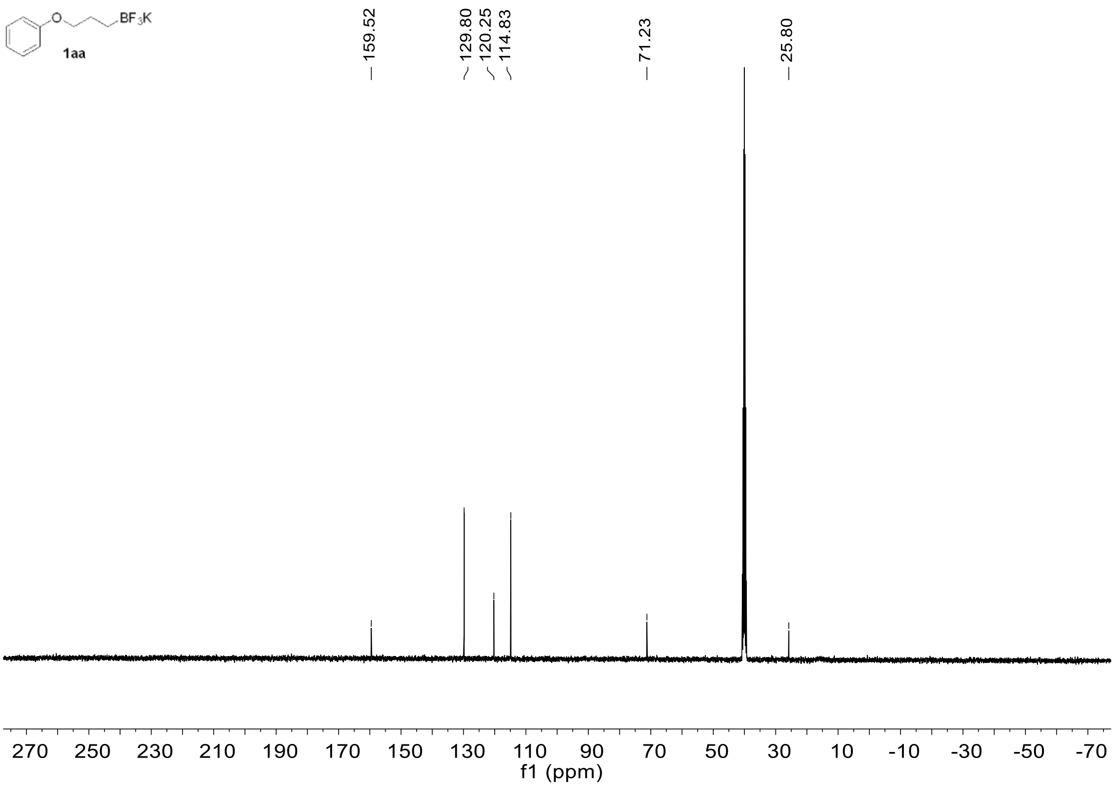

Supplementary Fig. 105. ^{13}C NMR (101 MHz, DMSO-d_6) of **1aa**

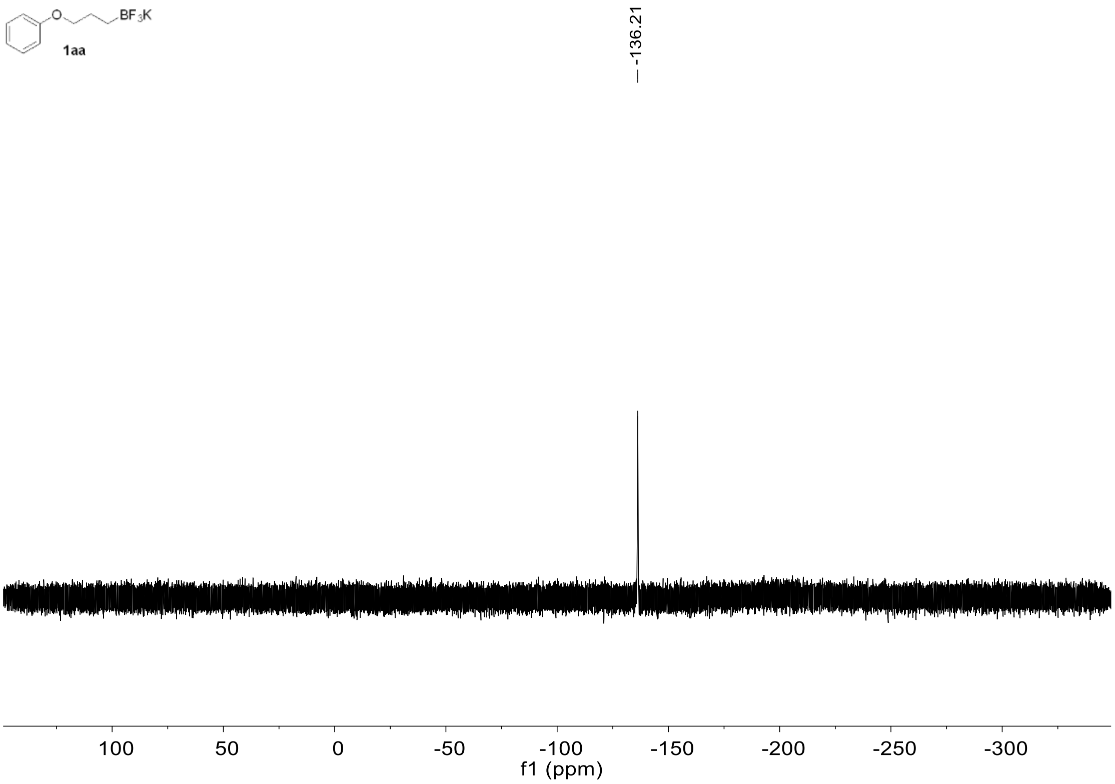

Supplementary Fig. 106. ^{19}F NMR (376 MHz, DMSO-d_6) of **1aa**

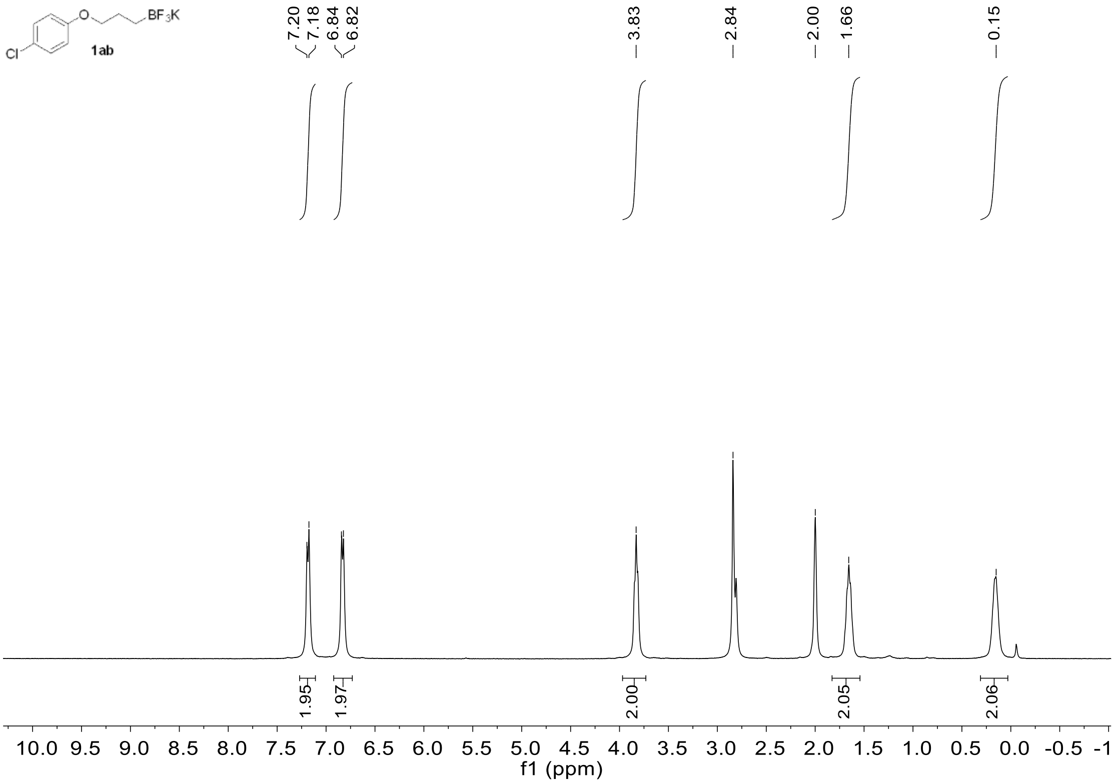

Supplementary Fig. 107. ^{1}H NMR spectrum (400 MHz, Acetone-d_6) of **1ab**

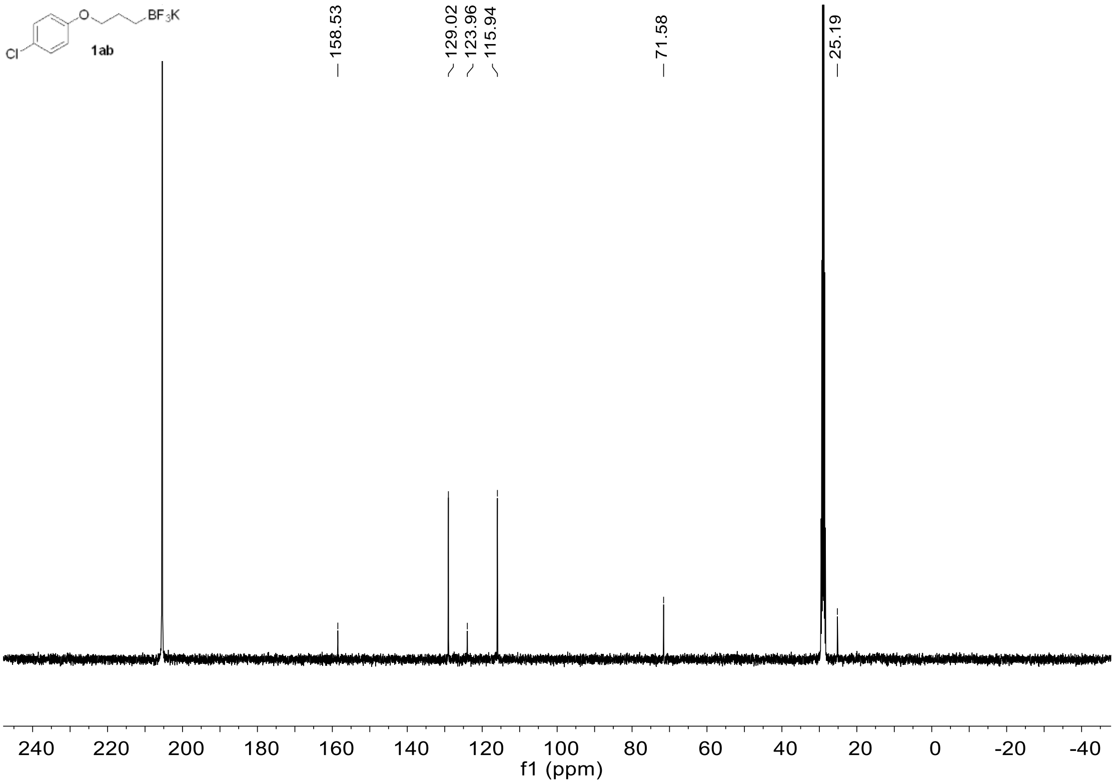

Supplementary Fig. 108. ^{13}C NMR spectrum (101 MHz, Acetone-d_6) of **1ab**

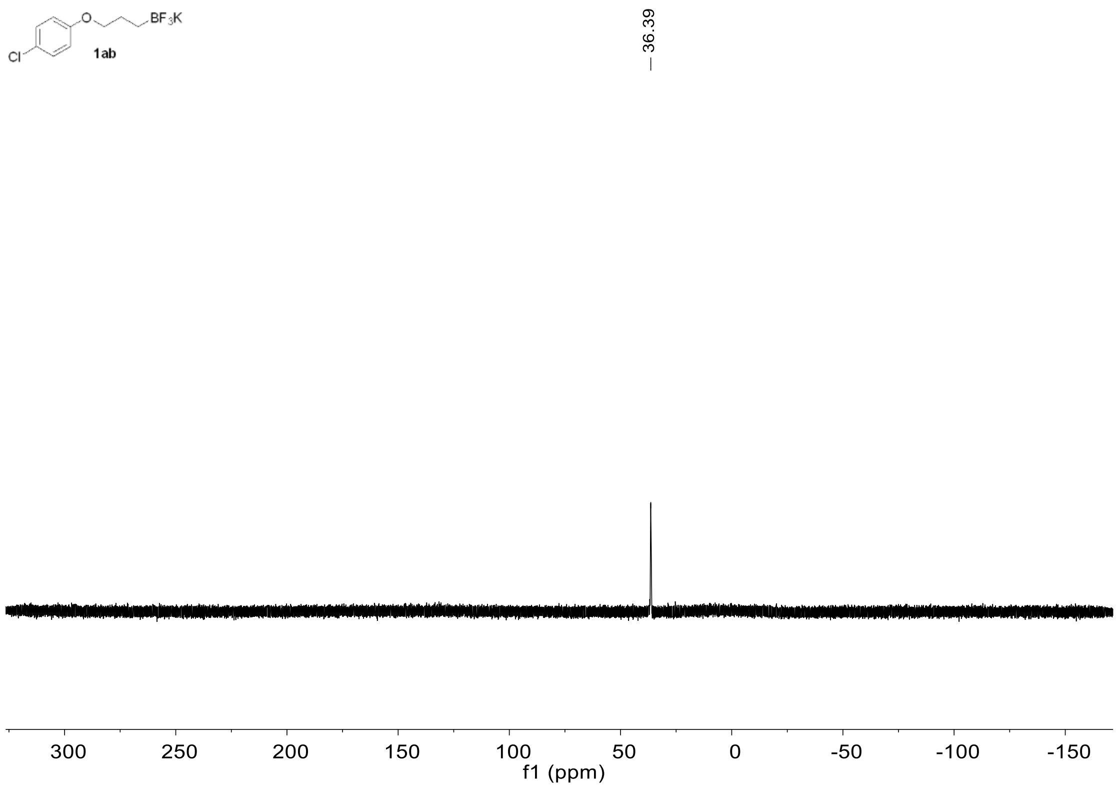

Supplementary Fig. 109. ^{19}F NMR spectrum (376 MHz, Acetone-d_6) of **1ab**

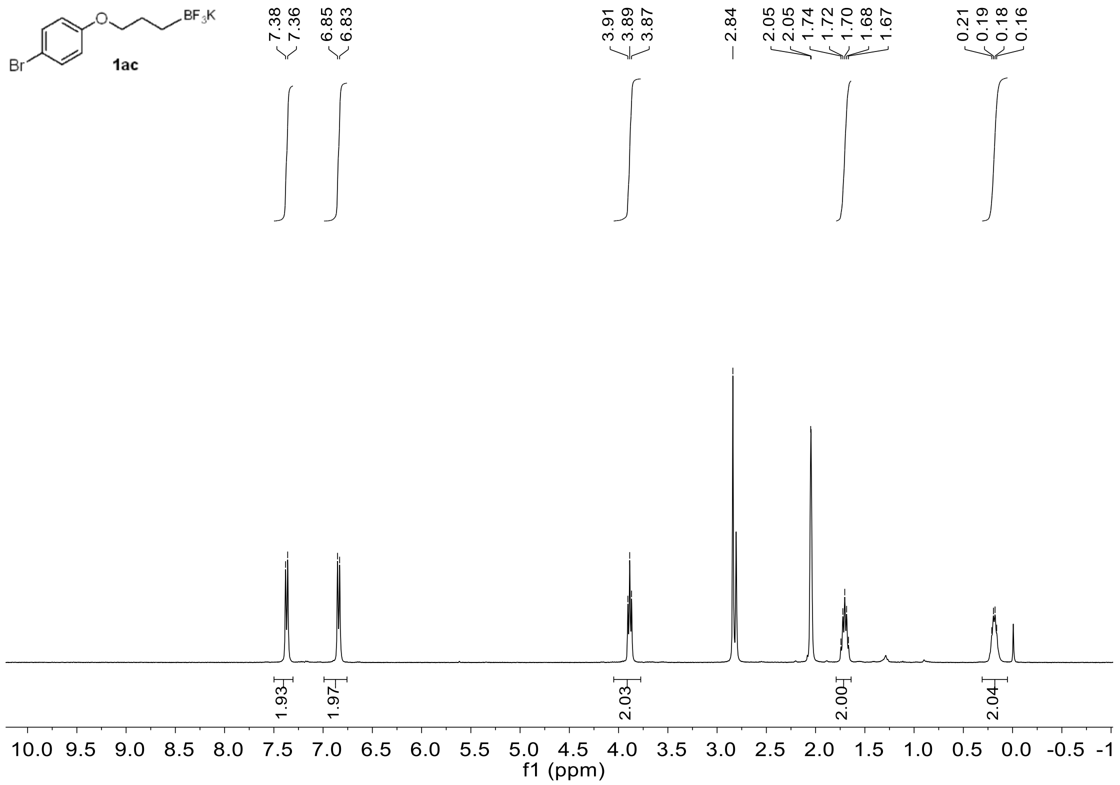

Supplementary Fig. 110. ^{1}H NMR spectrum (400 MHz, Acetone-d_6) of **1ac**

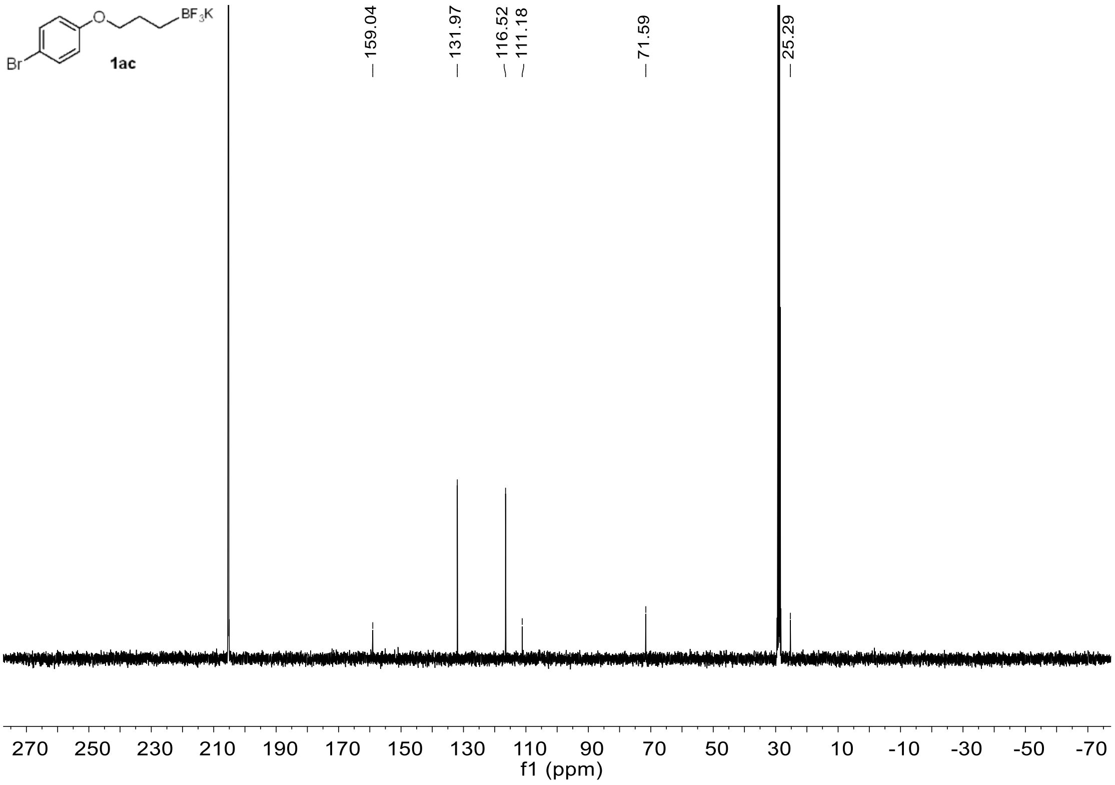

Supplementary Fig. 111. ^{13}C NMR spectrum (101 MHz, Acetone-d_6) of **1ac**

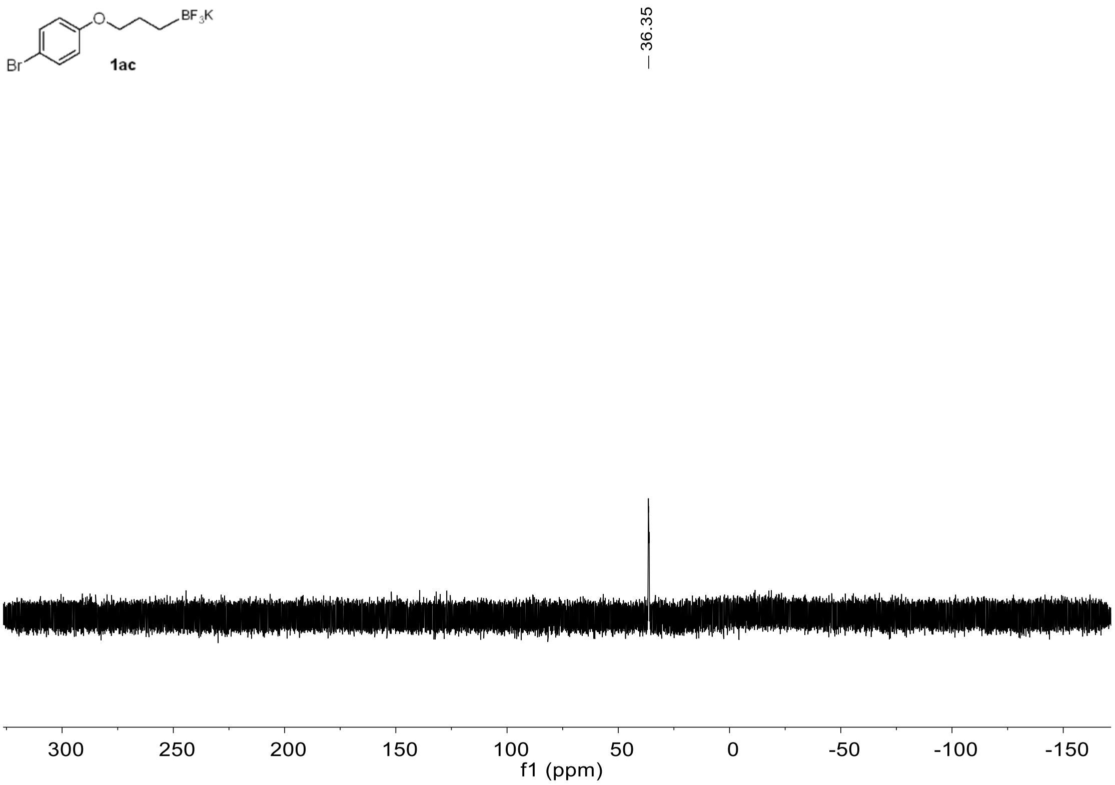

Supplementary Fig. 112. ^{19}F NMR spectrum (376 MHz, Acetone-d_6) of **1ac**

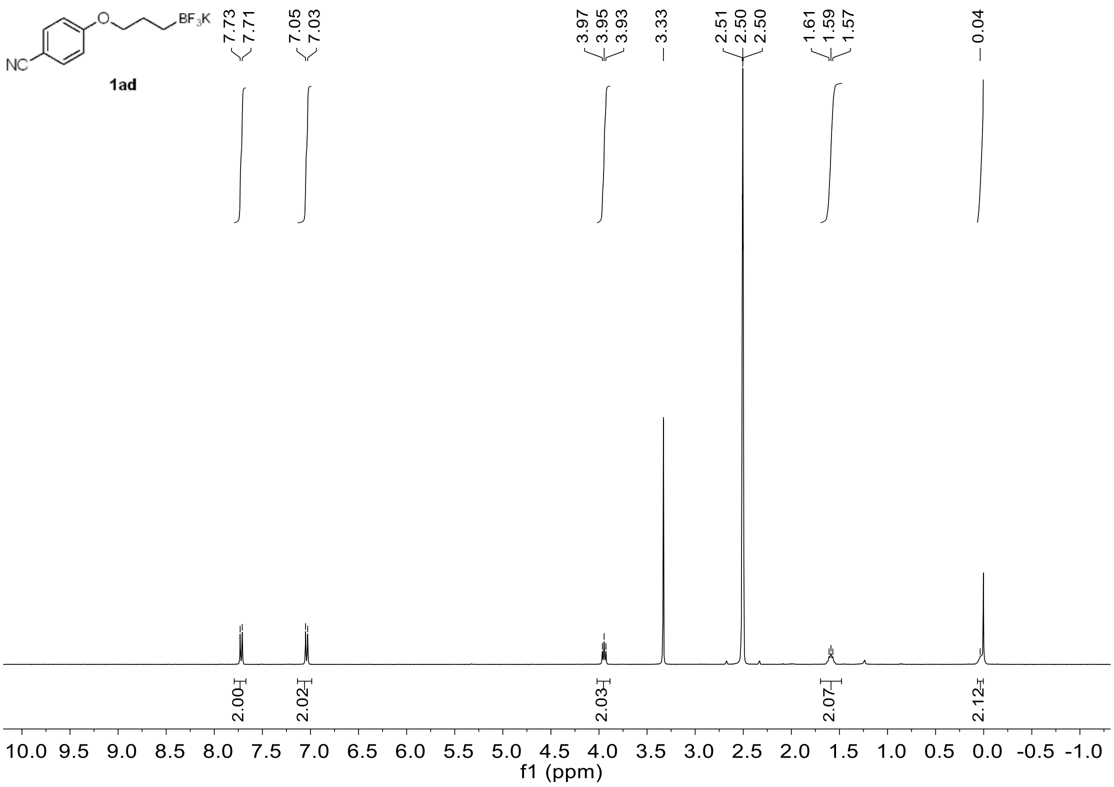

Supplementary Fig. 113. ^{1}H NMR (400 MHz, DMSO-d_6) of **1ad**

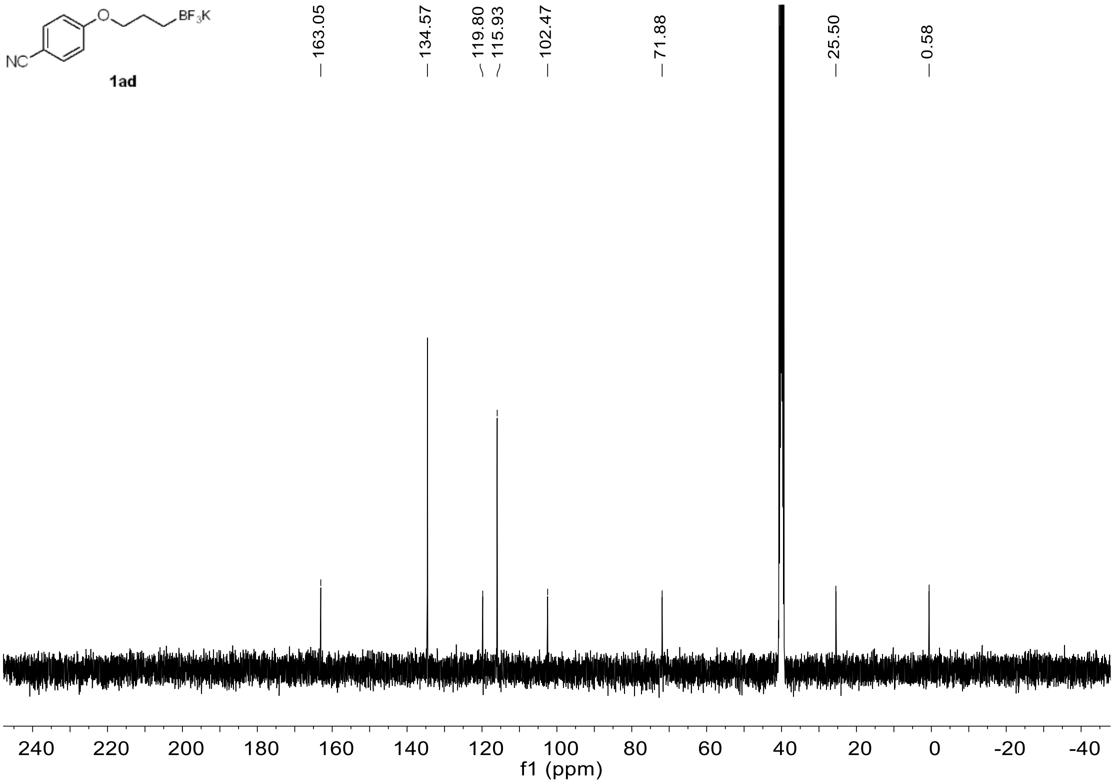

Supplementary Fig. 114. ^{13}C NMR (101 MHz, DMSO-d_6) of **1ad**

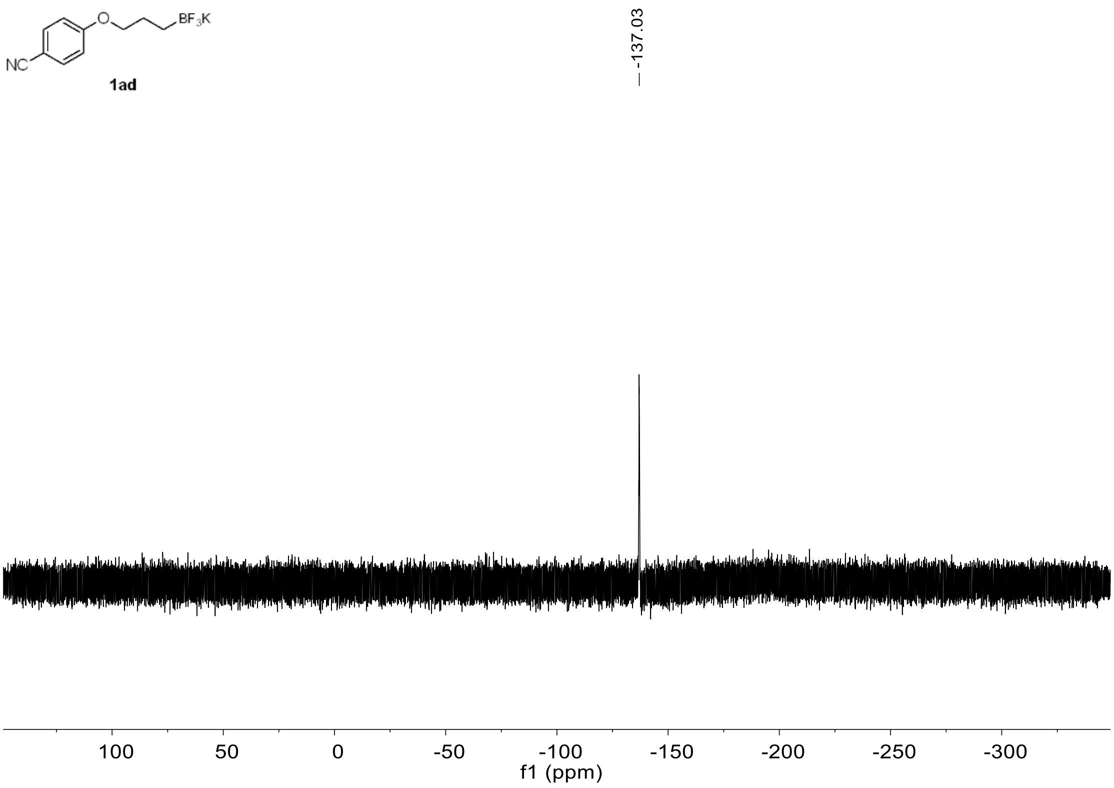

Supplementary Fig. 115. ^{19}F NMR (376 MHz, DMSO-d_6) of **1ad**

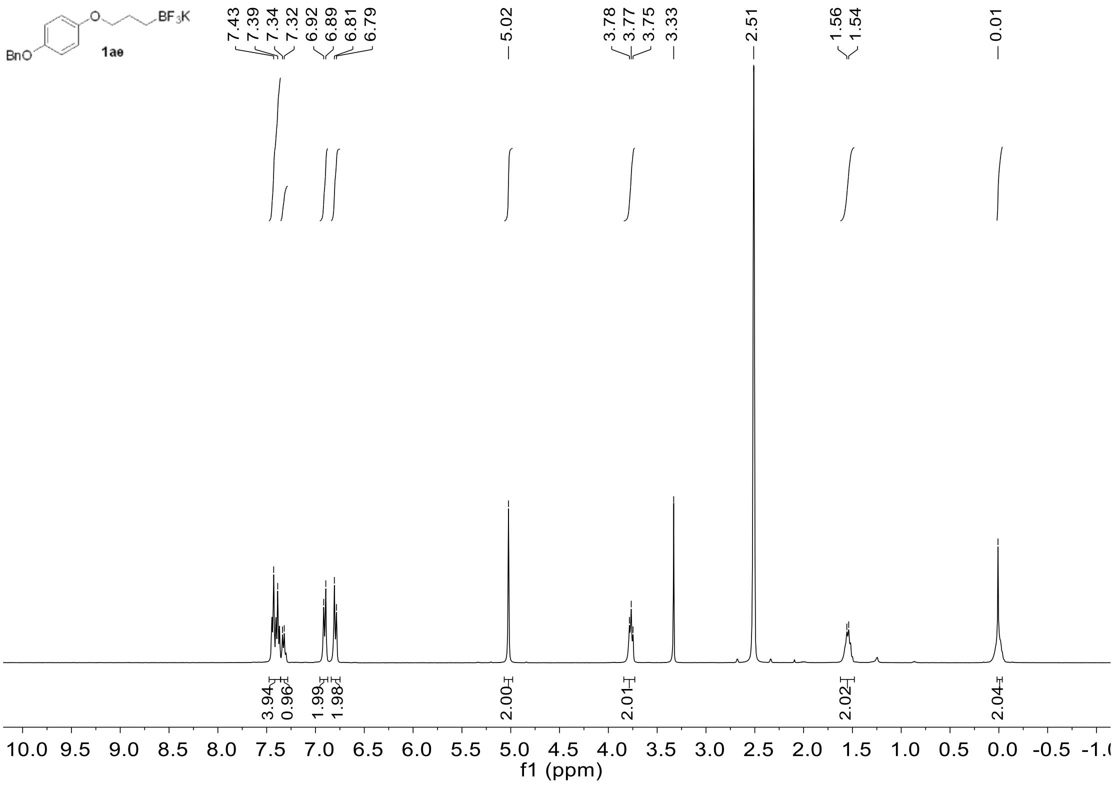

Supplementary Fig. 116. ^{1}H NMR (400 MHz, DMSO-d_6) of **1ae**

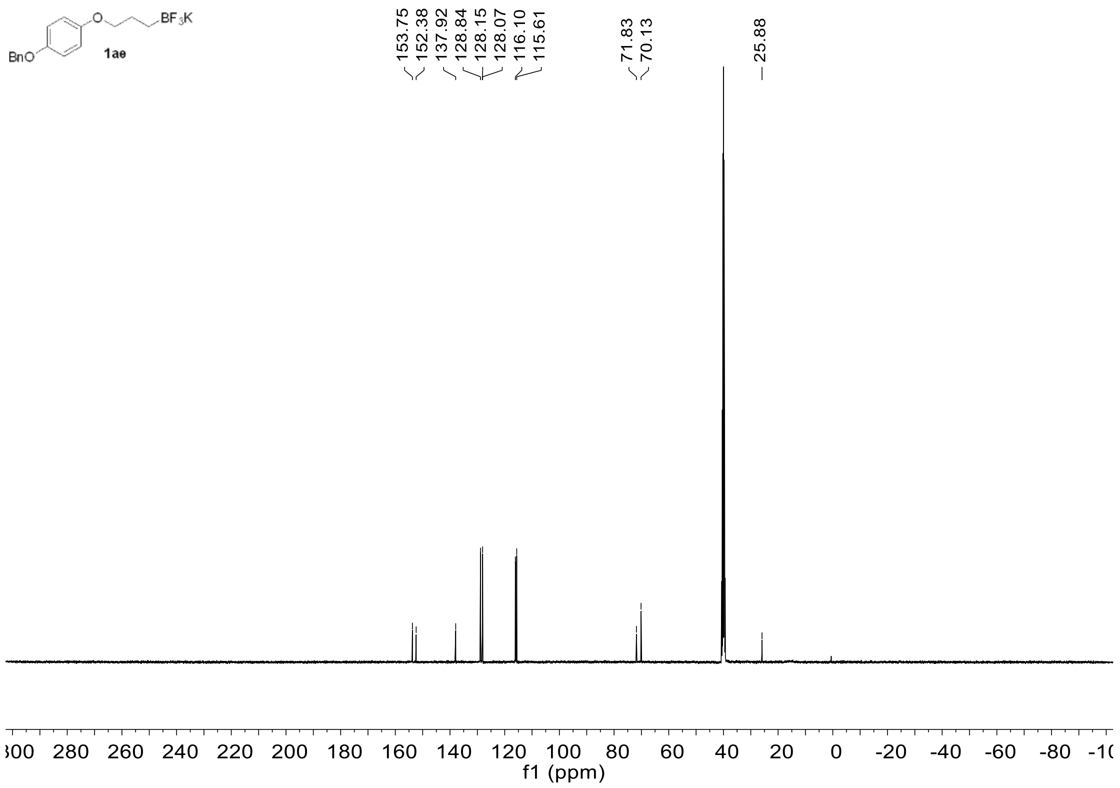

Supplementary Fig. 117. ^{13}C NMR (101 MHz, DMSO-d_6) of **1ae**

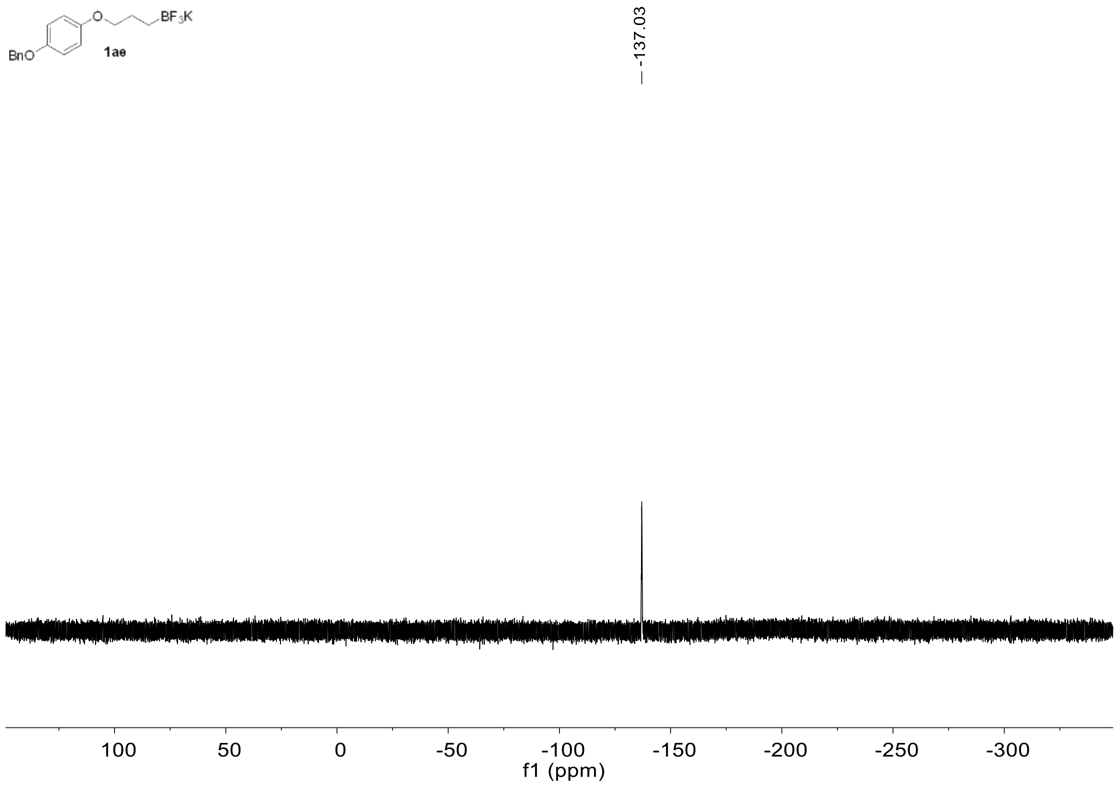

Supplementary Fig. 118. ^{19}F NMR (376 MHz, DMSO-d_6) of **1ae**

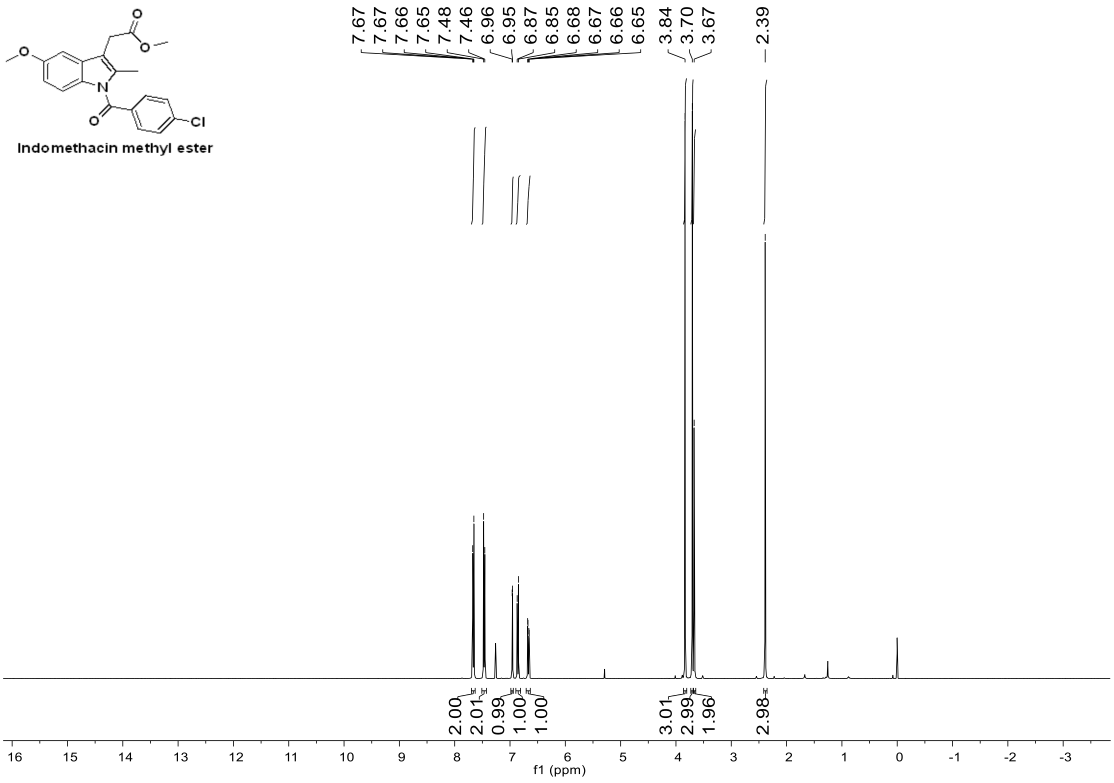

Supplementary Fig. 119. ^{1}H NMR spectrum (400 MHz, $CDCl_3$) of **Indomethacin methyl ester**

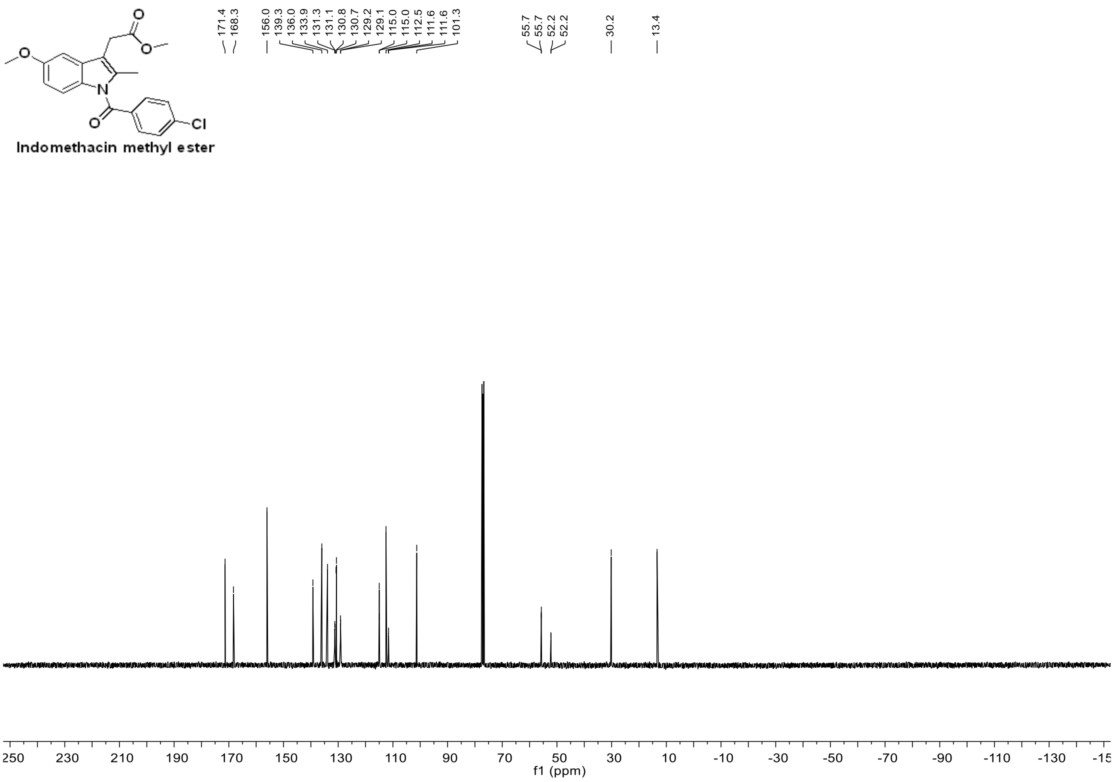

Supplementary Fig. 120. ^{13}C NMR spectrum (101 MHz, $CDCl_3$) of **Indomethacin methyl ester**

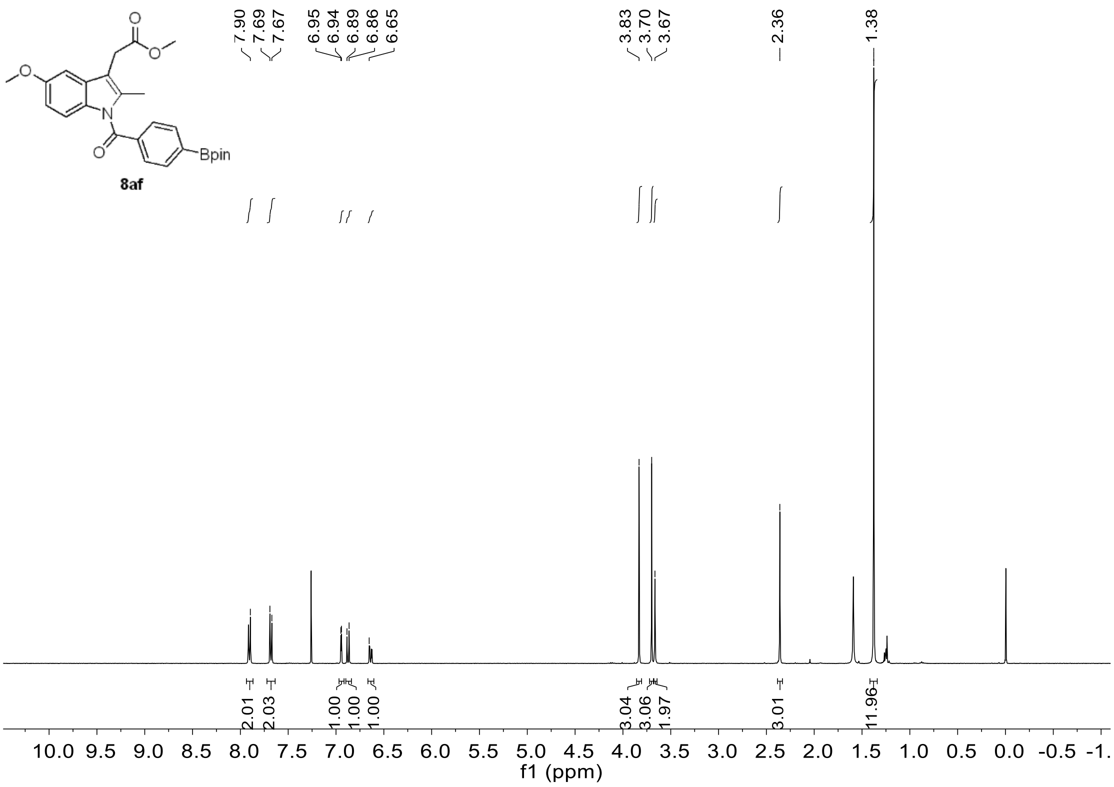

Supplementary Fig. 121. ^{1}H NMR spectrum (400 MHz, $CDCl_3$) of **8af**

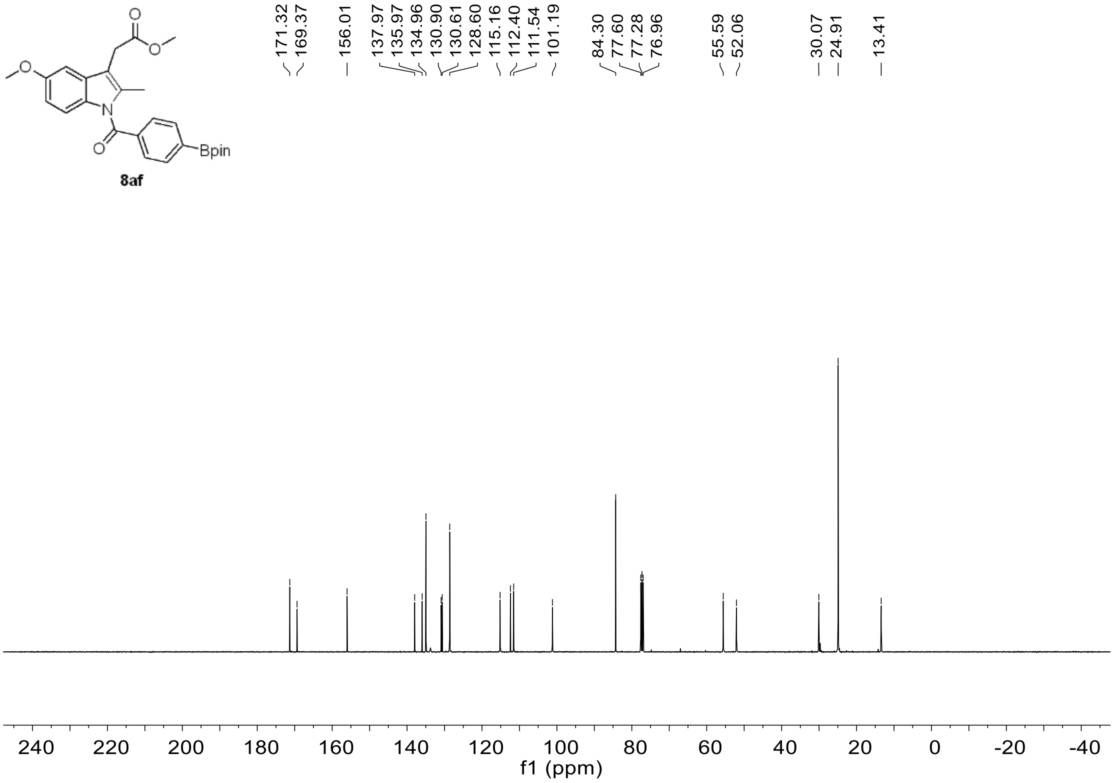

Supplementary Fig. 122. ^{13}C NMR spectrum (101 MHz, $CDCl_3$) of **8af**

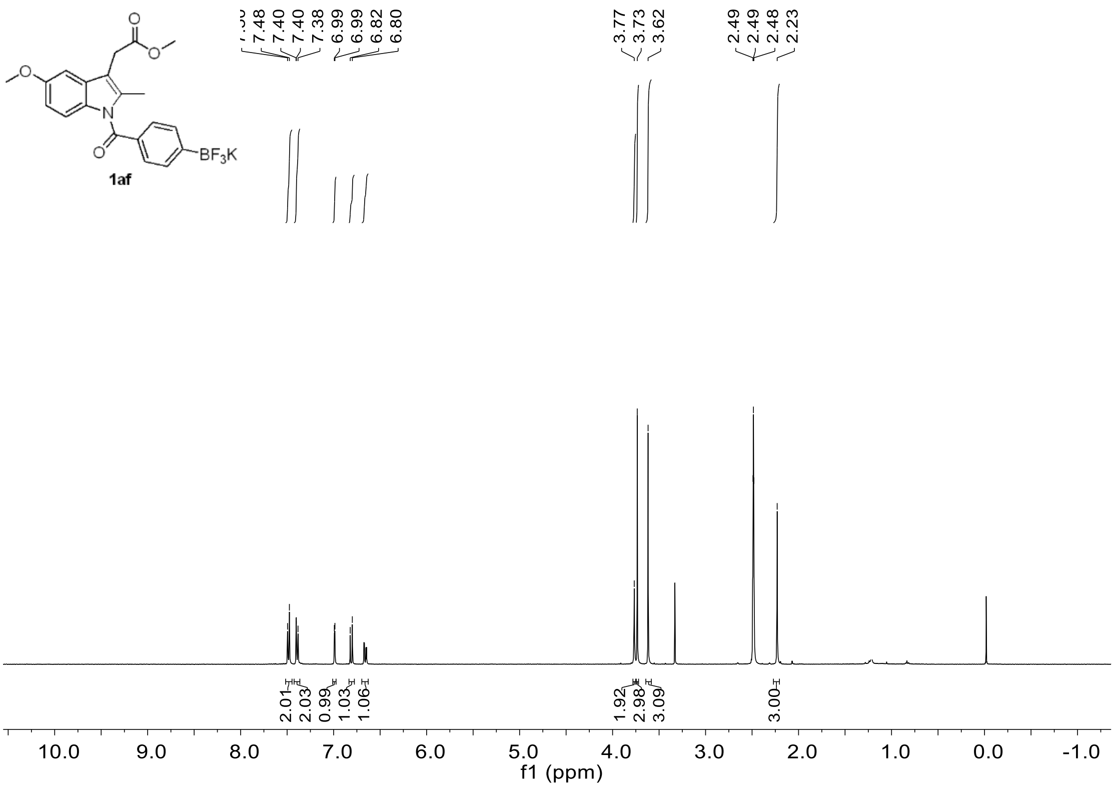

Supplementary Fig. 123. ^{1}H NMR spectrum (400 MHz, DMSO-d_6) of **1af**

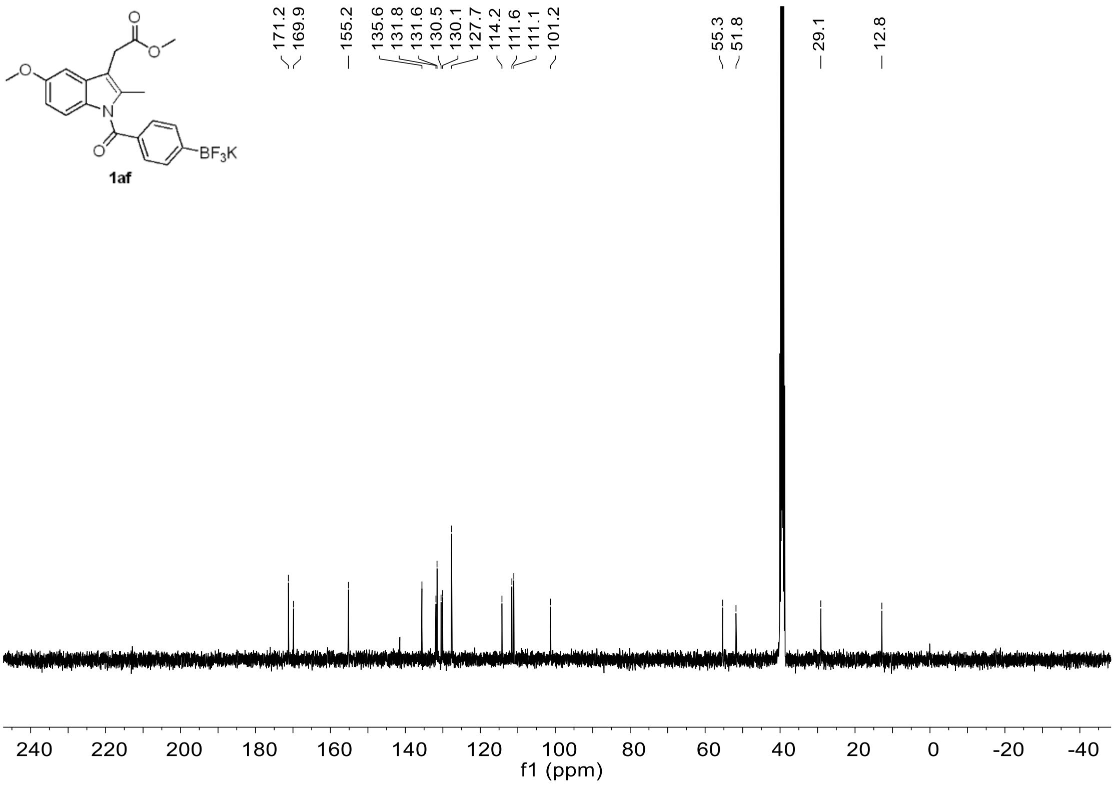

Supplementary Fig. 124. ^{13}C NMR spectrum (101 MHz, DMSO-d_6) of **1af**

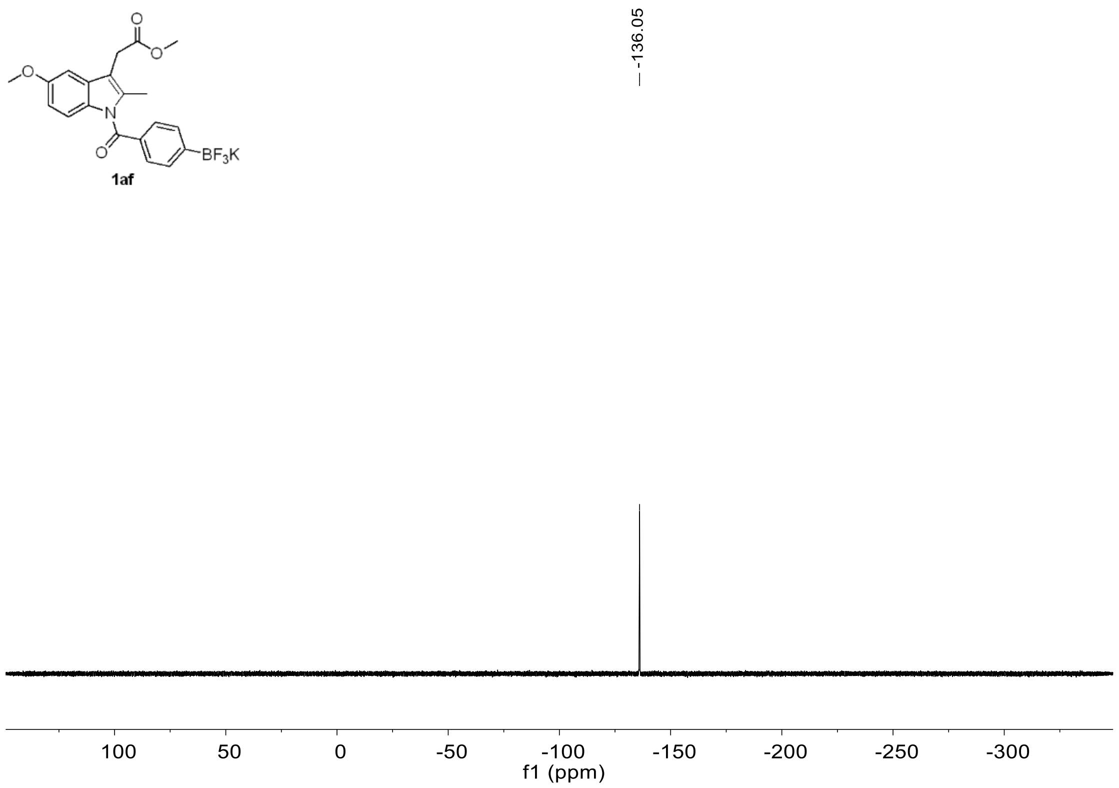

Supplementary Fig. 125. ^{19}F NMR spectrum (376 MHz, DMSO-d_6) of **1af**

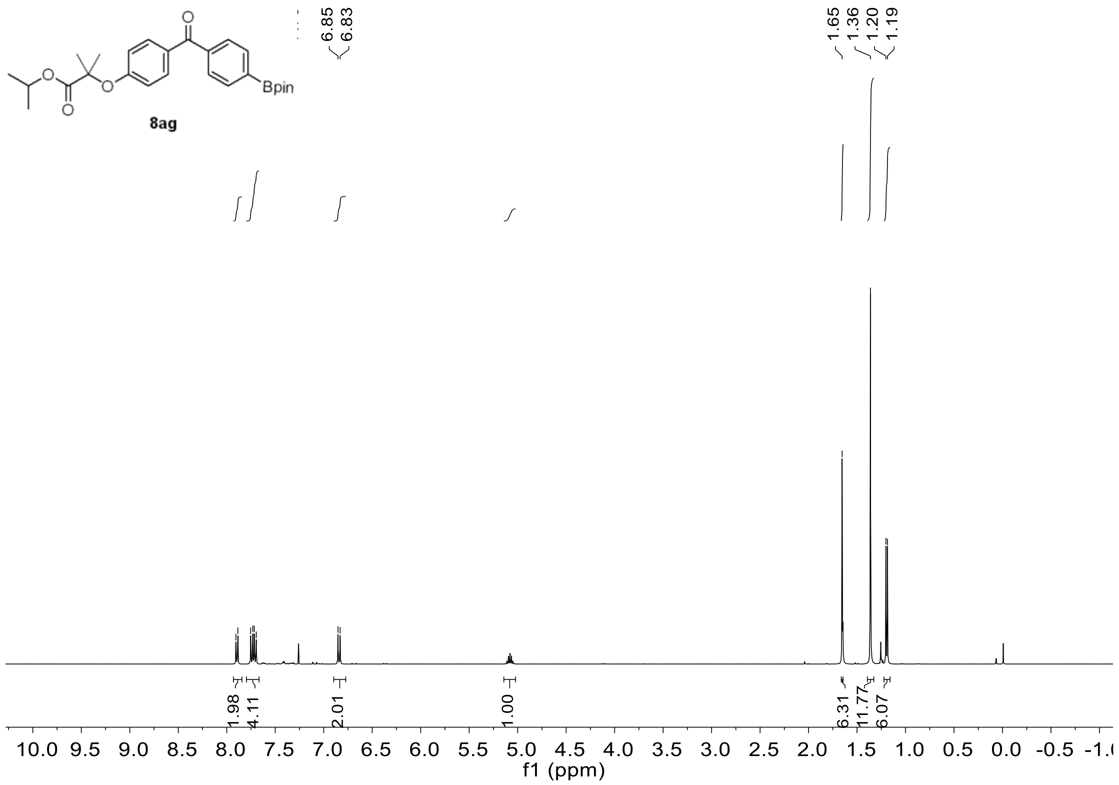

Supplementary Fig. 126. ^{1}H NMR spectrum (400 MHz, $CDCl_3$) of **8ag**

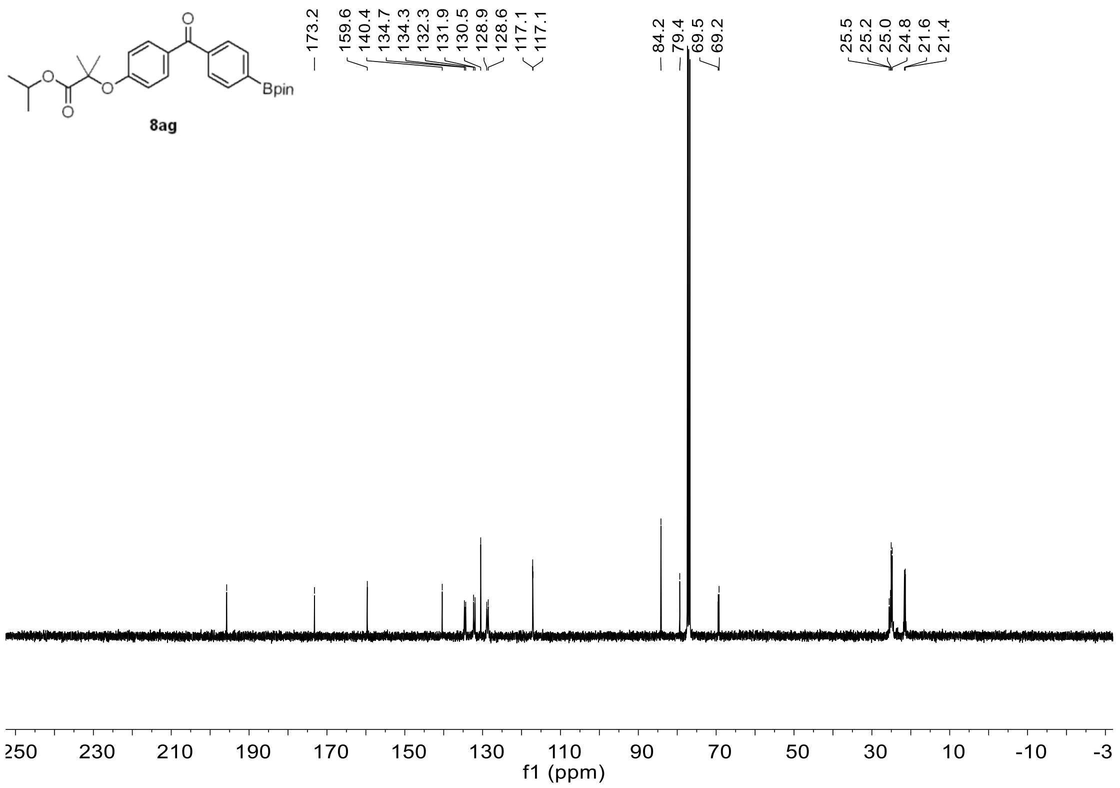

Supplementary Fig. 127. ^{13}C NMR spectrum (101 MHz, $CDCl_3$) of **8ag**

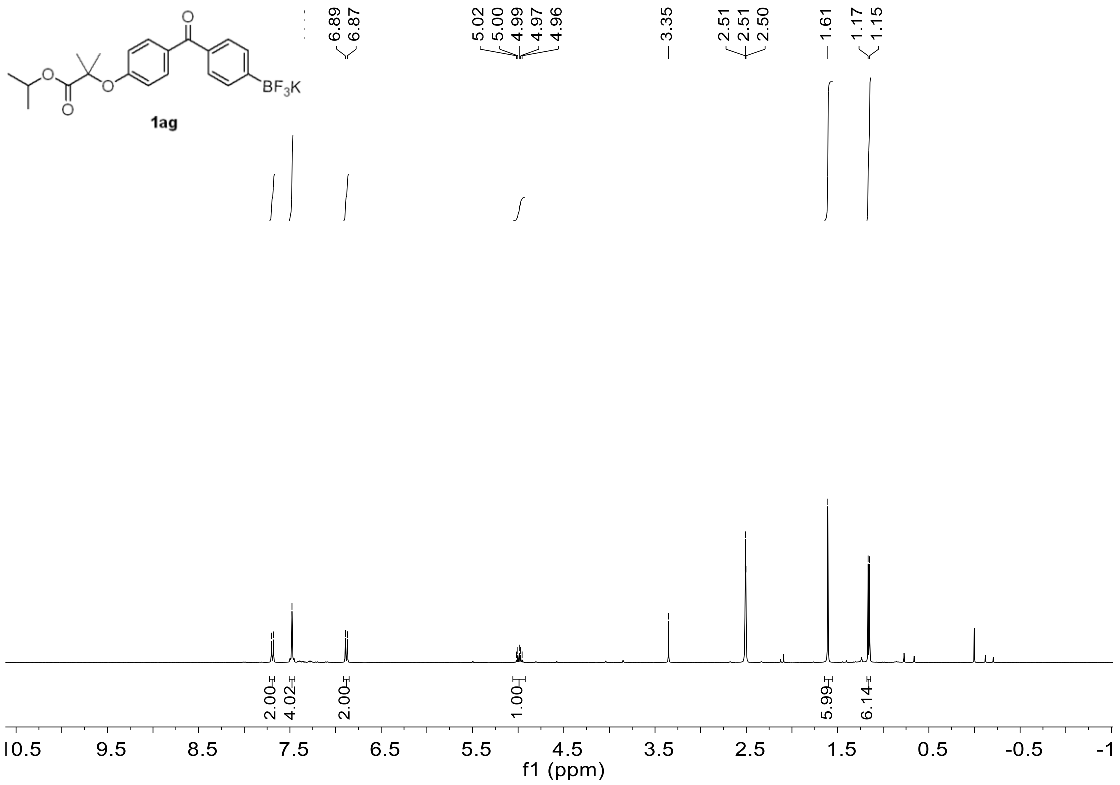

Supplementary Fig. 128. ^{1}H NMR spectrum (400 MHz, DMSO-d_6) of **1ag**

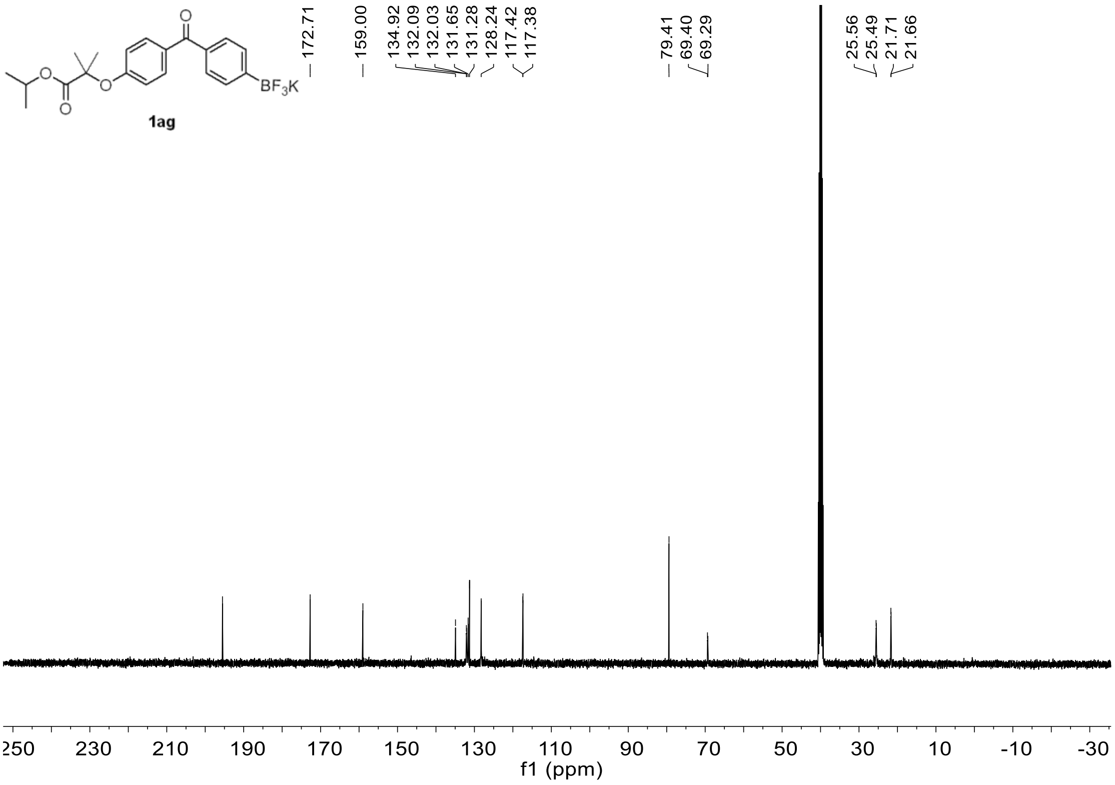

Supplementary Fig. 129. ^{13}C NMR spectrum (101 MHz, DMSO-d_6) of **1ag**

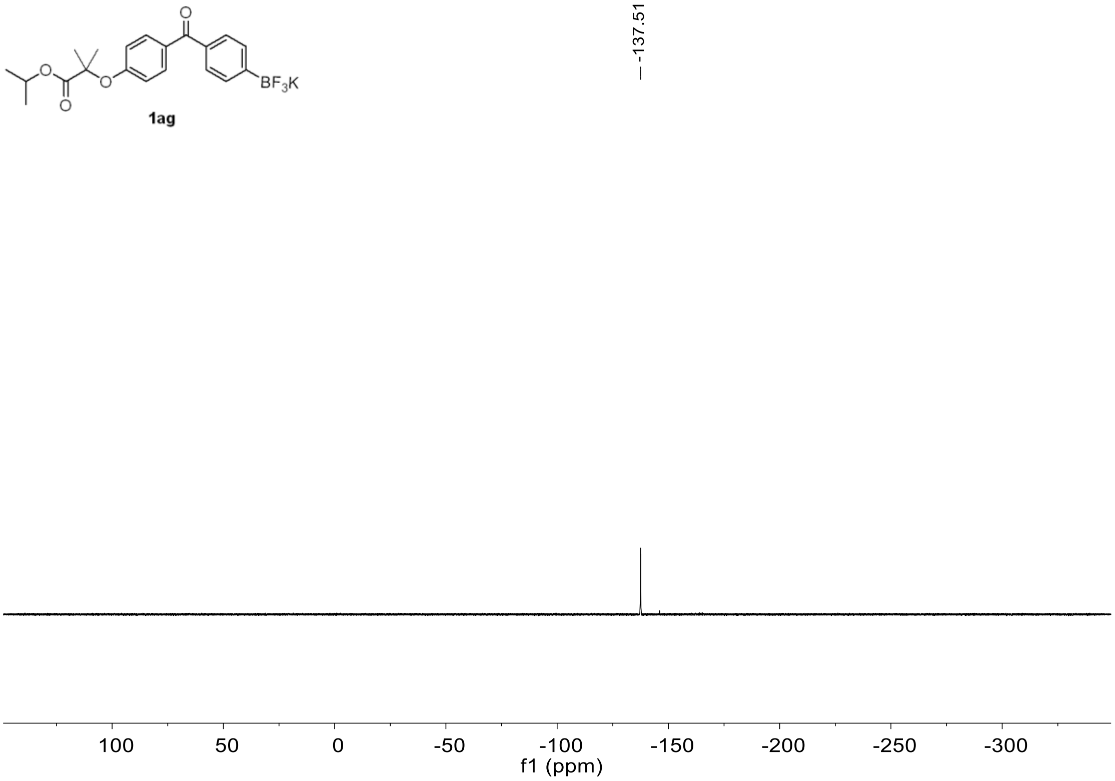

Supplementary Fig. 130. ^{19}F NMR spectrum (376 MHz, DMSO-d_6) of **1ag**

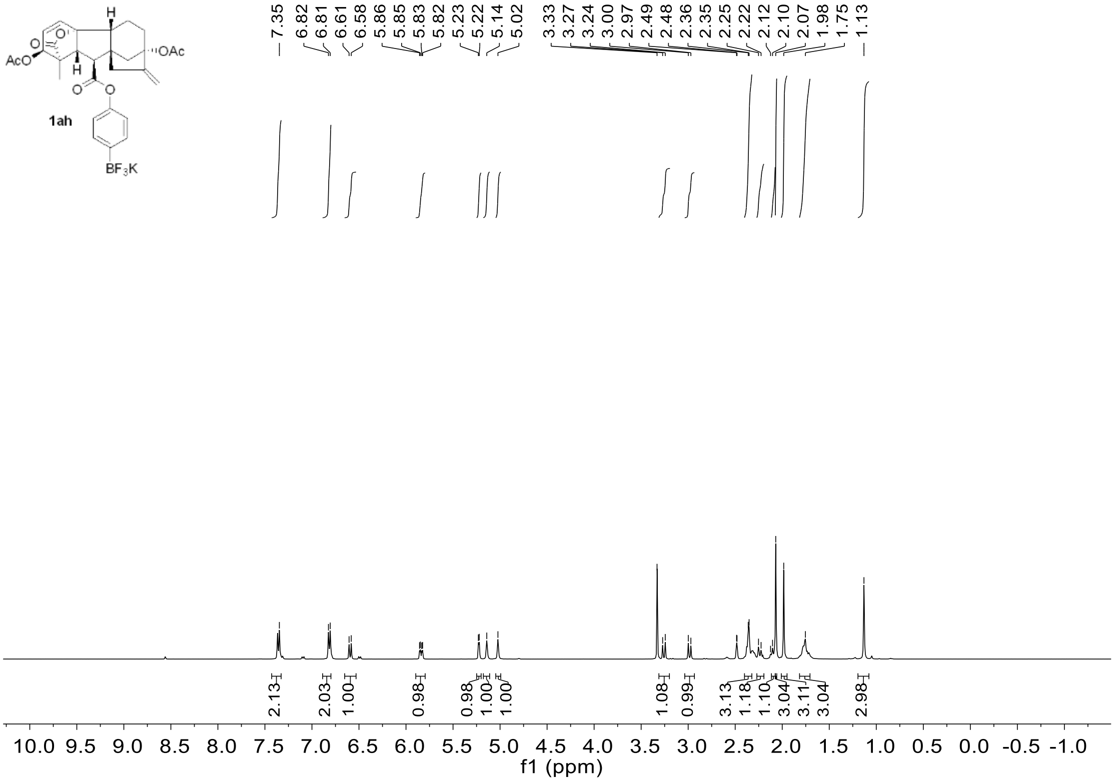

Supplementary Fig. 131. ^{1}H NMR (400 MHz, DMSO-d_6) of **1ah**

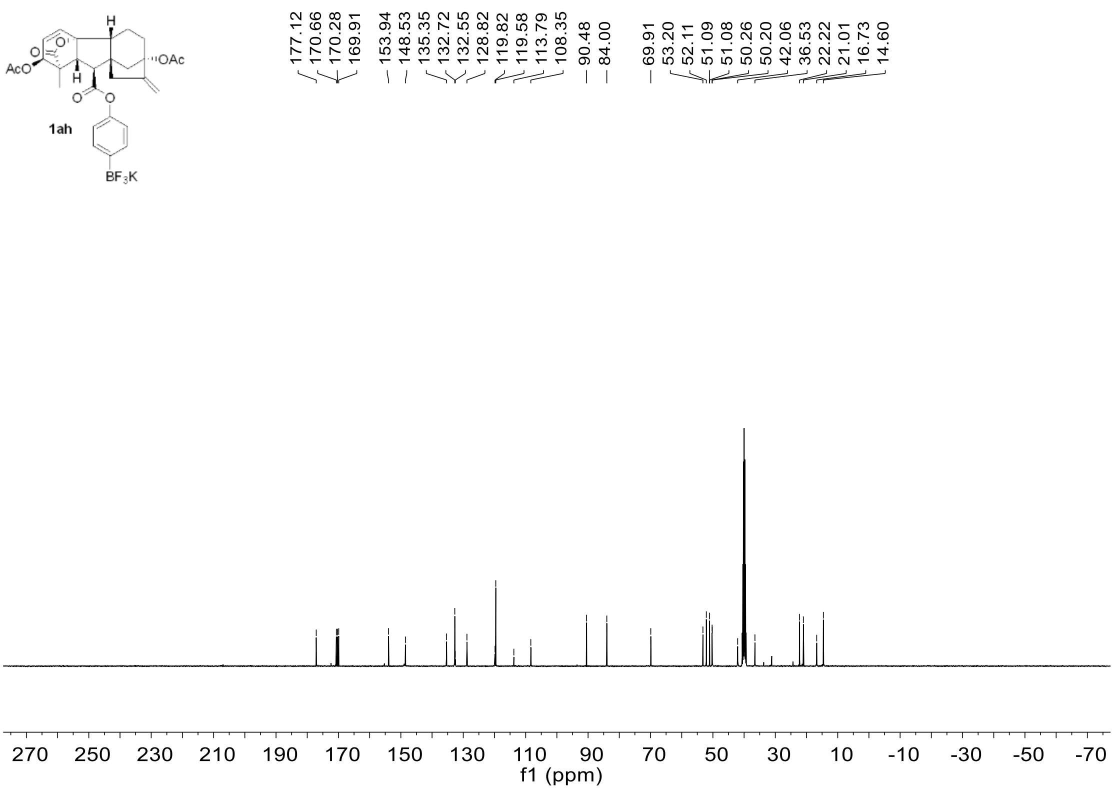

Supplementary Fig. 132. ^{13}C NMR (101 MHz, DMSO-d_6) of **1ah**

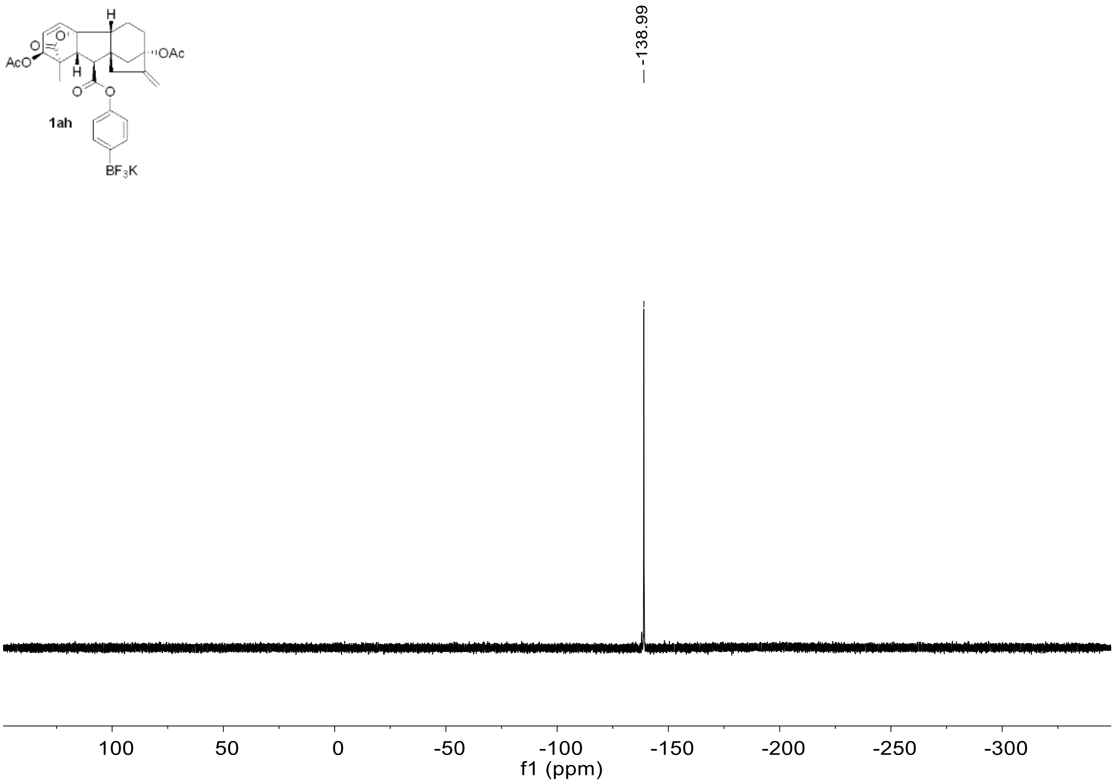

Supplementary Fig. 133. ^{19}F NMR (376 MHz, DMSO-d_6) of **1ah**

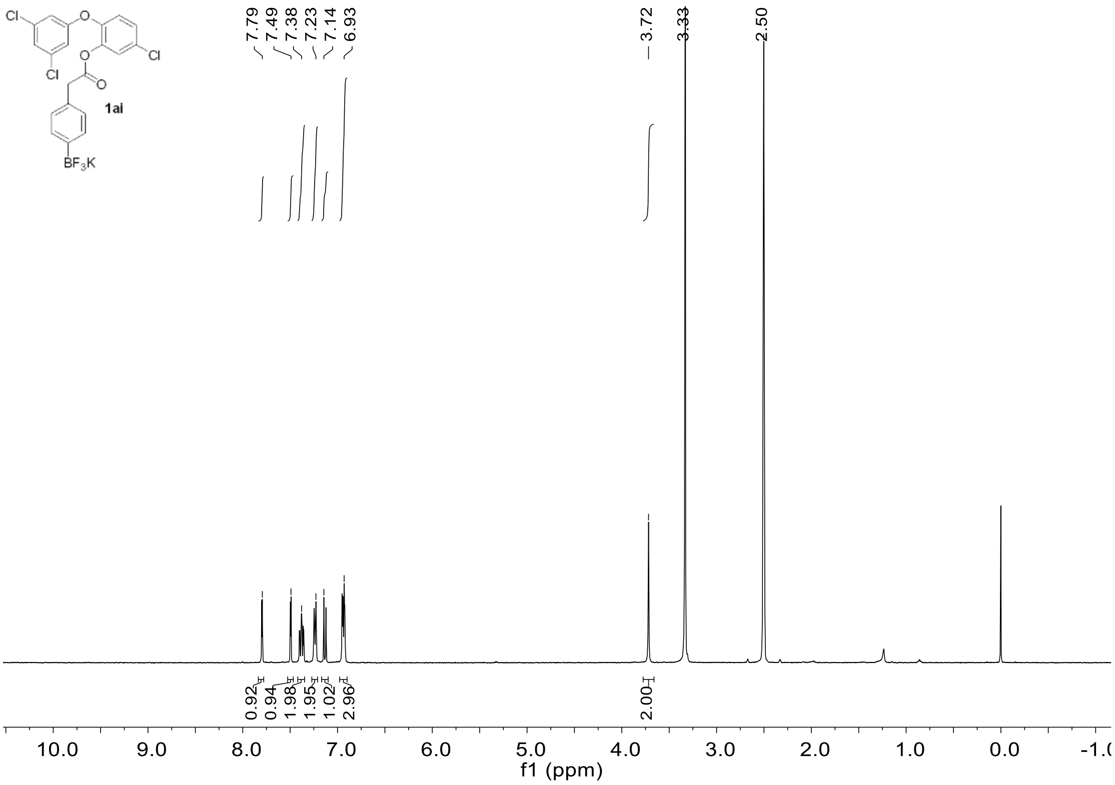

Supplementary Fig. 134. ^{1}H NMR (400 MHz, DMSO-d_6) of **1ai**

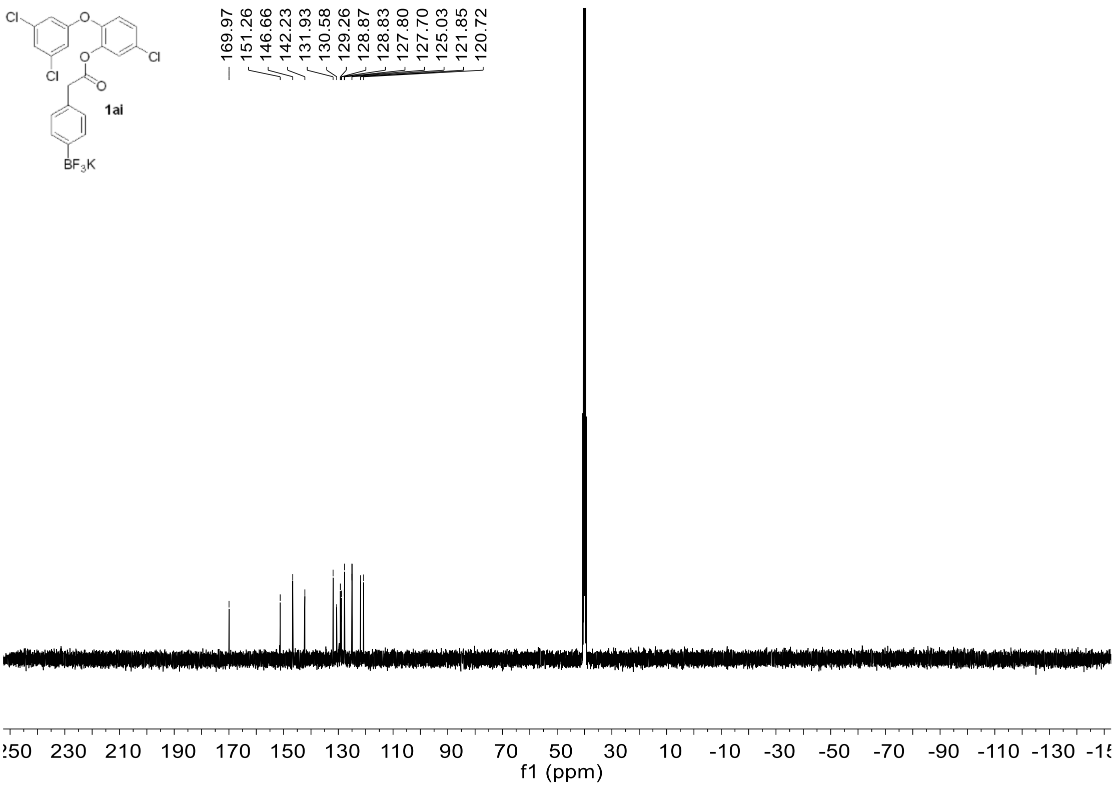

Supplementary Fig. 135. ^{13}C NMR (101 MHz, DMSO-d_6) of **1ai**

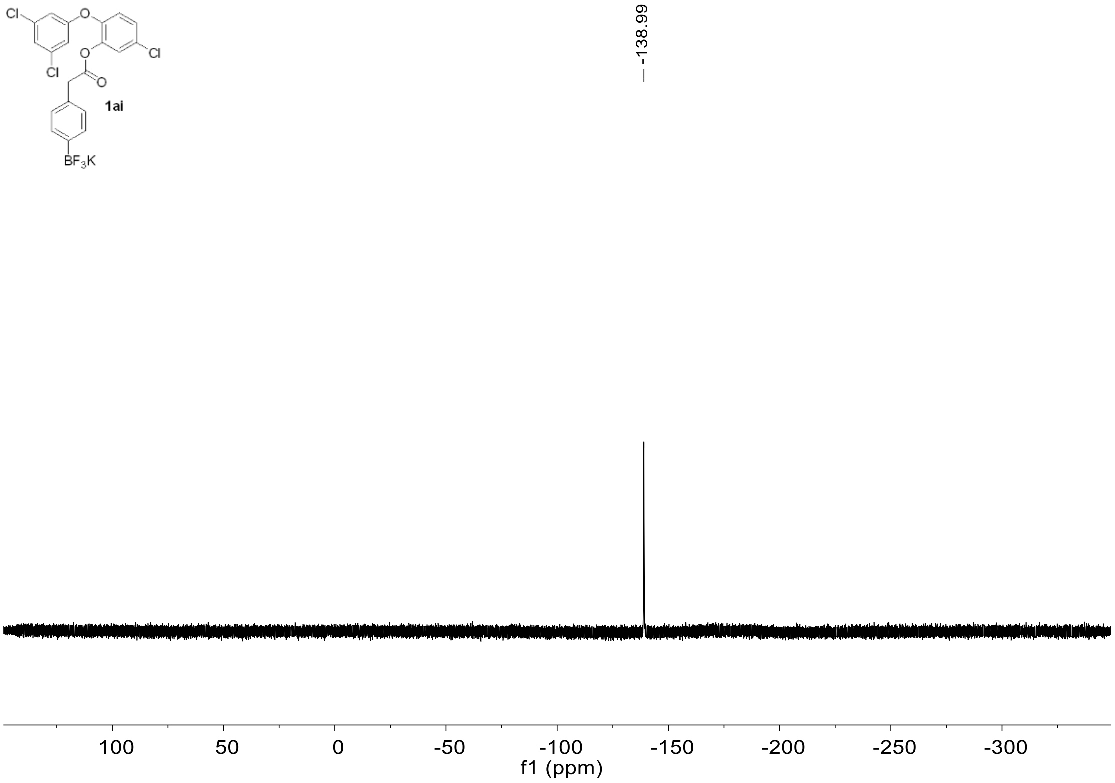

Supplementary Fig. 136. ^{19}F NMR (376 MHz, DMSO-d_6) of **1ai**

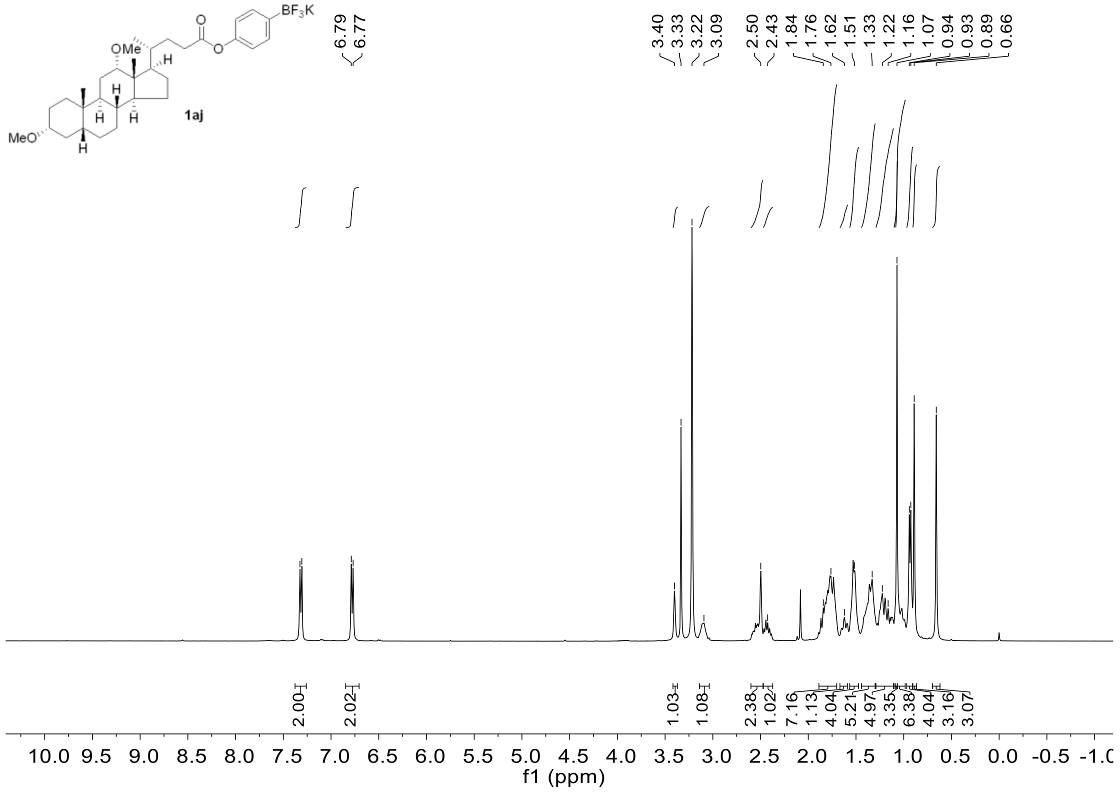

Supplementary Fig. 137. ^{1}H NMR (400 MHz, DMSO-d_6) of **1aj**

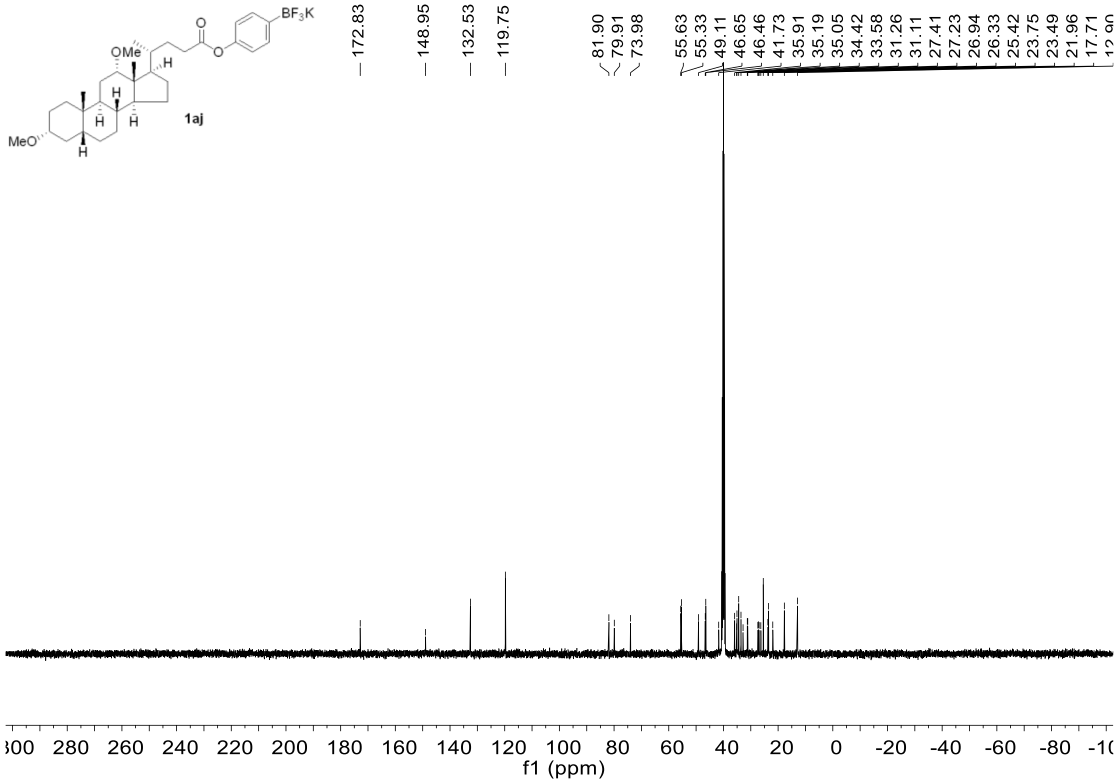

Supplementary Fig. 138. ^{13}C NMR (101 MHz, DMSO-d_6) of **1aj**

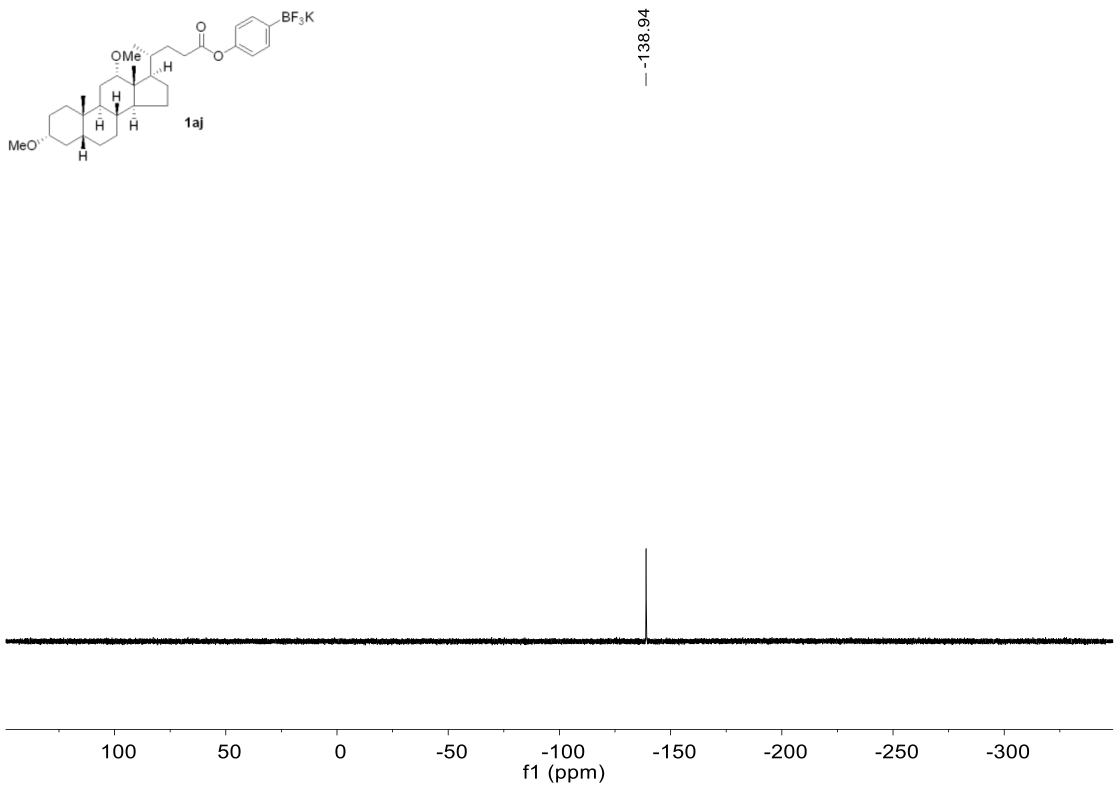

Supplementary Fig. 139. ^{19}F NMR (376 MHz, DMSO-d_6) of **1aj**

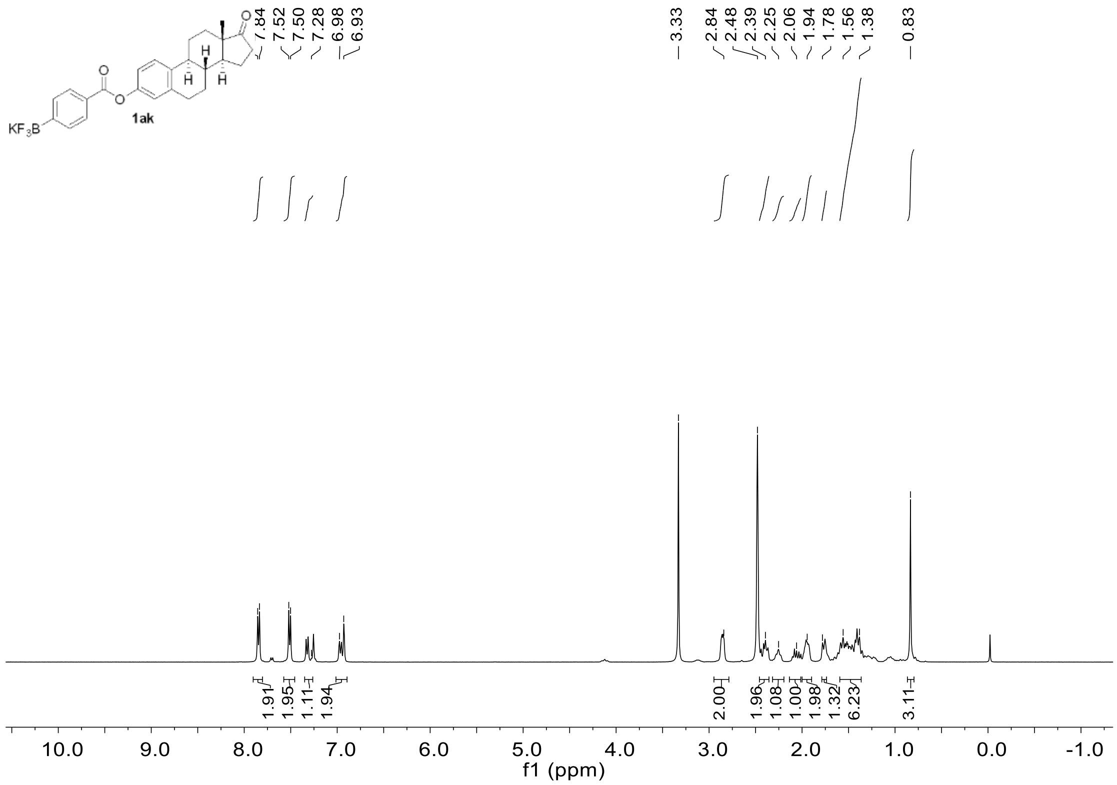

Supplementary Fig. 140. ^{1}H NMR (400 MHz, DMSO-d_6) of **1ak**

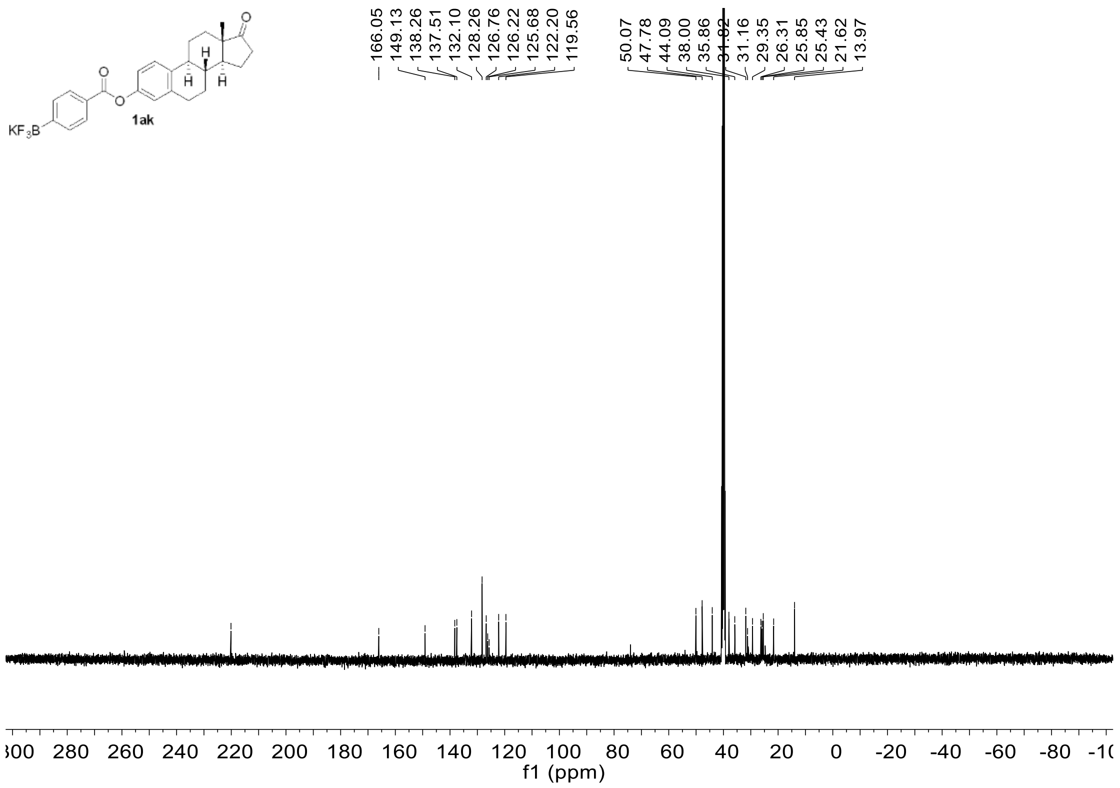

Supplementary Fig. 141. ^{13}C NMR (101 MHz, DMSO-d_6) of **1ak**

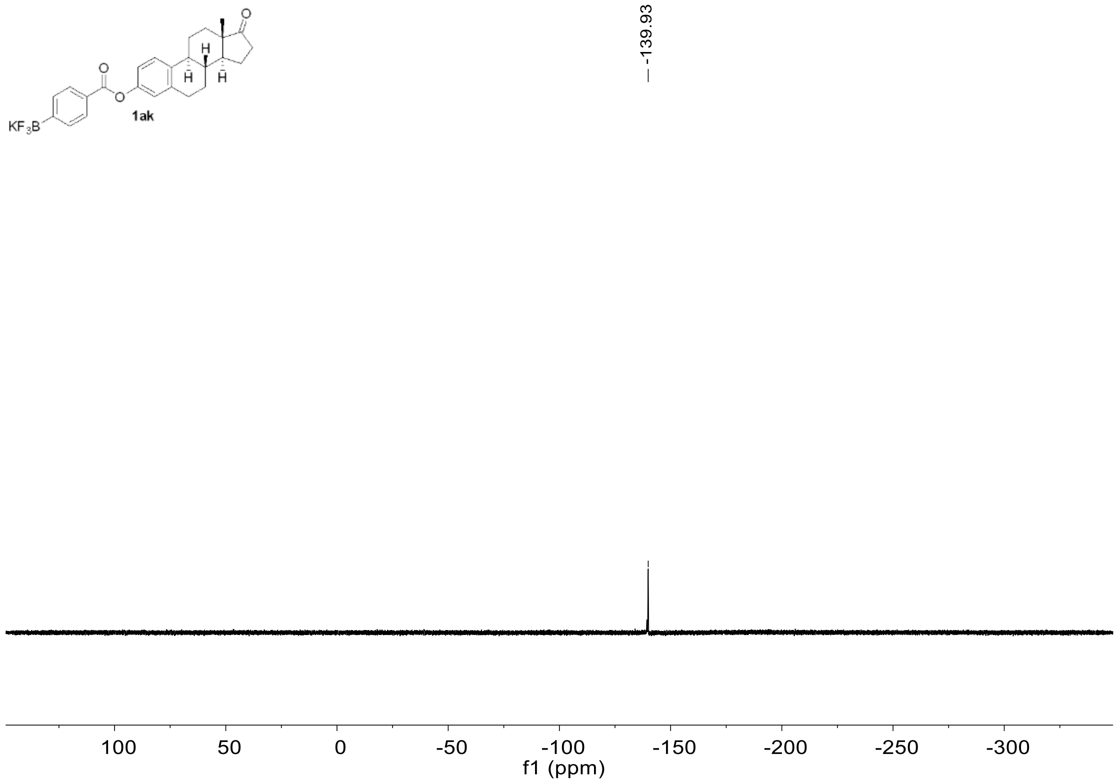

Supplementary Fig. 142. ^{19}F NMR (376 MHz, DMSO-d_6) of **1ak**

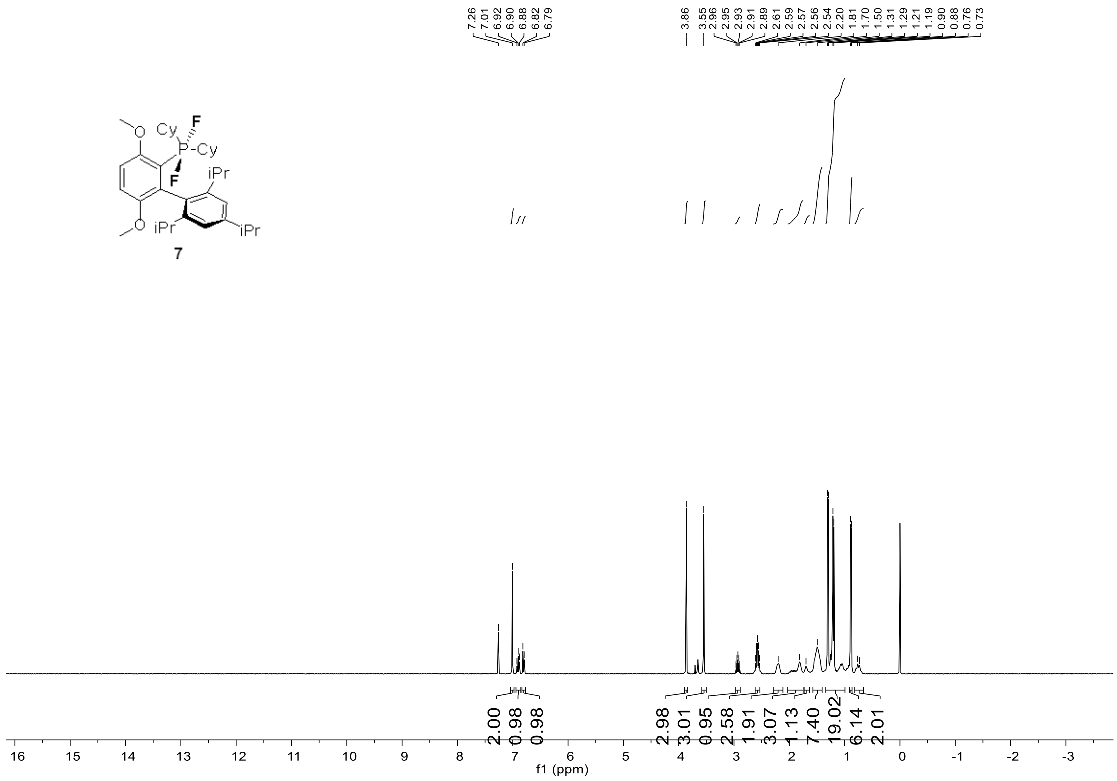

Supplementary Fig. 143. ^{1}H NMR spectrum (400 MHz, $CDCl_3$) of **7**

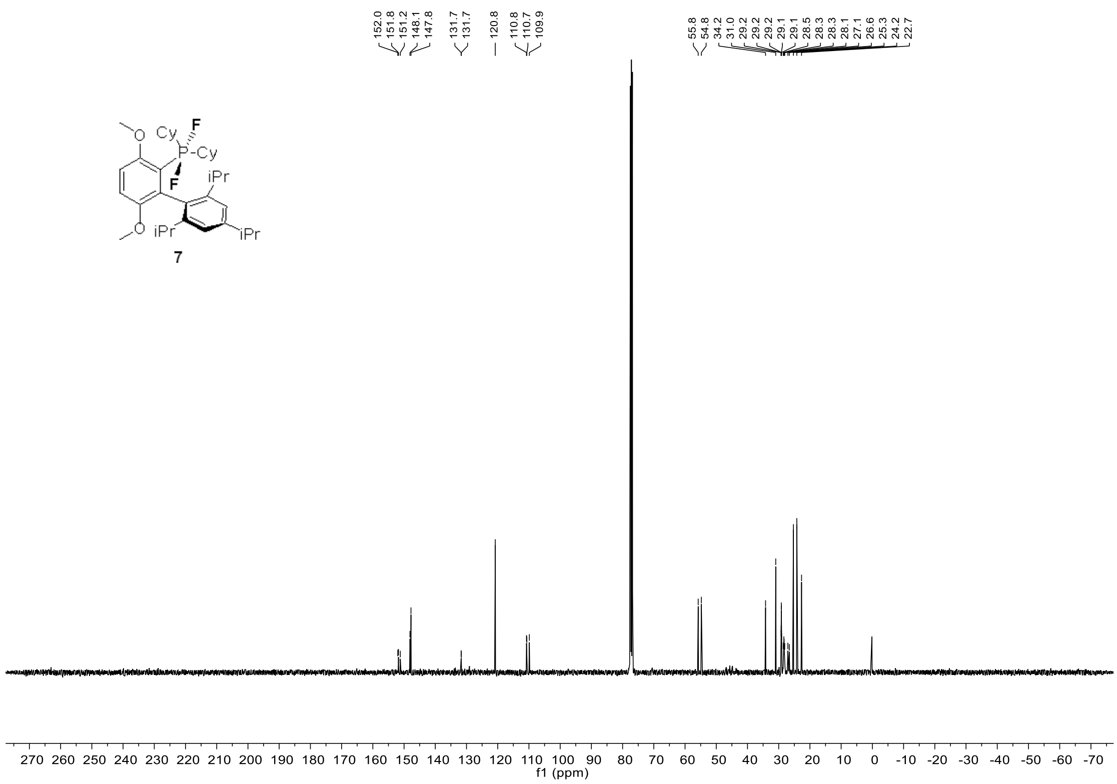

Supplementary Fig. 144. ^{13}C NMR spectrum (101 MHz, $CDCl_3$) of **7**

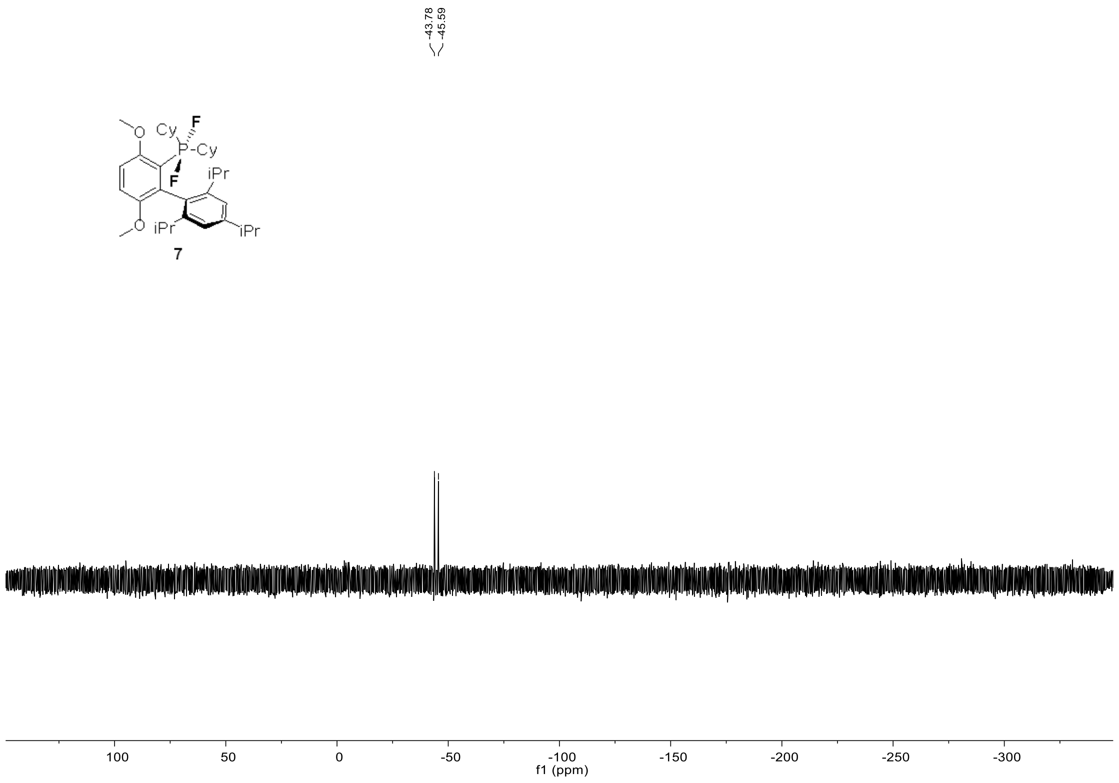

Supplementary Fig. 145. ^{19}F NMR spectrum (376 MHz, $CDCl_3$) of **7**

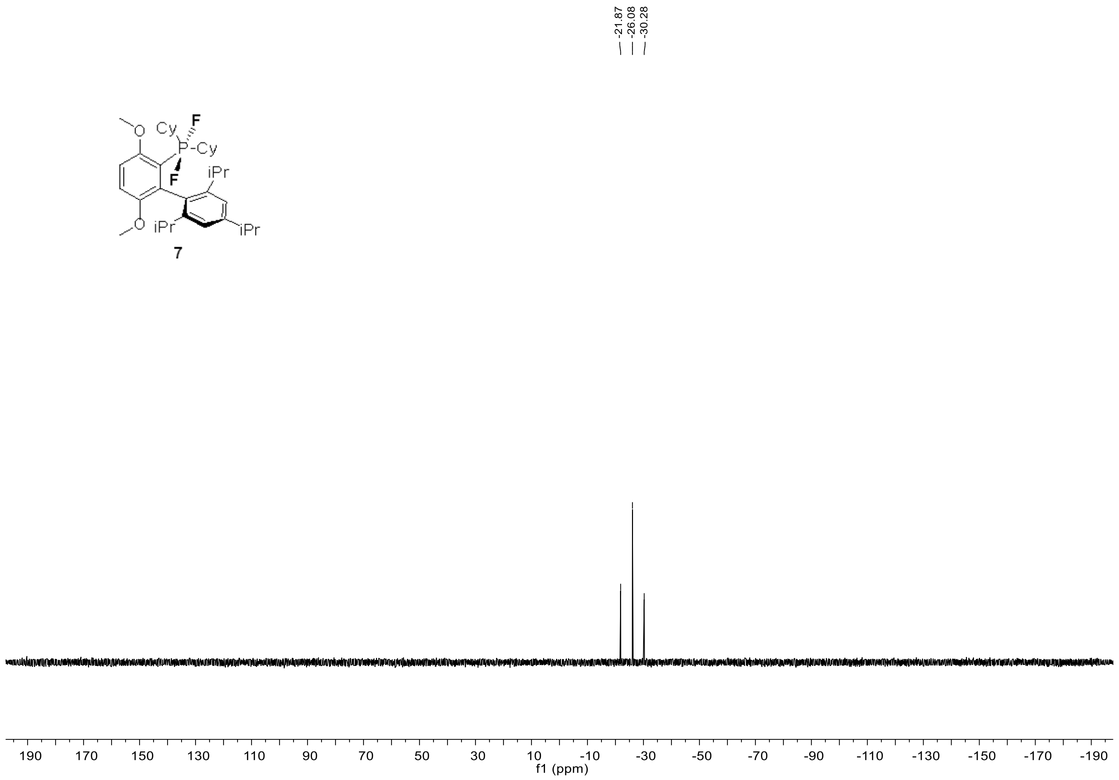

Supplementary Fig. 146. ^{31}P NMR spectrum (162 MHz, $CDCl_3$) of **7**

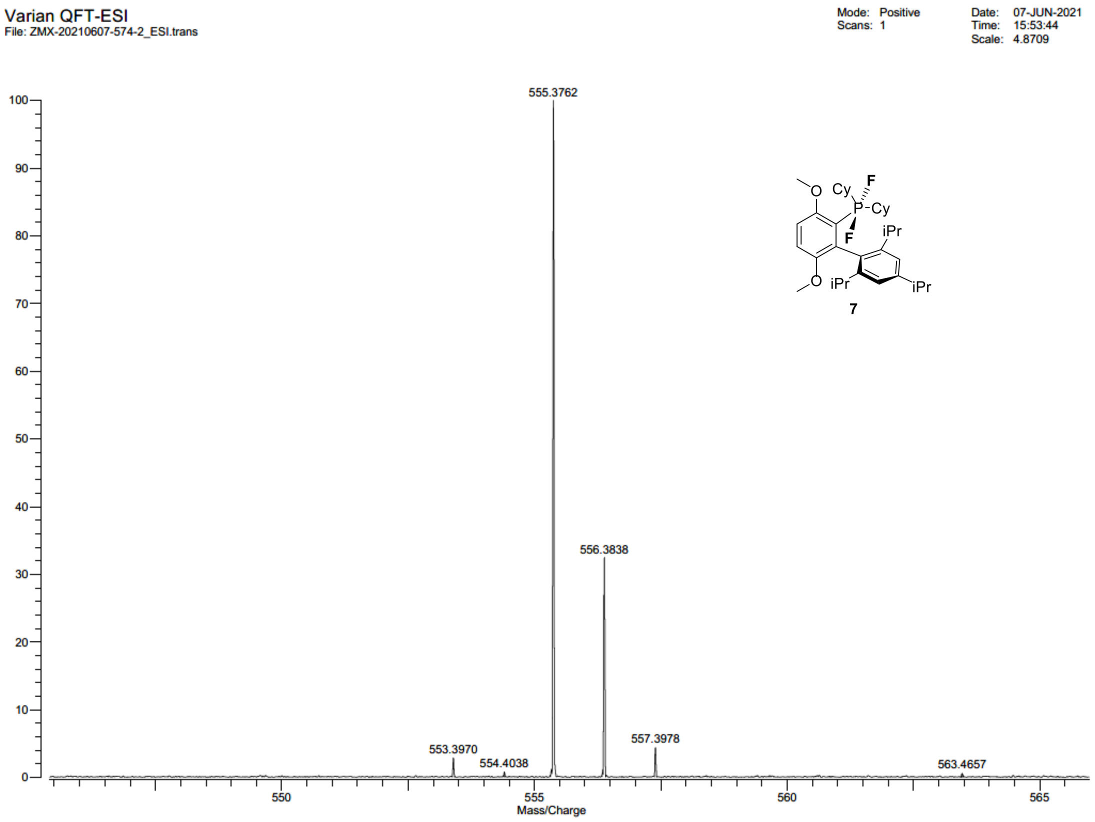

Supplementary Fig. 147. HRMS spectrum of **7**

Supplementary References

[1] Nikolaos F. N., Mary K. A., Elpida S., Anna T., Christoforos G. K. *Chem. Eur. J.* **2021**, *27*, 7915-7922.

[2] P. Veeraraghavan R., Henry J. H. *Org. Lett.* **2021**, *23*, 2938-2942.

[3] Wu, X., Wang H., Yang, Z., Tang, X., Ye Y., Wei S., Cheng C., Francis V. *Org. Chem. Front.* **2019**, *6*, 563.

[4] C. Liana A., Simge D., Jonathan M. J. W. *Org. Lett.* **2010**, *12*, 5096-5099.

[5] Wu, X., Mats L. *Org. Lett.* **2005**, *7*, 3327-3329.

[6] Naeem I., Eun, J. C. *J. Org. Chem.* **2016**, *81*, 1905-1911.

[7] Wu, L., Hao, Y., Liu, Y., Wang, Q. *Org. Biomol. Chem.* **2019**, *17*, 6762.

[8] Wei R., Motoki Y. *J. Org. Chem.* **2009**, *74*, 8332-8335.

[9] Liang, Y., Zhao, Z., Akihito T., Norio S. *Org. Lett.* **2021**, *23*, 847-852

[10] Tomomi, Y., Saori, T., Shunya, S., Ryu, S., Jiang, J., Miho, H., Akira, M. and Keiji, *M. Chem. Sci.*, **2020**, *11*, 5772–5778

[11] Wang, X., Wang, F., Huang, F., Ni, C, and Hu, J. *Org. Lett.* **2021**, *23*, 1764−1768

[12] Marie, G., Chloé, B. and Jean, F. P. *J. Org. Chem.* **2020**, *85*, 10253−10260

[13] Dai, J., Zhang, W., Shu, Y., Sun Y., Xu J., Feng, Y and Xu, H. *Chem. Commun.,* **2016**, *52*, 6793−6796

[14] Jiang, X. and Tang, P. *Org. Lett.* **2020**, *22*, 5135−5139

[15] Niwa, T., Uetake, Y., Isoda, M. *Nat Catal.* **2021**, *4*, 1080–1088.

[16] Jin, S., Hang. T. D., Graham C. H., Ru H., Viet D. N., Vu T. N., Hadi D. A., Kirk S. S., Oleg V. L. *J. Am. Chem. Soc.* **2020**, *142*, 1603–1613

[17] Da, Z., Peng, X., Tobias, R. *Chem.* **2019**, *5*, 97-107.

[18] Wu, J., Zhao, Q., Thomas C. W., Stefan V., Lu, Long., Véronique G., Shen, Q. *Angew. Chem. Int. Ed.* **2019**, *58*, 2413 –2417

[19] Frisch, M. J.; Trucks, G. W.; Schlegel, H. B.; Scuseria, G. E.; Robb, M. A.; Cheeseman, J. R.; Scalmani, G.; Barone, V.; Petersson, G. A.; Nakatsuji, H.; Li, X.; Caricato, M.; Bloino, J.; Gomperts, R.; Mennucci, B.; Hratchian, H. P.; Ortiz, J. V.; Izmaylov, A. F.; Sonnenberg, J. L.; Zakrzewski, V. G.; Rega, N.; Zheng, G.; Hada, M.; Ehara, M.; Toyota, K.; Fukuda, R.; Hasegawa, J.; Ishida, M.; Nakajima, T.; Honda, Y.; Kitao, O.; Nakai, H.; Vreven, T.; Montgomery Jr., J. A.; Peralta, J. E.; Ogliaro, F.; Bearpark, M. J.; Heyd, J. J.; Brothers, E. N.; Kudin, K. N.; Staroverov, V. N.; Keith, T. A.; Knox, J. E.; Cross, J. B.; Bakken, V.; Jaramillo, J.; Stratmann, R. E.; Yazyev, O.; Austin, A. J.; Pomelli, C.; Voth, G. A.; Salvador, P.; Dannenberg, J. J.; Dapprich, S.; Daniels, A. D.; Cioslowski, J.; Kobayashi, R.; Normand, J.; Raghavachari, K.; Rendell, A. P.; Burant, J. C.; Iyengar, S. S.; Tomasi, J.; Cossi, M.; Millam, J. M.; Klene, M.; Adamo, C.; Cammi, R.; Ochterski, J. W.; Martin, R. L.; Morokuma, K.; Farkas, O.; Foresman, J. B.; Fox, D. J. Gaussian 09 Rev. D.01, Wallingford, CT, 2013.

[20] Lee, C.; Yang, W.; Parr, R. G., Development of the Colle-Salvetti correlation-energy formula into a functional of the electron density. *Phys. Rev. B* **1988**, *37,* 785-789.

[21] Becke, A. D., Density-functional thermochemistry. III. The role of exact exchange. *J.*

Chem. Phys. **1993**, *98*, 5648-5652.

[22] Grimme, S.; Antony, J.; Ehrlich, S.; Krieg, H., A consistent and accurate ab initio parametrization of density functional dispersion correction (DFT-D) for the 94 elements H-Pu. *J. Chem. Phys.* **2010**, *132*, 154104-154122.

[23] Dolg, M.; Wedig, U.; Stoll, H.; Preuss, H., Energy-adjusted ab initio pseudopotentials for the first row transition elements. *J. Chem. Phys.* **1987**, *86*, 866-872.

[24] Weigend, F.; Ahlrichs, R., Balanced basis sets of split valence, triple zeta valence and quadruple zeta valence quality for H to Rn: Design and assessment of accuracy. *Phys. Chem. Chem. Phys.* **2005**, *7*, 3297-3305.

[25] Marenich, A. V.; Cramer, C. J.; Truhlar, D. G., Universal solvation model based on solute electron density and on a continuum model of the solvent defined by the bulk dielectric constant and atomic surface tensions. *J. Phys. Chem. B* **2009**, *113*, 6378-6396.

[26] Kenichi, F., The path of chemical reactions - the IRC approach. *Acc. Chem. Res.* **1981**, *14*, 363-368.

[27] Legault, C. Y., CYLview, version 1.0b; Université de Sherbrooke, 2009 (http://www.cylview.org).
