# Supplementary material for: Fluorocarbonylation via palladium/phosphine synergistic catalysis
Source: Nat Commun. 2023 Jul 31;14:4583. doi: 10.1038/s41467-023-40180-6 (PMC10390470; doi:10.1038/s41467-023-40180-6)
Supplement: Supplementary file 3 — Description of Additional Supplementary Files [file 41467_2023_40180_MOESM3_ESM.docx]

**Description of Additional Supplementary Files**

**File Name: Supplementary Data 1
Description:** Cartesian Coordinates of the Stationary Points.
