# Supplementary material for: Fluorocarbonylation via palladium/phosphine synergistic catalysis
Source: Nat Commun. 2023 Jul 31;14:4583. doi: 10.1038/s41467-023-40180-6 (PMC10390470; doi:10.1038/s41467-023-40180-6)
Supplement: Supplementary file 4 — Supplementary Data 1 [file 41467_2023_40180_MOESM4_ESM.pdf]

Supplementary Data 1

Cartesian Coordinates of the Stationary Points

BF_3

B	0.00000000	0.00000000	0.00000000
F	0.00000000	1.31826200	0.00000000
F	1.14164800	-0.65913100	0.00000000
F	-1.14164800	-0.65913100	0.00000000

CO

C	0.00000000	0.00000000	-0.65025600
O	0.00000000	0.00000000	0.48769200

COF_2

C	0.00000000	0.14313200	0.00000000
O	-0.00006300	1.32355300	0.00000000
F	1.06924600	-0.63590400	0.00000000
F	-1.06919000	-0.63600900	0.00000000

AgF

Ag	0.00000000	0.00000000	0.31794400
F	0.00000000	0.00000000	-1.66037600

KF

K	0.00000000	0.00000000	0.69967000
F	0.00000000	0.00000000	-1.47708100

cod

C	-0.03016700	1.70073700	-0.22348200
C	1.19669600	1.24373700	-0.50124200
H	1.79404900	1.84775300	-1.18647200
C	1.92088300	0.01075400	-0.01777000
H	2.72880000	0.32192300	0.66419300
H	2.43448900	-0.43181100	-0.88422300
C	1.09146600	-1.09531500	0.66982300
H	1.78686900	-1.88914300	0.97001600
C	-1.09142800	1.09526100	0.66982600
H	-1.78677000	1.88907600	0.97020700
C	-1.92091600	-0.01074600	-0.01773600
H	-2.72874000	-0.32196300	0.66431500
C	-1.19670900	-1.24369600	-0.50128300
H	-1.79398200	-1.84766600	-1.18662700

C	0.03013600	-1.70071900	-0.22345900
H	0.33085800	-2.61982900	-0.72743100
H	-0.33086000	2.61991100	-0.72734900
H	-2.43463900	0.43182900	-0.88412600
H	0.66220100	-0.71506800	1.59977100
H	-0.66203400	0.71491300	1.59966700

Pd(cod)F_2

Pd	0.79053300	-0.00005000	-0.00008500
C	-0.78050100	-1.00194400	1.19871900
C	-0.64449000	-1.65426700	-0.02103000
H	0.02245000	-2.51372500	-0.04362100
C	-1.50234000	-1.45850600	-1.25836000
H	-2.40606300	-2.08160900	-1.18685100
H	-0.93033700	-1.84415300	-2.10909500
C	-1.89041800	0.01429900	-1.54262400
H	-2.13018400	0.11417100	-2.60607700
C	-1.89025200	-0.01413300	1.54268600
H	-2.13026300	-0.11412800	2.60607300
C	-1.50180100	1.45859200	1.25870400
H	-2.40529400	2.08208400	1.18781500
C	-0.64438300	1.65434900	0.02108400
H	0.02264700	2.51375000	0.04352200
C	-0.78062600	1.00206500	-1.19865000
H	-0.19442000	1.39024800	-2.02913100
H	-0.19418300	-1.39007500	2.02914400
H	-0.92925200	1.84373800	2.10931000
H	-2.79973000	0.28573900	-0.99863400
H	-2.79948200	-0.28531900	0.99844200
F	2.12885200	1.25268900	-0.61659700
F	2.12875500	-1.25281400	0.61658000

1a

C	-2.67831300	1.21551200	-0.00308500
C	-1.28233000	1.19984500	-0.02366400
C	-0.55481000	-0.00000300	-0.02886700
C	-1.28236500	-1.19987100	-0.02368100
C	-2.67832000	-1.21550100	-0.00300500
C	-3.35164500	0.00002500	0.00988800
H	-3.24706600	2.14045000	-0.00075700
H	-0.74308500	2.14384200	-0.04563300
H	-0.74335400	-2.14405000	-0.04565000
H	-3.24708600	-2.14042300	-0.00047400
B	1.03432200	0.00000200	-0.00503200

F	1.61309600	-1.14304400	-0.66516300
F	1.61272900	1.15502300	-0.64427300
F	-4.70791600	0.00001600	0.03017100
F	1.61117400	-0.01217300	1.31841900
K	3.82178600	0.00009100	0.01051400

1a-AgF

C	-5.57794100	1.21501700	-0.00107200
C	-4.18127000	1.19928600	-0.02375200
C	-3.45195000	0.00033900	-0.03109300
C	-4.18074000	-1.19907800	-0.02382400
C	-5.57730700	-1.21543100	-0.00093600
C	-6.25179100	-0.00032100	0.01258000
H	-6.14663600	2.14022000	0.00237500
H	-3.64139000	2.14297400	-0.04608300
H	-3.64103500	-2.14296600	-0.04627100
H	-6.14561400	-2.14086800	0.00272300
B	-1.85741900	0.00050700	-0.01088400
F	-1.29306000	-1.13652100	-0.68569700
F	-1.29411500	1.16538000	-0.63723300
F	-7.60955600	-0.00073800	0.03412500
F	-1.29679100	-0.02778900	1.31571600
K	0.97115100	0.00007700	0.01328500
F	3.47435000	0.00013000	0.00540500
Ag	5.48741400	-0.00013600	0.00014900

2a

C	1.39731900	-1.21777100	0.00000300
C	2.09282300	-0.01156500	0.00000900
C	1.44860300	1.22419200	0.00000500
C	0.05944400	1.24700000	0.00000000
C	-0.66796700	0.04586000	0.00000300
C	0.00662500	-1.18449200	0.00000300
H	1.94862400	-2.15167900	-0.00000300
H	2.03861900	2.13414300	0.00000500
H	-0.48113400	2.18764700	0.00000100
H	-0.56086300	-2.10769800	0.00000400
C	-2.14025200	0.12648900	0.00000300
F	3.43614600	-0.03990700	-0.00001000
F	-2.72150200	-1.10759600	-0.00000300
O	-2.81957600	1.11085500	-0.00000500

L4

P	1.20099800	0.29811300	-0.81207000

O	3.07983700	-0.53712600	1.46725800
O	-2.26659700	-0.89711000	2.82777100
C	2.28759600	-1.06974300	-1.61292400
H	1.94485700	-1.03983400	-2.65856300
C	1.94301800	-2.46991400	-1.06964100
H	2.28370300	-2.54148400	-0.02928100
H	0.86138800	-2.61977700	-1.05690100
C	2.60997000	-3.58018900	-1.89627800
H	2.36000300	-4.56196000	-1.47273400
H	2.20247000	-3.56618300	-2.91807900
C	4.13169600	-3.39167600	-1.95340200
H	4.59344400	-4.16453500	-2.58150600
H	4.54677100	-3.51702300	-0.94183900
C	4.49045500	-1.99288100	-2.47308000
H	4.16611500	-1.90372600	-3.52074100
H	5.57943900	-1.85113200	-2.46996900
C	3.81811500	-0.88558500	-1.64130400
H	4.20302400	-0.91506600	-0.61642200
H	4.09183600	0.08951800	-2.05910400
C	2.45805100	1.67320300	-0.46096000
H	3.42267000	1.21996700	-0.21708300
C	2.61937200	2.51916500	-1.74277400
H	1.65222300	2.97883500	-1.99344000
H	2.88844100	1.88647200	-2.59865800
C	3.67525500	3.62021100	-1.55568400
H	4.65748800	3.15213600	-1.39156500
H	3.76190500	4.22017000	-2.47098600
C	3.33564900	4.51862500	-0.35761600
H	2.40763300	5.06853400	-0.57437700
H	4.12017100	5.27171800	-0.20769100
C	3.13667800	3.68776500	0.91859700
H	2.84715500	4.33758700	1.75507200
H	4.09226300	3.21970900	1.19950100
C	2.07990400	2.58891500	0.71768900
H	1.97061900	2.00685300	1.63855400
H	1.10989400	3.06234200	0.52421400
C	0.79347000	-0.34629000	0.89555900
C	1.77406800	-0.64741100	1.87222900
C	1.41279600	-1.02959200	3.16030700
H	2.16175200	-1.25698900	3.90963900
C	0.06251200	-1.12142500	3.50932300
H	-0.19747200	-1.41231200	4.52023900
C	-0.91948600	-0.84218800	2.56534100
C	-0.56625600	-0.46621300	1.24671600

C	4.11142900	-0.76363400	2.41091500
H	4.09220300	-1.79267200	2.79546400
H	4.04897400	-0.06213400	3.25404500
H	5.04727500	-0.59949800	1.87287700
C	-2.68247800	-1.25187100	4.13508200
H	-2.35130600	-2.26430700	4.40555300
H	-3.77352200	-1.22296700	4.11747300
H	-2.31280100	-0.53877800	4.88507100
C	-1.69606300	-0.21476800	0.28316000
C	-2.27661300	-1.29301300	-0.41597400
C	-3.36280800	-1.04500700	-1.26358500
H	-3.81242300	-1.87410900	-1.80483500
C	-3.90073000	0.22980900	-1.42861000
C	-3.32341700	1.27889600	-0.70835200
H	-3.73950100	2.27757900	-0.80863800
C	-2.23613500	1.08263300	0.14471900
C	-1.79880400	-2.72941400	-0.23004600
H	-0.82633200	-2.69862900	0.27131600
C	-2.76267200	-3.50338800	0.69105800
H	-2.87567100	-2.99036000	1.64988900
H	-2.39371900	-4.52046700	0.87417300
H	-3.75544300	-3.58041200	0.23081400
C	-1.61134100	-3.47629500	-1.56332000
H	-2.57189700	-3.68374500	-2.04882200
H	-1.11686200	-4.44010700	-1.39294600
H	-1.00300200	-2.89799500	-2.26668400
C	-5.08546700	0.46673500	-2.35248800
H	-5.35072000	-0.50065600	-2.80133000
C	-6.31605800	0.97123900	-1.57650800
H	-6.59172200	0.27336100	-0.77825700
H	-7.17746600	1.08885400	-2.24530000
H	-6.11680900	1.94523500	-1.11386200
C	-4.72631800	1.42834400	-3.50004200
H	-4.46648800	2.42121000	-3.11403400
H	-5.57271600	1.54731000	-4.18745200
H	-3.86735300	1.05767300	-4.06974900
C	-1.70168900	2.25685700	0.95569500
H	-0.72208200	1.97283300	1.35066400
C	-2.62030600	2.52909100	2.16289900
H	-3.61743800	2.83899500	1.82605600
H	-2.21085900	3.32795800	2.79400000
H	-2.73702100	1.62542100	2.76886800
C	-1.50544800	3.52834200	0.11327900
H	-0.89502800	3.32343500	-0.77273400

H	-1.00584800	4.30390400	0.70643000
H	-2.46078500	3.94545300	-0.22540100

5a

Pd	9.14668200	11.09671300	9.72556400
P	6.91414400	10.85003900	9.34253000
F	11.36823200	11.00341900	7.72389700
F	13.03002800	11.89278100	8.84639900
F	12.57293700	9.76744100	9.06369700
F	10.00953600	5.02157500	9.49861300
O	11.20620900	11.30235500	9.95771800
O	3.96860000	11.64865300	9.25816200
O	7.32199500	15.85616700	10.50935800
C	11.98393200	11.00334700	8.98365500
C	9.41012600	9.11572900	9.60044900
C	9.46433800	8.43650000	8.37862500
H	9.37057700	8.98036900	7.44429600
C	9.66656900	7.05294300	8.33651100
H	9.71094800	6.51301100	7.39570500
C	9.82867800	6.36300200	9.53099500
C	9.82104300	7.01426100	10.75922900
H	9.97818900	6.44399200	11.66957300
C	9.61725300	8.39735600	10.78594500
H	9.62441400	8.90614800	11.74553800
C	6.61597600	10.25023900	7.59269100
H	7.06513000	9.24997600	7.65683300
C	7.43335100	11.04350900	6.55683600
H	6.99635100	12.04462200	6.44274000
H	8.46155100	11.18315500	6.90223700
C	7.42289100	10.33157100	5.19551200
H	7.99329500	10.92035000	4.46656000
H	7.94054100	9.36593900	5.29375800
C	5.99043000	10.09390500	4.69613300
H	6.00127200	9.53678200	3.75106600
H	5.51819400	11.06512600	4.48484200
C	5.15263400	9.34994300	5.74667000
H	5.55396300	8.33439600	5.88154700
H	4.11807900	9.23476300	5.39836900
C	5.16643000	10.08211800	7.10017700
H	4.70873600	11.07104300	6.98340100
H	4.55732100	9.54155900	7.83087300
C	5.94261700	9.66848000	10.43154900
H	4.89151300	9.93779800	10.29626300
C	6.11109700	8.18446500	10.03342400

H	7.16554700	7.90068600	10.06499600
H	5.76689200	8.01909400	9.00722100
C	5.31908800	7.27138700	10.98464200
H	4.24242100	7.47404900	10.87744800
H	5.47503700	6.22502600	10.69439700
C	5.73131200	7.49154300	12.44603100
H	6.79057200	7.22062900	12.56385200
H	5.15694300	6.83403200	13.11057400
C	5.53711500	8.96004800	12.84415200
H	5.85871300	9.12668400	13.88015000
H	4.46618500	9.20942800	12.80115400
C	6.31618400	9.89649200	11.90882100
H	6.12580900	10.93881200	12.18784700
H	7.39009700	9.72422200	12.03648200
C	6.14885500	12.51822100	9.60219700
C	4.74800000	12.73566700	9.54096800
C	4.21673500	14.00006800	9.77751300
H	3.14828600	14.17201600	9.73026800
C	5.05847000	15.06694800	10.09452900
H	4.62187000	16.04065800	10.28161700
C	6.43320800	14.87010700	10.17888700
C	6.99781800	13.59444900	9.92624400
C	2.57717500	11.82561400	9.04914200
H	2.38110700	12.53916100	8.23797900
H	2.07089600	12.16431300	9.96262100
H	2.19411800	10.84310300	8.76752700
C	6.83169600	17.16770800	10.73467100
H	6.33114700	17.56915800	9.84286300
H	7.70913900	17.77539500	10.96064300
H	6.13847600	17.19905700	11.58641300
C	8.50173800	13.52351900	10.06829600
C	9.33091400	13.97297500	8.99513000
C	10.66932400	14.26848600	9.25247500
H	11.31214800	14.56414200	8.42932900
C	11.22506600	14.16923600	10.52926700
C	10.39646800	13.77139400	11.58019500
H	10.81115500	13.68977100	12.57922400
C	9.05794200	13.44028600	11.38313600
C	8.77072100	14.18876600	7.59523400
H	7.83427800	13.62859100	7.52854500
C	8.43895100	15.67591800	7.35613700
H	7.71476000	16.05029500	8.08305100
H	8.02854400	15.81663200	6.34867800
H	9.34614400	16.28588000	7.44170700

C	9.71249900	13.67432500	6.49051900
H	10.55506900	14.35879500	6.33752300
H	9.17244500	13.60737700	5.53865600
H	10.12772300	12.69466900	6.73571500
C	12.68726300	14.50917400	10.75880400
H	13.17747200	14.49483100	9.77765200
C	12.81560100	15.93014200	11.34287500
H	12.35140900	16.67559600	10.68672500
H	13.86972400	16.19993200	11.47954500
H	12.32160700	15.99001100	12.32086800
C	13.40653300	13.47710900	11.64331500
H	13.03171600	13.49721400	12.67396400
H	14.47924900	13.70043200	11.68153300
H	13.26766700	12.47124500	11.24279100
C	8.20661400	13.05640500	12.58710700
H	7.24464400	12.69500700	12.21408200
C	7.92477600	14.28418000	13.47464500
H	8.85754100	14.68233000	13.89125800
H	7.26886700	14.01257800	14.31071500
H	7.44832400	15.08276700	12.89921800
C	8.84193800	11.92007300	13.40836700
H	9.11962000	11.08067800	12.76405700
H	8.13891400	11.56098100	14.16955500
H	9.74872200	12.25484100	13.92431900

INT1

P	1.33911500	0.31778900	-0.48056000
O	3.05448600	-0.81376700	1.52213500
O	-2.23546000	-1.83132000	2.71358600
C	2.37582500	-0.85219200	-1.52343400
H	1.95365100	-0.67035800	-2.51828400
C	2.15034100	-2.34076100	-1.19377500
H	2.55621000	-2.56470900	-0.20072300
H	1.08208100	-2.56782800	-1.16635100
C	2.84818000	-3.22879100	-2.23559700
H	2.69341800	-4.28510000	-1.98169200
H	2.37993000	-3.06895300	-3.21712400
C	4.34757300	-2.91150900	-2.32475100
H	4.82610900	-3.53140700	-3.09319700
H	4.82712400	-3.16809000	-1.36778200
C	4.57953600	-1.42291600	-2.62018800
H	4.19044100	-1.18771000	-3.62106400
H	5.65341000	-1.19705600	-2.63851600
C	3.87915200	-0.52318000	-1.58742700

H	4.32926700	-0.67986000	-0.60033800
H	4.03604700	0.52732500	-1.85360100
C	2.38964300	1.73186600	0.18274000
H	3.35697600	1.27900400	0.42170300
C	2.61678100	2.86874000	-0.84009600
H	1.65107900	3.30994800	-1.10579200
H	3.03744900	2.48127400	-1.77061600
C	3.54473400	3.94155100	-0.24828600
H	4.53651100	3.50269400	-0.05934200
H	3.69137100	4.74092200	-0.98533100
C	2.98800200	4.51473900	1.06088400
H	2.04489400	5.03998100	0.85161900
H	3.67822500	5.25664700	1.48205000
C	2.72226900	3.39313100	2.07223900
H	2.27467800	3.79754500	2.98908000
H	3.67746700	2.93088000	2.36419000
C	1.80061500	2.31272400	1.48626400
H	1.64034200	1.52819000	2.23118700
H	0.82224000	2.75437500	1.26815200
C	0.75279800	-0.62583400	1.00669700
C	1.76363000	-1.10023100	1.88525100
C	1.43987400	-1.80701400	3.03522000
H	2.21071300	-2.16741900	3.70539800
C	0.10145100	-2.06432400	3.33910300
H	-0.13251800	-2.61959900	4.23932900
C	-0.90342700	-1.61352100	2.49120700
C	-0.59658400	-0.88352900	1.31362000
C	4.12087100	-1.27560900	2.33648100
H	4.12479600	-2.37104500	2.40835600
H	4.07225400	-0.84286100	3.34405800
H	5.03563300	-0.94290300	1.84283800
C	-2.61968200	-2.55637100	3.87041000
H	-2.20782100	-3.57492700	3.86417300
H	-3.70909400	-2.60771500	3.83706600
H	-2.30824600	-2.04338900	4.79077000
C	-1.75546700	-0.45195600	0.46628900
C	-2.29971400	-1.35270100	-0.46990100
C	-3.38005400	-0.93528500	-1.24835200
H	-3.78460400	-1.60949300	-1.99909600
C	-3.91915400	0.34402500	-1.13815900
C	-3.40481900	1.19462300	-0.15750600
H	-3.82966300	2.18710800	-0.04481200
C	-2.34093800	0.81727100	0.66181500
C	-1.75328100	-2.76375600	-0.65484900

H	-0.81752600	-2.84095000	-0.09045500
C	-2.72480800	-3.80260100	-0.06256100
H	-2.91925600	-3.59430300	0.99396300
H	-2.31399700	-4.81616800	-0.15331300
H	-3.68442500	-3.77914700	-0.59296400
C	-1.43094800	-3.08389100	-2.12657300
H	-2.34598900	-3.17754000	-2.72298800
H	-0.89543000	-4.03923000	-2.19832600
H	-0.82640500	-2.29077500	-2.57342800
C	-4.96824500	0.82633300	-2.12517000
H	-5.29262800	-0.04440000	-2.71193700
C	-6.21410200	1.41937700	-1.44699400
H	-6.67540400	0.69768800	-0.76331100
H	-6.96011200	1.70839900	-2.19711500
H	-5.96550700	2.31688700	-0.86847000
C	-4.32475000	1.83332700	-3.09963400
H	-4.02633500	2.74502600	-2.56796800
H	-5.02627200	2.11585300	-3.89462900
H	-3.42192100	1.40970900	-3.55173700
C	-1.89044900	1.74157500	1.78828100
H	-0.87820700	1.44668000	2.08277200
C	-2.79630400	1.54406700	3.02127000
H	-3.82623300	1.83920600	2.78629500
H	-2.44958400	2.15770600	3.86261200
H	-2.81246900	0.49553700	3.33150600
C	-1.84195200	3.22722500	1.39224700
H	-1.28927100	3.37157400	0.46091500
H	-1.36119800	3.81228600	2.18598200
H	-2.84792400	3.64028300	1.25425900
C	0.04355900	0.94106800	-1.92105800
F	1.09227800	1.50509300	-2.74956900
F	-0.52892800	2.09946700	-1.35595500
O	-0.66053900	0.10112400	-2.44123300
**INT2**			
P	1.49446000	0.12094500	-0.78550200
O	3.03409100	-0.56744300	1.47165400
O	-2.22410400	-0.42816200	3.10936000
C	2.10939400	-1.61624900	-1.20660400
H	1.60015300	-1.73078800	-2.17071000
C	1.65130700	-2.76963500	-0.30240900
H	2.16529900	-2.71946000	0.66274700
H	0.58127200	-2.70358600	-0.10157000
C	1.97319900	-4.11788800	-0.96931100
H	1.64318700	-4.93728900	-0.31804200

H	1.39885800	-4.20691600	-1.90258300
C	3.47219600	-4.24288000	-1.27484800
H	3.68510900	-5.19563000	-1.77611700
H	4.03078600	-4.25274000	-0.32657100
C	3.95788200	-3.06593400	-2.13211700
H	3.48282900	-3.11821200	-3.12235600
H	5.04056600	-3.13447800	-2.29964300
C	3.62102300	-1.71002600	-1.48705100
H	4.17173200	-1.61200500	-0.54406100
H	3.92909100	-0.89697400	-2.14649000
C	2.47360300	1.65068900	-0.24248700
H	2.37485000	1.69336600	0.84497100
C	3.97249700	1.56833300	-0.59450400
H	4.07434500	1.53754800	-1.68337000
H	4.41109600	0.65046100	-0.19762800
C	4.72276700	2.78763600	-0.03425400
H	4.66276600	2.77817400	1.06484000
H	5.78743400	2.71374900	-0.29113100
C	4.13248800	4.10088700	-0.56464700
H	4.29397200	4.15287200	-1.65107800
H	4.65119300	4.96231900	-0.12479600
C	2.62725900	4.17496000	-0.27780700
H	2.19756100	5.08720200	-0.71097300
H	2.46711700	4.23919500	0.80920700
C	1.87822600	2.94971400	-0.82621100
H	0.82064800	3.03572400	-0.58144100
H	1.95131000	2.91116600	-1.91710700
C	0.75102400	-0.22209300	0.99009500
C	1.74539100	-0.52082200	1.95827900
C	1.44180400	-0.76733200	3.28921200
H	2.21762300	-0.99324500	4.01088300
C	0.11042400	-0.73652200	3.70959500
H	-0.11921400	-0.93512600	4.74944800
C	-0.89051100	-0.46166000	2.78794300
C	-0.58792700	-0.19581000	1.42431800
C	4.10413600	-0.81919200	2.36475700
H	4.01492600	-1.80994000	2.83026600
H	4.16223200	-0.05281400	3.14910900
H	5.01223700	-0.78603000	1.75995500
C	-2.60162700	-0.66957100	4.45373400
H	-2.30652400	-1.67540000	4.78340900
H	-3.68992000	-0.58883000	4.47445400
H	-2.16975900	0.07595700	5.13582300
C	-1.77722300	0.07039900	0.54230400

C	-2.52234200	-1.01839500	0.03403700
C	-3.63261900	-0.76075100	-0.76912700
H	-4.18608300	-1.59916100	-1.18293800
C	-4.04683900	0.53922800	-1.06639100
C	-3.33676200	1.59740300	-0.50154900
H	-3.66524900	2.61090300	-0.71166400
C	-2.21383900	1.39319600	0.30713300
C	-2.17110400	-2.46888300	0.35047200
H	-1.23069300	-2.47518600	0.90978600
C	-3.24117000	-3.09653200	1.26474900
H	-3.36465900	-2.50429700	2.17582300
H	-2.95870000	-4.11928800	1.54397900
H	-4.21162500	-3.14323000	0.75606700
C	-1.96568300	-3.32107800	-0.91568600
H	-2.90296800	-3.44254800	-1.47114400
H	-1.61230600	-4.32346400	-0.64392300
H	-1.23929700	-2.86136900	-1.59033500
C	-5.22157700	0.79572500	-1.99653600
H	-5.37450200	1.88294300	-2.04153100
C	-4.90365900	0.30959700	-3.42323900
H	-3.98134600	0.76693500	-3.79675200
H	-5.72099400	0.55758700	-4.11144600
H	-4.76392400	-0.77764400	-3.44261600
C	-6.52398000	0.16486500	-1.47276000
H	-7.36445300	0.40554600	-2.13482900
H	-6.76246300	0.52851900	-0.46711300
H	-6.44309000	-0.92746000	-1.42264900
C	-1.51996900	2.58862300	0.95443700
H	-0.46693100	2.32305400	1.10152800
C	-2.11904100	2.86782300	2.34882500
H	-3.17602000	3.14594700	2.25566100
H	-1.59063800	3.69649700	2.83659900
H	-2.06355800	1.98783100	2.99275700
C	-1.56700500	3.87101400	0.10523400
H	-1.28195000	3.67934200	-0.93283200
H	-0.88377100	4.62073800	0.52094100
H	-2.56919100	4.31503800	0.10361500
C	-0.08338000	0.29198600	-1.78437100
F	2.07133900	0.55334700	-2.44910700
F	-0.44454100	1.58404700	-2.00065600
O	-0.64706400	-0.59579100	-2.34167700

INT3

P	-1.55480700	0.25282900	-0.41734600

O	-2.47852900	-2.76363900	-1.14163600
O	3.04274800	-2.57109000	-1.04725700
C	-2.73443500	-0.45911300	0.96043600
H	-2.81580000	0.39189700	1.64736200
C	-2.17731400	-1.61959900	1.80686200
H	-2.14738700	-2.53783500	1.21148200
H	-1.15769300	-1.40101000	2.12688000
C	-3.05480000	-1.86694600	3.04661000
H	-2.64643800	-2.70007300	3.63846100
H	-3.02497400	-0.97563700	3.69265400
C	-4.50926200	-2.16116900	2.64928500
H	-5.13752900	-2.29292500	3.54345700
H	-4.54060300	-3.11170300	2.09372300
C	-5.07173000	-1.04593800	1.75420000
H	-5.14839500	-0.11744700	2.34319200
H	-6.09347300	-1.29872400	1.43105000
C	-4.17544400	-0.79699300	0.52545200
H	-4.16166600	-1.69107500	-0.10162400
H	-4.60842400	0.00514400	-0.08061500
C	-2.57147200	1.95042700	-0.52684000
H	-3.59843900	1.57181900	-0.59568900
C	-2.56073100	2.93071600	0.66656900
H	-1.57816900	3.41106000	0.75445000
H	-2.74100700	2.41328700	1.61595700
C	-3.62405600	4.03099400	0.48638200
H	-4.62458500	3.56941900	0.47369900
H	-3.60411900	4.71898500	1.34525400
C	-3.41054800	4.80796600	-0.82160000
H	-2.45613500	5.35448600	-0.75786500
H	-4.19988200	5.56340100	-0.95624100
C	-3.35924600	3.85334300	-2.02414500
H	-3.14335400	4.41589800	-2.94560200
H	-4.35240000	3.39521300	-2.16068000
C	-2.31456900	2.73984400	-1.82373100
H	-2.32303400	2.05962800	-2.67959500
H	-1.31085300	3.18393000	-1.78554200
C	-0.45871200	-1.44268600	-0.70669600
C	-1.09564700	-2.62528700	-1.16312400
C	-0.39104900	-3.75444500	-1.57796500
H	-0.94758400	-4.63435000	-1.89035600
C	0.99865600	-3.77324900	-1.58124400
H	1.53031800	-4.65649800	-1.91758500
C	1.66221800	-2.64501400	-1.10908700
C	0.96214000	-1.50375800	-0.64690200

C	-3.10872200	-2.88942300	-2.41091900
H	-2.88943900	-3.86325200	-2.87649800
H	-2.80389400	-2.07716300	-3.07795900
H	-4.18550200	-2.81860400	-2.22777600
C	3.80090100	-3.66647300	-1.51285200
H	3.60501700	-4.57835400	-0.92767700
H	4.84781600	-3.37797200	-1.38742800
H	3.60667700	-3.87460000	-2.57615500
C	1.86839100	-0.45066100	-0.05932400
C	2.21906400	-0.53852300	1.30847300
C	3.10499500	0.40359500	1.84445500
H	3.34650800	0.36424300	2.90433300
C	3.68417400	1.40237400	1.06278900
C	3.38455400	1.42207400	-0.30163900
H	3.84763200	2.17626400	-0.93351800
C	2.49572500	0.51394500	-0.88111100
C	1.72094000	-1.67905200	2.19692500
H	0.82836900	-2.10231200	1.72772600
C	2.77982500	-2.80138700	2.26624000
H	3.04948600	-3.15703900	1.26910300
H	2.40175300	-3.64940300	2.85418400
H	3.69406400	-2.43106900	2.75031400
C	1.33313800	-1.24974600	3.62379100
H	2.21576000	-0.97202600	4.21552100
H	0.84712900	-2.08802500	4.14064500
H	0.64403200	-0.40343800	3.61185100
C	4.59931500	2.44629300	1.68488500
H	4.68960600	2.20757300	2.75482500
C	6.01680200	2.41160700	1.08200600
H	6.46376400	1.41549500	1.18542000
H	6.67191100	3.13997000	1.57991000
H	5.99476200	2.65682600	0.01286100
C	3.99045000	3.85757600	1.57385400
H	3.90390600	4.16210900	0.52338600
H	4.61737300	4.59724400	2.09150700
H	2.98458100	3.88278600	2.00687500
C	2.28149600	0.55947100	-2.39098500
H	1.42724800	-0.07901200	-2.62466800
C	3.51620100	0.00035200	-3.12909200
H	4.39994700	0.62606500	-2.94153300
H	3.33932000	-0.01718300	-4.21386500
H	3.74479600	-1.01560800	-2.79398000
C	1.94618100	1.97166800	-2.90514300
H	1.11061600	2.39478400	-2.34425000

H	1.66300500	1.92541900	-3.96539900
H	2.80846400	2.64802900	-2.82487600
C	-0.41523800	1.06262000	0.98741500
F	0.37851600	2.11248700	0.51789600
F	-0.44619700	0.86400300	-1.59115500
O	-0.47518700	0.95185800	2.17875100
F	-2.53525300	-0.24654800	-1.74265100

INT4

P	1.66601900	-0.31416400	0.21529900
O	3.25483900	2.09245900	0.60989800
O	-2.00613700	3.37986700	-0.46523100
C	0.89773700	-1.95689400	0.70814000
H	1.82083300	-2.52358800	0.91359700
C	0.10754300	-2.68728900	-0.39148100
H	-0.78350400	-2.10086700	-0.63640600
H	0.69456500	-2.76095300	-1.30975900
C	-0.31379500	-4.09391800	0.06581800
H	-0.90911000	-4.56774900	-0.72554400
H	0.58276700	-4.71851700	0.20044100
C	-1.10159800	-4.05957700	1.38131100
H	-1.36748200	-5.07634600	1.69816500
H	-2.04301600	-3.51434400	1.22409500
C	-0.29583900	-3.34231500	2.47008200
H	0.61032100	-3.92300900	2.70080400
H	-0.87495600	-3.28192400	3.40119500
C	0.09897000	-1.92830100	2.02147200
H	-0.81115000	-1.35462000	1.84650100
H	0.66734000	-1.42346200	2.80392600
C	3.40152900	-0.59342000	-0.43805200
H	3.61205800	0.31867300	-1.00865400
C	4.47497700	-0.74809200	0.65414100
H	4.26078300	-1.64931500	1.24614300
H	4.43370500	0.09313100	1.34596600
C	5.87680400	-0.87255600	0.03713200
H	6.12455600	0.06451600	-0.48549300
H	6.62268700	-0.99352700	0.83343800
C	5.95856700	-2.03852800	-0.95605000
H	5.81199900	-2.98401200	-0.41277400
H	6.95511400	-2.08855900	-1.41344600
C	4.87754800	-1.90596200	-2.03580300
H	4.90726600	-2.76492300	-2.71850200
H	5.08336800	-1.01344600	-2.64548600
C	3.47093100	-1.78074000	-1.42718000

H	2.73295800	-1.65642300	-2.22133800
H	3.22842000	-2.71587800	-0.90240000
C	1.00757200	1.43589200	0.22345800
C	1.96309700	2.47333300	0.36739800
C	1.59121800	3.80718200	0.22494900
H	2.32287900	4.59961700	0.32424000
C	0.26876400	4.14121800	-0.06173400
H	0.00431000	5.18481800	-0.18190700
C	-0.68405900	3.13685300	-0.18579700
C	-0.33644600	1.77420700	-0.01770200
C	4.26551300	3.07977700	0.69377800
H	4.09113900	3.76749300	1.53233600
H	4.34796000	3.65791400	-0.23704700
H	5.19572900	2.53399500	0.86397900
C	-2.42634300	4.72379400	-0.62448900
H	-2.26685700	5.30858900	0.29218700
H	-3.49535200	4.67576400	-0.83952800
H	-1.90993400	5.21381900	-1.46134600
C	-1.47024500	0.78619800	-0.06958700
C	-2.17100200	0.46778800	1.12100000
C	-3.25453300	-0.41639600	1.04435100
H	-3.78568900	-0.68574000	1.95264900
C	-3.67962800	-0.97154700	-0.16348600
C	-3.00968200	-0.59323700	-1.32825400
H	-3.35019600	-0.98261400	-2.28332500
C	-1.91967700	0.28149600	-1.31099400
C	-1.80978600	1.10560100	2.46764500
H	-0.73018300	1.29492100	2.46797800
C	-2.53121900	2.46037800	2.63927100
H	-2.30961700	3.14836600	1.82456000
H	-2.23796300	2.93038700	3.58593600
H	-3.61732000	2.30658800	2.65828300
C	-2.12676100	0.23677800	3.69969100
H	-3.20724300	0.15586300	3.86779500
H	-1.69782300	0.70412800	4.59325600
H	-1.72154600	-0.77475500	3.62279900
C	-4.86067900	-1.92938200	-0.21248300
H	-5.15670500	-2.14027800	0.82453700
C	-6.06738500	-1.28459900	-0.92028400
H	-6.35476400	-0.34682900	-0.43233400
H	-6.93229900	-1.95895300	-0.91028800
H	-5.82990400	-1.05739300	-1.96652400
C	-4.49172500	-3.27032800	-0.87282200
H	-4.21085000	-3.13079100	-1.92321500

H	-5.34298300	-3.96113400	-0.84532800
H	-3.64713200	-3.74622600	-0.36358700
C	-1.31122800	0.71994100	-2.64036400
H	-0.37341000	1.23731400	-2.42684800
C	-2.25673100	1.71039800	-3.34991000
H	-3.20458700	1.22302200	-3.61097600
H	-1.80152600	2.07803900	-4.27809500
H	-2.48216300	2.56448400	-2.70638000
C	-0.97725100	-0.45860900	-3.57152900
H	-0.31881600	-1.17288500	-3.07490700
H	-0.46985100	-0.08904400	-4.47087700
H	-1.88014600	-0.98593400	-3.90280100
F	2.16131800	-0.09065000	1.81993300
F	1.06562200	-0.40250900	-1.38285900

INT5

P	-1.48132200	0.46936500	-0.38883600
O	-2.84179400	-2.29663300	-1.36098700
O	2.51345300	-3.09760700	-0.25058000
C	-2.92019700	-0.02374300	0.81127500
H	-2.89463200	0.82764400	1.50920600
C	-2.69994800	-1.26595300	1.69267400
H	-2.75202400	-2.17720400	1.08735300
H	-1.70260300	-1.22933200	2.14031700
C	-3.76555500	-1.35708100	2.79848500
H	-3.60384800	-2.25469700	3.41375500
H	-3.66537200	-0.49214400	3.47275800
C	-5.18280000	-1.36959100	2.20604800
H	-5.93786900	-1.40308800	3.00566000
H	-5.30975400	-2.28704600	1.61109100
C	-5.41082100	-0.15311000	1.29541600
H	-5.38815300	0.76130300	1.90955200
H	-6.41363400	-0.20185400	0.84449200
C	-4.33267700	-0.06024000	0.20192800
H	-4.40939300	-0.92726000	-0.45912200
H	-4.51188400	0.82340500	-0.42061800
C	-2.00091700	2.33925300	-0.34747100
H	-3.07796500	2.33984100	-0.12156400
C	-1.30945200	3.15815000	0.75834300
H	-0.22764400	3.16240500	0.56903500
H	-1.44244600	2.67865800	1.73327200
C	-1.83509100	4.60296200	0.82209800
H	-2.89499900	4.58546400	1.12308300
H	-1.30007100	5.17074800	1.59815500

C	-1.71905600	5.31681900	-0.53184400
H	-0.65382300	5.42856000	-0.78630300
H	-2.13855700	6.33240600	-0.47267300
C	-2.40836500	4.50239300	-1.63578900
H	-2.28864000	5.00353500	-2.60828000
H	-3.49078200	4.46758100	-1.43293300
C	-1.84951900	3.07151400	-1.69629700
H	-2.34651300	2.50004600	-2.48526800
H	-0.78712300	3.11504800	-1.96210700
C	-0.70535300	-1.33339700	-0.64098900
C	-1.47908600	-2.41956900	-1.10727400
C	-0.96387700	-3.70357400	-1.26930800
H	-1.63391100	-4.49271200	-1.60092700
C	0.36924000	-3.97754500	-0.99244400
H	0.75544300	-4.98299500	-1.11516700
C	1.17145000	-2.92924700	-0.54936700
C	0.66303300	-1.61964700	-0.37665300
C	-3.17829000	-2.23771900	-2.73948200
H	-2.95555900	-3.18942500	-3.24903200
H	-2.65252100	-1.41838300	-3.23669000
H	-4.25601000	-2.05216500	-2.78645300
C	3.07796000	-4.37885600	-0.41198500
H	2.59905100	-5.12633000	0.23859200
H	4.12913000	-4.28066300	-0.12860100
H	3.01805200	-4.72787200	-1.45411700
C	1.70783800	-0.62249700	0.06482900
C	2.00631800	-0.46172500	1.43100700
C	3.04053300	0.39989000	1.80964400
H	3.26274300	0.53053600	2.86740900
C	3.79921600	1.09950800	0.87226700
C	3.50855600	0.90176600	-0.47964000
H	4.10002500	1.42196300	-1.22954100
C	2.48264000	0.05244200	-0.90139100
C	1.25499300	-1.23891800	2.50523300
H	0.40145600	-1.72120300	2.02271500
C	2.14810200	-2.35131800	3.08686700
H	2.51629800	-3.00351700	2.28892200
H	1.59228700	-2.96021700	3.81286100
H	3.01860000	-1.92403200	3.60233700
C	0.69519600	-0.33854800	3.61896500
H	1.49588000	0.13631300	4.20061000
H	0.09020400	-0.93223500	4.31721700
H	0.06625800	0.44139200	3.18600800
C	4.90300100	2.05024800	1.31049000

H	4.94906000	2.01866500	2.40876500
C	6.28126500	1.61661100	0.77830800
H	6.51773800	0.59408800	1.09369000
H	7.07389100	2.28437900	1.14192400
H	6.30201300	1.63831800	-0.31782400
C	4.59023400	3.50157200	0.90122800
H	4.53929400	3.59533600	-0.19011000
H	5.36374100	4.19193300	1.26437300
H	3.62263500	3.81943000	1.30402400
C	2.27311600	-0.17739200	-2.39491000
H	1.30381900	-0.66344300	-2.51976600
C	3.35940000	-1.12572200	-2.93892900
H	4.35353700	-0.66595000	-2.85630300
H	3.18213300	-1.35945200	-3.99761400
H	3.37597400	-2.06045600	-2.37004900
C	2.22195800	1.12700300	-3.20644800
H	1.45084600	1.78552300	-2.80224200
H	1.98024800	0.90840900	-4.25505100
H	3.18386100	1.65619300	-3.19834000
F	-0.14811500	0.96289300	-1.35402800
F	-2.39402000	0.27366900	-1.80367900
F	-0.50455400	0.63672300	0.99607400

INT6

P	-1.28365300	-0.06707300	-0.73626300
O	-2.94654400	0.45492600	1.62287100
O	2.42574700	0.47901800	2.89057700
C	-2.61176500	1.14026500	-1.39798000
H	-2.30402800	1.22090100	-2.45110200
C	-2.43791100	2.54666800	-0.76303900
H	-2.73741800	2.49222200	0.29040100
H	-1.39199700	2.85017500	-0.79154600
C	-3.30280800	3.59615700	-1.47798900
H	-3.16818600	4.57248900	-0.99427700
H	-2.95110600	3.70841800	-2.51421700
C	-4.78346100	3.19941800	-1.48544300
H	-5.38079500	3.93751500	-2.03588100
H	-5.16064900	3.19321000	-0.45156800
C	-4.96143800	1.80302200	-2.09190600
H	-4.67772500	1.82981300	-3.15447800
H	-6.01578900	1.49920000	-2.05727500
C	-4.10248900	0.75101600	-1.36549900
H	-4.43592200	0.67991800	-0.32440500
H	-4.28032400	-0.22215100	-1.82722300

C	-2.39506400	-1.59864800	-0.41177000
H	-3.32507900	-1.20431100	0.00289600
C	-2.72868100	-2.35629200	-1.71966700
H	-1.81719300	-2.83045000	-2.09868800
H	-3.06498400	-1.67515700	-2.51069600
C	-3.79037800	-3.43737400	-1.46582900
H	-4.72187700	-2.96200500	-1.12296800
H	-4.02939600	-3.95976100	-2.40142200
C	-3.30577200	-4.43891100	-0.40697100
H	-2.44294000	-4.98976900	-0.80955800
H	-4.08428200	-5.18314300	-0.19468300
C	-2.88061900	-3.72343300	0.88329800
H	-2.47163100	-4.44644600	1.60150600
H	-3.76707700	-3.27614200	1.35872600
C	-1.84838100	-2.61509200	0.60946600
H	-1.58463900	-2.12864700	1.55420300
H	-0.93043200	-3.06620900	0.21665200
C	-0.67772200	0.28006300	0.98089300
C	-1.63062600	0.45895900	2.01113500
C	-1.23438700	0.64195100	3.33069300
H	-1.96426300	0.78752700	4.11759900
C	0.12269900	0.64122800	3.65582500
H	0.41349300	0.77755100	4.69046600
C	1.07578300	0.47972700	2.65591200
C	0.69385400	0.31180800	1.30101500
C	-3.95828200	0.48576500	2.61569700
H	-3.94699100	1.43125500	3.17389300
H	-3.85789000	-0.35297900	3.31704000
H	-4.90446900	0.39827200	2.07836300
C	2.88141700	0.68902000	4.21635300
H	2.56284600	1.66712000	4.60272100
H	3.97102100	0.66040300	4.16171700
H	2.53210200	-0.10056500	4.89612200
C	1.82212700	0.21370900	0.31035500
C	2.35974700	1.39901700	-0.23171900
C	3.46920700	1.31020800	-1.07530200
H	3.88971100	2.22119300	-1.49464900
C	4.06145400	0.08807800	-1.39703400
C	3.51544700	-1.06922100	-0.83975800
H	3.96913200	-2.03002300	-1.06732800
C	2.40844800	-1.03077500	0.01302100
C	1.78970200	2.76990900	0.11624700
H	0.82025400	2.61712400	0.59883000
C	2.70450000	3.48731300	1.12775700

H	2.85762600	2.87045200	2.01886000
H	2.27090500	4.44765200	1.43431400
H	3.68885000	3.68669200	0.68629100
C	1.53947600	3.64701700	-1.12222200
H	2.47535300	3.93104600	-1.61764100
H	1.03217800	4.57518300	-0.83021100
H	0.91394700	3.11763300	-1.84491300
C	5.26872300	0.02377800	-2.32045500
H	5.49833000	1.05197300	-2.63311400
C	6.50890200	-0.52704700	-1.59243300
H	6.74192600	0.07077500	-0.70432100
H	7.38474700	-0.52011300	-2.25289100
H	6.34521500	-1.56058800	-1.26460700
C	4.96803000	-0.79157000	-3.59105700
H	4.74498100	-1.83626500	-3.34410500
H	5.82843800	-0.78432200	-4.27151000
H	4.10200600	-0.38377100	-4.12337500
C	1.91067100	-2.32548700	0.64405000
H	0.94131300	-2.12279100	1.10897900
C	2.86787800	-2.77866000	1.76355300
H	3.85808600	-3.01345600	1.35406100
H	2.48564100	-3.67780900	2.26319200
H	2.99247600	-1.98703600	2.50876200
C	1.69663500	-3.44521600	-0.38842800
H	1.04616500	-3.10381300	-1.19826700
H	1.23613400	-4.31952600	0.08807000
H	2.64415200	-3.77695800	-0.82873300
F	-0.19760800	1.08039200	-1.37721100
F	-0.30219700	-1.10319100	-1.56904100

INT7

C	-3.05251000	0.21455600	-1.21255700
C	-1.68483200	-0.08128700	-1.19884600
C	-0.95659400	-0.23974300	0.00013900
C	-1.68498100	-0.08123400	1.19900500
C	-3.05266300	0.21461300	1.21252700
C	-3.71397100	0.35917500	-0.00005800
H	-3.60588900	0.33081400	-2.14008900
H	-1.18197400	-0.19698900	-2.15664700
H	-1.18223000	-0.19686700	2.15687100
H	-3.60614400	0.33091700	2.13999100
F	-5.03931000	0.65016500	-0.00015000
Ag	1.05221000	-0.67762000	0.00003000
F	3.12211500	-0.76200000	-0.00016000

K	3.27633800	1.59317300	-0.00000100

INT8

P	1.53223100	0.15486100	-0.27156100
O	3.19621500	-0.67073900	2.15483800
O	-2.25940000	-1.27018800	2.82237400
C	2.64814900	-0.97868400	-1.25077100
H	2.64687600	-0.43655500	-2.20778600
C	1.99057800	-2.34516700	-1.50755000
H	1.90189400	-2.89624700	-0.55929000
H	0.98276200	-2.19702000	-1.89961000
C	2.81127400	-3.17485800	-2.50731600
H	2.33965600	-4.15595200	-2.64701500
H	2.78911300	-2.67113000	-3.48414300
C	4.26764100	-3.33543800	-2.05173000
H	4.84394600	-3.89390900	-2.80001300
H	4.29439900	-3.92779400	-1.12443400
C	4.91175400	-1.96638400	-1.79094500
H	4.97129200	-1.40595100	-2.73534400
H	5.94172300	-2.08885200	-1.43134800
C	4.10082100	-1.15205800	-0.76827500
H	4.10374000	-1.67141900	0.19598300
H	4.58202800	-0.18168700	-0.60643000
C	2.60236600	1.63286600	0.11478100
H	3.47654400	1.23069800	0.64030300
C	3.06391900	2.33803200	-1.17941300
H	2.18567400	2.66159300	-1.74819400
H	3.60050000	1.63988200	-1.83044900
C	3.97229600	3.53360800	-0.84930000
H	4.89918100	3.16775600	-0.38091200
H	4.26655600	4.03634800	-1.77880800
C	3.28706800	4.52435800	0.10181800
H	2.41492400	4.96450300	-0.40343900
H	3.96374700	5.35289100	0.34650400
C	2.82079100	3.81740600	1.38178200
H	2.29459800	4.51943800	2.04130000
H	3.69787400	3.45538900	1.93927900
C	1.90367100	2.62872800	1.05518400
H	1.59487800	2.12886600	1.98121100
H	0.99816100	3.00093900	0.56467400
C	0.98960600	-0.55571000	1.33328100
C	1.86580700	-0.87661100	2.39409100
C	1.35825900	-1.35236200	3.60000900
H	2.01545100	-1.60519500	4.42351800

C	-0.02228400	-1.50247900	3.76609800
H	-0.39526500	-1.87250200	4.71373300
C	-0.89780200	-1.17122600	2.73475700
C	-0.39796800	-0.68948400	1.50127300
C	4.13125600	-0.96026400	3.18217500
H	4.09897900	-2.01977400	3.46889300
H	3.95706100	-0.33799700	4.07003500
H	5.11209100	-0.72900200	2.76336600
C	-2.83203700	-1.73590200	4.03337600
H	-2.50843000	-2.75992200	4.26473900
H	-3.91117200	-1.72566900	3.87306200
H	-2.58340100	-1.07654600	4.87622800
C	-1.43371300	-0.32906800	0.45018900
C	-2.06500100	-1.40511000	-0.27565500
C	-3.30803700	-1.19601100	-0.85641200
H	-3.76002600	-1.99112200	-1.44146700
C	-3.98639300	0.02209800	-0.75082800
C	-3.41105400	1.04321600	0.00591700
H	-3.93332300	1.98983400	0.09600100
C	-2.16555100	0.90413400	0.61005400
C	-1.41230100	-2.77445700	-0.38916500
H	-0.37233200	-2.67609400	-0.06765200
C	-2.09105900	-3.78980000	0.55199200
H	-2.04661800	-3.45964200	1.59328700
H	-1.60044100	-4.76751700	0.47356600
H	-3.14674900	-3.91685900	0.28464600
C	-1.41393400	-3.29310100	-1.83993500
H	-2.41623700	-3.60808600	-2.15126400
H	-0.75352200	-4.16343700	-1.93093600
H	-1.07934600	-2.51941200	-2.53830100
C	-5.30168900	0.23759500	-1.47305400
H	-5.54840800	-0.69814800	-1.99270300
C	-6.45004300	0.54757800	-0.49491900
H	-6.57524000	-0.25327500	0.24314000
H	-7.39451100	0.66376900	-1.03934300
H	-6.26431100	1.48102600	0.04967500
C	-5.15163600	1.34200900	-2.53842500
H	-4.99080400	2.31743500	-2.06394500
H	-6.06094600	1.40811200	-3.14814900
H	-4.28580100	1.14471200	-3.17507900
C	-1.63999300	2.04049100	1.47467400
H	-0.60958000	1.80541100	1.74818900
C	-2.44883300	2.15177000	2.78206300
H	-3.49674700	2.39468300	2.57059700

H	-2.03828300	2.94529900	3.41805400
H	-2.42859600	1.21082300	3.33911200
C	-1.63432900	3.38105800	0.71631800
H	-1.15095600	3.27953100	-0.26090600
H	-1.10502500	4.14415500	1.29929700
H	-2.65215900	3.74728900	0.54189300
Pd	-0.37267100	0.52113800	-1.41218600
F	-1.96683000	0.85124900	-2.53014500
F	0.83681600	1.02473400	-2.88695600

INT9

P	1.59454800	0.03868600	0.24323500
O	3.76994100	-1.39492100	1.85130600
O	-0.92102600	-4.28428200	1.42107300
C	2.87034100	0.12664100	-1.11801400
H	2.72583300	1.15709500	-1.47252700
C	2.51073300	-0.76021500	-2.32448200
H	2.65539200	-1.82001400	-2.06856400
H	1.45967700	-0.62316200	-2.59202500
C	3.38798000	-0.37971200	-3.52684100
H	3.13777300	-1.01840600	-4.38391200
H	3.13907100	0.65336300	-3.79852400
C	4.88098400	-0.49821900	-3.18916200
H	5.49252200	-0.17627100	-4.04142200
H	5.13027200	-1.55445600	-3.00167200
C	5.23481900	0.32666300	-1.94222500
H	5.09097000	1.39385200	-2.16114000
H	6.29280400	0.19217700	-1.68041300
C	4.35194100	-0.06274200	-0.74229300
H	4.53448300	-1.11088700	-0.47665000
H	4.62568600	0.53360100	0.13467300
C	2.21842100	1.20238600	1.56589000
H	3.12545000	0.72235900	1.94592200
C	2.61627700	2.58561200	1.01182900
H	1.75851200	3.05926600	0.52259500
H	3.38312200	2.48836900	0.23766700
C	3.13705800	3.49497800	2.13819400
H	4.07973200	3.08021000	2.52604500
H	3.37447400	4.48379100	1.72692700
C	2.13330500	3.61833100	3.29299700
H	1.22861100	4.13245300	2.93940100
H	2.55183000	4.24052900	4.09394600
C	1.75039500	2.23585500	3.83799000
H	0.99857600	2.32986300	4.63180300

H	2.63694400	1.77125300	4.29519100
C	1.21651000	1.31556500	2.72920600
H	1.02034400	0.32143700	3.14757000
H	0.26100500	1.69958100	2.35509500
C	1.55882200	-1.63437900	1.03561500
C	2.64901700	-2.17050000	1.76610700
C	2.54592900	-3.42392800	2.36400100
H	3.37450600	-3.84240800	2.92232800
C	1.36344800	-4.15912300	2.25916200
H	1.30558400	-5.12878500	2.73902600
C	0.27979300	-3.64254500	1.55569500
C	0.37075100	-2.37616500	0.92879100
C	4.93525000	-1.92204900	2.46566400
H	5.26823800	-2.84237000	1.96840500
H	4.77281700	-2.12175100	3.53306600
H	5.70075300	-1.15247000	2.35349500
C	-1.08184700	-5.56190000	2.01650400
H	-0.37186100	-6.29081000	1.60267900
H	-2.09972100	-5.87273600	1.77687800
H	-0.96280600	-5.51561700	3.10752400
C	-0.87380600	-1.92575600	0.19614300
C	-1.17406600	-2.52920100	-1.07468000
C	-2.48064700	-2.49075500	-1.54586100
H	-2.70230400	-2.90625300	-2.52405600
C	-3.51558900	-1.88321400	-0.83001600
C	-3.23279700	-1.33542500	0.42522300
H	-4.03192200	-0.86493500	0.98840300
C	-1.95220400	-1.35353000	0.96617900
C	-0.09704000	-3.21044100	-1.90565400
H	0.87220200	-2.92150400	-1.49043100
C	-0.20416100	-4.74566800	-1.81481800
H	-0.10552800	-5.09370200	-0.78378500
H	0.58000900	-5.21649400	-2.41963800
H	-1.17527300	-5.08601900	-2.19351700
C	-0.13490700	-2.75543400	-3.37775700
H	-0.97764900	-3.20790200	-3.91311000
H	0.78394800	-3.06269700	-3.88990200
H	-0.23739700	-1.66961600	-3.45852500
C	-4.90603100	-1.77007100	-1.42417300
H	-4.89887900	-2.30172000	-2.38506400
C	-5.97509800	-2.42669900	-0.53168100
H	-5.74624800	-3.48218400	-0.34507500
H	-6.95927400	-2.36800100	-1.01072700
H	-6.04896900	-1.92154800	0.43860000

C	-5.23940500	-0.29126600	-1.70927400
H	-5.29965500	0.27926500	-0.77431300
H	-6.20555000	-0.20898900	-2.22141100
H	-4.45625700	0.16319900	-2.32120100
C	-1.73488700	-0.84395800	2.38406000
H	-0.65831000	-0.76077400	2.54561100
C	-2.28102600	-1.85863500	3.40878100
H	-3.36574600	-1.97202000	3.29572300
H	-2.07996700	-1.51332800	4.42996500
H	-1.82544000	-2.84354200	3.27510800
C	-2.34832400	0.54783500	2.61295600
H	-2.04093000	1.25426500	1.83892300
H	-2.03234100	0.94024300	3.58660200
H	-3.44342900	0.51185500	2.61484800
Pd	-0.45503700	0.27025900	-0.72485900
F	-2.03606700	0.30724800	-1.93490900
C	-1.33669400	2.92440900	-0.33806500
C	-1.03392200	3.63724500	0.83745000
C	-2.03728500	4.16138300	1.65831200
C	-3.36296300	3.95466200	1.29834000
C	-3.70866900	3.25904400	0.14485700
C	-2.69450400	2.74333700	-0.66508500
H	-0.00494500	3.80423500	1.13185400
H	-1.80405000	4.71476200	2.56292600
H	-4.75681700	3.11601700	-0.10025800
H	-2.95498500	2.14900700	-1.53143000
F	1.83751500	2.47576100	-2.84486100
F	-4.34292900	4.43578300	2.09669000
Ag	0.19883400	2.63823600	-1.69372400

INT10

P	-0.76294000	1.25677800	0.21081300
O	-0.54805000	4.21870600	-0.17291800
O	4.36130500	1.68646900	-0.04719700
C	-1.86968200	1.53378900	-1.29183300
H	-2.25953900	0.52813600	-1.47884000
C	-1.05781400	1.88685400	-2.55319000
H	-0.65112800	2.90150900	-2.45475800
H	-0.21735100	1.19986300	-2.65964000
C	-1.94870500	1.77826000	-3.80072200
H	-1.36750700	2.07072500	-4.68480800
H	-2.21778400	0.72208400	-3.93575000
C	-3.19752900	2.66295200	-3.67317600
H	-3.84959100	2.53029900	-4.54529200

H	-2.89468600	3.72183100	-3.66246700
C	-3.97537500	2.35799100	-2.38458700
H	-4.37802700	1.33607900	-2.44042100
H	-4.83534900	3.03280900	-2.28393600
C	-3.07908300	2.47338500	-1.13757100
H	-2.72905400	3.50499900	-1.02118000
H	-3.66441200	2.23037400	-0.24318900
C	-1.57469600	2.05638400	1.71438900
H	-1.96212800	3.01175800	1.34456200
C	-2.74274200	1.23695800	2.29604300
H	-2.37406700	0.25399300	2.61443300
H	-3.50430400	1.04686800	1.53710000
C	-3.39624900	1.96455400	3.48263900
H	-3.85633100	2.89707900	3.12269300
H	-4.20938800	1.34658200	3.88299400
C	-2.37902200	2.29505600	4.58117500
H	-1.99811300	1.35864100	5.01475200
H	-2.85991300	2.84933500	5.39677000
C	-1.20450700	3.09514400	4.00380600
H	-0.45193500	3.29012100	4.77839600
H	-1.56418100	4.07583100	3.65813600
C	-0.54940800	2.35508700	2.82757400
H	0.28595200	2.94747500	2.44192000
H	-0.13524000	1.40814000	3.19190300
C	0.76413700	2.26321900	-0.03257600
C	0.70604800	3.67097200	-0.15985600
C	1.87587900	4.41646900	-0.25692500
H	1.84631400	5.49483400	-0.35631600
C	3.11730700	3.77385100	-0.21815700
H	4.01700800	4.37353100	-0.28635400
C	3.18880600	2.38917700	-0.09901000
C	2.00495200	1.61305900	-0.02497000
C	-0.68201500	5.62333300	-0.32888700
H	-0.25038600	5.96373100	-1.27898800
H	-0.21104900	6.16546900	0.50161600
H	-1.75536900	5.82057100	-0.32885200
C	5.58467400	2.39708800	-0.15784600
H	5.65597700	2.92586100	-1.11791800
H	6.37048200	1.64234200	-0.10169800
H	5.70968500	3.11470400	0.66440100
C	2.19070600	0.11470700	0.01488800
C	2.47187900	-0.55839700	-1.22098900
C	3.02366200	-1.83552900	-1.17614100
H	3.20699000	-2.36246700	-2.10795700

C	3.35627600	-2.46584500	0.02537600
C	3.14198400	-1.77701100	1.22163000
H	3.40586700	-2.25445000	2.16022500
C	2.56794900	-0.50941500	1.24930600
C	2.30339100	0.12894000	-2.57167700
H	1.77138000	1.06994800	-2.40723900
C	3.69089100	0.49053700	-3.14570800
H	4.27386700	1.08340200	-2.43578700
H	3.57865400	1.06198900	-4.07460800
H	4.26208600	-0.41681900	-3.37451200
C	1.50254700	-0.69606400	-3.59436300
H	1.98930300	-1.65497200	-3.80535300
H	1.44566200	-0.14620700	-4.54174200
H	0.47578400	-0.90802400	-3.28402800
C	3.93012900	-3.87056900	0.02882900
H	4.00977600	-4.19542200	-1.01725000
C	5.34385000	-3.90577700	0.63919100
H	6.02639300	-3.23581700	0.10389600
H	5.75458400	-4.92136000	0.59626200
H	5.32732600	-3.59796600	1.69157700
C	2.98271000	-4.84815000	0.75070700
H	2.91376700	-4.60514600	1.81803300
H	3.35533600	-5.87578100	0.66166800
H	1.97471300	-4.78256300	0.33414700
C	2.42374400	0.21246700	2.58240900
H	1.88488700	1.14441600	2.39641900
C	3.80513200	0.58722700	3.15289100
H	4.38951300	-0.31128000	3.38300700
H	3.69415600	1.16487800	4.07858300
H	4.37340400	1.18214600	2.43220500
C	1.60913200	-0.60664300	3.60044200
H	0.63281400	-0.88944700	3.19083900
H	1.45041400	-0.02552200	4.51716900
H	2.12881200	-1.53023300	3.87918100
Pd	-0.06374500	-0.91584700	0.31346200
F	0.60938100	-2.78782200	0.23249900
C	-1.93706900	-1.80053700	0.59617400
C	-3.22880600	-1.26494100	0.36117600
C	-4.39375100	-1.90014700	0.80786000
C	-4.27337800	-3.08085800	1.52371500
C	-3.03098800	-3.65359800	1.78550500
C	-1.88026400	-3.02500600	1.31652200
H	-3.35584100	-0.32319000	-0.15614000
H	-5.37620300	-1.48368600	0.61003400

H	-2.97976800	-4.58520100	2.34055400
H	-0.91444200	-3.48433400	1.48205500
F	-1.70880200	-1.56326500	-3.68468600
F	-5.38668600	-3.69359900	1.97471600
Ag	-1.66490900	-2.04081400	-1.71634400

INT11

P	1.11673100	0.74692200	-0.08100000
F	5.10509300	-4.50581500	0.46197700
O	1.53429000	3.61321900	-1.08273300
O	-3.79278100	2.26467700	-0.51836700
C	1.63851500	-2.23041300	0.47439800
C	2.54232700	-2.16198600	1.54205200
H	2.34862600	-1.50996100	2.38902600
C	3.71336700	-2.92998100	1.54751400
H	4.42303300	-2.88094100	2.36791000
C	3.96419800	-3.77455400	0.47454200
C	3.07008800	-3.89713200	-0.58362700
H	3.28870500	-4.58872500	-1.39165800
C	1.90246900	-3.12881600	-0.57074000
H	1.19313700	-3.23815300	-1.38568800
C	2.17631100	1.30911900	1.36029600
H	2.88724800	0.47264500	1.40545000
C	1.40332300	1.30656500	2.69003000
H	0.68751000	2.14000900	2.69662600
H	0.82208100	0.38611200	2.78663700
C	2.36376400	1.45361100	3.88093600
H	1.79284800	1.47253000	4.81783600
H	3.01281300	0.56643600	3.92555700
C	3.23292500	2.71277300	3.75342800
H	3.94740100	2.77080200	4.58404500
H	2.58924700	3.60218100	3.82750100
C	3.97104900	2.74143200	2.40700400
H	4.69629100	1.91501400	2.37005400
H	4.54786500	3.67007000	2.30752300
C	2.99111800	2.60866600	1.22809900
H	2.30624100	3.46426300	1.22978100
H	3.53132500	2.64392300	0.27754300
C	2.30591300	0.70280800	-1.53690400
H	2.49853200	1.74683700	-1.79933600
C	3.65855700	0.03251100	-1.20155300
H	3.49594000	-0.99124200	-0.85445600
H	4.16775700	0.56637900	-0.39251300
C	4.57316100	0.00358700	-2.43756900

H	4.82444900	1.03420300	-2.73193600
H	5.51735200	-0.49128500	-2.17819300
C	3.89959200	-0.70879200	-3.61726300
H	3.71459000	-1.75758400	-3.34335900
H	4.56364300	-0.71807100	-4.49084500
C	2.56586500	-0.03352700	-3.96181100
H	2.06485300	-0.56114300	-4.78360600
H	2.75755700	0.99147100	-4.31377500
C	1.63695600	0.01166400	-2.73957000
H	0.70726800	0.52780500	-3.00067300
H	1.36438800	-1.01046200	-2.45479000
C	-0.14515800	2.04776300	-0.48309500
C	0.20172100	3.35214800	-0.92039100
C	-0.78505300	4.29776300	-1.18396900
H	-0.52472400	5.29666900	-1.51253600
C	-2.13263800	3.96462700	-1.04120200
H	-2.88308300	4.71608200	-1.25550600
C	-2.49536600	2.68037800	-0.64787300
C	-1.50684300	1.70468500	-0.36405700
C	1.95369900	4.93849400	-1.36039000
H	1.60598800	5.63930500	-0.58972800
H	1.60305000	5.27651700	-2.34449700
H	3.04487900	4.90981000	-1.35750400
C	-4.83351400	3.18059800	-0.81504000
H	-4.80757600	4.05279600	-0.14725100
H	-5.76333100	2.63225200	-0.65606600
H	-4.78600400	3.52062800	-1.85878100
C	-2.04634900	0.33591700	-0.01425700
C	-2.51030400	0.06683000	1.30686500
C	-3.33739200	-1.03604800	1.52530900
H	-3.66026100	-1.26239000	2.53704100
C	-3.73597800	-1.88215800	0.49255400
C	-3.30825600	-1.58943400	-0.80512300
H	-3.60722100	-2.24463200	-1.61704400
C	-2.47546600	-0.51148600	-1.08407100
C	-2.16359100	0.98102200	2.47315500
H	-1.32060000	1.60403900	2.16212400
C	-3.33819100	1.92128500	2.80728800
H	-3.62566900	2.52083100	1.93989200
H	-3.06797400	2.59622600	3.62877500
H	-4.21522800	1.34121200	3.11852300
C	-1.72984900	0.19345600	3.72343400
H	-2.58667500	-0.28028500	4.21580100
H	-1.26395000	0.86633300	4.45303400

H	-1.01822200	-0.59737100	3.46674600
C	-4.58404500	-3.10911900	0.77243900
H	-4.76357500	-3.14298100	1.85573200
C	-5.95332100	-3.02556900	0.07252200
H	-6.50719100	-2.13120600	0.38106200
H	-6.56164500	-3.90623200	0.31094400
H	-5.83578100	-2.98801100	-1.01732800
C	-3.82940300	-4.39506100	0.38262100
H	-3.70665600	-4.45818800	-0.70563800
H	-4.38990600	-5.27999200	0.70844100
H	-2.82987300	-4.40048800	0.82455900
C	-2.06780500	-0.24424800	-2.52621600
H	-1.27563000	0.50872300	-2.51275700
C	-3.24345500	0.33679600	-3.33456400
H	-4.07090300	-0.38109400	-3.38193400
H	-2.93139600	0.56354400	-4.36143400
H	-3.62297400	1.25365300	-2.87390900
C	-1.49730200	-1.50242200	-3.20586000
H	-0.74636000	-1.98029600	-2.56979300
H	-1.03411600	-1.24042500	-4.16462500
H	-2.28051700	-2.24175800	-3.40795800
Pd	-0.04495200	-1.15933100	0.37306800
F	-1.01694000	-2.85270300	0.74397600

INT12

P	1.21430600	-0.77905000	-0.26121600
F	4.56156700	5.14478400	0.65259100
O	1.83454800	-3.37892800	0.95758600
O	-3.23914600	-1.78161800	2.38836800
C	1.54249700	2.53418300	-0.51501100
C	2.46159000	3.05136300	-1.44768600
H	2.40646600	2.75146600	-2.49179100
C	3.48353000	3.92509200	-1.06465800
H	4.20248200	4.31121900	-1.78063000
C	3.57180600	4.30568400	0.26927500
C	2.66389000	3.84892400	1.21563000
H	2.75684900	4.17165700	2.24824400
C	1.65199600	2.97101300	0.81049100
H	0.96930800	2.60053700	1.55962000
C	1.68636700	-1.84212100	-1.76728700
H	1.69313600	-1.09399000	-2.56973700
C	0.56988900	-2.84995100	-2.10688700
H	0.52795100	-3.61797500	-1.32389300
H	-0.38223800	-2.32477200	-2.12983500

C	0.83337500	-3.51550200	-3.46546000
H	0.04085400	-4.24474700	-3.67704800
H	0.77325200	-2.74978700	-4.25219500
C	2.21148100	-4.18891800	-3.51009700
H	2.40134400	-4.61716700	-4.50270800
H	2.22802000	-5.02633800	-2.79585300
C	3.31936000	-3.19340300	-3.13791300
H	3.37460100	-2.40773000	-3.90608600
H	4.29756800	-3.69185200	-3.12802500
C	3.06013800	-2.54228700	-1.76663600
H	3.07487900	-3.31539500	-0.99386600
H	3.87176100	-1.84484400	-1.54093200
C	2.76772400	-0.62744100	0.79447400
H	3.16924600	-1.64280700	0.82088900
C	3.85571400	0.28438200	0.18873600
H	3.52433600	1.32049100	0.19515900
H	4.05536600	0.02987800	-0.85762700
C	5.14599100	0.18132400	1.01846500
H	5.53311600	-0.84842400	0.98160100
H	5.91682200	0.82732200	0.58017000
C	4.88380000	0.58060500	2.47808400
H	4.58698000	1.63883800	2.50585000
H	5.80130700	0.49051600	3.07330400
C	3.76392800	-0.27240700	3.09195300
H	3.55146900	0.05375700	4.11821800
H	4.09943900	-1.31875600	3.15664200
C	2.47649600	-0.21389700	2.25201900
H	1.71499600	-0.86309400	2.69697000
H	2.07966900	0.80671000	2.27051800
C	0.06253700	-1.80043500	0.81261400
C	0.58800600	-2.99845900	1.37770800
C	-0.13522400	-3.75226400	2.29299100
H	0.27985400	-4.65549100	2.72222600
C	-1.41771900	-3.35487000	2.66512800
H	-1.96681700	-3.95175000	3.38311100
C	-1.97731800	-2.22160800	2.09175700
C	-1.26186900	-1.43371400	1.14544700
C	2.45664900	-4.50825900	1.54661300
H	1.89797300	-5.43050300	1.34020500
H	2.57006100	-4.38483500	2.63175200
H	3.44345900	-4.57607600	1.08451500
C	-3.97540900	-2.45528900	3.39297700
H	-4.19417700	-3.49431100	3.10950100
H	-4.91331400	-1.90568500	3.48901600

H	-3.44723700	-2.44534500	4.35664200
C	-2.09683300	-0.33967100	0.53108200
C	-2.96881400	-0.69960000	-0.52412200
C	-3.89177700	0.24328400	-0.98706900
H	-4.56574600	-0.03154100	-1.79407700
C	-3.99436400	1.51691600	-0.43594900
C	-3.13140600	1.84763900	0.61288900
H	-3.21802900	2.83222300	1.05708000
C	-2.19300400	0.94404100	1.12425100
C	-3.01605000	-2.10650400	-1.11970100
H	-2.09578100	-2.62525600	-0.83816800
C	-4.20088400	-2.90464200	-0.53509500
H	-4.14992200	-2.97040400	0.55329500
H	-4.21684100	-3.92072100	-0.94881500
H	-5.15026400	-2.42037700	-0.79526900
C	-3.09738100	-2.12064600	-2.65822200
H	-4.08088700	-1.78798300	-3.01216700
H	-2.95508800	-3.14525800	-3.02455500
H	-2.33303600	-1.47182900	-3.08704700
C	-5.06066500	2.49228200	-0.91403500
H	-5.57666300	2.02206400	-1.76244400
C	-6.10921800	2.73935500	0.18801300
H	-6.55606400	1.79704700	0.52408800
H	-6.91186500	3.39215300	-0.17628400
H	-5.65316200	3.22262000	1.06058100
C	-4.46630300	3.82098500	-1.41217100
H	-3.92161800	4.33744300	-0.61262300
H	-5.26037900	4.49267000	-1.76056200
H	-3.76843400	3.65760200	-2.23859500
C	-1.34023700	1.35222400	2.33043000
H	-0.32049400	0.98979900	2.14547000
C	-1.81880100	0.71378900	3.65378300
H	-2.88475900	0.91405600	3.81007300
H	-1.26186700	1.14240300	4.49624900
H	-1.67060000	-0.36511700	3.67123500
C	-1.27943100	2.87683700	2.54258100
H	-1.04442100	3.42090800	1.62333700
H	-0.51448400	3.12008500	3.28918400
H	-2.23302000	3.25691400	2.92743600
Pd	0.21393900	1.16170000	-1.24685100
F	-0.82539400	-0.18998300	-2.33426800
C	-0.67984900	2.68305000	-2.04401000
O	-1.21314700	3.58775000	-2.49159800

INT13

P	-0.31949400	-1.45110900	-0.05742600
O	-2.37936000	-3.23068100	1.16592500
O	-3.76986500	2.10975600	1.41623400
C	-0.68336600	-2.62907600	-1.48848200
H	0.07840200	-2.31884900	-2.21462500
C	-2.06679500	-2.35391900	-2.10827200
H	-2.85040000	-2.64613100	-1.39706800
H	-2.19062000	-1.28598500	-2.30229100
C	-2.24866400	-3.13967800	-3.41627500
H	-3.24700300	-2.94535100	-3.82914600
H	-1.52230600	-2.77561800	-4.15685000
C	-2.04032700	-4.64400600	-3.19589800
H	-2.13839200	-5.18823500	-4.14366800
H	-2.82982500	-5.02353200	-2.52926100
C	-0.67022700	-4.91865100	-2.56098900
H	0.12121100	-4.62485800	-3.26522800
H	-0.54270200	-5.99265800	-2.37269000
C	-0.48741000	-4.13872500	-1.24621700
H	-1.21481600	-4.49301900	-0.50795000
H	0.51095100	-4.34428500	-0.84572700
C	0.55956400	-2.48261700	1.24186100
H	0.07042000	-3.46111000	1.21793400
C	2.04964900	-2.65524400	0.89213500
H	2.53842700	-1.67553300	0.93787400
H	2.17975100	-3.01470100	-0.13472700
C	2.74219700	-3.60184100	1.88390600
H	2.30699300	-4.60857700	1.79519500
H	3.80441700	-3.69096400	1.62382500
C	2.58474700	-3.09972500	3.32650200
H	3.12535600	-2.14777900	3.43346800
H	3.04312400	-3.80455300	4.03159000
C	1.10621600	-2.87650500	3.67752100
H	1.01123800	-2.46882800	4.69212600
H	0.58115800	-3.84354700	3.67511000
C	0.42352900	-1.93296200	2.67308500
H	-0.62991700	-1.80096100	2.94124000
H	0.89822100	-0.94730200	2.72985400
C	-1.92793000	-0.96028500	0.71311800
C	-2.79984000	-1.93106000	1.26084500
C	-3.99889900	-1.55333400	1.85517600
H	-4.67280800	-2.29008900	2.27553800
C	-4.34890200	-0.20159600	1.91770400
H	-5.28677600	0.07351900	2.38513800

C	-3.50170800	0.76748500	1.38876600
C	-2.27660200	0.39950600	0.77653000
C	-3.20531600	-4.25952600	1.68642200
H	-4.18135600	-4.28448000	1.18394000
H	-3.35531500	-4.14555500	2.76818700
H	-2.67512500	-5.19326900	1.49057300
C	-4.98329200	2.54660500	2.00593900
H	-5.85713000	2.13756700	1.48037500
H	-4.98218000	3.63379800	1.91301500
H	-5.03781500	2.27276000	3.06859700
C	-1.44338400	1.53907800	0.24319700
C	-1.77656200	2.11871400	-1.01218100
C	-1.21255800	3.34632100	-1.36451200
H	-1.43926100	3.76946700	-2.33891100
C	-0.33487600	4.02924300	-0.52530200
C	-0.04241100	3.46676300	0.72002400
H	0.63333200	3.99075900	1.38919200
C	-0.57158200	2.24286100	1.12460000
C	-2.75450800	1.45086800	-1.96904100
H	-3.02168500	0.47539600	-1.55090600
C	-4.05727300	2.26517700	-2.07898900
H	-4.50614000	2.40961500	-1.09099600
H	-4.78074600	1.75075800	-2.72321500
H	-3.86572500	3.25483900	-2.50994600
C	-2.12177300	1.21084300	-3.35261400
H	-1.91950300	2.15629900	-3.86808400
H	-2.79731600	0.62220000	-3.98548000
H	-1.16740400	0.68009400	-3.26309900
C	0.31158300	5.33159200	-0.96209400
H	-0.03944900	5.54529600	-1.98066600
C	-0.11314100	6.50659500	-0.06195600
H	-1.20233200	6.62977000	-0.05318500
H	0.33652300	7.44334000	-0.41254700
H	0.21425400	6.34718800	0.97270400
C	1.84512800	5.19105100	-1.01831600
H	2.25890500	5.05357900	-0.01103000
H	2.29786800	6.09516600	-1.44358400
H	2.12325000	4.31875900	-1.61510300
C	-0.25687800	1.72208300	2.52018000
H	-0.62650800	0.69553900	2.58827700
C	-1.00805500	2.54838300	3.58208600
H	-0.66143300	3.58869600	3.58062300
H	-0.84138100	2.13574100	4.58472200
H	-2.08352500	2.55404100	3.37827300

C	1.25554900	1.69062000	2.80427800
H	1.79169000	1.13004900	2.03054600
H	1.45129600	1.22056900	3.77587400
H	1.68140400	2.69994900	2.83757300
Pd	0.66572800	0.44171600	-0.87290600
F	1.45527300	2.08190500	-1.72439300
C	2.23594700	-0.55465400	-1.59204400
C	3.57011500	-0.18491600	-1.00805500
C	3.80378900	1.02906700	-0.34186000
C	4.59198600	-1.14782900	-1.07405200
C	5.03717600	1.27078800	0.25953800
H	3.03037200	1.78885100	-0.35221400
C	5.82080700	-0.92217300	-0.46168800
H	4.40044700	-2.07802300	-1.59894500
C	6.01924900	0.28709200	0.20034000
H	5.24941200	2.20686100	0.76564200
H	6.61962300	-1.65615200	-0.48622100
O	2.13297700	-1.43673500	-2.42127200
F	7.20673100	0.51187000	0.79937600

INT14

P	1.54164600	0.20593200	-0.59796400
O	3.16829800	-0.38897000	1.91441800
O	-2.25895800	-1.01888300	2.73012100
C	2.71768700	-1.12421200	-1.27562200
H	2.65661600	-0.91681600	-2.35408700
C	2.12271300	-2.52530500	-1.04999900
H	2.13675600	-2.75302400	0.02564000
H	1.07668700	-2.53508300	-1.36465800
C	2.90564700	-3.60470000	-1.81298100
H	2.47912100	-4.59402800	-1.60192600
H	2.78799700	-3.43452400	-2.89346600
C	4.39843200	-3.57409700	-1.45727400
H	4.94890300	-4.32117200	-2.04345400
H	4.52206400	-3.84837400	-0.39865000
C	4.98597900	-2.17339100	-1.68343900
H	4.94695000	-1.93316300	-2.75650200
H	6.04554800	-2.15203200	-1.39593300
C	4.21007500	-1.10517700	-0.89179900
H	4.31175900	-1.30295700	0.17838100
H	4.65076700	-0.11946700	-1.07889800
C	2.67158000	1.66315600	-0.24761200
H	3.56986900	1.28745900	0.25181800
C	3.07279500	2.32283000	-1.58261400

H	2.16025300	2.64597300	-2.10362300
H	3.56598400	1.59746100	-2.24260500
C	3.99339300	3.53243500	-1.35126400
H	4.94014200	3.18797500	-0.90832900
H	4.24667000	3.99882900	-2.31206700
C	3.34053500	4.55838800	-0.41344100
H	2.44792400	4.97543600	-0.90318000
H	4.02241700	5.39900100	-0.23116900
C	2.92495000	3.90632500	0.91313700
H	2.42066400	4.63785000	1.55804800
H	3.82601900	3.57739300	1.45262300
C	2.00753700	2.69582400	0.67877800
H	1.74583400	2.23368200	1.63768300
H	1.06986500	3.03511200	0.22089300
C	0.95028300	-0.35983000	1.09402400
C	1.83723900	-0.58462700	2.17503800
C	1.36616600	-0.96593300	3.42812200
H	2.04605000	-1.13830000	4.25396800
C	-0.00445500	-1.12757800	3.63831700
H	-0.35229100	-1.42381900	4.62069600
C	-0.89870500	-0.89897000	2.59781500
C	-0.43748800	-0.51002100	1.31463400
C	4.10575800	-0.53356100	2.96589100
H	4.11182900	-1.55596600	3.36791300
H	3.90871300	0.17474500	3.78208100
H	5.08075700	-0.31588100	2.52523600
C	-2.78900700	-1.36562400	3.99708200
H	-2.44249900	-2.35550500	4.32567800
H	-3.87249500	-1.38844500	3.86801400
H	-2.53009100	-0.61979800	4.76165700
C	-1.53628700	-0.26052100	0.29095400
C	-2.17321400	-1.37869000	-0.33864000
C	-3.42692900	-1.21335900	-0.93908900
H	-3.89766300	-2.07518600	-1.40695400
C	-4.10868600	0.00577300	-0.94100800
C	-3.48491200	1.09585400	-0.32508700
H	-3.99694100	2.05411800	-0.30120900
C	-2.23636400	0.99592400	0.30474600
C	-1.54395400	-2.76562900	-0.30476900
H	-0.52991500	-2.65933400	0.09071900
C	-2.31241900	-3.70350800	0.64626500
H	-2.37014300	-3.27499900	1.65069100
H	-1.81984100	-4.68203700	0.70814500
H	-3.33767600	-3.86309700	0.29062800

C	-1.42891900	-3.38501500	-1.70932200
H	-2.41512100	-3.61279200	-2.13059000
H	-0.86137200	-4.32307100	-1.67041700
H	-0.92224100	-2.69952100	-2.39784300
C	-5.47525600	0.14106300	-1.59176100
H	-5.74582700	-0.84505800	-1.99464900
C	-6.55699400	0.53620000	-0.56875600
H	-6.59874900	-0.18338200	0.25650300
H	-7.54619900	0.57797500	-1.04128200
H	-6.35038500	1.52406900	-0.13959800
C	-5.44632900	1.13177900	-2.77081900
H	-5.19298700	2.14210000	-2.42769300
H	-6.42410100	1.18118000	-3.26586900
H	-4.69772600	0.83480800	-3.51407700
C	-1.71453800	2.18511100	1.10715700
H	-0.68959200	1.95657700	1.41070700
C	-2.54511300	2.38039500	2.39113900
H	-3.58206900	2.64169300	2.14675400
H	-2.12733500	3.18847900	3.00462100
H	-2.56435500	1.46247400	2.98528500
C	-1.66931800	3.48348200	0.28369900
H	-1.09739600	3.33816000	-0.63976800
H	-1.19705100	4.28663800	0.86274500
H	-2.67465100	3.82330300	0.00808500
Pd	-0.46130700	0.48890100	-1.57266500

INT15

P	1.49724700	0.04693200	-0.61059900
O	3.03236700	-1.37669000	1.66711200
O	-2.42415200	-2.03710100	2.19398500
C	1.74424300	1.73281600	0.34780500
H	0.70685800	2.09744200	0.39438900
C	2.54207200	2.83794200	-0.37836400
H	3.60693400	2.58248100	-0.39391900
H	2.21668600	2.92723400	-1.41523200
C	2.38054600	4.18717600	0.34397600
H	2.95234500	4.96025700	-0.18583000
H	1.32459100	4.49494200	0.30590600
C	2.83021800	4.09576100	1.80740100
H	2.68185700	5.05523200	2.31970400
H	3.90954300	3.88344500	1.83888400
C	2.07587300	2.97744900	2.53652700
H	1.01104700	3.24234800	2.59939000
H	2.43651800	2.87908100	3.56910400

C	2.21144400	1.62257700	1.81623000
H	3.25617000	1.29640300	1.85699200
H	1.62803800	0.87738200	2.36122100
C	3.19520800	-0.65659400	-1.06921100
H	3.14203800	-1.66556000	-0.64825000
C	3.36732000	-0.81068800	-2.60018300
H	3.48076700	0.18034000	-3.05627700
H	2.48054800	-1.26512800	-3.04280800
C	4.60686900	-1.66874000	-2.90824500
H	4.44368800	-2.68558100	-2.52055000
H	4.72902500	-1.76452300	-3.99505300
C	5.88173400	-1.08780300	-2.28066300
H	6.11603600	-0.12941500	-2.76720200
H	6.73790700	-1.75091600	-2.46099300
C	5.69444300	-0.84500500	-0.77617000
H	6.58729700	-0.36588400	-0.35261000
H	5.57685900	-1.81011800	-0.26026900
C	4.45438500	0.02419500	-0.50525600
H	4.36279500	0.21883500	0.56303700
H	4.59858900	0.99007100	-1.00300800
C	0.82808100	-0.97327500	0.89062200
C	1.68927200	-1.60181600	1.82321100
C	1.18585500	-2.38177300	2.86159200
H	1.85191500	-2.86185700	3.56840600
C	-0.19025500	-2.55295000	3.01134000
H	-0.56065200	-3.16445000	3.82529900
C	-1.05678900	-1.93130500	2.12094300
C	-0.55956300	-1.13406400	1.06015100
C	3.94325200	-1.92151200	2.60241900
H	3.91333100	-3.01967100	2.60709000
H	3.75009800	-1.55055300	3.61849800
H	4.93203600	-1.59173600	2.27742800
C	-2.98613200	-2.82951100	3.22572900
H	-2.66184400	-3.87666100	3.15237400
H	-4.06717000	-2.77627400	3.08522100
H	-2.72953700	-2.43962000	4.22057400
C	-1.62449600	-0.51801700	0.17779300
C	-2.32251000	-1.36710900	-0.75549200
C	-3.53359600	-0.94290300	-1.28863400
H	-4.05694400	-1.59552000	-1.98325500
C	-4.10610200	0.30460800	-0.97933400
C	-3.43171600	1.13548800	-0.09331600
H	-3.87071400	2.09002100	0.17916800
C	-2.20848800	0.76025400	0.53110500

C	-1.77374600	-2.73664800	-1.13939300
H	-0.79196700	-2.84698000	-0.67392000
C	-2.67932200	-3.86822800	-0.61622600
H	-2.80889100	-3.79719900	0.46719800
H	-2.24366500	-4.84642100	-0.85368700
H	-3.67339200	-3.82385500	-1.07773900
C	-1.56551500	-2.85908800	-2.66016300
H	-2.51560000	-2.79178400	-3.20403800
H	-1.11390800	-3.82951300	-2.90067800
H	-0.89993200	-2.07053300	-3.01912900
C	-5.42230100	0.73082700	-1.61144500
H	-5.77114800	-0.10356100	-2.23530200
C	-6.50674700	1.00337000	-0.55284400
H	-6.65700600	0.12983600	0.09115300
H	-7.46292600	1.24839400	-1.03073000
H	-6.22947200	1.84840400	0.08864900
C	-5.23052500	1.95117200	-2.53171100
H	-4.88852700	2.82163600	-1.95899900
H	-6.17253000	2.22104300	-3.02452700
H	-4.48227600	1.74599900	-3.30548000
C	-1.76829500	1.53649900	1.77876200
H	-0.75595800	1.20848800	2.03507500
C	-2.68647600	1.19112200	2.96882100
H	-3.70993700	1.53450000	2.77422100
H	-2.33112300	1.68182800	3.88317500
H	-2.72279600	0.11321700	3.13781100
C	-1.73285100	3.06035300	1.57230700
H	-1.09694300	3.34116100	0.72658100
H	-1.34592200	3.55258000	2.47197500
H	-2.73362900	3.46724300	1.38627200
F	0.78102500	-1.18025800	-1.57995100
Pd	-0.73776300	0.97834000	-1.14886300
F	1.33689100	1.01900300	-2.11981600

INT16

P	1.36608500	0.07733400	-0.37051800
O	2.87607900	-2.55040700	-0.08120400
O	-2.59590400	-3.03174800	0.55224300
C	1.79265900	0.62426200	1.45329700
H	0.80140300	0.63557400	1.91256100
C	2.33548900	2.06459000	1.57127300
H	3.35071400	2.11987300	1.16293300
H	1.72183200	2.75257500	0.98274700
C	2.38387400	2.50822800	3.04393900

H	2.80745200	3.51859700	3.11152000
H	1.35916900	2.57158000	3.43688400
C	3.19321300	1.52917200	3.90584000
H	3.18499000	1.84281200	4.95797900
H	4.24435100	1.54476400	3.58003000
C	2.65078900	0.09974600	3.77169400
H	1.62870600	0.06016400	4.17823600
H	3.25316300	-0.59841900	4.36867400
C	2.62708500	-0.36257200	2.30182100
H	3.65523200	-0.42166000	1.93367200
H	2.20909100	-1.37297900	2.24698100
C	2.99749200	0.30141400	-1.29258700
H	2.94131200	-0.48907400	-2.04672300
C	2.94397200	1.66570500	-2.03077200
H	2.99033800	2.48242900	-1.30098900
H	1.99239700	1.77224500	-2.55353500
C	4.11242800	1.78458600	-3.02178300
H	3.99026700	1.02912600	-3.81247300
H	4.07704200	2.76497700	-3.51349600
C	5.46842600	1.57925200	-2.33333500
H	5.63969600	2.40019700	-1.62118400
H	6.28456300	1.62123900	-3.06630400
C	5.49876500	0.24570400	-1.57432200
H	6.45606200	0.12274200	-1.05060100
H	5.42305200	-0.58435700	-2.29305600
C	4.34289600	0.14557000	-0.56121000
H	4.38986200	-0.81108800	-0.04012800
H	4.47417800	0.93194800	0.18762300
C	0.72010800	-1.61628400	0.00458300
C	1.53896300	-2.75465600	0.12206700
C	0.97587300	-3.99348900	0.42154000
H	1.59434600	-4.87745900	0.52245800
C	-0.40727500	-4.11411100	0.58603200
H	-0.82334200	-5.08770100	0.81574200
C	-1.22902100	-2.99918100	0.43906700
C	-0.66979200	-1.73733500	0.13832700
C	3.76491500	-3.64451200	0.06646800
H	3.54803000	-4.43981900	-0.65907100
H	3.72744900	-4.05891100	1.08309500
H	4.76261200	-3.24500300	-0.12460000
C	-3.22144200	-4.27915300	0.80907400
H	-3.01618900	-5.00256400	0.00849300
H	-4.29240200	-4.07261100	0.84439900
H	-2.90022700	-4.70051500	1.77128300

C	-1.61856200	-0.58268300	-0.09414900
C	-2.30741800	-0.50053800	-1.36207500
C	-3.38485900	0.37144300	-1.49121100
H	-3.89358800	0.44024100	-2.44825800
C	-3.82715600	1.18553500	-0.43477000
C	-3.18558000	1.07974600	0.79930200
H	-3.53099100	1.68488800	1.63032700
C	-2.11040000	0.19030800	1.01669300
C	-1.92033800	-1.38346700	-2.54075500
H	-0.97596200	-1.87151900	-2.29719000
C	-2.98923600	-2.47289000	-2.76237500
H	-3.14377800	-3.06589800	-1.85683800
H	-2.68164100	-3.14388000	-3.57324900
H	-3.95151600	-2.02653800	-3.04186600
C	-1.68889400	-0.57125100	-3.82695700
H	-2.61256800	-0.10196200	-4.18656500
H	-1.32759100	-1.23510400	-4.62141800
H	-0.93925400	0.20327800	-3.65493100
C	-4.95060700	2.18436500	-0.65047700
H	-5.34374600	2.02131800	-1.66296100
C	-6.11210400	1.98191200	0.33924600
H	-6.50369100	0.95987400	0.28893300
H	-6.93028100	2.67568600	0.11379800
H	-5.79432600	2.17077800	1.37137300
C	-4.40741700	3.62574800	-0.58456400
H	-4.01835200	3.84982000	0.41567900
H	-5.20104100	4.34811900	-0.80922900
H	-3.58999400	3.77362500	-1.29861400
C	-1.65550400	-0.06420600	2.45130900
H	-0.71349000	-0.61920400	2.41011800
C	-2.68512300	-0.95814600	3.17245300
H	-3.64937000	-0.44216800	3.25428500
H	-2.33859400	-1.19360200	4.18577500
H	-2.84611100	-1.89088300	2.62739900
C	-1.41717800	1.22945000	3.24976700
H	-0.79228500	1.93608500	2.69551500
H	-0.92126900	0.99710700	4.19902500
H	-2.36049400	1.73316700	3.49017200
F	0.72234500	-0.19177400	-2.05787500
Pd	-0.51309900	1.37937700	-0.35674200
F	0.39992500	3.10959800	-0.68318600

TS1

P	1.28520400	0.35553400	-0.44824900

O	3.08409800	-0.95381000	1.45783200
O	-2.20202700	-2.02921900	2.63071100
C	2.34568100	-0.70441000	-1.59678000
H	1.95099200	-0.41694500	-2.57870800
C	2.08732800	-2.21444300	-1.42599000
H	2.46626900	-2.54302900	-0.45111900
H	1.01527000	-2.42200600	-1.44087700
C	2.78699500	-3.01252200	-2.53687500
H	2.60498600	-4.08507800	-2.39222300
H	2.34301400	-2.74507200	-3.50637800
C	4.29369200	-2.72149200	-2.56811500
H	4.77505000	-3.27275600	-3.38571500
H	4.74912500	-3.08122300	-1.63289200
C	4.55954300	-1.21627600	-2.70990800
H	4.19582500	-0.87454800	-3.68971000
H	5.63803900	-1.01301900	-2.68708700
C	3.85784800	-0.40879200	-1.60390600
H	4.28530500	-0.67589700	-0.63132700
H	4.04685400	0.65841700	-1.76005900
C	2.37688500	1.69626300	0.30369100
H	3.34824200	1.22535900	0.48319500
C	2.58030600	2.89732000	-0.64735800
H	1.61056100	3.36600800	-0.84884400
H	2.96465900	2.57145400	-1.61735600
C	3.53799600	3.92370900	-0.02184400
H	4.53141500	3.46589700	0.10034700
H	3.66523300	4.77107200	-0.70716900
C	3.03359100	4.40931600	1.34291300
H	2.09005600	4.95686200	1.20344900
H	3.74627600	5.11482600	1.78864500
C	2.78970400	3.22424100	2.28626300
H	2.37854600	3.57160500	3.24280100
H	3.74934900	2.73594200	2.51366700
C	1.83754300	2.19291700	1.66076000
H	1.69343100	1.35937500	2.35452600
H	0.85772100	2.65873900	1.50938100
C	0.77826300	-0.72129800	0.98044100
C	1.79561900	-1.25881200	1.81176200
C	1.47889200	-2.04464000	2.91268400
H	2.25390500	-2.45437100	3.54892300
C	0.14227300	-2.31826900	3.21192100
H	-0.08473400	-2.93295100	4.07462800
C	-0.86960000	-1.80632500	2.40783700
C	-0.56803200	-0.99989300	1.28150000

C	4.15221900	-1.44350200	2.25235200
H	4.17062200	-2.54117800	2.27010700
H	4.09486900	-1.06298400	3.28062400
H	5.06503000	-1.07570000	1.78019300
C	-2.57723900	-2.82895200	3.73998600
H	-2.17403300	-3.84776900	3.65847800
H	-3.66748400	-2.86988800	3.71864800
H	-2.24908800	-2.38302500	4.68907300
C	-1.72644100	-0.50145000	0.46910000
C	-2.27551900	-1.32231100	-0.53620600
C	-3.35514200	-0.84083700	-1.27897100
H	-3.76038300	-1.45154600	-2.08156700
C	-3.89253200	0.42600000	-1.06626200
C	-3.37563900	1.19295200	-0.02024900
H	-3.79699100	2.17446600	0.17259900
C	-2.31009600	0.75005100	0.76328400
C	-1.74165400	-2.72056700	-0.82639600
H	-0.78925800	-2.83566400	-0.29770700
C	-2.70158300	-3.79002700	-0.26994400
H	-2.86456400	-3.64566100	0.80222100
H	-2.29977200	-4.79788200	-0.43442200
H	-3.67576500	-3.72889500	-0.77013600
C	-1.46894200	-2.95326000	-2.32440600
H	-2.40291500	-3.02346300	-2.89363600
H	-0.92839200	-3.89720700	-2.46868800
H	-0.88532200	-2.13276700	-2.74972900
C	-4.93294600	0.99066000	-2.01813900
H	-5.28151300	0.16385900	-2.65266700
C	-6.16138000	1.57760100	-1.30421100
H	-6.63444200	0.83458100	-0.65222400
H	-6.90494000	1.91735200	-2.03514800
H	-5.89114800	2.44191300	-0.68631000
C	-4.26335200	2.03422000	-2.93538600
H	-3.92157800	2.89571300	-2.34888000
H	-4.96319500	2.39550900	-3.69935600
H	-3.38385400	1.60697600	-3.42820400
C	-1.84912100	1.58949100	1.95037300
H	-0.84183300	1.26147200	2.22630100
C	-2.76037800	1.32546800	3.16647300
H	-3.78597200	1.64899400	2.95068000
H	-2.40677600	1.87783100	4.04642200
H	-2.79023300	0.25901300	3.40705800
C	-1.77834100	3.09634500	1.64809800
H	-1.21818500	3.29120900	0.72984300

H	-1.29410100	3.62558700	2.47789400
H	-2.77736500	3.53157600	1.52879600
C	-0.09344300	1.15163300	-2.00974000
F	0.97187400	1.75528300	-2.70695400
F	-0.68394700	2.20497300	-1.33985700
O	-0.74259700	0.30215000	-2.56577100

TS2

P	1.36694000	0.15037700	-0.50413200
O	2.96581800	-0.70657100	1.64589200
O	-2.34514800	-0.76967000	3.13913300
C	1.98668200	-1.47198300	-1.24888900
H	1.30366200	-1.51879600	-2.10409700
C	1.74175500	-2.72837200	-0.39224200
H	2.43083600	-2.73993100	0.45904300
H	0.72826000	-2.73715800	0.01346800
C	1.96765500	-3.99240800	-1.23796700
H	1.79649500	-4.88159800	-0.61763000
H	1.22221200	-4.02330100	-2.04490400
C	3.37851400	-4.01807400	-1.84018700
H	3.51288500	-4.90929100	-2.46604200
H	4.11549700	-4.08956900	-1.02558500
C	3.64778100	-2.74517300	-2.65308300
H	2.98369600	-2.72467100	-3.52891500
H	4.67548600	-2.74504200	-3.03811700
C	3.41400300	-1.47300100	-1.81997800
H	4.14299900	-1.44346100	-1.00018600
H	3.55807200	-0.59055100	-2.44463800
C	2.50716400	1.58518000	-0.04538800
H	2.56228900	1.51693000	1.04479100
C	3.93816500	1.47919700	-0.59750400
H	3.89121900	1.51906600	-1.69026400
H	4.38611100	0.52486300	-0.31258100
C	4.81412200	2.62555000	-0.06444400
H	4.92033100	2.52153600	1.02637800
H	5.82319300	2.53756400	-0.48712900
C	4.21272700	3.99934900	-0.38415900
H	4.20239400	4.14344400	-1.47398400
H	4.83522300	4.79877300	0.03755600
C	2.77825000	4.09226200	0.14776800
H	2.32885200	5.05682800	-0.11974100
H	2.79418100	4.04757700	1.24760800
C	1.89783500	2.95820300	-0.39920100
H	0.89342000	3.04899900	0.01330700

H	1.80771800	3.03068800	-1.48529500
C	0.68811000	-0.31847800	1.17146800
C	1.67232800	-0.71958800	2.11148500
C	1.32321600	-1.11193100	3.39583600
H	2.07544700	-1.41681400	4.11360000
C	-0.02303600	-1.12824100	3.77041600
H	-0.28256200	-1.44450400	4.77356900
C	-1.00541800	-0.75437500	2.85926100
C	-0.66601700	-0.33486500	1.54774300
C	4.01114500	-1.15692300	2.49331300
H	3.86050500	-2.20337500	2.78938800
H	4.09711500	-0.53166400	3.39136200
H	4.92595800	-1.07303700	1.90446600
C	-2.76353800	-1.18922800	4.42774300
H	-2.46072400	-2.22517100	4.63287000
H	-3.85290400	-1.12660400	4.41902800
H	-2.36870500	-0.53233900	5.21496000
C	-1.79368900	0.02419600	0.62498200
C	-2.46443900	-1.00181200	-0.07383700
C	-3.49345500	-0.65866700	-0.94999700
H	-3.98379600	-1.44555400	-1.51598500
C	-3.87902500	0.66659600	-1.15122500
C	-3.24721000	1.65624800	-0.39894700
H	-3.55466100	2.68789900	-0.54027900
C	-2.21242400	1.36573600	0.49363500
C	-2.11054300	-2.47387200	0.10389500
H	-1.21855300	-2.53528200	0.73569200
C	-3.23663000	-3.21489500	0.84936800
H	-3.44087100	-2.73596500	1.81206600
H	-2.96103400	-4.26195300	1.02735600
H	-4.16367400	-3.20574700	0.26377200
C	-1.77824600	-3.16276100	-1.23233700
H	-2.67042500	-3.26078200	-1.86163700
H	-1.38823300	-4.17255600	-1.05418700
H	-1.04170300	-2.58595300	-1.79759200
C	-4.90092100	1.03387000	-2.21396500
H	-5.09038700	2.11309100	-2.12990000
C	-4.31502200	0.76817200	-3.61487000
H	-3.34551900	1.26301500	-3.73285300
H	-4.99531600	1.12471600	-4.39819400
H	-4.15166500	-0.30507500	-3.76835800
C	-6.24446800	0.30989400	-2.02412100
H	-6.96813800	0.63429000	-2.78153800
H	-6.66807400	0.51295900	-1.03410800

H	-6.12858100	-0.77594500	-2.12243400
C	-1.59059200	2.48179700	1.32891700
H	-0.55131400	2.20239200	1.54071900
C	-2.31709900	2.60467600	2.68438000
H	-3.36132500	2.89995100	2.52464800
H	-1.83985700	3.36731500	3.31253300
H	-2.32087200	1.65489600	3.22390700
C	-1.56904600	3.84830100	0.62220100
H	-1.18528800	3.76561700	-0.39765400
H	-0.93565500	4.54833000	1.17970600
H	-2.57090800	4.29049700	0.57309200
C	0.09094500	0.59171100	-1.81464600
F	1.67203100	0.90364800	-2.57454600
F	-0.34752200	1.88819100	-1.71008000
O	-0.56509900	-0.21994500	-2.41377300

TS2'

P	1.48746400	0.21798200	-0.58012900
O	2.97845900	-0.59867200	1.62182900
O	-2.37181800	-1.17426200	2.78058000
C	2.54423500	-1.19188200	-1.29958600
H	2.27793700	-1.11846700	-2.36500700
C	2.08731300	-2.58556200	-0.82311600
H	2.32885000	-2.69966800	0.24032800
H	1.00225700	-2.68358900	-0.91824200
C	2.77967400	-3.69794900	-1.62515300
H	2.46293600	-4.67761800	-1.24575700
H	2.45053400	-3.64525500	-2.67294400
C	4.30736400	-3.56419800	-1.56421500
H	4.78527600	-4.34624700	-2.16730700
H	4.64177200	-3.71601000	-0.52683700
C	4.75649300	-2.17525700	-2.03945400
H	4.50515600	-2.05783000	-3.10356600
H	5.84679000	-2.07627600	-1.96193400
C	4.07776000	-1.05489200	-1.23184300
H	4.40550400	-1.11467400	-0.18960700
H	4.39761000	-0.08069900	-1.61986700
C	2.64181100	1.63943000	-0.19467500
H	3.57714600	1.14552300	0.08262300
C	2.91163400	2.55039200	-1.41054700
H	1.96784400	3.01852400	-1.70054200
H	3.25436100	1.96659600	-2.27145900
C	3.95656900	3.61921600	-1.05211400
H	4.91916200	3.13282600	-0.83107500

H	4.12555700	4.26635500	-1.92181900
C	3.51904500	4.45213200	0.15941000
H	2.61204000	5.01578800	-0.10132600
H	4.29001600	5.18944700	0.41670900
C	3.21648200	3.54816600	1.36119700
H	2.85787000	4.14429800	2.20985600
H	4.14600800	3.05826600	1.69101700
C	2.17020600	2.47899600	1.00994600
H	1.98830700	1.84269500	1.88134900
H	1.21844600	2.94739500	0.74742900
C	0.70794800	-0.40725600	0.98346500
C	1.66274900	-0.75010700	1.97869900
C	1.27963000	-1.20582400	3.23107000
H	2.01864000	-1.45688100	3.98182600
C	-0.07386900	-1.35263700	3.52602000
H	-0.36197300	-1.71494700	4.50520600
C	-1.02822700	-1.03863100	2.56576200
C	-0.66574900	-0.54931000	1.28008700
C	3.98098000	-0.64810600	2.62563200
H	4.07922800	-1.65637100	3.04797400
H	3.77251100	0.06858700	3.42993400
H	4.91327200	-0.37630100	2.12707000
C	-2.81665000	-1.62840400	4.04799400
H	-2.44912900	-2.64038000	4.26719900
H	-3.90591100	-1.64581200	3.98689200
H	-2.50923600	-0.94622600	4.85254300
C	-1.82938500	-0.26201500	0.37396000
C	-2.40027700	-1.31852600	-0.35978900
C	-3.54139300	-1.06622700	-1.12617800
H	-3.97798800	-1.87426600	-1.70848200
C	-4.13248800	0.19365100	-1.17596700
C	-3.57242300	1.21327200	-0.40091000
H	-4.03618300	2.19514800	-0.40817900
C	-2.43879200	1.01170000	0.38665700
C	-1.83889100	-2.73643800	-0.31809000
H	-0.87963700	-2.70657700	0.20990900
C	-2.76878400	-3.66449100	0.48796400
H	-2.93723300	-3.26780900	1.49335200
H	-2.33814100	-4.67046000	0.57223900
H	-3.74439900	-3.75581400	-0.00443100
C	-1.57184200	-3.31714300	-1.71866200
H	-2.50647100	-3.48780800	-2.26552700
H	-1.06005900	-4.28471000	-1.63692800
H	-0.95890000	-2.63628600	-2.31390800

C	-5.34604200	0.45074500	-2.05531500
H	-5.62074900	-0.50500100	-2.52323000
C	-6.55984900	0.93347100	-1.24123100
H	-6.81433800	0.21863400	-0.45072600
H	-7.43671300	1.05886900	-1.88829700
H	-6.35755600	1.90009700	-0.76492600
C	-5.00889500	1.44168200	-3.18507900
H	-4.74029900	2.42286000	-2.77585100
H	-5.86762500	1.57851400	-3.85419700
H	-4.15878800	1.08560500	-3.77647400
C	-1.95315900	2.13163700	1.30222100
H	-0.92321800	1.90361100	1.58939600
C	-2.81035800	2.17426400	2.58375900
H	-3.85145900	2.41882400	2.33879500
H	-2.43709300	2.94103500	3.27476100
H	-2.80955200	1.20856400	3.09659800
C	-1.93413400	3.51385600	0.62816500
H	-1.37008800	3.46673400	-0.30408300
H	-1.45882600	4.24495700	1.29492700
H	-2.94624900	3.88178500	0.42003300
C	0.35098800	0.42863300	-2.01453800
F	0.99251500	1.20837600	-2.96014900
F	0.01414500	1.79982500	-0.75168100
O	-0.57128500	-0.25474400	-2.34619700

TS3

P	1.31723100	0.26184300	-0.68604900
O	2.97848900	-0.57001700	1.58660400
O	-2.35165600	-0.68768100	2.99332900
C	2.28370100	-1.32385200	-1.29122300
H	1.94677100	-1.41654700	-2.32866300
C	1.86685800	-2.61802600	-0.57091500
H	2.23425700	-2.60884600	0.45995100
H	0.77694700	-2.69735700	-0.51981900
C	2.43974300	-3.84942600	-1.29258500
H	2.14370000	-4.76271500	-0.76040900
H	2.00292200	-3.91621200	-2.29979700
C	3.96796000	-3.76255600	-1.40383300
H	4.36355600	-4.62888800	-1.94916700
H	4.40381100	-3.79904100	-0.39397000
C	4.39696500	-2.45827800	-2.08949800
H	4.05042500	-2.46736500	-3.13323500
H	5.49179200	-2.38519400	-2.12346400
C	3.81737200	-1.22069100	-1.37840000

H	4.24577100	-1.15017300	-0.37323600
H	4.11113600	-0.32081300	-1.92798000
C	2.70297700	1.51927000	-0.35955600
H	3.57850400	0.93210000	-0.08103000
C	3.06231000	2.35693900	-1.60574100
H	2.18726400	2.94697500	-1.90112000
H	3.29188500	1.71141800	-2.45656300
C	4.25422900	3.28285900	-1.31300300
H	5.14510700	2.66906900	-1.10846900
H	4.48471300	3.87957300	-2.20501300
C	3.98907900	4.19911600	-0.11118000
H	3.17021900	4.88981800	-0.36008600
H	4.87048400	4.81662800	0.10524200
C	3.58872600	3.37755500	1.12108000
H	3.34908400	4.03998600	1.96324700
H	4.44201700	2.75933000	1.44044100
C	2.38658700	2.46921000	0.81806000
H	2.11251500	1.91075300	1.71790700
H	1.52120500	3.08762800	0.55639600
C	0.69753400	-0.28491300	1.01244500
C	1.67010400	-0.59502000	1.99694000
C	1.31210200	-0.90900700	3.30248300
H	2.06610600	-1.13556300	4.04610400
C	-0.03199400	-0.93835500	3.66997700
H	-0.29325500	-1.18244500	4.69250700
C	-1.00831700	-0.66585700	2.71978100
C	-0.66336900	-0.34515200	1.38154400
C	4.01272400	-0.66738400	2.55057200
H	4.02519100	-1.65349500	3.03369000
H	3.92096700	0.11353000	3.31669800
H	4.94475400	-0.52741500	1.99947600
C	-2.76791100	-0.98344800	4.31583200
H	-2.45686300	-1.99181600	4.62238500
H	-3.85795400	-0.93224300	4.30019500
H	-2.38064300	-0.24938000	5.03569800
C	-1.82679700	-0.13145300	0.45155000
C	-2.44618400	-1.24914300	-0.14603800
C	-3.55327300	-1.04195000	-0.97637700
H	-4.02315000	-1.89650600	-1.45707800
C	-4.07188900	0.22845500	-1.21438000
C	-3.47754100	1.31347600	-0.56150200
H	-3.89137900	2.30695300	-0.70852100
C	-2.37618700	1.16048900	0.28029300
C	-1.98601600	-2.67948100	0.12751600

H	-1.02100000	-2.63150900	0.64220500
C	-2.97415900	-3.38437800	1.07854900
H	-3.09643300	-2.81341400	2.00232800
H	-2.62010100	-4.39239900	1.32841900
H	-3.95999000	-3.47936100	0.60748800
C	-1.78927400	-3.51744000	-1.14893000
H	-2.74148500	-3.70405200	-1.65800600
H	-1.35713100	-4.49293700	-0.89514600
H	-1.12737600	-3.02259800	-1.86407200
C	-5.24712400	0.42851200	-2.15813600
H	-5.55135900	-0.56384500	-2.51885000
C	-6.45856900	1.05421900	-1.44380400
H	-6.76970500	0.44518900	-0.58776000
H	-7.30936200	1.14499900	-2.12992500
H	-6.22294100	2.05809800	-1.07118000
C	-4.83451900	1.26283400	-3.38501100
H	-4.53780100	2.27573600	-3.08780300
H	-5.66639300	1.35039000	-4.09450100
H	-3.98359700	0.80537900	-3.90107400
C	-1.85268800	2.36274800	1.06055500
H	-0.85666400	2.11126500	1.43438900
C	-2.76068900	2.62489600	2.27948200
H	-3.76989800	2.90646800	1.95377800
H	-2.36163300	3.44397100	2.89105500
H	-2.84812900	1.73036100	2.90240600
C	-1.71043500	3.63728600	0.21215600
H	-1.11102900	3.44888800	-0.68080500
H	-1.21994300	4.42239200	0.80069400
H	-2.68525600	4.03121400	-0.09937900
C	-0.02886900	-0.30613300	-2.00039300
F	1.24715600	0.64834500	-2.73474900
F	0.23307800	1.56923000	-0.73650000
O	-0.84435900	-0.66446800	-2.73174100

TS3'

P	1.49446000	0.12094500	-0.78550200
O	3.03409100	-0.56744300	1.47165400
O	-2.22410400	-0.42816200	3.10936000
C	2.10939400	-1.61624900	-1.20660400
H	1.60015300	-1.73078800	-2.17071000
C	1.65130700	-2.76963500	-0.30240900
H	2.16529900	-2.71946000	0.66274700
H	0.58127200	-2.70358600	-0.10157000
C	1.97319900	-4.11788800	-0.96931100

H	1.64318700	-4.93728900	-0.31804200
H	1.39885800	-4.20691600	-1.90258300
C	3.47219600	-4.24288000	-1.27484800
H	3.68510900	-5.19563000	-1.77611700
H	4.03078600	-4.25274000	-0.32657100
C	3.95788200	-3.06593400	-2.13211700
H	3.48282900	-3.11821200	-3.12235600
H	5.04056600	-3.13447800	-2.29964300
C	3.62102300	-1.71002600	-1.48705100
H	4.17173200	-1.61200500	-0.54406100
H	3.92909100	-0.89697400	-2.14649000
C	2.47360300	1.65068900	-0.24248700
H	2.37485000	1.69336600	0.84497100
C	3.97249700	1.56833300	-0.59450400
H	4.07434500	1.53754800	-1.68337000
H	4.41109600	0.65046100	-0.19762800
C	4.72276700	2.78763600	-0.03425400
H	4.66276600	2.77817400	1.06484000
H	5.78743400	2.71375000	-0.29113100
C	4.13248800	4.10088700	-0.56464700
H	4.29397200	4.15287200	-1.65107800
H	4.65119300	4.96231900	-0.12479600
C	2.62725900	4.17496000	-0.27780700
H	2.19756100	5.08720200	-0.71097300
H	2.46711700	4.23919500	0.80920700
C	1.87822600	2.94971400	-0.82621100
H	0.82064800	3.03572400	-0.58144100
H	1.95131000	2.91116600	-1.91710700
C	0.75102400	-0.22209300	0.99009500
C	1.74539100	-0.52082200	1.95827900
C	1.44180400	-0.76733200	3.28921200
H	2.21762300	-0.99324500	4.01088300
C	0.11042400	-0.73652200	3.70959500
H	-0.11921400	-0.93512600	4.74944800
C	-0.89051100	-0.46166000	2.78794300
C	-0.58792700	-0.19581000	1.42431800
C	4.10413600	-0.81919200	2.36475700
H	4.01492600	-1.80994000	2.83026600
H	4.16223200	-0.05281400	3.14910900
H	5.01223700	-0.78603000	1.75995500
C	-2.60162700	-0.66957100	4.45373400
H	-2.30652400	-1.67540000	4.78340900
H	-3.68992000	-0.58883000	4.47445400
H	-2.16975900	0.07595700	5.13582300

C	-1.77722300	0.07039900	0.54230400
C	-2.52234200	-1.01839500	0.03403700
C	-3.63261900	-0.76075100	-0.76912700
H	-4.18608300	-1.59916100	-1.18293800
C	-4.04683900	0.53922800	-1.06639100
C	-3.33676200	1.59740300	-0.50154900
H	-3.66524900	2.61090300	-0.71166400
C	-2.21383900	1.39319600	0.30713300
C	-2.17110400	-2.46888300	0.35047200
H	-1.23069300	-2.47518600	0.90978600
C	-3.24117000	-3.09653200	1.26474900
H	-3.36465900	-2.50429700	2.17582300
H	-2.95870000	-4.11928800	1.54397900
H	-4.21162500	-3.14323000	0.75606700
C	-1.96568300	-3.32107800	-0.91568600
H	-2.90296800	-3.44254800	-1.47114400
H	-1.61230600	-4.32346400	-0.64392300
H	-1.23929700	-2.86136900	-1.59033500
C	-5.22157700	0.79572500	-1.99653600
H	-5.37450200	1.88294300	-2.04153100
C	-4.90365900	0.30959700	-3.42323900
H	-3.98134600	0.76693500	-3.79675200
H	-5.72099400	0.55758600	-4.11144600
H	-4.76392400	-0.77764400	-3.44261600
C	-6.52398000	0.16486400	-1.47276000
H	-7.36445300	0.40554500	-2.13482900
H	-6.76246300	0.52851800	-0.46711300
H	-6.44309000	-0.92746100	-1.42264900
C	-1.51996900	2.58862300	0.95443700
H	-0.46693100	2.32305400	1.10152800
C	-2.11904100	2.86782300	2.34882500
H	-3.17602000	3.14594700	2.25566100
H	-1.59063800	3.69649700	2.83659900
H	-2.06355800	1.98783100	2.99275700
C	-1.56700500	3.87101400	0.10523400
H	-1.28195000	3.67934200	-0.93283200
H	-0.88377100	4.62073800	0.52094100
H	-2.56919100	4.31503800	0.10361500
C	-0.02308795	-0.34621258	-1.87964683
F	2.07133900	0.55334700	-2.44910700
F	-0.05242853	1.23790485	-1.57240720
O	-0.91792048	-0.66102667	-2.53313015

TS4

P	-1.67208600	0.13563700	-0.55262200
O	-2.60665800	-2.80632500	-0.87265500
O	2.89854600	-2.68847100	-1.26308000
C	-2.61714100	-0.34102700	1.04411500
H	-2.60165700	0.60252200	1.59425500
C	-1.94817400	-1.38624200	1.94294500
H	-1.96966800	-2.37029100	1.46274000
H	-0.90589200	-1.11247500	2.10304100
C	-2.67433500	-1.46887000	3.29633200
H	-2.19590300	-2.23098300	3.92754300
H	-2.56010400	-0.50872100	3.81938900
C	-4.16543300	-1.78953000	3.11019900
H	-4.68271800	-1.79980200	4.08069100
H	-4.26504600	-2.80059200	2.68460200
C	-4.83499400	-0.78335500	2.16070100
H	-4.83405300	0.21237700	2.63004600
H	-5.88814700	-1.05610100	1.99619300
C	-4.10203000	-0.70482100	0.80703100
H	-4.16401800	-1.67179000	0.30175200
H	-4.60073100	0.03017600	0.16702200
C	-2.60778200	1.81186200	-0.80912700
H	-3.66705000	1.51612600	-0.77786400
C	-2.39618800	2.91608800	0.24339400
H	-1.34526400	3.22407800	0.25412200
H	-2.60626300	2.55018100	1.25371300
C	-3.30677000	4.12779300	-0.03141300
H	-4.36280700	3.82612900	0.07502700
H	-3.12136100	4.90257000	0.72569600
C	-3.09286100	4.69932700	-1.43992800
H	-2.07045800	5.10026700	-1.50954800
H	-3.78016900	5.53795200	-1.62801100
C	-3.27169100	3.60344900	-2.49989800
H	-3.06551900	4.00294100	-3.50430000
H	-4.32205000	3.26865100	-2.50278500
C	-2.35278000	2.40304000	-2.21560300
H	-2.49046800	1.63949300	-2.98863700
H	-1.31023600	2.73600400	-2.27586200
C	-0.54984200	-1.52448400	-0.73481900
C	-1.23101800	-2.72679800	-1.04967300
C	-0.57059700	-3.88167200	-1.45734600
H	-1.14359100	-4.77928900	-1.67236800
C	0.81565900	-3.89523500	-1.57720600
H	1.31857600	-4.79487500	-1.91388800
C	1.52218700	-2.75291200	-1.21033300

C	0.87114900	-1.58376500	-0.73924100
C	-3.38031800	-3.15571300	-2.01357800
H	-3.25683300	-4.21495400	-2.28262400
H	-3.12010400	-2.52353600	-2.86986600
H	-4.42551100	-2.98093800	-1.74044400
C	3.61504000	-3.80747800	-1.74163600
H	3.46746800	-4.69094300	-1.10183600
H	4.66824600	-3.51733600	-1.71524100
H	3.33349300	-4.05990000	-2.77527100
C	1.80801900	-0.54081600	-0.18016300
C	2.19800200	-0.64625400	1.17352500
C	3.06300400	0.31950000	1.70199800
H	3.31432700	0.28513400	2.75922200
C	3.57844100	1.35652600	0.92862400
C	3.25956900	1.37766600	-0.43109600
H	3.67192300	2.16570400	-1.05614700
C	2.38805300	0.44984500	-1.00114000
C	1.77832500	-1.82449900	2.05492900
H	0.88624600	-2.27938800	1.61342900
C	2.88414600	-2.90376900	2.06158400
H	3.13235800	-3.22761300	1.04808300
H	2.56544200	-3.77904900	2.64558600
H	3.79924200	-2.50348900	2.51842100
C	1.43414200	-1.44276400	3.50718800
H	2.33455400	-1.16777000	4.07189600
H	0.98237500	-2.30441900	4.01740900
H	0.74030700	-0.60150800	3.55882900
C	4.42129600	2.45452100	1.55669600
H	4.47215400	2.24629700	2.63545400
C	5.86409500	2.45923300	1.01563800
H	6.35664300	1.49291800	1.18409200
H	6.46131000	3.24296900	1.50274000
H	5.87364900	2.65423300	-0.06464500
C	3.75176700	3.83185500	1.38044600
H	3.78372200	4.14504200	0.32811600
H	4.27873200	4.59418900	1.97271800
H	2.69742500	3.78803100	1.67457100
C	2.11041200	0.51581600	-2.50037400
H	1.23919500	-0.11407200	-2.70441700
C	3.29960800	-0.05399900	-3.30163200
H	4.20026300	0.55250700	-3.13566500
H	3.08027700	-0.04763500	-4.37915700
H	3.52206100	-1.07967000	-2.99309400
C	1.77493100	1.93728600	-2.99007500

H	1.01326700	2.39887200	-2.35813800
H	1.40348500	1.90274600	-4.02348700
H	2.66116500	2.58518000	-2.98284900
C	-0.22423800	1.45496300	1.86983800
F	0.61131500	2.88740000	1.37217600
F	-0.31933800	0.90789900	-1.09040200
O	-0.29190300	1.45817500	3.04826400
F	-2.52428600	-0.43489300	-1.86472800

TS4'

P	1.98819400	0.49186600	-0.77716700
O	3.42212800	-0.74838600	1.66774300
O	-1.61754500	0.52520900	3.39869300
C	2.29941500	-1.29106200	-1.36903600
H	1.94476200	-1.25600800	-2.40163900
C	1.55791100	-2.40487300	-0.64005200
H	1.89885600	-2.46612900	0.39999700
H	0.48977800	-2.18595200	-0.63141900
C	1.81572400	-3.75357600	-1.33960400
H	1.29351800	-4.55743600	-0.80633800
H	1.38942200	-3.71522700	-2.35311100
C	3.31559700	-4.07038400	-1.42311200
H	3.47903900	-5.01633600	-1.95996000
H	3.70890900	-4.20983500	-0.40358500
C	4.08100200	-2.92583400	-2.10054600
H	3.76810000	-2.85242400	-3.15326300
H	5.16096600	-3.13561500	-2.10491200
C	3.82021600	-1.58020000	-1.40035200
H	4.19853600	-1.62740700	-0.37389400
H	4.35037800	-0.77932100	-1.91911500
C	0.99802700	2.12234600	-0.94284100
H	-0.03787100	1.84188900	-0.76153900
C	1.51501800	3.08754400	0.15649600
H	2.59079500	3.24633200	0.01370800
H	1.38820800	2.65157100	1.15339800
C	0.82074900	4.46133700	0.09987700
H	-0.23727700	4.35693500	0.36271300
H	1.26992200	5.11360400	0.86411900
C	0.93698700	5.10227200	-1.28901200
H	1.99764700	5.32271600	-1.49285700
H	0.40307400	6.06425900	-1.30871400
C	0.39855100	4.15785500	-2.37215600
H	0.55943500	4.59695000	-3.36753100
H	-0.68062400	4.03208300	-2.26245000

C	1.09352600	2.78690500	-2.33128200
H	0.63808300	2.12790300	-3.07220900
H	2.14597900	2.93312500	-2.59005600
C	1.22497800	0.05692200	1.09842300
C	2.22110800	-0.19192700	2.07705400
C	2.00803100	-0.00864600	3.44099900
H	2.81717700	-0.16996400	4.14738000
C	0.74409800	0.33997500	3.90439100
H	0.58310400	0.48981100	4.96576700
C	-0.30877700	0.37441600	2.99145300
C	-0.11173800	0.18455500	1.60006800
C	4.62639600	-0.17155300	2.15265000
H	4.83552200	-0.47440400	3.18939200
H	4.59096500	0.92195300	2.08583600
H	5.42690100	-0.54478800	1.50728600
C	-1.89357300	0.69689900	4.77352900
H	-1.58043900	-0.17713800	5.36449000
H	-2.97837300	0.80888100	4.84501900
H	-1.40994600	1.59854500	5.17825500
C	-1.37658100	-0.14154000	0.82407100
C	-1.72156200	-1.52187700	0.72264200
C	-2.82905600	-1.90032400	-0.04404800
H	-3.04895800	-2.95597900	-0.15960700
C	-3.65018900	-0.96267400	-0.67099200
C	-3.39210000	0.37936600	-0.41500700
H	-4.04401800	1.12052600	-0.86539400
C	-2.29620200	0.81447800	0.33626900
C	-1.01915600	-2.61210200	1.54027100
H	0.01749800	-2.31245100	1.71042600
C	-1.69219500	-2.75457600	2.92443200
H	-1.69156400	-1.81211200	3.47265300
H	-1.17281300	-3.51347400	3.52666700
H	-2.73722600	-3.06906800	2.80237000
C	-1.00814100	-3.99985400	0.86749300
H	-1.98200400	-4.49760500	0.96143800
H	-0.26605200	-4.64505300	1.35460100
H	-0.76806000	-3.94299600	-0.19622600
C	-4.74110600	-1.32962700	-1.66419900
H	-5.52035900	-0.55583000	-1.58522400
C	-4.16746800	-1.26718800	-3.09699300
H	-3.65064100	-0.31789100	-3.27718800
H	-4.96898700	-1.40037800	-3.83857400
H	-3.42193900	-2.05802100	-3.24105000
C	-5.41121600	-2.68585800	-1.39584900

H	-6.25306400	-2.83557600	-2.08451800
H	-5.78940200	-2.75470200	-0.36760900
H	-4.71094300	-3.51568200	-1.55499800
C	-2.20614600	2.31264400	0.61433200
H	-1.18428900	2.53693500	0.93824000
C	-3.15278700	2.72926700	1.76250400
H	-4.19264600	2.50030700	1.49409300
H	-3.07957900	3.81318200	1.93145200
H	-2.91652400	2.21004900	2.69156000
C	-2.54649100	3.15414100	-0.63308600
H	-2.11308100	2.71734600	-1.53954300
H	-2.20709400	4.18857500	-0.50043000
H	-3.63400000	3.19939100	-0.77896700
C	-0.47245500	-0.30597900	-2.38718800
F	2.80027200	0.87358900	-2.29362100
F	-1.59032500	1.21123800	-2.91640000
O	-0.86970100	-1.23730800	-2.95585500
F	3.36192800	1.02408600	-0.06394400

TS5

C	3.02801100	-0.33884100	-1.21457400
C	1.65197100	-0.57871800	-1.20061400
C	0.91211300	-0.70204700	-0.00028300
C	1.65154000	-0.57783200	1.20021700
C	3.02759700	-0.33797900	1.21450600
C	3.69229300	-0.21987900	0.00004700
H	3.58650900	-0.23853800	-2.14047100
H	1.13926400	-0.66014300	-2.15634600
H	1.13847200	-0.65843100	2.15582400
H	3.58575600	-0.23704900	2.14053900
B	0.14832200	1.83742300	0.00004400
F	1.38637600	2.27465500	-0.00172400
F	-0.57085200	1.82949700	-1.13626700
F	5.02297900	0.02390900	0.00018400
F	-0.56775800	1.82924800	1.13811900
Ag	-1.09842000	-1.19181500	0.00011700
F	-3.16334000	-1.21110100	0.00021000
K	-3.22703600	1.18096100	-0.00030100

TS5'

C	2.48718100	1.33317600	1.18870300
C	1.37768200	0.48752600	1.16824500
C	0.82927800	-0.02233900	-0.03456400
C	1.46078500	0.39115000	-1.23357300
C	2.57195700	1.23448700	-1.24361800

C	3.06315500	1.69288900	-0.02528300
H	2.92083100	1.70050200	2.11404400
H	0.95461100	0.16461300	2.11658300
H	1.09873900	0.00329800	-2.18305700
H	3.06873000	1.52939700	-2.16304800
B	1.02800500	-2.20781700	0.11838400
F	2.37128200	-2.23348000	0.11694500
F	0.41464700	-2.46047800	1.30005300
F	4.14157000	2.50692500	-0.02058700
F	0.41170300	-2.65496300	-1.00306400
Ag	-1.25001800	-0.38958700	-0.09603300
F	-3.30444300	-0.20483700	-0.12376000
K	-3.23606000	2.14063300	0.14165100

TS5''

C	-2.23915600	-1.18167000	1.21684600
C	-0.92147500	-0.70272300	1.20092700
C	-0.22000000	-0.44323000	0.00032100
C	-0.92100000	-0.70340300	-1.20041800
C	-2.23865800	-1.18240500	-1.21660600
C	-2.87704000	-1.41946100	0.00006200
H	-2.76135900	-1.39530100	2.14517700
H	-0.43259500	-0.51437300	2.15267200
H	-0.43172100	-0.51558200	-2.15206300
H	-2.76043700	-1.39659500	-2.14504600
B	0.77754800	1.66967500	0.00005900
F	0.08322900	2.01092700	-1.14660100
F	0.08233900	2.01141100	1.14607400
F	-4.15174600	-1.86861000	-0.00005100
F	2.07091200	2.03014900	0.00060900
Ag	1.84948000	-0.69034800	0.00027400
F	3.70511500	-1.36795100	-0.00105600
K	-2.31746600	1.91441300	-0.00060400

TS6

P	-0.57020400	1.39743800	0.23639700
O	0.09468300	4.24456600	1.09375600
O	4.46905400	0.87142900	0.96214500
C	-1.36360000	2.29806100	-1.21229100
H	-2.07337000	1.54243600	-1.57340300
C	-0.33385900	2.51157400	-2.33609700
H	0.43053200	3.22421500	-1.99213900
H	0.15021000	1.56364200	-2.56651100
C	-1.01171200	3.02200100	-3.61548100

H	-0.24425500	3.22183300	-4.37415600
H	-1.63021800	2.20473800	-4.00367400
C	-1.85345700	4.27852800	-3.36005600
H	-2.37543100	4.58247700	-4.27620000
H	-1.19308600	5.11426500	-3.08053100
C	-2.86323300	4.04496700	-2.22708200
H	-3.58630900	3.27666700	-2.53773600
H	-3.43818100	4.95853600	-2.02640200
C	-2.16011300	3.58746000	-0.93622000
H	-1.48073100	4.37249600	-0.59272200
H	-2.90457800	3.43583500	-0.14679100
C	-1.65144500	1.77982900	1.71073200
H	-1.78368000	2.86653000	1.67521100
C	-3.02026500	1.09472700	1.57533300
H	-2.87648500	0.01002700	1.56837300
H	-3.48972100	1.34917600	0.61812100
C	-3.95184900	1.47720000	2.73482900
H	-4.17540900	2.55352400	2.68575600
H	-4.90665900	0.94833500	2.62511200
C	-3.31125900	1.15031700	4.09103000
H	-3.19116900	0.06038400	4.17679600
H	-3.96939300	1.46195500	4.91159100
C	-1.93604100	1.81983500	4.22432000
H	-1.46686900	1.54845000	5.17846900
H	-2.06191200	2.91289900	4.23272500
C	-1.00787600	1.42873000	3.06261300
H	-0.04030000	1.93228300	3.17023400
H	-0.82251800	0.34990400	3.10355400
C	1.06660800	2.13907400	0.65045300
C	1.22921500	3.48398700	1.05721300
C	2.48810800	3.96168600	1.40972300
H	2.62848100	4.99050300	1.71843400
C	3.59512000	3.10859200	1.37906600
H	4.56437800	3.50105700	1.66256100
C	3.44587200	1.77819900	0.99858100
C	2.17410100	1.28218500	0.62221400
C	0.19092400	5.60888300	1.47401500
H	0.84739500	6.16923300	0.79566700
H	0.55573100	5.71302600	2.50434900
H	-0.82308200	6.00647300	1.40573200
C	5.77777800	1.30770400	1.29471800
H	6.12427100	2.09483000	0.61149700
H	6.41703400	0.43002700	1.18921700
H	5.82976400	1.67363100	2.32913700

C	2.12641300	-0.17448300	0.21346400
C	2.63453900	-0.51418300	-1.09652100
C	3.07900000	-1.80767700	-1.32696200
H	3.42263900	-2.07810000	-2.32080300
C	3.07684900	-2.79040100	-0.33007500
C	2.64641300	-2.44538500	0.95399100
H	2.65193100	-3.20114300	1.73280400
C	2.17928500	-1.17266000	1.25652000
C	2.74637900	0.52388800	-2.20361900
H	2.22947900	1.42795700	-1.87098800
C	4.22437000	0.90065400	-2.43724000
H	4.69648400	1.25806300	-1.51752200
H	4.29764800	1.68666300	-3.19791300
H	4.79323400	0.03306000	-2.79193200
C	2.08719500	0.06079600	-3.51693700
H	2.60952200	-0.80452600	-3.94126800
H	2.14092300	0.86906600	-4.25633400
H	1.03285000	-0.20632800	-3.39310600
C	3.50625400	-4.20953300	-0.64593300
H	3.82450800	-4.23160700	-1.69663100
C	4.70209500	-4.65474100	0.21657700
H	5.55831200	-3.98236000	0.09011300
H	5.01657100	-5.66721300	-0.06194900
H	4.44026800	-4.67011100	1.28119200
C	2.31376600	-5.17596700	-0.49632600
H	1.99073700	-5.23378500	0.55018000
H	2.59855000	-6.18433800	-0.81954600
H	1.46105100	-4.82669400	-1.08387500
C	1.79484500	-0.84668500	2.69159900
H	1.37431800	0.16093100	2.70369300
C	3.03798300	-0.83714600	3.60264600
H	3.49418600	-1.83275900	3.65216200
H	2.76267200	-0.53973800	4.62156900
H	3.79297400	-0.14103600	3.22552700
C	0.72086600	-1.81180300	3.22752400
H	-0.14926900	-1.84621900	2.56196800
H	0.38964800	-1.49710600	4.22442500
H	1.10687800	-2.83360100	3.31435500
Pd	-0.08116000	-0.77077500	-0.28434600
F	0.36947900	-2.62171500	-0.83758100
C	-2.72712300	-1.99924300	-0.43873100
C	-4.00202500	-1.51779900	-0.07670100
C	-4.83391500	-2.22727100	0.79396400
C	-4.36905400	-3.42424800	1.32758500

C	-3.11795100	-3.93490200	1.00306800
C	-2.30210100	-3.21988700	0.12040700
H	-4.36977200	-0.57622700	-0.47550800
H	-5.82299100	-1.86788400	1.06146700
H	-2.79561600	-4.87603200	1.43865300
H	-1.32000000	-3.60101400	-0.13582600
F	-1.24610200	-0.14204500	-3.74185500
F	-5.15851600	-4.10725200	2.18807200
Ag	-1.92153200	-1.12151600	-2.12539600

TS7

P	1.12663400	0.94013100	-0.09606800
O	1.57934800	3.78768300	-1.08200400
O	-3.75388900	2.36955400	-0.88888600
C	2.02390000	1.54487800	1.44387400
H	2.79943500	0.77890200	1.57050600
C	1.09417100	1.45289700	2.67249300
H	0.33054800	2.23751100	2.58958000
H	0.58457900	0.49059300	2.70421700
C	1.88253400	1.65156000	3.97481300
H	1.19184200	1.59926500	4.82584500
H	2.58002900	0.81317500	4.08416200
C	2.64150700	2.98453600	3.98032200
H	3.25235200	3.07713300	4.88714400
H	1.91893300	3.81465900	3.99970300
C	3.51960500	3.12170000	2.72876700
H	4.32073600	2.36864600	2.75991200
H	4.01075900	4.10355300	2.70869200
C	2.69970500	2.93147400	1.44010700
H	1.92316500	3.70464600	1.38868100
H	3.33592400	3.07609500	0.56541400
C	2.31383600	0.91514000	-1.57336900
H	2.12454900	1.86543800	-2.08072100
C	3.81914100	0.88470100	-1.23968100
H	4.06623200	-0.02138200	-0.68349400
H	4.09275700	1.73212600	-0.60652900
C	4.66164500	0.91680100	-2.52713200
H	4.50179600	1.87728300	-3.04014400
H	5.72673100	0.87314000	-2.26625800
C	4.29114700	-0.22999400	-3.47761600
H	4.52494600	-1.19117800	-2.99780400
H	4.89330500	-0.17651700	-4.39323800
C	2.79509200	-0.18733900	-3.81392600
H	2.52109100	-1.02807700	-4.46357900

H	2.57615700	0.73287000	-4.37670100
C	1.93442500	-0.22364500	-2.54218400
H	0.88071500	-0.14244400	-2.81369300
H	2.05558000	-1.19104800	-2.04485200
C	-0.12333400	2.21793000	-0.57972100
C	0.24063600	3.52454900	-0.99455200
C	-0.73865100	4.45856800	-1.31891100
H	-0.47011000	5.46256100	-1.62490200
C	-2.08878000	4.10443500	-1.27245200
H	-2.83363800	4.84598000	-1.53551500
C	-2.46002400	2.81202500	-0.91505100
C	-1.47769300	1.85474100	-0.55917700
C	2.00986200	5.12211000	-1.29717100
H	1.61053100	5.79914400	-0.53074600
H	1.71953900	5.48481100	-2.29188900
H	3.09869100	5.09692800	-1.22644300
C	-4.79064900	3.27453800	-1.23143000
H	-4.82199200	4.13035000	-0.54334900
H	-5.71839500	2.70715900	-1.14415800
H	-4.68250900	3.63953300	-2.26197000
C	-2.00501300	0.47720600	-0.22814800
C	-2.63956800	0.27699200	1.04830100
C	-3.54407100	-0.76669600	1.19719000
H	-3.98602900	-0.94863200	2.17194600
C	-3.88866700	-1.60885400	0.13862700
C	-3.32618400	-1.37057600	-1.12030200
H	-3.59643200	-2.02002400	-1.94625900
C	-2.40312900	-0.35849300	-1.33476600
C	-2.38667900	1.20992300	2.22158800
H	-1.51923300	1.82378100	1.97159400
C	-3.57876900	2.16453100	2.43736300
H	-3.77709800	2.76872000	1.54863900
H	-3.37446300	2.83624000	3.27968100
H	-4.48843800	1.59805500	2.66898100
C	-2.07186700	0.44222300	3.51998800
H	-2.97675800	-0.01526800	3.93733900
H	-1.67906400	1.13374100	4.27482100
H	-1.34251200	-0.35433100	3.35767900
C	-4.82211100	-2.78211900	0.36202400
H	-5.18813100	-2.71929200	1.39557400
C	-6.04574900	-2.74252100	-0.57141300
H	-6.60709800	-1.80814900	-0.45655800
H	-6.71987500	-3.57756100	-0.34845200
H	-5.74846100	-2.83011600	-1.62302700

C	-4.04729800	-4.10827200	0.21887500
H	-3.70174600	-4.24284200	-0.81309200
H	-4.69400500	-4.95588700	0.47613600
H	-3.16275800	-4.10797500	0.86040100
C	-1.87083500	-0.13163700	-2.74267700
H	-1.04312600	0.58112300	-2.67723500
C	-2.94914700	0.49926700	-3.64537200
H	-3.80889400	-0.17339400	-3.74629000
H	-2.54615700	0.68847200	-4.64759900
H	-3.31039800	1.44423400	-3.23017000
C	-1.32880700	-1.43320400	-3.36472100
H	-0.63345700	-1.93576900	-2.68517200
H	-0.80893200	-1.21698100	-4.30571200
H	-2.13890100	-2.13519000	-3.59153900
Pd	-0.11457300	-0.95650800	0.29739500
F	-1.32440200	-2.51297500	0.54833300
C	1.55886300	-2.36215400	0.63447500
C	2.91151000	-1.99308000	0.38841700
C	3.76569000	-2.73038100	-0.42018000
C	3.25641700	-3.86121000	-1.05478300
C	1.94422300	-4.28559800	-0.87201700
C	1.11691200	-3.54437700	-0.03475400
H	3.31957000	-1.14443300	0.92088900
H	4.80552200	-2.45697900	-0.56878400
H	1.59812500	-5.18318100	-1.37488500
H	0.09521500	-3.86474500	0.12791500
B	1.38822700	-2.32400600	2.52958200
F	0.09292600	-2.10753100	2.94562200
F	1.86883000	-3.55896100	2.86970300
F	2.22746700	-1.28004900	2.91237000
F	4.06657500	-4.56497100	-1.86591000

TS7'

P	1.62035400	-0.88347300	0.01991700
F	-6.55354400	-3.20685500	-0.77449600
O	4.54427600	-0.25931200	-0.17593400
O	1.45701600	4.27620000	-0.76038900
C	-2.75909400	-1.75366900	0.01892300
C	-3.06873600	-3.11586500	0.21069100
H	-2.30493600	-3.79798900	0.57614300
C	-4.34582000	-3.61172900	-0.04492200
H	-4.60144800	-4.65483100	0.11201200
C	-5.31457700	-2.73400900	-0.52524900
C	-5.04907100	-1.38829200	-0.75489900

H	-5.83482200	-0.74211800	-1.13359900
C	-3.76391900	-0.91433600	-0.49352600
H	-3.53514200	0.12851800	-0.67278800
C	2.15794100	-1.57524700	1.69388700
H	1.38231100	-2.33794500	1.86521100
C	2.00038600	-0.50772100	2.79126500
H	2.76227600	0.27039900	2.64178800
H	1.02837500	-0.01981700	2.70663300
C	2.15479000	-1.11818600	4.19260900
H	2.07447200	-0.32940900	4.95152400
H	1.32119100	-1.81338900	4.37152100
C	3.48491100	-1.87196800	4.33543600
H	3.55257700	-2.34547100	5.32296500
H	4.31438500	-1.15164200	4.27210900
C	3.64837500	-2.91968800	3.22411200
H	2.87966600	-3.69820000	3.34100100
H	4.61992000	-3.42258300	3.31409100
C	3.52183900	-2.28013200	1.83013800
H	4.32233200	-1.54865400	1.69213100
H	3.65118100	-3.04343500	1.05580600
C	2.40620700	-2.00451300	-1.25465200
H	3.47240600	-2.06059700	-1.02065100
C	1.80947300	-3.42787900	-1.16705100
H	0.73450200	-3.37663700	-1.35942200
H	1.94860400	-3.84531800	-0.16121600
C	2.46898300	-4.34720500	-2.20827800
H	3.54053900	-4.45777900	-1.97957200
H	2.02734000	-5.34998700	-2.14646400
C	2.30774200	-3.77782800	-3.62535600
H	1.23835400	-3.74320400	-3.87627200
H	2.79016400	-4.43586000	-4.35971100
C	2.88905400	-2.35939800	-3.71550500
H	2.74020100	-1.94759400	-4.72196000
H	3.97678000	-2.40325700	-3.54887000
C	2.25247600	-1.42437500	-2.67498000
H	2.72344100	-0.43484600	-2.73037500
H	1.18687800	-1.30075800	-2.89086600
C	2.41494300	0.78771900	-0.20697600
C	3.82674100	0.89435500	-0.33154700
C	4.43776300	2.11503100	-0.59749200
H	5.51398300	2.18923800	-0.69370200
C	3.66494200	3.26600200	-0.74402100
H	4.15859600	4.20642700	-0.95723700
C	2.28294100	3.19192700	-0.61820600

C	1.63667800	1.95900500	-0.34075000
C	5.93473400	-0.25899500	-0.45260500
H	6.48875800	0.37866900	0.24921500
H	6.13791000	0.06873100	-1.48061800
H	6.25940600	-1.29395600	-0.32947800
C	2.03259500	5.53159200	-1.07993600
H	2.71861000	5.87500500	-0.29319500
H	1.19600700	6.22798700	-1.15728500
H	2.56695400	5.49789700	-2.03931100
C	0.13613500	2.03650500	-0.23742400
C	-0.47116300	2.39931900	0.99129100
C	-1.85160400	2.60223000	1.02188300
H	-2.32456300	2.84578800	1.96805000
C	-2.65048400	2.52331700	-0.12404300
C	-2.02430700	2.19281500	-1.32591900
H	-2.61186600	2.12659000	-2.23382900
C	-0.64866600	1.93777200	-1.40867200
C	0.34473900	2.68236500	2.25279400
H	1.31628600	2.19070800	2.14467300
C	0.60635900	4.19752200	2.39528600
H	1.10988200	4.60018600	1.51536800
H	1.22252500	4.39918200	3.28048400
H	-0.34295300	4.73457000	2.51213600
C	-0.30453800	2.16498000	3.55046000
H	-1.18170400	2.76178700	3.82611800
H	0.40919300	2.23979400	4.37930400
H	-0.62580300	1.12552600	3.46656000
C	-4.14732600	2.80281600	-0.00520900
H	-4.53285200	2.11190500	0.75913900
C	-4.39613100	4.24230900	0.49275900
H	-3.89129500	4.44063300	1.44347400
H	-5.46831900	4.42233100	0.63604300
H	-4.02356700	4.96792100	-0.24069100
C	-4.94586400	2.56692200	-1.29545200
H	-4.64075500	3.26675400	-2.08318400
H	-6.01346200	2.72642700	-1.10809900
H	-4.82452600	1.55151000	-1.68640000
C	-0.03097300	1.65198600	-2.77626400
H	0.94847600	1.19312200	-2.61066200
C	0.19502400	2.96802200	-3.54941500
H	-0.76516200	3.45918600	-3.75092800
H	0.67944800	2.76614500	-4.51267500
H	0.81446500	3.66714300	-2.98331200
C	-0.85412300	0.67193300	-3.63072500

H	-1.05962000	-0.25370300	-3.08896600
H	-0.29381100	0.42419500	-4.54113700
H	-1.80428500	1.11604800	-3.95237800
Pd	-0.79167000	-1.17991000	-0.00904700
F	-0.76302300	-1.89388300	-1.83830000
B	-2.59602300	-0.90050600	2.23817500
F	-1.24145000	-0.65212400	2.11889500
F	-3.39983700	0.14982800	2.17757700
F	-2.93130500	-1.98012200	2.92562600

TS8

P	1.46513400	0.52537000	-0.13389300
F	-3.04427200	5.38481700	1.51169200
O	3.87094900	-0.29494600	1.33411300
O	-0.08718700	-4.10824500	1.77558700
C	-1.96066600	2.30171800	-0.98976600
C	-1.61395600	3.62882600	-1.30767900
H	-1.04337900	3.83460100	-2.20882500
C	-1.98102400	4.67735500	-0.46930600
H	-1.71706400	5.70741000	-0.68452200
C	-2.70267300	4.37947200	0.68523600
C	-3.08965300	3.08212600	1.01121400
H	-3.65613300	2.90456700	1.91938900
C	-2.72919100	2.04323400	0.15488700
H	-3.02061200	1.02462100	0.37997100
C	2.88159900	0.68126800	-1.39891500
H	2.33813400	1.06959600	-2.26661800
C	3.43599100	-0.68727700	-1.84216200
H	3.97605900	-1.15882300	-1.01259100
H	2.60358100	-1.33481500	-2.12506300
C	4.38808800	-0.52342600	-3.03777300
H	4.79958800	-1.50174100	-3.31837100
H	3.81317800	-0.16297800	-3.90232300
C	5.51987800	0.46516700	-2.72718900
H	6.17354500	0.58972900	-3.60012000
H	6.14652800	0.05724900	-1.91884300
C	4.95401800	1.82254700	-2.28717400
H	4.39925200	2.26919000	-3.12540800
H	5.76667000	2.51844900	-2.03999300
C	4.01084900	1.67650700	-1.07906000
H	4.58448400	1.33071300	-0.21437800
H	3.60351000	2.66157400	-0.82195500
C	1.78883800	1.85429800	1.16565400
H	2.87309100	1.97962500	1.23621200

C	1.15058600	3.17851700	0.70651600
H	0.06832500	3.03485600	0.63544400
H	1.49150100	3.45344900	-0.29998400
C	1.43070200	4.31603500	1.69895500
H	2.51216900	4.51356300	1.74020800
H	0.95140300	5.23906900	1.34819800
C	0.92213500	3.94812400	3.10005400
H	-0.17349500	3.85083700	3.06575300
H	1.14688100	4.74921400	3.81543000
C	1.53138500	2.62006900	3.57153200
H	1.12939600	2.34177600	4.55437900
H	2.61722200	2.74479500	3.69869600
C	1.27065300	1.48599800	2.56653600
H	1.73762400	0.56199000	2.92096900
H	0.19389500	1.29853000	2.51393200
C	1.70387800	-1.07614500	0.79014700
C	2.94857300	-1.30226400	1.43584500
C	3.19417500	-2.47077800	2.14682100
H	4.14953800	-2.64372900	2.62670600
C	2.19469800	-3.43706500	2.26200900
H	2.39892800	-4.33910400	2.82597900
C	0.95461200	-3.22326300	1.67341200
C	0.68845600	-2.04795200	0.92218100
C	5.15231100	-0.47382900	1.91278500
H	5.67343000	-1.33684900	1.47749700
H	5.08935600	-0.59713800	3.00222000
H	5.71126700	0.43567200	1.68497900
C	0.13696400	-5.34928100	2.42150800
H	0.93228900	-5.92320100	1.92636200
H	-0.80414700	-5.89646600	2.34433800
H	0.39167700	-5.21510000	3.48211800
C	-0.71414300	-1.92443600	0.38615400
C	-1.04464100	-2.40817200	-0.90535200
C	-2.37849800	-2.31994200	-1.32694100
H	-2.63693100	-2.64982100	-2.32752900
C	-3.39620300	-1.83323000	-0.50540100
C	-3.05471700	-1.43340300	0.78806900
H	-3.84114100	-1.10390100	1.46239200
C	-1.73596900	-1.46355800	1.25257200
C	-0.01116300	-3.08224900	-1.80966700
H	0.93585700	-2.55342800	-1.67040900
C	0.19147300	-4.56109900	-1.41071800
H	0.55113700	-4.67685700	-0.38913800
H	0.91595000	-5.03133300	-2.08681800

H	-0.75501400	-5.10952900	-1.49575400
C	-0.35064300	-3.02671200	-3.30842500
H	-1.19497300	-3.68351100	-3.55467700
H	0.51208500	-3.38469000	-3.88224000
H	-0.56191400	-2.00647200	-3.62648400
C	-4.83849800	-1.78767700	-0.98871500
H	-4.83872400	-2.08423800	-2.04614800
C	-5.70906100	-2.79806000	-0.21739000
H	-5.30574600	-3.81299600	-0.30453300
H	-6.73665300	-2.80013500	-0.60066300
H	-5.74893400	-2.54529400	0.84927500
C	-5.43715500	-0.37154200	-0.90691400
H	-5.47507400	-0.01486500	0.13003700
H	-6.46276700	-0.36525700	-1.29478600
H	-4.84836100	0.34198900	-1.49128200
C	-1.47445100	-1.09417900	2.71401700
H	-0.39401600	-0.99362400	2.85524100
C	-1.96700800	-2.21775100	3.64989800
H	-3.05495200	-2.33266100	3.56942500
H	-1.72862300	-1.98150000	4.69457800
H	-1.50837400	-3.17295300	3.38912000
C	-2.11806400	0.23904800	3.13652800
H	-1.82019300	1.06100300	2.48237200
H	-1.81762700	0.49361100	4.15994900
H	-3.21320800	0.18199000	3.12406100
Pd	-0.50969500	0.77977200	-1.60838200
F	0.57117700	-0.31549000	-2.90059900
C	-2.12324000	1.23505100	-2.44145100
O	-3.06103100	1.21030600	-3.14305500

TS9

P	0.80204000	0.99899000	-0.05348100
O	0.76795800	3.94119800	-0.74253200
O	-4.27806100	1.69507400	-0.81371100
C	1.70644200	1.62646000	1.47239400
H	2.28882300	0.73147200	1.73267500
C	0.71815000	1.85501800	2.62842500
H	0.09315700	2.73201400	2.40257500
H	0.05912700	0.99184700	2.72016800
C	1.46169800	2.06605200	3.95527000
H	0.73953000	2.26559200	4.75737200
H	1.97159600	1.12837200	4.21609100
C	2.48616800	3.20513800	3.85823800
H	3.04351600	3.30184700	4.79873400

H	1.95541500	4.15733000	3.70598100
C	3.45183600	2.98077200	2.68495300
H	4.05557500	2.08142900	2.87870100
H	4.15541200	3.81955100	2.60144000
C	2.69046100	2.80481800	1.35885600
H	2.13834000	3.72149400	1.13148400
H	3.40323300	2.65065300	0.54129100
C	2.02241500	1.26883900	-1.44840900
H	2.36261500	2.30685000	-1.38055300
C	3.22724500	0.32532200	-1.26224800
H	2.87307800	-0.71140700	-1.26394000
H	3.70334900	0.48269300	-0.28925000
C	4.26354100	0.50509600	-2.38144200
H	4.69973300	1.51358300	-2.32021400
H	5.08506900	-0.20732500	-2.23323000
C	3.62656600	0.31030400	-3.76404700
H	3.28555000	-0.73172500	-3.85641600
H	4.36820800	0.47212400	-4.55651300
C	2.42565900	1.24935500	-3.95234100
H	1.95271300	1.07769400	-4.92803100
H	2.77665900	2.29231000	-3.95053600
C	1.38867600	1.06302100	-2.83316600
H	0.55213700	1.75753600	-2.97729200
H	0.98021400	0.04774400	-2.88962900
C	-0.66097600	2.08520300	-0.42896100
C	-0.51086200	3.45531100	-0.75469900
C	-1.61476900	4.24005200	-1.07580700
H	-1.50255500	5.28924800	-1.32093500
C	-2.89132200	3.67584200	-1.09268600
H	-3.73669400	4.30273100	-1.34982200
C	-3.06233300	2.32803200	-0.79254600
C	-1.95328300	1.51494300	-0.45145700
C	0.99580000	5.29718100	-1.08725500
H	0.48637500	5.98143100	-0.39553300
H	0.67103300	5.51088700	-2.11438000
H	2.07461300	5.44400700	-1.00962000
C	-5.43149200	2.45279900	-1.13905000
H	-5.59591100	3.26705900	-0.41997800
H	-6.26782800	1.75365000	-1.08930000
H	-5.36754000	2.87118600	-2.15303500
C	-2.28178900	0.06769700	-0.15956600
C	-2.76662400	-0.30515800	1.12557800
C	-3.27126700	-1.59507700	1.31233300
H	-3.61872200	-1.88532400	2.29963000

C	-3.31633800	-2.54014300	0.28414700
C	-2.85353200	-2.15609500	-0.97472600
H	-2.88273800	-2.87340400	-1.78891400
C	-2.34109000	-0.87451500	-1.22461400
C	-2.74966700	0.66258900	2.30152100
H	-2.08326000	1.48975300	2.03909600
C	-4.15079700	1.25601000	2.54694100
H	-4.52928600	1.75863000	1.65284900
H	-4.12318300	1.97788200	3.37236800
H	-4.86151700	0.46396700	2.81309800
C	-2.20346100	0.01082200	3.58722200
H	-2.92519100	-0.69403800	4.01636400
H	-2.01213400	0.77999000	4.34505000
H	-1.27269000	-0.53570500	3.40307200
C	-3.78822200	-3.95955400	0.54892800
H	-4.13080000	-4.00155400	1.59188100
C	-4.97346100	-4.35687500	-0.34885900
H	-5.81471300	-3.66528900	-0.22628000
H	-5.32067000	-5.36774100	-0.10396100
H	-4.68753600	-4.35279500	-1.40763400
C	-2.61673100	-4.95125600	0.40155900
H	-2.26834200	-4.98495600	-0.63840600
H	-2.92899500	-5.96411200	0.68405900
H	-1.76838500	-4.65068700	1.02440500
C	-1.94811000	-0.50120600	-2.64928100
H	-1.35546300	0.41574100	-2.60274500
C	-3.20527300	-0.19933600	-3.48818200
H	-3.83049100	-1.09600800	-3.57878400
H	-2.92825300	0.12634200	-4.49837600
H	-3.80845800	0.58249300	-3.01813800
C	-1.08234500	-1.57688900	-3.32847900
H	-0.23533500	-1.85791500	-2.69242400
H	-0.69396500	-1.20327000	-4.28330800
H	-1.65675800	-2.48535600	-3.54320600
Pd	-0.01769000	-1.07639500	0.34683000
F	0.16708300	-3.06142000	1.31448400
C	1.31761900	-1.92655800	1.68280100
C	2.56681900	-2.36621200	0.98878200
C	2.56990800	-3.17878100	-0.15554500
C	3.77218500	-1.82523700	1.45568100
C	3.75509200	-3.41393500	-0.84636000
H	1.63797800	-3.60919100	-0.50258200
C	4.96398200	-2.04629600	0.76547200
H	3.76030700	-1.21876600	2.35529900

C	4.93040700	-2.82380900	-0.38540100
H	3.78361200	-4.03313300	-1.73694000
H	5.90573200	-1.62258200	1.09841400
O	1.25629600	-1.49234600	2.81695200
F	6.07255900	-3.01362200	-1.08156000

TS10

P	1.42720000	0.10925900	-0.47930100
O	2.96060300	-2.05858800	1.07340200
O	-2.51534800	-2.53095700	1.61098100
C	1.72735500	1.43336500	0.94623700
H	0.69501300	1.68423300	1.20985000
C	2.39175400	2.75830600	0.52375700
H	3.45492600	2.59693600	0.31459800
H	1.93945900	3.12619400	-0.39627900
C	2.27070400	3.80146800	1.64875600
H	2.76290300	4.73385000	1.34294200
H	1.20909000	4.04335900	1.80526100
C	2.87102400	3.28658400	2.96328900
H	2.74201000	4.02598600	3.76455100
H	3.95488100	3.14739000	2.83332700
C	2.24037900	1.94698300	3.36754400
H	1.17579900	2.10128300	3.59722100
H	2.70698700	1.56268600	4.28455400
C	2.36529800	0.89468800	2.24764400
H	3.42417600	0.66837500	2.09252600
H	1.88293600	-0.03172000	2.57150700
C	3.14684000	-0.25364700	-1.21108200
H	3.09454400	-1.34426500	-1.30426800
C	3.31272400	0.32168300	-2.63849800
H	3.41192200	1.41087100	-2.58114100
H	2.42443500	0.11948800	-3.23518700
C	4.55608200	-0.28297900	-3.31258700
H	4.40548800	-1.36552000	-3.44279400
H	4.67013500	0.14039700	-4.31902400
C	5.82930400	-0.04659400	-2.48927400
H	6.04736600	1.03143400	-2.46611200
H	6.69303100	-0.53374300	-2.96065300
C	5.64779700	-0.54592400	-1.05031700
H	6.53878200	-0.31914400	-0.44942300
H	5.53899600	-1.64152600	-1.05399200
C	4.40569000	0.08053100	-0.39038900
H	4.32240900	-0.27863600	0.63344200
H	4.54809200	1.16511700	-0.34879200

C	0.77640500	-1.29296300	0.60677500
C	1.61572400	-2.23171000	1.24793100
C	1.08082400	-3.25939400	2.02156000
H	1.72612300	-3.97500200	2.51677200
C	-0.30149200	-3.38300600	2.16945800
H	-0.69588600	-4.19173700	2.77293500
C	-1.14584600	-2.47645300	1.53817700
C	-0.61685200	-1.42557400	0.75137800
C	3.86363600	-2.95221800	1.69874300
H	3.71724300	-3.98339100	1.35012800
H	3.76955500	-2.92240600	2.79295400
H	4.86060200	-2.61257700	1.41146700
C	-3.11050500	-3.57650100	2.36141100
H	-2.84574400	-4.56299300	1.95703200
H	-4.18816300	-3.42812300	2.27396700
H	-2.82174200	-3.52891200	3.42039700
C	-1.64124900	-0.54096200	0.07017200
C	-2.33153600	-1.06419000	-1.08501700
C	-3.49985000	-0.44727400	-1.51364600
H	-4.01657400	-0.84864000	-2.38152900
C	-4.03368700	0.68959500	-0.87982900
C	-3.36933700	1.19901000	0.22988800
H	-3.78069700	2.05974900	0.74692800
C	-2.19432900	0.60079600	0.75598500
C	-1.82017500	-2.29637100	-1.82172900
H	-0.85175900	-2.56399000	-1.39454900
C	-2.77509000	-3.49026800	-1.62757700
H	-2.91999900	-3.70931700	-0.56591300
H	-2.37118700	-4.38417200	-2.11782400
H	-3.75907400	-3.28317200	-2.06579800
C	-1.58760500	-2.01062900	-3.31644400
H	-2.52425400	-1.77008500	-3.83364200
H	-1.15837400	-2.89490600	-3.80307900
H	-0.89289900	-1.17678700	-3.43925600
C	-5.29862100	1.34691100	-1.40946800
H	-5.65887600	0.73454800	-2.24724800
C	-6.41458300	1.38617000	-0.34935300
H	-6.63687100	0.38191800	0.02804600
H	-7.33438500	1.80648100	-0.77313800
H	-6.12777800	2.00991400	0.50563200
C	-5.00385100	2.75688500	-1.95565000
H	-4.64511600	3.41749100	-1.15731600
H	-5.90847100	3.20528900	-2.38367300
H	-4.23312100	2.72449000	-2.73370600

C	-1.74632100	0.99873500	2.16446200
H	-0.75531500	0.56582100	2.33506700
C	-2.70237400	0.39173300	3.21160000
H	-3.70956200	0.81153700	3.10011600
H	-2.35040100	0.61736700	4.22548800
H	-2.77591300	-0.69162200	3.09598800
C	-1.64304700	2.52044500	2.36463600
H	-1.01902400	2.98918100	1.59732400
H	-1.20718600	2.74121200	3.34566300
H	-2.62859400	2.99957100	2.33197300
F	0.83047500	-0.77457900	-1.86497600
Pd	-0.59919100	1.17062700	-0.87815500
F	1.11415500	1.84407700	-1.88224700

TS11

P	1.54114300	-0.20829900	-0.25899600
O	2.79199900	2.34542200	-1.32365600
O	-2.65083300	3.04419900	-0.70147300
C	1.65279900	-2.25755800	-0.32515900
H	0.62671000	-2.68735100	-0.19058000
C	2.48386700	-2.89018600	0.80404300
H	3.53834400	-2.60520500	0.69428100
H	2.13158200	-2.52312500	1.77027800
C	2.38448200	-4.42598900	0.73784800
H	2.99731300	-4.86710000	1.53441100
H	1.34513700	-4.72817400	0.93587400
C	2.81883500	-4.95895200	-0.63447300
H	2.71329200	-6.05063400	-0.67413300
H	3.88663300	-4.73897600	-0.78313700
C	2.01049500	-4.30274400	-1.76229200
H	0.95583900	-4.60685600	-1.67751300
H	2.36023100	-4.65440300	-2.74168000
C	2.09406000	-2.76571500	-1.70830000
H	3.12517600	-2.44621600	-1.90218000
H	1.47839600	-2.33155300	-2.49944300
C	3.06277000	0.17349900	0.76081400
H	3.04734600	-0.63664700	1.48709600
C	4.37109500	0.05321300	-0.04564200
H	4.41851800	0.83432800	-0.80619000
H	4.40743300	-0.90328100	-0.57993900
C	5.57886600	0.16384100	0.90147900
H	5.58933500	-0.70405500	1.57771300
H	6.50957300	0.12051500	0.32058900
C	5.52631700	1.44963100	1.73989000

H	5.59996300	2.31998600	1.06967600
H	6.39036600	1.49788300	2.41516100
C	4.21497200	1.53684100	2.53483800
H	4.18053500	2.46765200	3.11566200
H	4.17546300	0.71136100	3.26052800
C	2.99158300	1.45838400	1.60656200
H	2.06880700	1.43843700	2.19114800
H	2.97715200	2.34597500	0.97012000
C	0.67635400	1.52090500	-0.58841200
C	1.45870200	2.57609600	-1.13763400
C	0.87475800	3.79668100	-1.46870200
H	1.47644300	4.60500800	-1.86540100
C	-0.49764300	3.99537700	-1.32091700
H	-0.92626700	4.94955400	-1.60294300
C	-1.28741200	2.96222200	-0.83730000
C	-0.70942400	1.72385800	-0.46198200
C	3.63143600	3.41421600	-1.71968200
H	3.58686500	4.25004000	-1.00783500
H	3.37902300	3.77842400	-2.72456300
H	4.64421400	3.00652900	-1.73138200
C	-3.28745000	4.26742000	-1.02843400
H	-2.92044700	5.09241600	-0.40258500
H	-4.34956400	4.11064500	-0.83201000
H	-3.14835400	4.52553200	-2.08735500
C	-1.67975600	0.67201800	0.00098600
C	-2.39141900	0.85958300	1.24315700
C	-3.44781400	0.01661300	1.54047600
H	-3.96293300	0.13094800	2.49093100
C	-3.86271100	-1.01943900	0.67229200
C	-3.21352000	-1.16868100	-0.54478000
H	-3.55281000	-1.93062300	-1.23974200
C	-2.13332000	-0.32796100	-0.94978100
C	-1.94780600	1.91189900	2.25146700
H	-1.16707400	2.51922500	1.78312900
C	-3.09672300	2.85832400	2.64197600
H	-3.54188200	3.32044300	1.75551900
H	-2.72565800	3.65212900	3.30148500
H	-3.88860400	2.32637500	3.18246700
C	-1.31828300	1.24655200	3.49229200
H	-2.05669100	0.62538500	4.01529000
H	-0.96828900	2.01412500	4.19469800
H	-0.47557600	0.61205600	3.20142200
C	-4.99541900	-1.95113300	1.07720700
H	-5.36771700	-1.60945300	2.05269100

C	-6.17282600	-1.89417900	0.08671500
H	-6.54487000	-0.86974900	-0.02495300
H	-7.00039300	-2.52589700	0.43143900
H	-5.87278000	-2.25133600	-0.90553000
C	-4.49018600	-3.39578600	1.25313600
H	-4.11559700	-3.79552300	0.30302600
H	-5.29655600	-4.05289300	1.60099300
H	-3.67129100	-3.43917900	1.98005600
C	-1.77972100	-0.24367000	-2.43782400
H	-0.83649800	0.30282100	-2.52965800
C	-2.86480400	0.56694700	-3.17436500
H	-3.82655700	0.03995900	-3.14053300
H	-2.59293300	0.71419600	-4.22665200
H	-3.00288500	1.54608400	-2.70633700
C	-1.57345800	-1.61408900	-3.10002600
H	-0.81586900	-2.19851500	-2.56773500
H	-1.24138200	-1.48355600	-4.13653700
H	-2.49784000	-2.20322000	-3.12613900
F	0.85646400	-0.60460800	1.98786300
Pd	-0.48737700	-1.09433100	0.32642400
F	2.08134600	-0.14161500	-1.81557000

TS12

P	-1.38969900	-0.11295200	-0.28065400
O	-2.87437900	2.48708700	-0.17299200
O	2.58259900	3.12271700	0.40957100
C	-1.75575800	-0.55472400	1.54409700
H	-0.74457500	-0.55321600	1.96057100
C	-2.31103400	-1.97792400	1.76412800
H	-3.33987400	-2.04456300	1.39156400
H	-1.70660700	-2.70336300	1.21388000
C	-2.31420600	-2.32808100	3.26243900
H	-2.74215600	-3.32867300	3.40414700
H	-1.27649900	-2.37892300	3.62077300
C	-3.08726000	-1.29247300	4.09028000
H	-3.04531100	-1.54178300	5.15858000
H	-4.14904700	-1.31811200	3.80133800
C	-2.53957300	0.12099100	3.85152800
H	-1.50577200	0.17791600	4.22385100
H	-3.11948800	0.86084500	4.41945900
C	-2.55505300	0.49121200	2.35669400
H	-3.59283000	0.53237100	2.01091300
H	-2.13359100	1.49302800	2.22740400
C	-2.98975700	-0.34788600	-1.23737300

H	-2.92846500	0.42768100	-2.00512500
C	-2.93814100	-1.72937400	-1.94100300
H	-2.97920900	-2.52765500	-1.18799700
H	-1.99287500	-1.84354300	-2.47542100
C	-4.11303800	-1.87570300	-2.92167000
H	-3.99322700	-1.13989100	-3.73062400
H	-4.07764600	-2.86778600	-3.38903900
C	-5.46658500	-1.65507700	-2.23343300
H	-5.63686900	-2.46085000	-1.50414900
H	-6.28361900	-1.71211700	-2.96405500
C	-5.49411300	-0.30605400	-1.50217700
H	-6.44794400	-0.17415000	-0.97481500
H	-5.42319000	0.50992200	-2.23717800
C	-4.33204500	-0.18653900	-0.49925900
H	-4.37946900	0.77403600	0.01332000
H	-4.45010000	-0.96610800	0.25939800
C	-0.69916500	1.59805900	-0.05072200
C	-1.53850200	2.72814400	0.00319400
C	-1.00400400	3.99558700	0.22089000
H	-1.64258000	4.86933200	0.26945900
C	0.37468900	4.15562200	0.37389400
H	0.77060700	5.14949900	0.54405100
C	1.21825600	3.05074300	0.29176300
C	0.69292600	1.75834100	0.05991500
C	-3.78345000	3.57134300	-0.08712800
H	-3.58687000	4.32321000	-0.86280900
H	-3.74769200	4.04917100	0.90122700
H	-4.77457400	3.14213800	-0.24548400
C	3.17722500	4.39533900	0.60730000
H	2.96549200	5.07256200	-0.23122800
H	4.25193900	4.21455100	0.66360000
H	2.83698200	4.85823000	1.54366000
C	1.69218600	0.63506300	-0.09678200
C	2.28544400	0.39428400	-1.37283800
C	3.31951200	-0.55429600	-1.46514700
H	3.75399200	-0.76010700	-2.43901300
C	3.82526900	-1.22257000	-0.35016800
C	3.28742000	-0.90995700	0.90539200
H	3.68139700	-1.40973800	1.78432400
C	2.23887500	-0.00438400	1.06047900
C	1.93429100	1.21990800	-2.60608700
H	0.99550800	1.73654600	-2.40637300
C	3.03303700	2.27579500	-2.84634000
H	3.18024600	2.90024100	-1.96090600

H	2.76170100	2.92067200	-3.69098400
H	3.99063700	1.79516300	-3.08258800
C	1.71906000	0.36379400	-3.86518200
H	2.64697000	-0.11461800	-4.20144000
H	1.36563300	0.99856300	-4.68629800
H	0.96756800	-0.40630400	-3.67964000
C	4.90010700	-2.28564600	-0.49712900
H	5.20528800	-2.30150800	-1.55238800
C	6.14928300	-1.97117400	0.34551100
H	6.56282900	-0.98902000	0.09076700
H	6.92545800	-2.72659700	0.17610600
H	5.91708400	-1.97067100	1.41698800
C	4.33113500	-3.67621500	-0.15131000
H	4.02044900	-3.72060100	0.89909200
H	5.08533900	-4.45519200	-0.31545600
H	3.45215600	-3.90784200	-0.76269900
C	1.73157800	0.29977400	2.46423500
H	0.78167100	0.83578800	2.36921400
C	2.70826700	1.23328400	3.20663800
H	3.68277200	0.74621200	3.33276000
H	2.32057900	1.47958600	4.20262700
H	2.86413300	2.15989400	2.64838400
C	1.47785400	-0.98220300	3.28105300
H	0.91832200	-1.72261700	2.70065900
H	0.91203000	-0.74698600	4.19001500
H	2.41845700	-1.44898200	3.59488000
F	-0.65524400	0.13307100	-2.08557100
Pd	0.33925400	-1.46648900	-0.64907500
F	-0.12358600	-3.25233100	0.07815100

TS13A

P	2.04083800	0.85409300	0.14871900
C	3.36602400	-0.43812200	0.27846400
C	4.74815600	-0.11528500	0.27787300
C	5.70903200	-1.12094300	0.32852400
H	6.76525000	-0.88057800	0.32939900
C	5.32258500	-2.46105600	0.36631000
H	6.08852900	-3.22666600	0.39814200
C	3.97274800	-2.79821800	0.35470000
C	2.97645100	-1.79076300	0.32181900
C	1.55333400	-2.29845300	0.31338300
C	0.93714600	-2.67960900	1.54260700
C	-0.20165100	-3.48958800	1.50552600
H	-0.70074300	-3.73805900	2.43605900

C	-0.73177000	-3.97859500	0.31258100
C	-0.10357800	-3.62191700	-0.88547000
H	-0.50974500	-3.98432800	-1.82358700
C	1.01541700	-2.79497900	-0.91698100
C	1.53708000	-2.29100100	2.88824800
H	2.20083300	-1.43938800	2.71489700
C	2.39699600	-3.43987200	3.45475900
H	1.77876200	-4.33032300	3.61949700
H	2.83208400	-3.14617900	4.41782100
H	3.20686200	-3.71620900	2.77600400
C	0.47836000	-1.86537500	3.92247400
H	-0.27570600	-1.21148800	3.48086500
H	0.96546100	-1.35325800	4.76107900
H	-0.04286700	-2.73719700	4.33585200
C	-1.94039300	-4.89889100	0.33397700
H	-2.44801500	-4.73531000	1.29176800
C	-2.95536600	-4.59322500	-0.77942800
H	-2.55339300	-4.83463100	-1.77125600
H	-3.23512600	-3.53820900	-0.76234900
H	-3.85741000	-5.19997600	-0.63832000
C	-1.47838000	-6.36902700	0.27693100
H	-2.33713600	-7.04842400	0.33779500
H	-0.79428600	-6.60433400	1.10049300
H	-0.95230200	-6.57163100	-0.66461500
C	1.67034800	-2.48130900	-2.25618000
H	2.42248700	-1.70673600	-2.08488700
C	2.39756800	-3.71804900	-2.81819100
H	3.13973900	-4.09330300	-2.10838100
H	2.90318100	-3.47209100	-3.75989900
H	1.68543600	-4.52753200	-3.01754600
C	0.66257100	-1.92721600	-3.27881700
H	-0.05246400	-2.69401900	-3.59631300
H	1.18658500	-1.56882800	-4.17293000
H	0.09101900	-1.09826700	-2.85170600
C	2.05451900	1.98708300	1.64169000
H	1.27166300	2.69753400	1.34321800
C	3.33100100	2.79336300	1.94409700
H	3.71528300	3.28362700	1.04458000
H	4.11614100	2.10975700	2.28660800
C	3.04638600	3.83484900	3.04061700
H	3.95919300	4.40621700	3.25345400
H	2.30335900	4.55630800	2.66922000
C	2.51858700	3.17225200	4.32144100
H	3.30949300	2.53630700	4.74680800

H	2.28526700	3.93279500	5.07693100
C	1.28361200	2.30808200	4.02987600
H	0.45014100	2.95490200	3.71839100
H	0.95321000	1.78691200	4.93661200
C	1.55970300	1.28285600	2.91893000
H	2.32906700	0.57832800	3.26187500
H	0.65798000	0.69757100	2.71668500
C	2.55100600	1.94600500	-1.29139700
H	3.62105000	2.12052000	-1.14699400
C	2.36454500	1.19591800	-2.62419300
H	1.30943800	0.93030900	-2.74750100
H	2.92909400	0.25661300	-2.60762300
C	2.81407000	2.04995600	-3.81955300
H	3.89998400	2.21785500	-3.76022800
H	2.63034700	1.50081200	-4.75181900
C	2.09292100	3.40417100	-3.83724400
H	1.01451000	3.23910400	-3.97602800
H	2.43497700	4.01004300	-4.68561800
C	2.31838100	4.15233700	-2.51704200
H	1.78837500	5.11276100	-2.52380800
H	3.38962200	4.38141900	-2.40898500
C	1.84687600	3.32119300	-1.31192900
H	2.05433700	3.87493800	-0.39034100
H	0.76358200	3.19337900	-1.36826200
C	6.42892500	1.60407000	0.34076700
H	6.86066600	1.25187800	1.28678400
H	6.42177000	2.69544700	0.32992000
H	7.03640000	1.23643500	-0.49662900
C	4.45269900	-5.14536800	0.43548500
H	5.11595200	-5.15114600	-0.44029300
H	3.86244000	-6.06280100	0.44736900
H	5.05782100	-5.09041200	1.35073800
O	5.07207200	1.21151700	0.21328000
O	3.51218200	-4.08578900	0.36962300
Pd	0.08903900	-0.27417500	0.11756600
C	-0.84795500	1.42687100	-0.34369000
C	-1.24990900	1.62853000	-1.66906300
C	-1.23249400	2.34345600	0.63939100
C	-1.99853000	2.75648500	-2.02074500
H	-0.99259000	0.91096600	-2.44175000
C	-1.97319200	3.47908900	0.29864200
H	-0.98765300	2.16302200	1.68048000
C	-2.33446800	3.67102200	-1.02948400
H	-2.32030100	2.92984600	-3.04290000

H	-2.28496800	4.20005600	1.04789100
F	-3.04077800	4.77494900	-1.36659800
O	-1.66864500	-0.62948800	1.78985600
C	-2.56052800	-1.01861700	1.01961400
F	-3.18225700	-2.21892100	1.30186300
F	-3.57904900	-0.15900600	0.70507000
F	-1.98430900	-1.30438800	-0.35729300

TS13B

Pd	9.30264700	11.13802400	9.76381700
P	7.07428100	10.87824800	9.33171300
F	11.17771600	10.89630500	7.28724000
F	12.83173400	11.82547100	8.36234700
F	12.82875600	9.67446000	7.99579700
F	9.56461800	5.29439700	10.36909800
O	11.45318300	10.56492700	9.50889100
O	4.12486800	11.53067700	9.10637100
O	7.15381200	15.92215800	10.53805900
C	12.04812300	10.72393100	8.33081300
C	10.35170300	9.29297500	9.67355500
C	10.10046000	8.42817600	8.58473900
H	10.05187400	8.81871000	7.57622300
C	9.82703300	7.08473000	8.83384000
H	9.56321200	6.41379700	8.02203000
C	9.87393600	6.59547200	10.13565500
C	10.23905800	7.41127700	11.20383200
H	10.30607600	6.98955900	12.20179700
C	10.52449700	8.75325800	10.96993600
H	10.84300400	9.40277300	11.77871900
C	6.84972400	10.25169000	7.57083900
H	7.56095800	9.41091800	7.57876900
C	7.38184700	11.27434900	6.55297000
H	6.70741400	12.14269600	6.52658500
H	8.36228800	11.63655600	6.87161100
C	7.48537600	10.66166000	5.14789300
H	7.83179000	11.42125000	4.43539600
H	8.25124200	9.87178900	5.16159200
C	6.14730900	10.06285300	4.69164900
H	6.25544000	9.58517500	3.70958500
H	5.41376300	10.87409900	4.56989400
C	5.61258400	9.05753900	5.72250200
H	6.29538500	8.19647900	5.77903500
H	4.63832800	8.66479100	5.40331500
C	5.48649000	9.69898500	7.11596700

H	4.75688400	10.51389600	7.07583700
H	5.10020000	8.96384400	7.83078400
C	6.10655300	9.65193000	10.36257700
H	5.09396300	9.58012700	9.95604000
C	6.80201800	8.27979200	10.25940600
H	7.84537400	8.38894400	10.56713100
H	6.82061300	7.92867800	9.22160600
C	6.13038900	7.22266500	11.14765500
H	5.11591100	7.01534700	10.77480600
H	6.69612800	6.28492100	11.07774600
C	6.04853200	7.69395600	12.60575400
H	7.06875900	7.80098000	13.00367500
H	5.54308700	6.94264300	13.22574500
C	5.32694500	9.04577100	12.70458600
H	5.29523300	9.39006200	13.74654200
H	4.28242600	8.92452100	12.37993100
C	6.01177800	10.10674700	11.82886400
H	5.47040800	11.05879000	11.89548700
H	7.02391600	10.28487200	12.21044600
C	6.22795000	12.52488500	9.53696000
C	4.82056900	12.66108800	9.43743000
C	4.19862400	13.88260100	9.68011400
H	3.12332400	13.98811100	9.60168700
C	4.96018700	14.99452600	10.04139000
H	4.45591200	15.93365800	10.23479300
C	6.34186900	14.88405300	10.15884900
C	6.99751000	13.65486700	9.89849900
C	2.71188500	11.58796100	9.01646100
H	2.38361900	12.29207500	8.23997300
H	2.25828200	11.86927200	9.97634700
H	2.39222200	10.57983800	8.74619000
C	6.56379700	17.18419500	10.80055900
H	6.05531800	17.58492300	9.91277200
H	7.38848600	17.84522900	11.07226100
H	5.85060300	17.13207800	11.63476800
C	8.49954500	13.67800500	10.07047400
C	9.33155600	14.14191900	9.01693300
C	10.69704600	14.32932100	9.26249200
H	11.33891000	14.65660200	8.45004800
C	11.27253600	14.09519800	10.51257500
C	10.43408000	13.67323700	11.54770200
H	10.85623800	13.50290900	12.53280500
C	9.06370400	13.45787500	11.35869800
C	8.77225100	14.46150000	7.63544600

H	7.81398900	13.94229200	7.53957300
C	8.50060800	15.97221800	7.48554000
H	7.80938500	16.33167000	8.25063500
H	8.07749400	16.18942300	6.49692900
H	9.43540700	16.53725500	7.58624800
C	9.68692500	13.97897900	6.49347500
H	10.56186500	14.62987800	6.38003200
H	9.14283500	13.99902000	5.54191000
H	10.05181900	12.96372400	6.66733900
C	12.75896600	14.31472600	10.74295300
H	13.22084700	14.47391100	9.75948600
C	12.99710600	15.58111800	11.58811200
H	12.55067100	16.46309200	11.11508900
H	14.07008100	15.76606200	11.72026000
H	12.54882900	15.47241900	12.58350000
C	13.44070900	13.09023900	11.38021600
H	13.06008800	12.90339500	12.39164700
H	14.52168400	13.25639900	11.46113100
H	13.26857600	12.19302800	10.78075200
C	8.20361100	13.06920800	12.55717700
H	7.24254200	12.71082900	12.17837900
C	7.92496800	14.30283300	13.43889200
H	8.85907600	14.69063300	13.86354600
H	7.25670300	14.04241500	14.26895400
H	7.46543200	15.10462300	12.85467600
C	8.81842500	11.93250000	13.39333800
H	9.08150500	11.07755900	12.76087600
H	8.10480200	11.59529500	14.15457600
H	9.72551800	12.25657100	13.91653300

TS13

P	1.48151300	0.06099800	-0.82414300
O	2.96928800	-0.49976600	1.52754000
O	-2.28645300	0.12373300	3.05615000
C	2.11585200	-1.73850000	-1.03097200
H	1.64193600	-1.95752000	-1.99715000
C	1.56913300	-2.76840000	-0.02963400
H	2.05963700	-2.64235900	0.94013100
H	0.50031800	-2.61900400	0.13263700
C	1.81848400	-4.20004800	-0.53179900
H	1.42614000	-4.92010200	0.19788400
H	1.25913200	-4.36246000	-1.46488900
C	3.31242100	-4.44521000	-0.78196300
H	3.47784200	-5.45819900	-1.17036400

H	3.85103400	-4.38166300	0.17565500
C	3.88181500	-3.39990000	-1.75000100
H	3.42048000	-3.53388400	-2.73944400
H	4.96076300	-3.54997200	-1.88541900
C	3.61938400	-1.96261400	-1.26367200
H	4.16531400	-1.79677700	-0.32820500
H	3.99762300	-1.25450500	-2.00448300
C	2.69190300	1.50581100	-0.40931600
H	2.64303100	1.62249600	0.67545300
C	4.15345500	1.20462400	-0.79298800
H	4.21556200	1.07366800	-1.87987600
H	4.48725500	0.27539800	-0.33251500
C	5.09845600	2.33876000	-0.36282400
H	5.09012300	2.41893800	0.73537200
H	6.12759400	2.08666600	-0.65100500
C	4.67789500	3.68031900	-0.97012400
H	4.77864300	3.62756400	-2.06409400
H	5.34033400	4.48608800	-0.62804400
C	3.22062300	3.98635500	-0.61038800
H	2.89611000	4.92431600	-1.07925200
H	3.14418300	4.13907500	0.47725400
C	2.26489300	2.85593900	-1.02878200
H	1.25402900	3.11415700	-0.71507200
H	2.24470800	2.76219900	-2.11996800
C	0.70977200	-0.05358700	0.95829400
C	1.68267400	-0.28408600	1.96925000
C	1.35747300	-0.32405200	3.31840600
H	2.11883200	-0.49034300	4.07068300
C	0.03130300	-0.16850000	3.71893100
H	-0.21026000	-0.20413500	4.77424900
C	-0.95258700	0.00575100	2.75556800
C	-0.63420000	0.06170700	1.37141000
C	4.02918000	-0.53773300	2.46681600
H	3.95289800	-1.41099500	3.12837000
H	4.06124200	0.37672900	3.07371600
H	4.94571300	-0.61376900	1.87892500
C	-2.67862500	0.07854100	4.41738000
H	-2.41612700	-0.88186500	4.88236500
H	-3.76398400	0.19382700	4.41598600
H	-2.22818300	0.89655600	4.99641600
C	-1.82994300	0.19682900	0.46612400
C	-2.56533700	-0.95741600	0.11457100
C	-3.69563700	-0.82175300	-0.69468100
H	-4.24289000	-1.71405700	-0.98628300

C	-4.13614400	0.42342600	-1.14282100
C	-3.43714900	1.55675000	-0.72037800
H	-3.79177700	2.53328000	-1.03832400
C	-2.30244700	1.47466300	0.08742700
C	-2.18557700	-2.34963000	0.60863400
H	-1.22582100	-2.27408100	1.12775400
C	-3.21297100	-2.85530200	1.63960600
H	-3.30613100	-2.14549200	2.46652800
H	-2.91068600	-3.82990800	2.04270000
H	-4.20230900	-2.97149600	1.18103400
C	-2.01906700	-3.36279300	-0.53946900
H	-2.97715900	-3.57690900	-1.02614400
H	-1.62305100	-4.31136000	-0.15753900
H	-1.33625200	-2.99166100	-1.31022900
C	-5.33491600	0.54733400	-2.06926400
H	-5.49782000	1.61793700	-2.25468700
C	-5.05219800	-0.12295700	-3.42688900
H	-4.14408400	0.28449500	-3.88369100
H	-5.88957900	0.02890500	-4.11884600
H	-4.90640400	-1.20315900	-3.30665100
C	-6.61790900	-0.01443800	-1.43087500
H	-7.47785700	0.13126200	-2.09573100
H	-6.83299600	0.47891800	-0.47656500
H	-6.52517100	-1.08979900	-1.23777100
C	-1.63324200	2.75251000	0.58149900
H	-0.61203500	2.49749500	0.87821900
C	-2.36808900	3.28171600	1.82991800
H	-3.39661100	3.56752900	1.57628400
H	-1.85968800	4.16760500	2.23077000
H	-2.41694600	2.51940600	2.61196600
C	-1.53626500	3.85029100	-0.49108200
H	-1.08852500	3.45692800	-1.40603600
H	-0.91467100	4.67582300	-0.12351500
H	-2.51885100	4.27200200	-0.73461800
C	-0.19282500	-0.27852800	-1.74559100
F	1.88524200	0.30200700	-2.48367600
F	0.04889500	1.41786300	-1.33891700
O	-0.99184500	-0.54557300	-2.52999000
